# Association of eplet mismatch load and allograft injury in kidney transplant recipients

**DOI:** 10.1093/ckj/sfag341

**Published:** 2026-09-26

**Authors:** Fruzsina Toth, Kendon J Holdaway, Divya Raghavan, Silviana Marineci, Suayp Oygen, Katalin Fornadi, Miklos Z Molnar

**Affiliations:** Department of Internal Medicine, Division of Nephrology & Hypertension, Spencer Fox Eccles School of Medicine at the University of Utah, Salt Lake City, UT, USA; Doctoral College, Theoretical and Translational Medicine Division, Semmelweis University, Budapest, Hungary; Department of Internal Medicine, Division of Nephrology & Hypertension, Spencer Fox Eccles School of Medicine at the University of Utah, Salt Lake City, UT, USA; Department of Internal Medicine, Division of Nephrology & Hypertension, Spencer Fox Eccles School of Medicine at the University of Utah, Salt Lake City, UT, USA; Department of Internal Medicine, Division of Nephrology & Hypertension, Spencer Fox Eccles School of Medicine at the University of Utah, Salt Lake City, UT, USA; Department of Internal Medicine, Division of Nephrology & Hypertension, Spencer Fox Eccles School of Medicine at the University of Utah, Salt Lake City, UT, USA; Department of Surgery, Division of Transplantation and Advanced Hepatobiliary Surgery, Spencer Fox Eccles School of Medicine at the University of Utah, Salt Lake City, UT, USA; Transplant Institute, University of Rochester Medical Center, Rochester, NY, USA; Division of Nephrology, Department of Medicine, University of Rochester Medical Center, Rochester, NY, USA; Department of Surgery, University of Rochester Medical Center, Rochester, NY, USA

**Keywords:** donor-derived cell-free DNA, donor-specific antibody, eplet mismatch, graft rejection, kidney transplantation

## Abstract

**Introduction:**

Eplet mismatch load is a molecular measure of donor–recipient histocompatibility that has been associated with alloimmune outcomes after kidney transplantation. However, its relationship with comprehensive markers of early allograft injury remains incompletely defined.

**Methods:**

In this single-center retrospective cohort study, we evaluated 657 adult kidney transplant recipients who underwent transplantation between January 2021 and December 2024. Eplet mismatch load was calculated at the HLA-A, HLA-B, HLA-C, HLA-DRB1, HLA-DQA1, and HLA-DQB1 loci using the HLA Eplet Registry. The primary outcome was a composite of early allograft injury, defined as the independent occurrence of de novo donor-specific antibodies, histologic rejection, molecular rejection assessed by the Molecular Microscope Diagnostic System, or elevation of donor-derived cell-free DNA within the first year after transplantation.

**Results:**

During the first post-transplant year, 248 patients (37.7%) experienced the primary outcome. Median eplet mismatch load was significantly higher among patients with the primary outcome across most HLA loci. In adjusted Cox regression models, higher eplet mismatch load (per one-eplet increase) was independently associated with increased risk of the primary outcome for HLA-DRB1 (hazard ratio 1.025; 95% confidence interval 1.011–1.038), HLA-DQA1 (hazard ratio 1.075; 95% confidence interval 1.025–1.127), and HLA-DQB1 (hazard ratio 1.038; 95% confidence interval 1.020–1.057). Compared with zero eplet mismatch, high-risk class II eplet mismatch categories were associated with approximately two- to three-fold higher risk of early allograft injury. Associations for class I loci were weaker and pattern-dependent after adjustment.

**Conclusion:**

In conclusion, a higher class II HLA-eplet mismatch load was independently associated with early kidney allograft injury during the first post-transplant year.

## Graphical Abstract

**Graphical Abstract:**
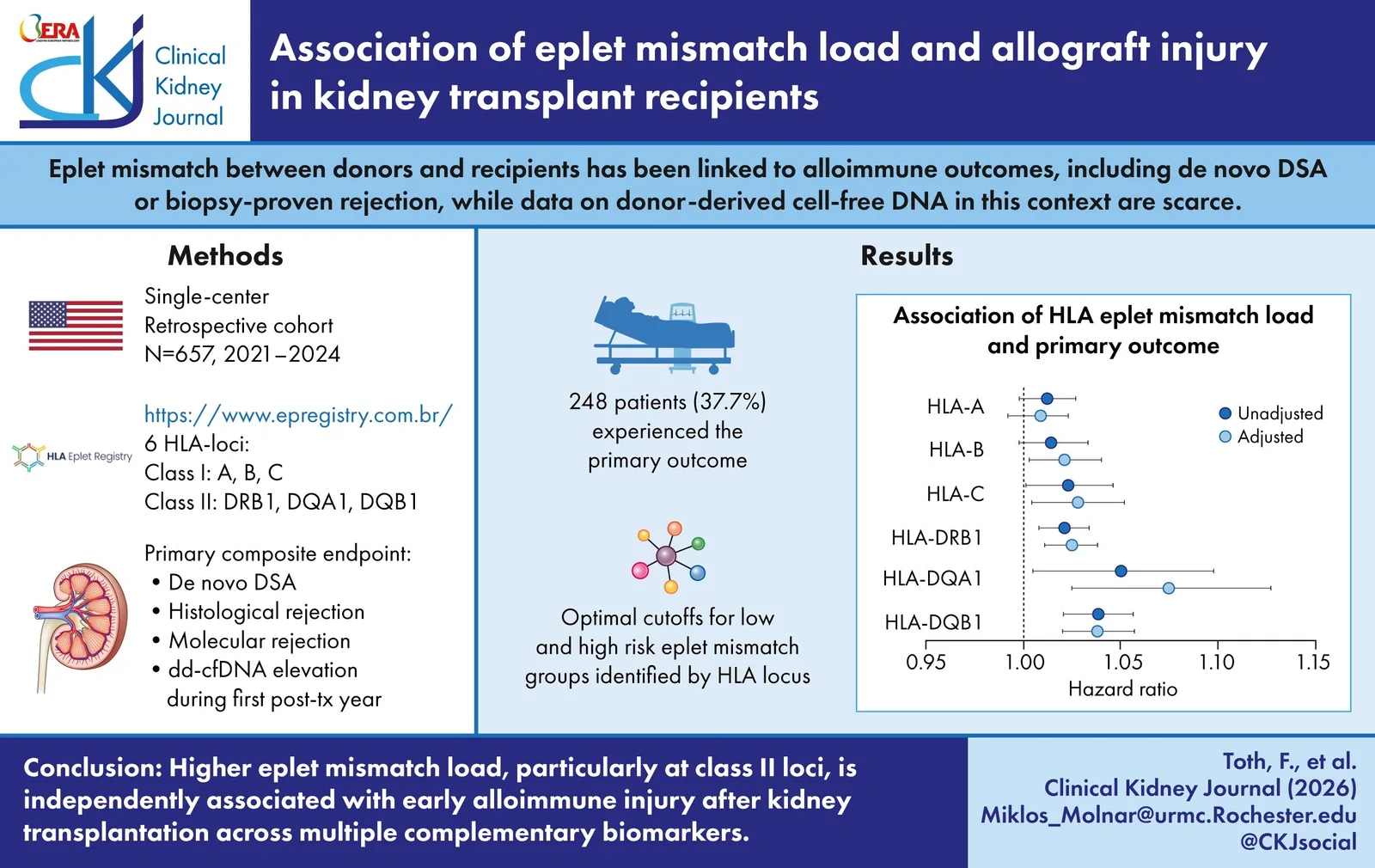

**ABBREVIATIONS**
AUC: area under the curve
ABMR: antibody-mediated rejection
cPRA: calculated panel reactive antibody
CI: confidence interval
CIT: cold ischemia time
DBD: deceased donation after brain death
DCD: donation after circulatory death
dd-cfDNA: donor-derived cell-free DNA
DSA: donor-specific antibody
dnDSA: de novo donor-specific antibodies
HR: hazard ratio
HLA: human leukocyte antigen
IQR: interquartile range
MFI: mean fluorescence intensity
MMDx: Molecular Microscope® Diagnostic System
POD: postoperative days
REDCaP: Research Electronic Data Capture
ROC: receiver operating characteristic
SD: standard deviation
TCMR: T cell–mediated rejection

KEY LEARNING POINTS **What was known:**

- Eplets are small structural units on the surface of the HLA molecule; in transplantation, eplet mismatch load analysis can be used to better assess immunologic risk between donors and recipients.
- Multiple cohorts have quantified donor-recipient HLA eplet mismatch and linked it to alloimmune outcomes, including de novo DSA or biopsy-proven rejection. However, data on donor-derived cell-free DNA in this context are scarce.
- Most studies focus on class II epitope mismatch; therefore, the role of class I epitope mismatch remains controversial.

**This study adds:**

- In this single-center, retrospective study, we evaluated the association between eplet mismatch load and kidney allograft injury using complementary biomarkers, including dd-cfDNA, de-novo DSA, and histologic and molecular rejection signatures assessed by MMDx.
- This study aimed to assess the performance characteristics of the eplet mismatch load in predicting alloimmune injury outcomes.

**Potential impact:**

- Our findings suggest that broader implementation of eplet-based risk stratification may meaningfully refine immunologic risk assessment and inform decision-making.
- Our findings indicate that eplet mismatch load—particularly at class II loci—is associated with global susceptibility to alloimmune injury, consistent with real-world clinical practice, where graft injury is often multifactorial.

## INTRODUCTION

An eplet is a small cluster of amino acids on the surface of a human leukocyte antigen (HLA) molecule. It represents the smallest functional unit of an epitope that contributes to immune recognition. In transplantation, eplet mismatch load analysis can be used to better assess immunologic risk between donors and recipients.

Across more than twenty adult kidney transplant cohorts, donor–recipient HLA eplet mismatch has been quantified (mostly via HLAMatchmaker on high-resolution HLA typing) and related to alloimmune outcomes [1–22]. Most studies focus on de novo donor-specific antibodies (dnDSA). Higher class II eplet mismatch, particularly HLA-DR/DQ, consistently predicts dnDSA, with DQ usually the dominant locus [2–4, 6, 7, 9–17, 23]. Simple thresholds define low- versus high-risk strata, although some work shows a near-linear increase in dnDSA risk with rising DQ antibody-verified eplet load and no evident safe cutoff [3, 13, 23].

Multiple cohorts link eplet burden to biopsy-proven rejection and microvascular injury. Higher DR/DQ eplet mismatch predicts antibody-mediated rejection (ABMR), some T-cell-mediated rejection (TCMR), and transplant glomerulopathy, outperforming traditional antigen mismatch [2, 5, 11, 15, 24]. Eplet and broader molecular mismatch scores are also associated with ABMR-like histology even when circulating donor-specific antibody (DSA) is absent [6, 25].

Several large, adjusted cohorts report that higher class II—particularly DQ and antibody-verified—eplet load independently predicts graft loss, often mediated by dnDSA, ABMR, and transplant glomerulopathy [2, 3, 20, 24, 26]. However, data on donor-derived cell-free DNA (dd-cfDNA) in this context are almost nonexistent. Among all retrieved kidney studies, only one small, single-center prospective cohort formally tested the relationship between eplet load and dd-cfDNA [2]. However, the study included only one early time point, had limited power, and included no modeling of longitudinal trajectories or mediation through dnDSA/ABMR [1]. No larger cohorts validate these findings. No study stratifies dd-cfDNA performance by eplet-risk categories or integrates dd-cfDNA with antibody-verified eplet hierarchies. Emerging evidence from several other kidney-transplant cohorts nonetheless supports dd-cfDNA as a marker of clinically important graft injury independent of concurrent biopsy-proven rejection, including associations with subsequent rejection risk, eGFR decline, and graft failure [27–31].

The role of class I eplet mismatch remains controversial because signals weaken or disappear once robust class II modeling is introduced. Early and modest-sized cohorts using mostly low-resolution typing suggested that higher HLA-A/B eplet load could associate with anti–HLA-I antibodies, acute rejection, or shorter graft survival [5, 19], and a postfailure cohort found electrostatic (not eplet) mismatch at HLA-A/B predicted DSA after graft loss [32]. However, in later high-resolution, multivariable analyses that include DR/DQ, the independent contribution of class I eplets is inconsistent: strong, reproducible effects are seen for class II/DQ, whereas class I either shows no clear association or loses significance when class II is modeled [3, 4, 6, 13, 18]. As a result, many groups now treat class I eplet mismatch as a possible but weak determinant, with ongoing debate about whether it adds actionable prognostic value beyond class II/DQ in adult kidney transplantation.

In this retrospective, single-center study, we evaluated the association between eplet mismatch load and kidney allograft injury using complementary biomarkers such as dd-cfDNA, de-novo DSA, histologic rejection, and molecular rejection signatures assessed with the MMDx [33]. We hypothesized that a higher eplet mismatch load is associated with an increased risk of post-transplant de-novo DSA development, a higher incidence of rejection, and elevated levels of dd-cfDNA during the first year following kidney transplantation. Additionally, we sought to assess the performance characteristics of eplet mismatch load for predicting these alloimmune injury outcomes and compare prediction performance compared to traditional HLA antigen matching.

## MATERIALS AND METHODS

### Data source and study population

This single-center, retrospective, observational study included all adult kidney transplant recipients who underwent transplantation at the University of Utah between 1 January 2021, and 31 December 2024. All patients were followed for up to 1 year post-transplantation. Donor-related data were collected, including graft type [living donor, deceased donation after brain death (DBD), or donation after circulatory death (DCD)], demographics, comorbidities, terminal serum creatinine, cause of death, and serologic characteristics.

Recipient-level data included demographics, cause of end-stage renal disease, anthropometric measures, dialysis vintage, prior transplant history, multiorgan transplantation status, serologic data, and transplant-related variables, including induction immunosuppression, HLA-related data, laboratory values, and other relevant clinical parameters. Protocol biopsies are not performed at our center; all kidney allograft biopsies were performed for clinical indications. Histopathologic findings and molecular rejection assessments using the MMDx were collected for all biopsies.

DSA and dd-cfDNA results were also collected. During the study period, DSA testing was performed at 3, 6, 9, and 12 months post-transplant, per institutional protocol or at the treating provider’s discretion. Similarly, dd-cfDNA measurements were obtained at 1, 2, 3, 4, 6, 9, and 12 months post-transplant per protocol or as clinically indicated. Data were extracted from electronic medical records and local transplant databases and managed using the Research Electronic Data Capture (REDCap) system hosted by the University of Utah. The study was approved by the University of Utah Institutional Review Board, which granted a waiver of informed consent (#IRB_00162331).

### Exposure variables/eplet mismatch load

HLA typing was performed using molecular methods, including genomic DNA amplification followed by sequence-based typing and/or sequence-specific oligonucleotide probe hybridization. The proportion of low-resolution typing for either donor or recipient was 14% for HLA-A, 14% for HLA-B, 15% for HLA-C, 4% for HLA-DRB1, 27% for HLA-DQA1, and 1% for HLA-DQB1.

Molecular matching was assessed using eplet mismatch load based on donor–recipient mismatches at the HLA-A, HLA-B, HLA-C, HLA-DRB1, HLA-DQA1, and HLA-DQB1 loci, allowing derivation of both single molecular eplet mismatches and total eplet load. In this study, total eplet load across the abovementioned loci was used as the exposure variable.

Eplet mismatch loads were calculated using the HLA Eplet Registry web service (https://www.epregistry.com.br/; software version accessed February 2025). The HLA Eplet Registry is an online database established under the auspices of the 16th International HLA and Immunogenetics Workshop in 2012. It documents all theoretically defined eplets and their antibody verification status [34], with the aim of reflecting the eplet repertoire incorporated in the widely used HLAMatchmaker software [35]. The Registry has become a pivotal resource in the field of histocompatibility, providing comprehensive information on eplets defined on HLA molecules [36, 37].

### Outcome variables

Three distinct endpoints were evaluated. The first endpoint was the development of de-novo DSA. De novo DSA was defined as DSA detected within 1 year after transplantation with an MFI >2000 that had not been detected either historically or at the time of transplantation. HLA antibody testing was performed using a Luminex-based single-antigen bead assay (LabScreen, One Lambda/Thermo Fisher Scientific).

The second endpoint was the detection of any type of rejection within the first year after transplantation, as determined by histopathological evaluation or molecular rejection assessed using the MMDx [33]. Kidney biopsies were performed only when clinically indicated and were interpreted according to the Banff 2019 classification.

The third endpoint was elevation of dd-cfDNA at any time during the first year after transplantation. dd-cfDNA elevation was defined as either (a) an absolute dd-cfDNA value ≥1.0%, or (b) an absolute dd-cfDNA value ≥0.5% accompanied by a ≥61% relative increase compared with the immediately preceding measurement [38].

The three endpoints are independently defined and non-hierarchical, rather than a single rule requiring biopsy confirmation for every event. The primary endpoint of the study was the occurrence of any of the above events within 12 months after transplantation. Time-to-event was defined as the interval from the date of transplantation to the date of the first occurrence of any endpoint event. Secondary endpoints consisted of each of the three outcomes evaluated independently.

### Statistical analysis

Patient characteristics were summarized using mean ± standard deviation (SD) or median with interquartile range (IQR) for continuous variables and counts with percentages for categorical variables. Comparisons between groups were performed using the Student’s *t*-test or the Mann–Whitney *U* test for continuous variables, and the chi-square test for categorical variables, as appropriate.

To assess the predictive performance of each HLA-locus eplet mismatch load, receiver operating characteristic (ROC) curve analyses were conducted, and the area under the ROC curve (AUC) was calculated for the primary endpoint and each secondary endpoint separately. The Youden Index was calculated to evaluate the discriminatory performance of each HLA loci eplet mismatch load for each outcome. Optimal cutoff values were identified based on the maximum Youden Index, and these thresholds were used to classify patients into “zero-eplet mismatch load,” “low-eplet mismatch load,” and “high-eplet mismatch load” groups.

Associations between each HLA loci eplet mismatch load—both as continuous variables and as categorical risk groups—and clinical outcomes were evaluated using Cox proportional hazards regression models and Kaplan–Meier survival analyses with log-rank testing. The proportional hazards assumption was assessed using scaled Schoenfeld residuals. Variables included in multivariable-adjusted models were selected *a priori* based on biological plausibility, published literature, and clinical relevance, and were required to be available in the study database. Adjustments were made for recipient characteristics (age, sex, race/ethnicity, body mass index at transplantation, cause of end-stage renal disease, dialysis vintage, calculated panel reactive antibody at allocation, history of prior kidney transplantation, and preemptive transplant status), donor characteristics [age, sex, race/ethnicity, and donor type (living vs. deceased)], and transplantation-related factors (induction regimen, cold ischemia time, cytomegalovirus risk status, and kidney-alone versus multiorgan transplantation).

To further address whether eplet-mismatch load outperforms conventional antigen-level HLA mismatch, we conducted a locus-by-locus comparator analysis of the primary outcome. For each locus, antigen-level HLA mismatch was substituted for eplet mismatch load in otherwise identical Cox models (“antigen-only” vs. “eplet-only”), using the same covariate set (a reduced covariate set of recipient age, donor type, and cPRA was used for HLA-C, -DQA1, and -DQB1 given missing antigen-level data). Discrimination was compared using Harrell’s C-statistic (bootstrap-tested, 1000 replications) and area under the receiver operating characteristic curve.

To characterize how the three components of the primary composite endpoint (de novo DSA, rejection, and dd-cfDNA elevation) co-occur within the same patients, we cross-tabulated patients with complete data on all three indicators and classified endpoint-positive patients as “concordant” (≥2 markers) or “discordant” (1 marker). Eplet mismatch load was compared between concordant and discordant patients at each locus using *t*-tests, rank-sum tests, and multivariable logistic regression.

All statistical tests were two-sided, and a *P* value <.05 was considered statistically significant. Statistical analyses were performed using STATA version 19 (StataCorp, College Station, TX).

## RESULTS

### Patient characteristics

Between 1 January 2021, and 31 December 2024, a total of 689 patients underwent kidney transplantation at the University of Utah and were initially included in the study cohort (Fig. 1). Thirty-two patients were excluded: early death (*n* = 3), primary non-function (*n* = 1), early graft loss (*n* = 2), and zero HLA-antigen mismatch (*n* = 26). The final analytic cohort therefore consisted of 657 patients. Overall patient characteristics are shown in Table 1.

**Figure 1:**
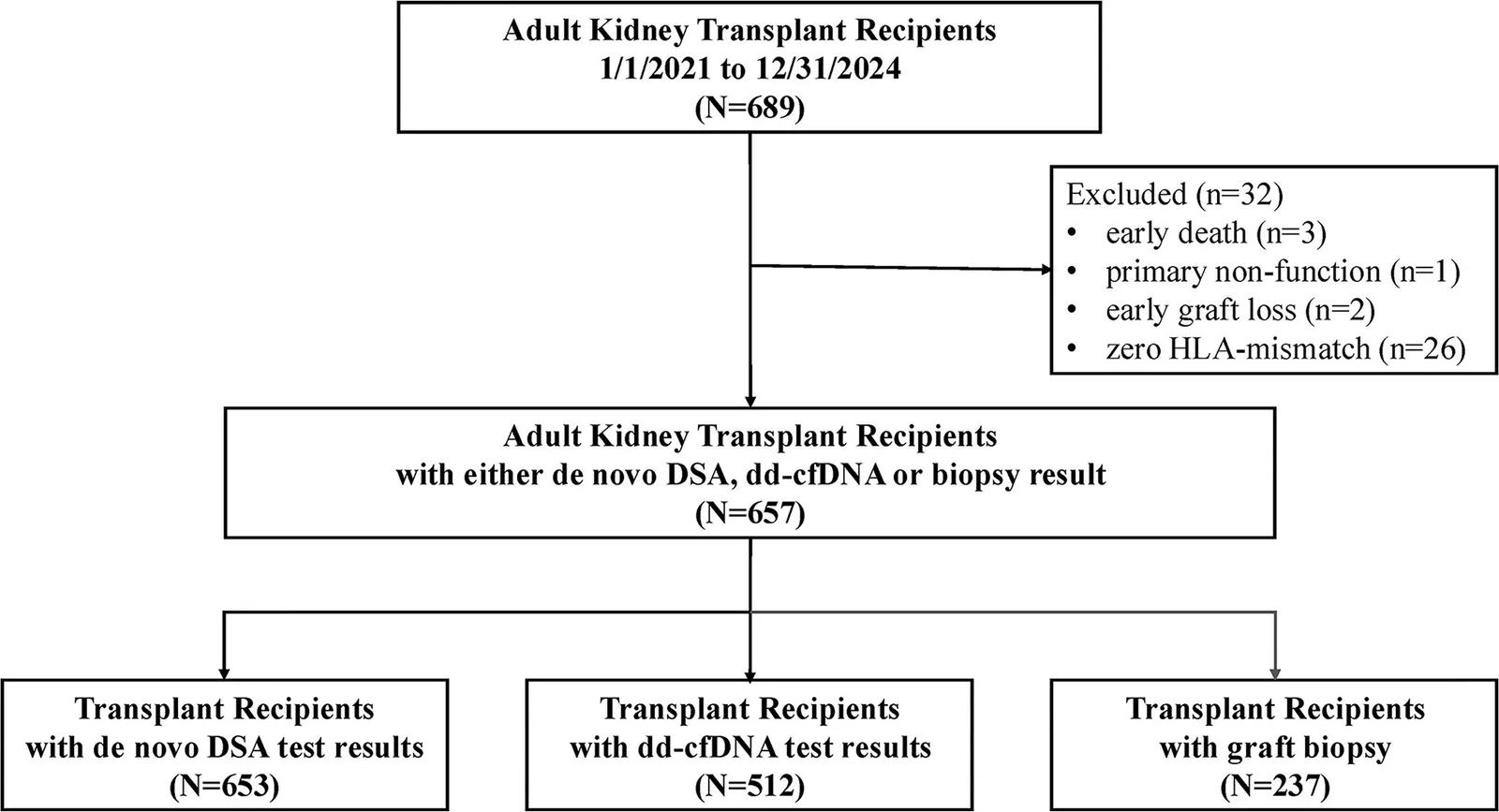
Flowchart of cohort selection.

**Table 1:** Patient characteristics.

	Total cohort
***N***	657
**Recipients’ characteristics**	
**Age (years), mean (SD)**	50.0 (15.0)
**Sex [*N* (%)]**	
Male	407 (61.9%)
Female	250 (38.1%)
**Race/ethnicity [*N* (%)]**	
White	437 (69.1%)
Hispanic	124 (19.6%)
African American	15 (2.4%)
Asian	26 (4.1%)
Other	29 (4.6%)
Unknown	1 (0.2%)
**Body Mass Index (kg/m^2^), mean (SD)**	28.7 (5.7)
**Dialysis vintage (months), median (IQR)**	33.8 (15.8–56.5)
**Cause of kidney failure [*N* (%)]**	
Diabetes mellitus	208 (31.7%)
Hypertension/vascular disease	85 (12.9%)
Cystic kidney disease	51 (7.8%)
Glomerulonephritis	153 (23.3%)
Other/unknown	160 (24.3%)
**Prior kidney transplant [*N* (%)]**	
No	596 (90.7%)
Yes	61 (9.3%)
**Multiorgan transplantation [*N* (%)]**	
Liver + kidney	15 (2.3%)
Pancreas + kidney	18 (2.7%)
Heart + kidney	11 (1.7%)
None	610 (92.8%)
Liver + Heart + Kidney	3 (0.5%)
**CMV risk categories [*N* (%)]**	
Low	166 (25.3%)
Intermediate	285 (43.4%)
High	186 (28.3%)
Unknown	20 (3.0%)
**Was the donor organ pumped? [*N* (%)]**	
No	175 (28.3%)
Yes	443 (71.7%)
**Transplantation characteristics**	
**Cold ischemic time (hours), median (IQR)**	13.4 (5.4–20.0)
**cPRA (%), median (IQR)**	0 (0–0)
**Donors’ characteristics**	
**Age (years), mean (SD)**	37.3 (15.7)
**Sex [*N* (%)]**	
Male	375 (57.1%)
Female	282 (42.9%)
**Race/ethnicity**	
White	532 (81.0%)
African American	21 (3.2%)
Asian	13 (2.0%)
Other	9 (1.4%)
Unknown	82 (12.5%)
**Donor type [*N* (%)]**	
Living	201 (30.6%)
Deceased	456 (69.4%)
**Donor DCD [*N* (%)]**	
No	261 (57.2%)
Yes	195 (42.8%)
**KDPI, median (IQR)**	27 (12–53)
**Donor cause of death [*N* (%)]**	
Anoxia	222 (48.7%)
Cerebrovascular/stroke	65 (14.3%)
Head trauma	153 (33.6%)
Central nervous system tumor	3 (0.7%)
Other	13 (2.9%)
**Donor terminal creatinine (mg/dl), median (IQR)**	0.82 (0.65–1.03)
**Donor diabetes [*N* (%)]**	
No	620 (95.4%)
Yes	30 (4.6%)
**Donor hypertension [*N* (%)]**	
No	550 (85.1%)
Yes	96 (14.9%)
**Donor malignancy [*N* (%)]**	
No	640 (97.4%)
Yes	17 (2.6%)
**Immunological characteristics**	
**Number of HLA mismatches (HLA A, B and DR) [*N* (%)]**	
1	17 (2.6%)
2	40 (6.1%)
3	122 (18.6%)
4	163 (24.8%)
5	212 (32.3%)
6	103 (15.7%)
**HLA mismatches A [*N* (%)]**	
0	19 (11.9%)
1	75 (46.9%)
2	66 (41.2%)
**HLA mismatches B [*N* (%)]**	
0	8 (5.0%)
1	46 (28.7%)
2	106 (66.2%)
**HLA mismatches DR [*N* (%)]**	
0	39 (11.8%)
1	169 (51.2%)
2	122 (37.0%)
**HLA mismatches DQ [*N* (%)]**	
0	30 (18.9%)
1	79 (49.7%)
2	50 (31.4%)
**Current flow B cell [*N* (%)]**	
<2 SD compatible	313 (91.8%)
2–3 SD compatible	15 (4.4%)
Weak positive	5 (1.5%)
Positive	8 (2.3%)
**Current flow T cell [*N* (%)]**	
<2 SD compatible	317 (93.0%)
2–3 SD compatible	12 (3.5%)
Weak positive	4 (1.2%)
Positive	8 (2.3%)
**Induction treatment [*N* (%)]**	
No Induction	0 (0.0%)
Basiliximab	75 (11.4%)
Thymoglobulin	552 (84.0%)
Steroid	656 (99.8%)
Alemtuzumab	19 (2.9%)
**Outcomes**	
**Proportion of Antibody Mediated Rejection of the subset of patients who underwent biopsy [*N* (%)]**	
No	176 (74.3%)
Yes	61 (25.7%)
**Proportion of rejection of the subset of patients who underwent biopsy [*N* (%)]**	
No	138 (58.2%)
Yes	99 (41.8%)
**Proportion of T-cell Mediated Rejection of the subset of patients who underwent biopsy [*N* (%)]**	
No	155 (65.4%)
Yes	82 (34.6%)
**Post-Transplant de novo Donor Specific Antibodies [*N* (%)]**	
No	522 (79.9%)
Yes	131 (20.1%)
**Dd-cf-DNA elevation [*N* (%)]**	
No	402 (78.5%)
Yes	110 (21.5%)
**Primary outcome [*N* (%)]**	
No	409 (62.3%)
Yes	248 (37.7%)
**Delayed Graft Function [*N* (%)]**	
No	601 (91.5%)
Yes	56 (8.5%)
**Death [*N* (%)]**	
No	628 (95.6%)
Yes	29 (4.4%)
**Graft Loss [*N* (%)]**	
No	644 (98.0%)
Yes	13 (2.0%)

Values are expressed as mean (standard deviation), median (interquartile range), or number (%).

### Primary outcome

Among the 657 patients included in the analysis, 248 (37.7%) experienced the primary outcome within the first year following transplantation. Baseline recipient, donor, and transplant characteristics stratified by risk category in all HLA loci are presented in Tables S1–S6. The median (IQR) eplet mismatch load across all HLA loci was significantly higher among patients who experienced the primary outcome than among those who did not (Figs 2 –7).

**Figure 2:**
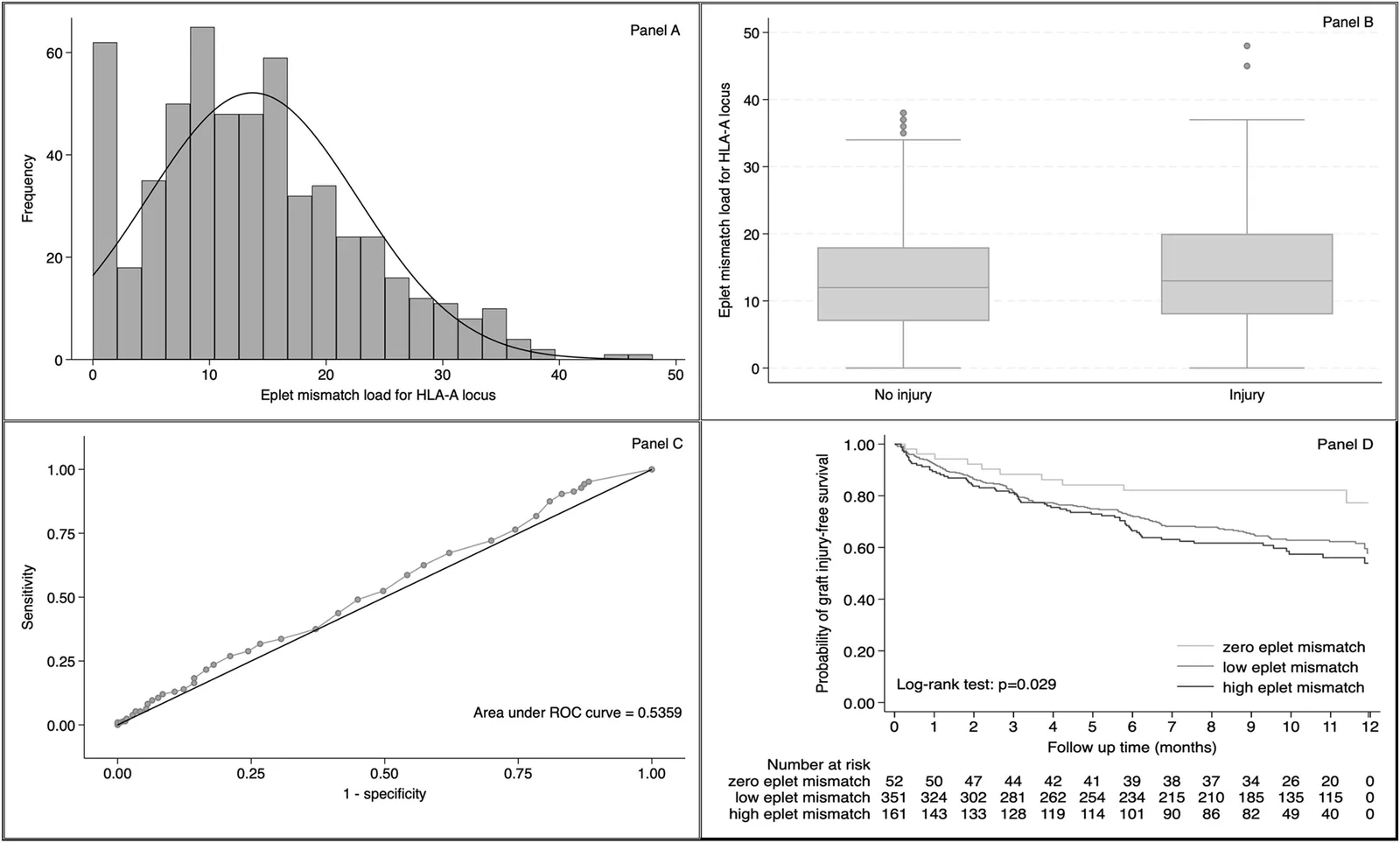
Result of HLA-A locus eplet mismatch load (panel A: Histogram of HLA-A locus eplet mismatch load; Panel B: Distribution of HLA-A locus eplet mismatch load by primary outcome; Panel C: Receiver Operating Characteristic curves of HLA-A locus eplet mismatch load for primary outcome; Panel D: Kaplan-Meier curves by HLA-A locus eplet mismatch load risk groups for primary outcome).

**Figure 3:**
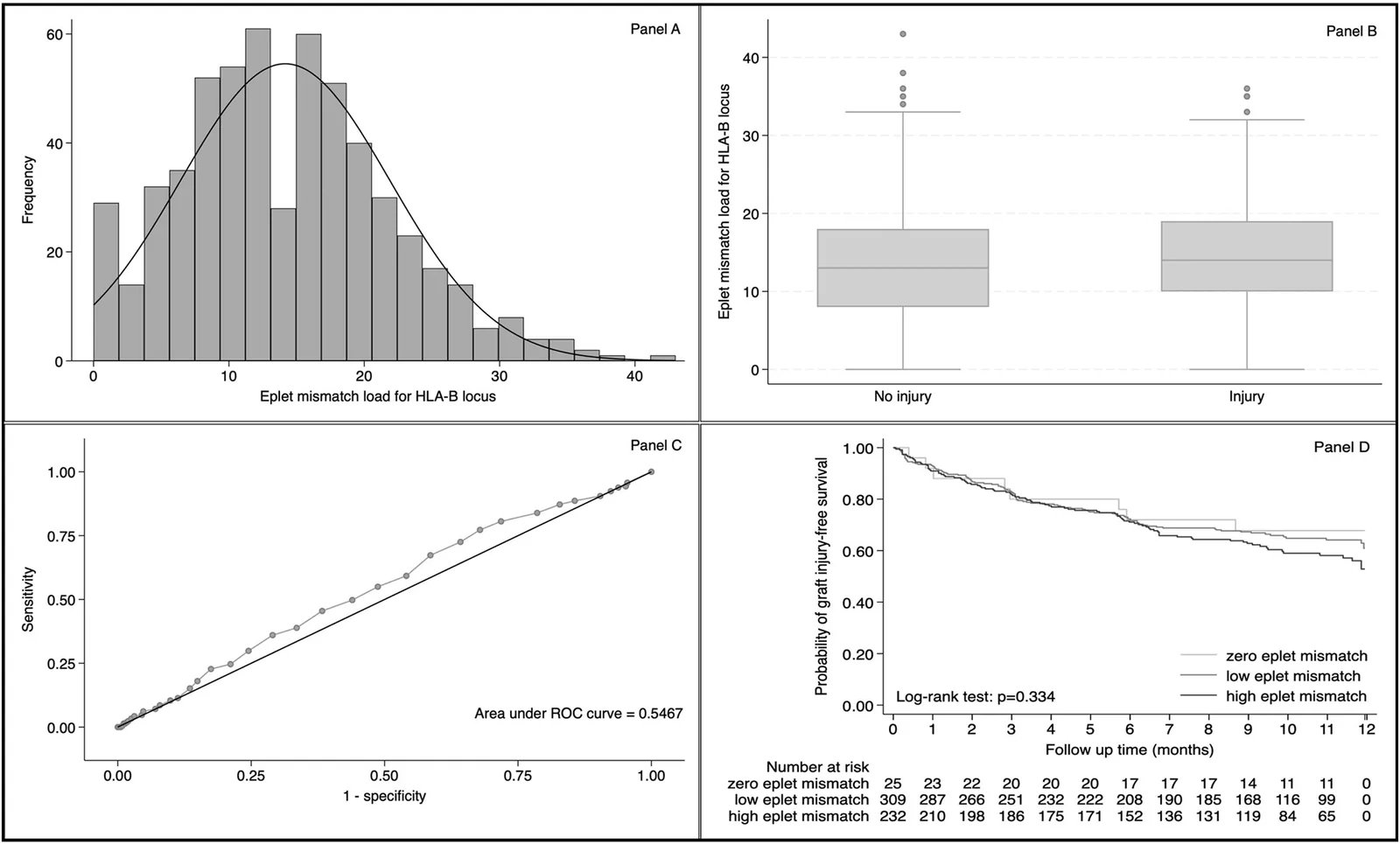
Result of HLA-B locus eplet mismatch load (panel A: Histogram of HLA-B locus eplet mismatch load; Panel B: Distribution of HLA-B locus eplet mismatch load by primary outcome; Panel C: Receiver Operating Characteristic curves of HLA-B locus eplet mismatch load for primary outcome; Panel D: Kaplan-Meier curves by HLA-B locus eplet mismatch load risk groups for primary outcome).

**Figure 4:**
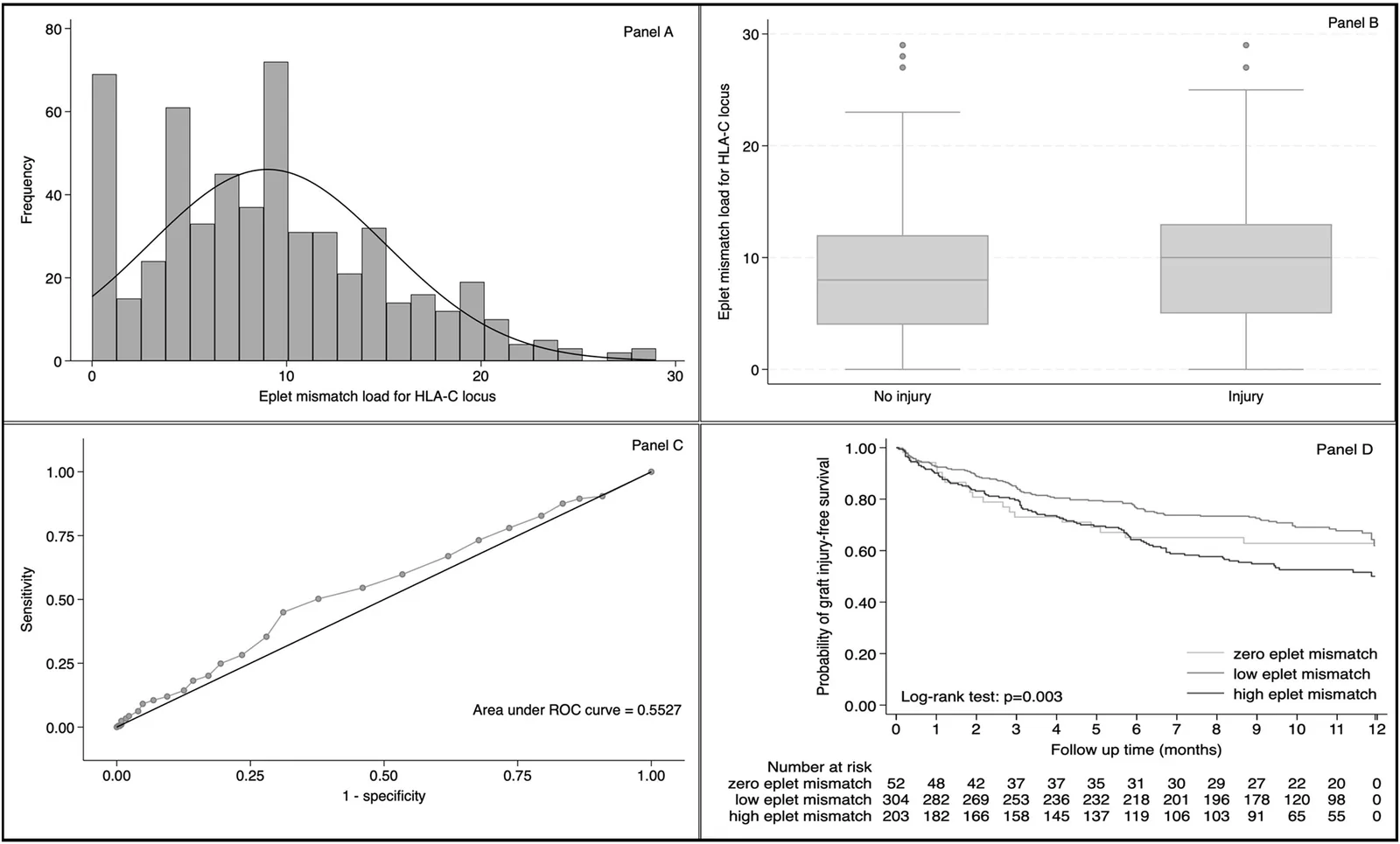
Result of HLA-C locus eplet mismatch load (panel A: Histogram of HLA-C locus eplet mismatch load; Panel B: Distribution of HLA-C locus eplet mismatch load by primary outcome; Panel C: Receiver Operating Characteristic curves of HLA-C locus eplet mismatch load for primary outcome; Panel D: Kaplan-Meier curves by HLA-C locus eplet mismatch load risk groups for primary outcome).

**Figure 5:**
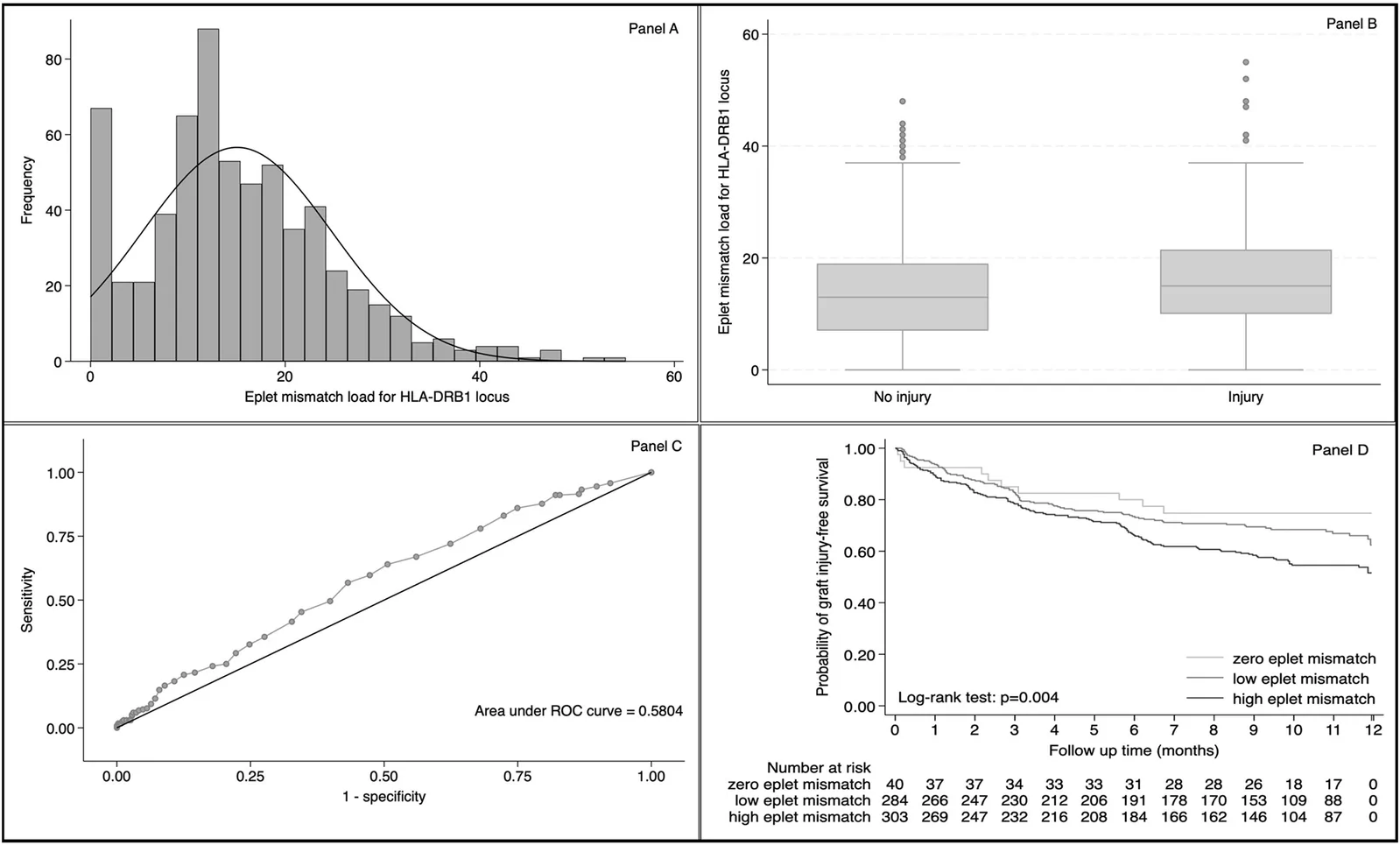
Result of HLA-DRB1 locus eplet mismatch load (panel A: Histogram of HLA-DRB1 locus eplet mismatch load; Panel B: Distribution of HLA-DRB1 locus eplet mismatch load by primary outcome; Panel C: Receiver Operating Characteristic curves of HLA-DRB1 locus eplet mismatch load for primary outcome; Panel D: Kaplan-Meier curves by HLA-DRB1 locus eplet mismatch load risk groups for primary outcome).

**Figure 6:**
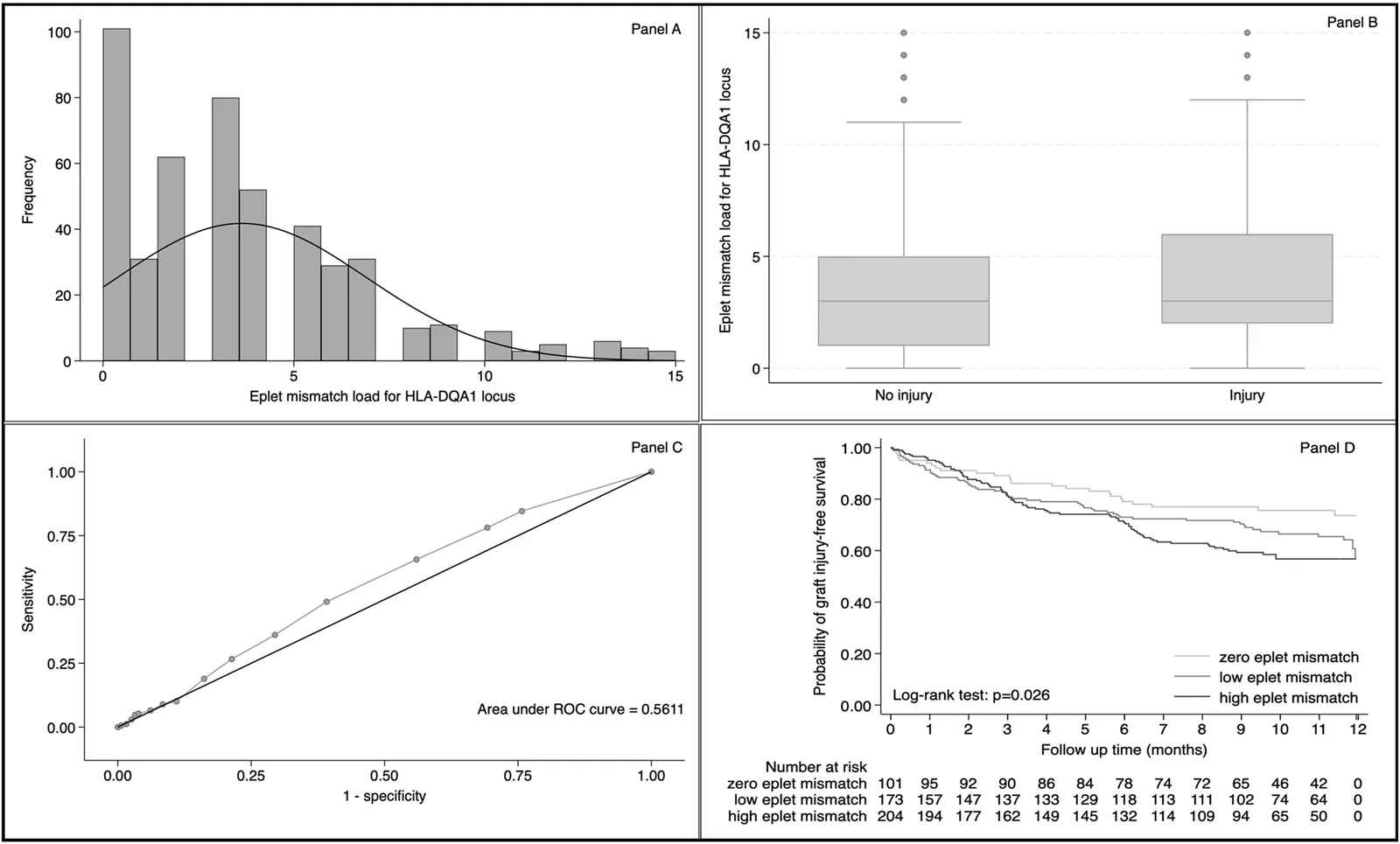
Result of HLA-DQA1 locus eplet mismatch load (panel A: Histogram of HLA-DQA1 locus eplet mismatch load; Panel B: Distribution of HLA-DQA1 locus eplet mismatch load by primary outcome; Panel C: Receiver Operating Characteristic curves of HLA-DQA1 locus eplet mismatch load for primary outcome; Panel D: Kaplan-Meier curves by HLA-DQA1 locus eplet mismatch load risk groups for primary outcome).

**Figure 7:**
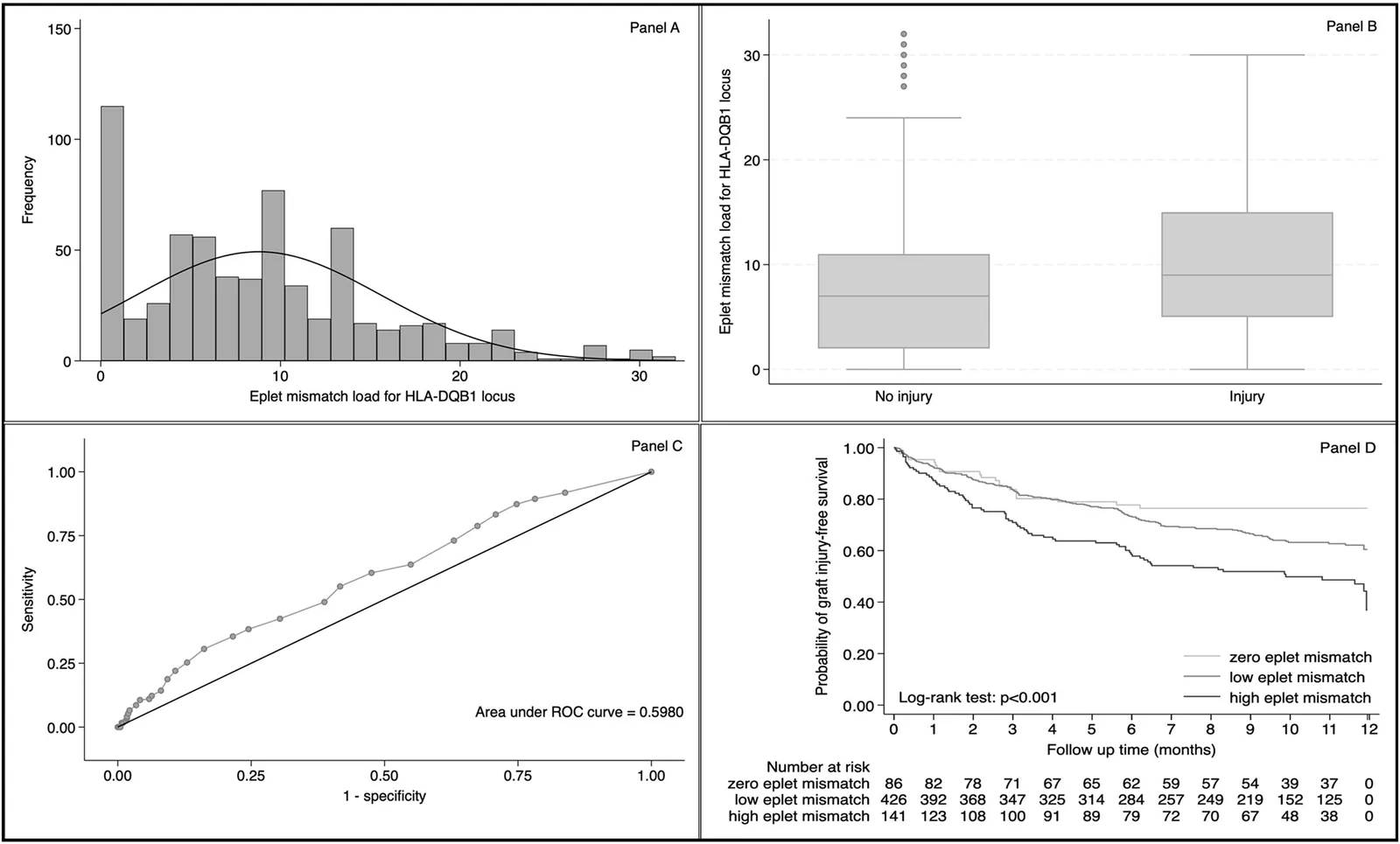
Result of HLA-DQB1 locus eplet mismatch load (panel A: Histogram of HLA-DQB1 locus eplet mismatch load; Panel B: Distribution of HLA-DQB1 locus eplet mismatch load by primary outcome; Panel C: Receiver Operating Characteristic curves of HLA-DQB1 locus eplet mismatch load for primary outcome; Panel D: Kaplan-Meier curves by HLA-DQB1 locus eplet mismatch load risk groups for primary outcome.

ROC analyses demonstrated that all six HLA loci exhibited weak discriminatory ability for the primary outcome (Figs 2–7). Based on the Youden Index, optimal cutoff values were identified to define low- and high-risk groups, in addition to the zero eplet mismatch load group: <18 versus ≥18 for the HLA-A locus, <16 versus ≥16 for HLA-B, <11 versus ≥11 for HLA-C, <15 versus ≥15 for HLA-DRB1, <4 versus ≥4 for HLA-DQA1, and <14 versus ≥14 for HLA-DQB1. Detailed diagnostic performance metrics are provided in Tables  S7–S12.

Higher eplet mismatch load at the HLA-C locus was significantly associated with an increased risk of the primary outcome as a continuous measure, both unadjusted (hazard ratio 1.023 per one-eplet increase, 95% confidence interval 1.001–1.046, *P* = .037) and after adjustment (hazard ratio 1.028, 95% confidence interval 1.004–1.052, *P* = .019) (Table 2). For HLA-B, the continuous per-eplet association reached significance only after adjustment (hazard ratio 1.021, 95% confidence interval 1.003–1.040, *P* = .024), while the unadjusted estimate did not (*P* = .091). For HLA-A, the continuous per-eplet measure was not significantly associated with the primary outcome in either unadjusted or adjusted models; however, compared with zero eplet mismatch, both low (adjusted hazard ratio 2.138, 95% confidence interval 1.076–4.248, *P* = .030) and high (adjusted hazard ratio 2.426, 95% confidence interval 1.184–4.971, *P* = .015) eplet mismatch categories were significantly associated with increased risk, a pattern not observed for HLA-B or HLA-C, where categorical low- and high-risk comparisons versus zero mismatch did not reach significance (Table 2; Figs 2–4).

**Table 2:** Association of class I HLA Eplet mismatch load and primary outcome using Cox proportional regression models.

Model		Hazard ratio (HR)	95% Confidence interval (CI) of HR	*P*-value		Hazard ratio (HR)	95% Confidence interval (CI) of HR	*P*-value		Hazard Ratio (HR)	95% Confidence interval (CI) of HR	*P*-value
**Unadjusted**	***N* = 564**				***N* = 566**				***N* = 559**			
	**HLA-A** (+1 eplet increase)	1.012	0.998–1.027	.102	**HLA-B** (+1 eplet increase)	1.014	0.998–1.033	.091	**HLA-C** (+1 eplet increase)	1.023	1.001–1.046	.037
	**HLA-A** (ref = Zero)				**HLA-B** (ref = Zero)				**HLA-C** (ref = Zero)			
	**Low**	2.108	1.108–4.010	.023	**Low**	1.022	0.517–2.018	.950	**Low**	0.765	0.472–1.240	.277
	**High**	2.410	1.239–4.688	.010	**High**	1.251	0.631–2.478	.521	**High**	1.252	0.773–2.030	.361
**Adjusted** ^a^	***N* = 542**				***N* = 544**				***N* = 540**			
	**HLA-A** (+1 eplet increase)	1.008	0.992–1.023	.344	**HLA-B** (+1 eplet increase)	1.021	1.003–1.040	.024	**HLA-C** (+1 eplet increase)	1.028	1.004–1.052	.019
	**HLA-A** (ref = Zero)				**HLA-B** (ref = Zero)				**HLA-C** (ref = Zero)			
	**Low**	2.138	1.076–4.248	.030	**Low**	1.334	0.653–2.726	.429	**Low**	0.904	0.541–1.511	.700
	**High**	2.426	1.184–4.971	.015	**High**	1.650	0.798–3.409	.177	**High**	1.552	0.915–2.632	.103

a Adjusted for: recipient age, recipient sex, recipient race/ethnicity, recipient BMI, dialysis vintage, cause of end stage renal disease, preemptive kidney transplantation, history of prior kidney transplantation, multiorgan transplantation, type of induction therapy, donor age, donor sex, donor race/ethnicity, donor type (living vs. deceased), cold ischemic time, cytomegalovirus risk status, cPRA at allocation

Stronger and more consistent associations were observed for class II HLA loci. Each one-eplet increase in mismatch load at HLA-DRB1, HLA-DQA1, and HLA-DQB1 was significantly associated with a higher risk of the primary outcome in both unadjusted and adjusted Cox models [adjusted hazard ratio 1.025 (95% confidence interval 1.011–1.038), 1.075 (1.025–1.127), and 1.038 (1.020–1.057), respectively; all *P* ≤ .003] (Table 3). Compared with zero eplet mismatch, high eplet mismatch was significantly associated with increased risk across all three class II loci in both unadjusted and adjusted models (adjusted hazard ratio 2.643, 2.240, and 3.128 for HLA-DRB1, HLA-DQA1, and HLA-DQB1, respectively; all *P* ≤ .004). The low-risk category reached significance versus zero mismatch for HLA-DQA1 (adjusted hazard ratio 1.679, *P* = .042) and HLA-DQB1 (adjusted hazard ratio 1.892, *P* = .011), but not for HLA-DRB1 (adjusted hazard ratio 1.690, *P* = .121) (Table 3; Figs 5–7).

**Table 3:** Association of class II HLA Eplet mismatch load and primary outcome using Cox proportional regression models.

Model		Hazard ratio (HR)	95% Confidence interval (CI) of HR	*P*-value		Hazard Ratio (HR)	95% Confidence interval (CI) of HR	*P*-value		Hazard ratio (HR)	95% Confidence INTERVAL (CI) of HR	*P*-value
**Unadjusted**	***N* = 627**				***N* = 478**				***N* = 653**			
	**HLA-DRB1** (+1 eplet increase)	1.021	1.008–1.034	.001	**HLA-DQA1** (+1 eplet increase)	1.050	1.005–1.098	.030	**HLA-DQB1** (+1 eplet increase)	1.038	1.021–1.056	<.001
	**HLA-DRB1** (ref = Zero)				**HLA-DQA1** (ref = Zero)				**HLA-DQB1** (ref = Zero)			
	**Low**	1.404	0.731–2.697	.308	**Low**	1.459	0.921–2.311	.108	**Low**	1.598	1.002–2.548	.049
	**High**	2.052	1.079–3.904	.028	**High**	1.811	1.165–2.816	.008	**High**	2.718	1.659–4.453	<.001
**Adjusted** ^a^	***N* = 602**				***N* = 461**				***N* = 628**			
	**HLA-DRB1** (+1 eplet increase)	1.025	1.011–1.038	<.001	**HLA-DQA1** (+1 eplet increase)	1.075	1.025–1.127	.003	**HLA-DQB1** (+1 eplet increase)	1.038	1.020–1.057	<.001
	**HLA-DRB1** (ref = Zero)				**HLA-DQA1** (ref = Zero)				**HLA-DQB1** (ref = Zero)			
	**Low**	1.690	0.870–3.283	.121	**Low**	1.679	1.020–2.765	.042	**Low**	1.892	1.158–3.092	.011
	**High**	2.643	1.357–5.145	.004	**High**	2.240	1.386–3.622	.001	**High**	3.128	1.859–5.264	<.001

a Adjusted for: recipient age, recipient sex, recipient race/ethnicity, recipient BMI, dialysis vintage, cause of end stage renal disease, preemptive kidney transplantation, history of prior kidney transplantation, multi-organ transplantation, type of induction therapy, donor age, donor sex, donor race/ethnicity, donor type (living vs. deceased), cold ischemic time, cytomegalovirus risk status, cPRA at allocation.

### Eplet mismatch versus conventional antigen-level HLA mismatch, and endpoint overlap

In a locus-by-locus comparator analysis, eplet mismatch load did not outperform conventional antigen-level HLA mismatch at any of the six loci tested; discrimination was statistically similar throughout (all bootstrap *P* > .05 for the difference in Harrell’s C-statistic), including at HLA-DRB1, the locus underlying our strongest eplet-based associations (Table 4, Fig. S1).

**Table 4:** Eplet mismatch vs. conventional antigen-level HLA mismatch: locus-by-locus comparator analysis.

Locus	Antigen variable (*r* with eplet)	*N* (model)	Antigen-only model HR (95% CI), *P*	Eplet-only model HR (95% CI), p	Harrell’s C antigen vs. eplet (Δ, boot p)	AUC (1-yr) antigen vs. eplet, *P*	Nested LR test p (eplet added to antigen)	Notes/caveats
**HLA-A**	A locus mismatch level (*r* = 0.73)	534	HR 2.12 (1.11–4.02) *P* = .022	HR 1.17 (0.84–1.62) *P* = .362	0.630 vs. 0.624 (Δ = –0.006, *P* = .47)	0.638 vs. 0.630 *P* = .42	*P* = .67	Adequately powered; antigen numerically favored
**HLA-B**	B locus mismatch level (*r* = 0.65)	536	HR 2.06 (0.98–4.35) *P* = .058	HR 1.62 (1.14–2.30) *P* = .007	0.632 vs. 0.630 (Δ = –0.002, *P* = .85)	0.641 vs. 0.643 *P* = .81	*P* = .17	Adequately powered; discrimination essentially equivalent
**HLA-C**	C locus mismatch level (*r* = 0.68)	82 (reduced covariates)	HR 1.12 (0.28–4.39) *P* = .873	HR 0.59 (0.25–1.38) *P* = .223	0.708 vs. 0.724 (Δ = +0.016, *P* = .61)	0.699 vs. 0.724 *P* = .54	*P* = .06	85% missing antigen-level data; underpowered (*N* = 82); interpret cautiously
**HLA-DRB1**	DR locus mismatch level (*r* = 0.74)	593	HR 3.04 (1.78–5.21) *P* < .001	HR 1.64 (1.24–2.16) *P* < .001	0.646 vs. 0.631 (Δ = –0.017, *P* = .29)	0.658 vs. 0.642 *P* = .20	*P* = .46	Best-powered locus; largest (non-significant) gap favoring antigen
**HLA-DQA1**	DQ alpha locus mismatch level (*r* = 0.38)	130 (reduced covariates)	HR 0.60 (0.24–1.54) *P* = .292	HR 1.00 (0.55–1.85) *P* = .990	0.651 vs. 0.611 (Δ = –0.040, *P* = .23)	0.621 vs. 0.570 *P* = .13	*P* = .78	Weak antigen–eplet correlation; DQA1 not routinely typed at the antigen level; comparison not considered reliable
**HLA-DQB1**	DQ beta locus mismatch level (*r* = 0.74)	158 (reduced covariates)	HR 1.36 (0.58–3.18) *P* = .481	HR 0.80 (0.42–1.85) *P* = .605	0.648 vs. 0.638 (Δ = –0.010, *P* = .67)	0.622 vs. 0.595 *P* = .27	*P* = .60	76% missing antigen-level data; underpowered (*N* = 158)

Abbreviations: HR, hazard ratio; CI, confidence interval; AUC, area under the receiver operating characteristic curve; LR, likelihood ratio; cPRA, calculated panel-reactive antibody. Eplet mismatch dichotomized at the locus-specific Youden-index-optimal cutoff (HLA-A ≥ 20; HLA-B ≥ 10; HLA-C ≥ 11; HLA-DRB1 ≥ 15; HLA-DQA1 ≥ 4; HLA-DQB1 ≥ 14). Antigen-level HR shown for the highest mismatch category vs. zero mismatch.

Among the 657 patients in the analytic cohort, 248 (37.7%) met the primary composite endpoint; of these, 106 had complete data on all three component markers (de-novo DSA, rejection, dd-cfDNA elevation), of whom 62 (58.5%) met two or more markers concurrently (‘concordant’) and 44 (41.5%) met exactly one (‘discordant’). Eplet mismatch load did not differ significantly between concordant and discordant patients at any of the six HLA loci, either unadjusted or after covariate adjustment (all *P* > .05) (Table S13, Fig. S2).

### Secondary outcomes

#### Donor specific antibodies

Among the 653 patients with de novo DSA data included in the analysis, 131 (20.1%) developed post-transplant de-novo DSA within the first year following transplantation. Baseline recipient, donor, and transplant characteristics, stratified by risk category across all HLA loci, are presented in Tables S14–S19. The median (IQR) eplet mismatch load for all HLA loci and ROC analyses are shown in Figs S3–S8.

ROC analyses demonstrated that all six HLA loci exhibited weak discriminative ability for post-transplant de novo DSA (Figs S3–S8). Detailed diagnostic performance metrics are provided in Tables S20–S25.

For class I HLA loci, only the low- and high-risk HLA-A categories, compared to the zero eplet mismatch load category, were significantly associated with an increased risk of post-transplant de-novo DSA, as shown by Cox regression analyses (Table S26) and Kaplan–Meier survival curves (Figs S3–S5). In contrast, eplet mismatch load at class II HLA loci was significantly associated with an increased risk of post-transplant de-novo DSA, as demonstrated by Cox regression analyses (Table S27) and Kaplan–Meier survival curves (Figs S6–S8).

#### Rejection based on histopathological evaluation and/or molecular microscope diagnostic system

Among the 657 patients included in the analysis, of whom 237 underwent kidney biopsy, 99 patients (15%) experienced rejection within the first year after transplantation, including 61 (9%) cases of ABMR and 82 (13%) cases of TCMR. Baseline recipient, donor, and transplant characteristics stratified by risk category in all HLA loci are presented in Table S28–S33. The median (IQR) eplet mismatch load for all HLA loci and ROC analyses are shown in Figs S9–S14.

ROC analyses demonstrated that all six HLA loci exhibited weak discriminatory ability for rejection (Figs S9–S14). Detailed diagnostic performance metrics are provided in Tables S34–S39.

None of the class I HLA loci eplet mismatch loads were associated with rejection (Table S40 and Figs S9–S11). Among class II loci, higher eplet mismatch load at HLA-DRB1 (continuous measure, adjusted hazard ratio 1.035, *P* = .011) and HLA-DQB1 (categorical high-risk, adjusted hazard ratio 4.368, *P* = .006; low-risk hazard ratio 3.081, *P* = .020) was significantly associated with an increased risk of rejection; HLA-DQA1 was not significantly associated with rejection in this cohort, as demonstrated by Cox regression analyses (Table S41) and Kaplan–Meier survival curves (Figs S12–S14).

#### Elevation of donor-derived cell-free DNA

Among the 512 patients with dd-cfDNA data included in the analysis, 110 (21.5%) developed elevated dd-cfDNA within the first year following transplantation. Baseline recipient, donor, and transplant characteristics stratified by risk category in all HLA loci are presented in Tables S42–S47. The median (IQR) eplet mismatch load for all HLA loci and ROC analyses are shown in Figs S15–S20.

ROC analyses demonstrated that all six HLA locus exhibited weak discriminatory ability for developing elevated dd-cfDNA within the first year following transplantation (Figs S15–S20). Detailed diagnostic performance metrics are provided in Tables S48–S53.

In unadjusted models, high-risk HLA-A eplet mismatch was significantly associated with elevated dd-cfDNA (hazard ratio 3.293, *P* = .022); this association was attenuated after adjustment (hazard ratio 3.211, *P* = .052). No significant associations were observed for HLA-B or HLA-C (Table S54). In contrast, eplet mismatch load at class II HLA loci, except HLA-DQB1, was significantly associated with an increased risk of elevated dd-cfDNA, most consistently in the high-risk category (Table S55) and in Kaplan–Meier survival curves (Figs S15–S17).

## DISCUSSION

In this single-center retrospective study of over 650 kidney transplant recipients, we demonstrate that higher class II eplet mismatch load is independently associated with early allograft injury during the first year following transplantation. By integrating multiple complementary and clinically validated biomarkers—including donor-specific antibodies, donor-derived cell-free DNA, histologic rejection, and molecular rejection assessed by the Molecular Microscope® Diagnostic System—we provide a comprehensive and clinically relevant assessment of early alloimmune injury. In addition, patients with high HLA-A eplet mismatch load experienced a two- to three-fold increased risk of the composite primary outcome compared with those with zero class I mismatch, supporting the relevance of molecular mismatch assessment beyond conventional HLA antigen matching.

Our findings confirm and extend prior studies demonstrating a robust association between higher class II eplet mismatch load and post-transplant de-novo DSA development and rejection risk [2–7, 9–17, 23, 24]. The consistency of these results across cohorts reinforces the validity of class II eplet mismatch as a marker of humoral alloimmune risk. In contrast, evidence linking eplet mismatch load to donor-derived cell-free DNA has been extremely limited. To date, only one small kidney transplant study evaluated this relationship, enrolling 42 patients and assessing cell-free DNA at a single time point [2]. In this context, our study represents the first large-scale analysis demonstrating an independent association between class II eplet mismatch load and subsequent elevation of donor-derived cell-free DNA, a sensitive marker of active graft injury. Notably, compared with zero eplet mismatch, patients with high-risk HLA-DRB1 or HLA-DQA1 eplet mismatch had a two- to six-fold higher adjusted risk of cell-free DNA elevation; HLA-DQB1 was not significantly associated with this outcome, underscoring that the class II-dd-cfDNA association is locus-dependent rather than uniform across class II loci.

Our results also provide novel insight into the role of class I eplet mismatch. Across the kidney transplant literature, class I eplet mismatch has shown weaker and less consistent associations with donor-specific antibodies and rejection than class II mismatch, particularly at HLA-DQ. In several well-powered cohorts, class I effects diminished or disappeared after adjustment for class II eplets, leading most studies to focus primarily on DR and DQ loci [2–4, 6, 7]. Nevertheless, smaller or earlier studies reported associations between higher class I eplet load—especially at HLA-B—and sensitization or anti–HLA class I antibodies [19]. These discrepancies likely reflect differences in HLA typing resolution, endpoints examined, statistical modeling, and the dominant immunologic influence of class II mismatch.

In contrast to prior reports, we observed independent associations between class I eplet mismatch load and early allograft injury, although the pattern differed by locus. Compared with zero eplet mismatch, high eplet load at the HLA-A locus was associated with a two- to three-fold increase in the risk of early injury (adjusted hazard ratio, 2.43), evident as a categorical risk-group effect rather than a continuous per-eplet effect. HLA-B and HLA-C, in contrast, showed significant continuous per-eplet associations after adjustment but no significant categorical risk-group differences, suggesting more modest, dose-dependent effects at these loci. Consistent with the absence of a class I association with rejection specifically (see Secondary outcomes), these class I associations with the composite outcome appear to be driven predominantly by DSA development and dd-cfDNA elevation rather than by rejection. A likely explanation for these findings is our comprehensive outcome of outcomes, which captures multiple manifestations of alloimmune injury rather than relying on a single biomarker or endpoint.

Contrary to our initial hypothesis, eplet-mismatch load did not demonstrate superior discrimination for the primary composite outcome compared with conventional antigen-level HLA mismatch at any of the six loci examined. Head-to-head comparisons showed only small, non-significant differences in Harrell’s C-statistic between antigen-only and eplet-only models (Δ ranging from −0.040 to +0.016 across loci; all bootstrap *P* ≥.13), and AUC comparisons were similarly non-significant (all likelihood-ratio *P* ≥ .06). At HLA-A, HLA-DRB1, HLA-DQA1, and HLA-DQB1, antigen-level mismatch was even numerically favored over eplet mismatch, though none of these differences reached statistical significance. This finding likely reflects the strong correlation between eplet and antigen mismatch scores at most loci (*r* = 0.65–0.74, except HLA-DQA1 at *r* = 0.38), since eplets are structurally nested within antigen-level HLA differences and therefore largely recapitulate the same alloimmunogenic signal already captured by conventional matching. Comparisons at HLA-C, HLA-DQA1, and HLA-DQB1 were additionally underpowered due to substantial missingness in antigen-level typing data (76%–85%) and should be interpreted with caution. Taken together, these results suggest that any clinical value of eplet mismatch may lie less in superior outcome discrimination than in providing a more mechanistically granular, quantitative framework for risk stratification, and future studies with complete antigen-level typing and larger, multicenter cohorts are needed to clarify whether eplet-based matching offers meaningful incremental benefit over standard antigen matching.

Our results have important implications for clinical practice. We used the HLA Eplet Registry, a free and publicly accessible web-based tool (https://www.epregistry.com.br/) that enables calculation of class II eplet mismatch load and I using high-resolution donor and recipient HLA typing. We also identified pragmatic, locus-specific cutoff values that categorize transplant pairs into zero-, low-, and high-eplet-mismatch risk groups. While class II eplet mismatch is increasingly incorporated into living donor exchange programs (both the Alliance for Paired Kidney Donation and the National Kidney Registry), class I eplet mismatch and its application in deceased donor transplantation remain uncommon. Our findings suggest that broader implementation of eplet-based risk stratification may meaningfully refine immunologic risk assessment and inform decisions regarding induction therapy, maintenance immunosuppression, and post-transplant monitoring.

Another strength of this study is the use of a composite outcome that integrates serologic, histologic, molecular, and cell-free DNA–based markers of injury. This approach reflects real-world clinical practice, where graft injury is often multifactorial and incompletely captured by any single test. Our findings indicate that eplet mismatch load—particularly at class II loci—is associated with a global susceptibility to alloimmune injury rather than a single downstream manifestation.

Several limitations warrant consideration. The retrospective single-center design limits causal inference and generalizability, and external validation in diverse cohorts is required. Importantly, the eplet-mismatch risk thresholds and model estimates reported here were derived and evaluated entirely within this single retrospective cohort; no external or independent validation cohort was available to confirm their reproducibility, and these thresholds should therefore be regarded as hypothesis-generating until confirmed in independent, ideally multicenter and multiethnic, kidney transplant populations. Although most HLA typing was performed at high resolution, a minority of cases (<15%) required exclusion due to insufficient typing resolution. Residual confounding also cannot be fully excluded despite comprehensive multivariable adjustment. The composite primary endpoint combines protocol-driven surveillance measures (de novo DSA, assessed at fixed intervals, and dd-cfDNA) with clinically indicated histologic and molecular rejection, which were ascertained only in the subset of patients who underwent a clinically indicated biopsy; this differential ascertainment introduces the potential for surveillance/detection bias and may weight the composite outcome toward the two protocol-driven biomarkers. We were also unable to perform locus-specific analyses linking eplet mismatch load at a given HLA locus to de novo DSA directed against that same locus, as granular per-locus de novo DSA data were not available. Access to the HLA Eplet Registry and the Molecular Microscope Diagnostic System, while increasingly available, remains limited at many transplant centers, which may constrain the immediate generalizability of eplet-based risk stratification. Finally, we did not collect data on maintenance immunosuppression or corticosteroid use, which may independently influence graft injury during the first post-transplant year; as in most retrospective studies in this field, reliably capturing this information was not feasible.

Despite these limitations, the strengths of this study—including its large contemporary cohort, integrated biomarker assessment, and robust modeling—support its translational relevance. Eplet mismatch load offers a mechanistically grounded, readily implementable framework for immunologic risk stratification at no additional cost or patient burden; however, given the modest discriminatory performance observed across all HLA loci, prospective external validation is needed before any locus-specific cutoff can be considered clinically actionable.

In conclusion, higher eplet mismatch load, particularly at class II loci, is independently associated with early alloimmune injury after kidney transplantation across multiple complementary biomarkers. While discriminatory performance was modest, eplet-based matching provides valuable baseline risk stratification and may guide individualized donor selection, immunosuppressive strategies, and post-transplant surveillance. Prospective studies are needed to determine whether eplet-guided clinical decision-making improves long-term transplant outcomes.

## Supplementary Material

sfag341_Supplemental_File

## Data Availability

The original contributions presented in the study are included in the article/supplementary material. Further inquiries can be directed to the corresponding author.
