# Supplementary material for: Association of eplet mismatch load and allograft injury in kidney transplant recipients
Source: Clin Kidney J. 2026 Sep 26;19(10):sfag341. doi: 10.1093/ckj/sfag341 (PMC13655737; doi:10.1093/ckj/sfag341)
Supplement: Supplementary file 1 [file sfag341_supplemental_file.pdf]

Association of Eplet Mismatch Load and Allograft Injury in Kidney Transplant Recipients

Supplemental Material:

Legend of Supplemental Figures:

Figure S1: Receiver Operating Characteristic curves of HLA vs. Eplet mismatch risk for primary outcome (Panel A: A locus; Panel B: B locus; Panel C: C locus; Panel D: DRB1 locus; Panel E: DQA1 locus; Panel F: DQB1 locus)

Figure S2. UpSet plot of DSA, rejection, and dd-cfDNA elevation co-occurrence among endpoint-positive patients with complete data on all three markers (n=106)

Figure S3: Result of HLA-A locus eplet mismatch load (panel A: Histogram of HLA-A locus eplet mismatch load; Panel B: Distribution of HLA-A locus eplet mismatch load by de novo donor specific antibody outcome; Panel C: Receiver Operating Characteristic curves of HLA-A locus eplet mismatch load for de novo donor specific antibody outcome; Panel D: Kaplan-Meier curves by HLA-A locus eplet mismatch load risk groups for de novo donor specific antibody outcome)

Figure S4: Result of HLA-B locus eplet mismatch load (panel A: Histogram of HLA-B locus eplet mismatch load; Panel B: Distribution of HLA-B locus eplet mismatch load by de novo donor specific antibody outcome; Panel C: Receiver Operating Characteristic curves of HLA-B locus eplet mismatch load for de novo donor specific antibody outcome; Panel D: Kaplan-Meier curves by HLA-B locus eplet mismatch load risk groups for de novo donor specific antibody outcome)

Figure S5: Result of HLA-C locus eplet mismatch load (panel A: Histogram of HLA-C locus eplet mismatch load; Panel B: Distribution of HLA-C locus eplet mismatch load by de novo donor specific antibody outcome; Panel C: Receiver Operating Characteristic curves of HLA-C locus eplet mismatch load for de novo donor specific antibody outcome; Panel D: Kaplan-Meier curves by HLA-C locus eplet mismatch load risk groups for de novo donor specific antibody outcome)

Figure S6: Result of HLA-DRB1 locus eplet mismatch load (panel A: Histogram of HLA-DRB1 locus eplet mismatch load; Panel B: Distribution of HLA-DRB1 locus eplet mismatch load by de novo donor specific antibody outcome; Panel C: Receiver Operating Characteristic curves of HLA-DRB1 locus eplet mismatch load for de novo donor specific antibody outcome; Panel D: Kaplan-Meier curves by HLA-DRB1 locus eplet mismatch load risk groups for de novo donor specific antibody outcome)

Figure S7: Result of HLA-DQA1 locus eplet mismatch load (panel A: Histogram of HLA-DQA1 locus eplet mismatch load; Panel B: Distribution of HLA-DQA1 locus eplet mismatch load by de novo donor specific antibody outcome; Panel C: Receiver Operating Characteristic curves of HLA-DQA1 locus eplet mismatch load for de novo donor specific antibody outcome; Panel D: Kaplan-Meier curves by HLA-DQA1 locus eplet mismatch load risk groups for de novo donor specific antibody outcome)

Figure S8: Result of HLA-DQB1 locus eplet mismatch load (panel A: Histogram of HLA-DQB1 locus eplet mismatch load; Panel B: Distribution of HLA-DQB1 locus eplet mismatch load by de novo donor specific antibody outcome; Panel C: Receiver Operating Characteristic curves of HLA-DQB1 locus eplet mismatch load for de novo donor specific antibody outcome;

Panel D: Kaplan-Meier curves by HLA-DQB1 locus eplet mismatch load risk groups for de novo donor specific antibody outcome)

Figure S9: Result of HLA-A locus eplet mismatch load (panel A: Histogram of HLA-A locus eplet mismatch load; Panel B: Distribution of HLA-A locus eplet mismatch load by rejection outcome; Panel C: Receiver Operating Characteristic curves of HLA-A locus eplet mismatch load for rejection outcome; Panel D: Kaplan-Meier curves by HLA-A locus eplet mismatch load risk groups for rejection outcome)

Figure S10: Result of HLA-B locus eplet mismatch load (panel A: Histogram of HLA-B locus eplet mismatch load; Panel B: Distribution of HLA-B locus eplet mismatch load by rejection outcome; Panel C: Receiver Operating Characteristic curves of HLA-B locus eplet mismatch load for rejection outcome; Panel D: Kaplan-Meier curves by HLA-B locus eplet mismatch load risk groups for rejection outcome)

Figure S11: Result of HLA-C locus eplet mismatch load (panel A: Histogram of HLA-C locus eplet mismatch load; Panel B: Distribution of HLA-C locus eplet mismatch load by rejection outcome; Panel C: Receiver Operating Characteristic curves of HLA-C locus eplet mismatch load for rejection outcome; Panel D: Kaplan-Meier curves by HLA-C locus eplet mismatch load risk groups for rejection outcome)

Figure S12: Result of HLA-DRB1 locus eplet mismatch load (panel A: Histogram of HLA-DRB1 locus eplet mismatch load; Panel B: Distribution of HLA-DRB1 locus eplet mismatch load by rejection outcome; Panel C: Receiver Operating Characteristic curves of HLA-DRB1 locus eplet mismatch load for rejection outcome; Panel D: Kaplan-Meier curves by HLA-DRB1 locus eplet mismatch load risk groups for rejection outcome)

Figure S13: Result of HLA-DQA1 locus eplet mismatch load (panel A: Histogram of HLA-DQA1 locus eplet mismatch load; Panel B: Distribution of HLA-DQA1 locus eplet mismatch load by rejection outcome; Panel C: Receiver Operating Characteristic curves of HLA-DQA1 locus eplet mismatch load for rejection outcome; Panel D: Kaplan-Meier curves by HLA-DQA1 locus eplet mismatch load risk groups for rejection outcome)

Figure S14: Result of HLA-DQB1 locus eplet mismatch load (panel A: Histogram of HLA-DQB1 locus eplet mismatch load; Panel B: Distribution of HLA-DQB1 locus eplet mismatch load by rejection outcome; Panel C: Receiver Operating Characteristic curves of HLA-DQB1 locus eplet mismatch load for rejection outcome; Panel D: Kaplan-Meier curves by HLA-DQB1 locus eplet mismatch load risk groups for rejection outcome)

Figure S15: Result of HLA-A locus eplet mismatch load (panel A: Histogram of HLA-A locus eplet mismatch load; Panel B: Distribution of HLA-A locus eplet mismatch load by elevated donor-derived cell free DNA outcome; Panel C: Receiver Operating Characteristic curves of HLA-A locus eplet mismatch load for elevated donor-derived cell free DNA outcome; Panel D: Kaplan-Meier curves by HLA-A locus eplet mismatch load risk groups for elevated donor-derived cell free DNA outcome)

Figure S16: Result of HLA-B locus eplet mismatch load (panel A: Histogram of HLA-B locus eplet mismatch load; Panel B: Distribution of HLA-B locus eplet mismatch load by elevated donor-derived cell free DNA outcome; Panel C: Receiver Operating Characteristic curves of HLA-B locus eplet mismatch load for elevated donor-derived cell free DNA outcome; Panel D: Kaplan-Meier curves by HLA-B locus eplet mismatch load risk groups for elevated donor-derived cell free DNA outcome)

Figure S17: Result of HLA-C locus eplet mismatch load (panel A: Histogram of HLA-C locus eplet mismatch load; Panel B: Distribution of HLA-C locus eplet mismatch load by elevated donor-derived cell free DNA outcome; Panel C: Receiver Operating Characteristic curves of HLA-C locus eplet mismatch load for elevated donor-derived cell free DNA outcome; Panel D: Kaplan-Meier curves by HLA-C locus eplet mismatch load risk groups for elevated donor-derived cell free DNA outcome)

Figure S18: Result of HLA-DRB1 locus eplet mismatch load (panel A: Histogram of HLA-DRB1 locus eplet mismatch load; Panel B: Distribution of HLA-DRB1 locus eplet mismatch load by elevated donor-derived cell free DNA outcome; Panel C: Receiver Operating Characteristic curves of HLA-DRB1 locus eplet mismatch load for elevated donor-derived cell free DNA outcome; Panel D: Kaplan-Meier curves by HLA-DRB1 locus eplet mismatch load risk groups for elevated donor-derived cell free DNA outcome)

Figure S19: Result of HLA-DQA1 locus eplet mismatch load (panel A: Histogram of HLA-DQA1 locus eplet mismatch load; Panel B: Distribution of HLA-DQA1 locus eplet mismatch load by elevated donor-derived cell free DNA outcome; Panel C: Receiver Operating Characteristic curves of HLA-DQA1 locus eplet mismatch load for elevated donor-derived cell free DNA n outcome; Panel D: Kaplan-Meier curves by HLA-DQA1 locus eplet mismatch load risk groups for elevated donor-derived cell free DNA outcome)

Figure S20: Result of HLA-DQB1 locus eplet mismatch load (panel A: Histogram of HLA-DQB1 locus eplet mismatch load; Panel B: Distribution of HLA-DQB1 locus eplet mismatch load by elevated donor-derived cell free DNA outcome; Panel C: Receiver Operating Characteristic curves of HLA-DQB1 locus eplet mismatch load for elevated donor-derived cell

free DNA outcome; Panel D: Kaplan-Meier curves by HLA-DQB1 locus eplet mismatch load risk groups for elevated donor-derived cell free DNA outcome)

Legend of Supplemental Tables:

Table S1: Patient characteristics based on eplet mismatch load for HLA-A locus for primary outcome

Table S2: Patient characteristics based on eplet mismatch load for HLA-B locus for primary outcome

Table S3: Patient characteristics based on eplet mismatch load for HLA-C locus for primary outcome

Table S4: Patient characteristics based on eplet mismatch load for HLA-DQA1 locus for primary outcome

Table S5: Patient characteristics based on eplet mismatch load for HLA-DQB1 locus for primary outcome

Table S6: Patient characteristics based on eplet mismatch load for HLA-DRB1 locus for primary outcome

Table S7: Performance characteristics table of eplet mismatch load for HLA-A locus for primary outcome

Table S8: Performance characteristics table of eplet mismatch load for HLA-B locus for primary outcome

Table S9: Performance characteristics table of eplet mismatch load for HLA-C locus for primary outcome

Table S10: Performance characteristics table of eplet mismatch load for HLA-DQA1 locus for primary outcome

Table S11: Performance characteristics table of eplet mismatch load for HLA-DQB1 locus for primary outcome

Table S12: Performance characteristics table of eplet mismatch load for HLA-DRB1 locus for primary outcome

Table S13. Eplet mismatch load, concordant vs. discordant patients, by locus

Table S14: Patient characteristics of the de novo DSA cohort based on eplet mismatch load for HLA-A locus

Table S15: Patient characteristics of the de novo DSA cohort based on eplet mismatch load for HLA-B locus

Table S16: Patient characteristics for the de novo DSA cohort based on eplet mismatch load for HLA-C locus

Table S17: Patient characteristics for the de novo DSA cohort based on eplet mismatch load for HLA-DQA1 locus

Table S18: Patient characteristics for the de novo DSA cohort based on eplet mismatch load for HLA-DQB1 locus

Table S19: Patient characteristics for the de novo DSA cohort based on eplet mismatch load for HLA-DRB1 locus

Table S20: Performance characteristics table of eplet mismatch load for HLA-A locus for de novo DSA

Table S21: Performance characteristics table of eplet mismatch load for HLA-B locus for de novo DSA

Table S22: Performance characteristics table of eplet mismatch load for HLA-C locus for de novo DSA

Table S23: Performance characteristics table of eplet mismatch load for HLA-DQA1 locus for de novo DSA

Table S24: Performance characteristics table of eplet mismatch load for HLA-DQB1 locus for de novo DSA

Table S25: Performance characteristics table of eplet mismatch load for HLA-DRB1 locus for de novo DSA

Table S26: Association of class I HLA Eplet mismatch load and de novo DSA using Cox proportional regression models

Table S27: Association of class II HLA Eplet mismatch load and de novo DSA using Cox proportional regression models

Table S28: Patient characteristics for the rejection cohort based on eplet mismatch load for HLA-A locus

Table S29: Patient characteristics for the rejection cohort based on eplet mismatch load for HLA-B locus

Table S30: Patient characteristics for the rejection cohort based on eplet mismatch load for HLA-C locus

Table S31: Patient characteristics for the rejection cohort based on eplet mismatch load for HLA-DQA1 locus

Table S32: Patient characteristics for the rejection cohort based on eplet mismatch load for HLA-DQB1 locus

Table S33: Patient characteristics for the rejection cohort based on eplet mismatch load for HLA-DRB1 locus

Table S34: Performance characteristics table of eplet mismatch load for HLA-A locus for rejection

Table S35: Performance characteristics table of eplet mismatch load for HLA-B locus for rejection

Table S36: Performance characteristics table of eplet mismatch load for HLA-C locus for rejection

Table S37: Performance characteristics table of eplet mismatch load for HLA-DQA1 locus for rejection

Table S38: Performance characteristics table of eplet mismatch load for HLA-DQB1 locus for rejection

Table S39: Performance characteristics table of eplet mismatch load for HLA-DRB1 locus for rejection

Table S40: Association of class I HLA Eplet mismatch load and rejection using Cox proportional regression models

Table S41: Association of class II HLA Eplet mismatch load and rejection using Cox proportional regression models

Table S42: Patient characteristics for the donor-derived cell-free DNA cohort based on eplet mismatch load for HLA-A locus

Table S43: Patient characteristics for the donor-derived cell-free DNA cohort based on eplet mismatch load for HLA-B locus

Table S44: Patient characteristics for the donor-derived cell-free DNA cohort based on eplet mismatch load for HLA-C locus

Table S45: Patient characteristics for the donor-derived cell-free DNA cohort based on eplet mismatch load for HLA-DQA1 locus

Table S46: Patient characteristics for the donor-derived cell-free DNA cohort based on eplet mismatch load for HLA-DQB1 locus

Table S47: Patient characteristics for the donor-derived cell-free DNA cohort based on eplet mismatch load for HLA-DRB1 locus

Table S48: Performance characteristics table of eplet mismatch load for HLA-A locus for donor-derived cell-free DNA

Table S49: Performance characteristics table of eplet mismatch load for HLA-B locus for donor-derived cell-free DNA

Table S50: Performance characteristics table of eplet mismatch load for HLA-C locus for donor-derived cell-free DNA

Table S51: Performance characteristics table of eplet mismatch load for HLA-DQA1 locus for donor-derived cell-free DNA

Table S52: Performance characteristics table of eplet mismatch load for HLA-DQB1 locus for donor-derived cell-free DNA

Table S53: Performance characteristics table of eplet mismatch load for HLA-DRB1 locus for donor-derived cell-free DNA

Table S54: Association of class I HLA Eplet mismatch load and elevation of donor-derived cell-free DNA using Cox proportional regression models

Table S55: Association of class II HLA Eplet mismatch load and elevation of donor-derived cell-free DNA using Cox proportional regression models

Figure S1.

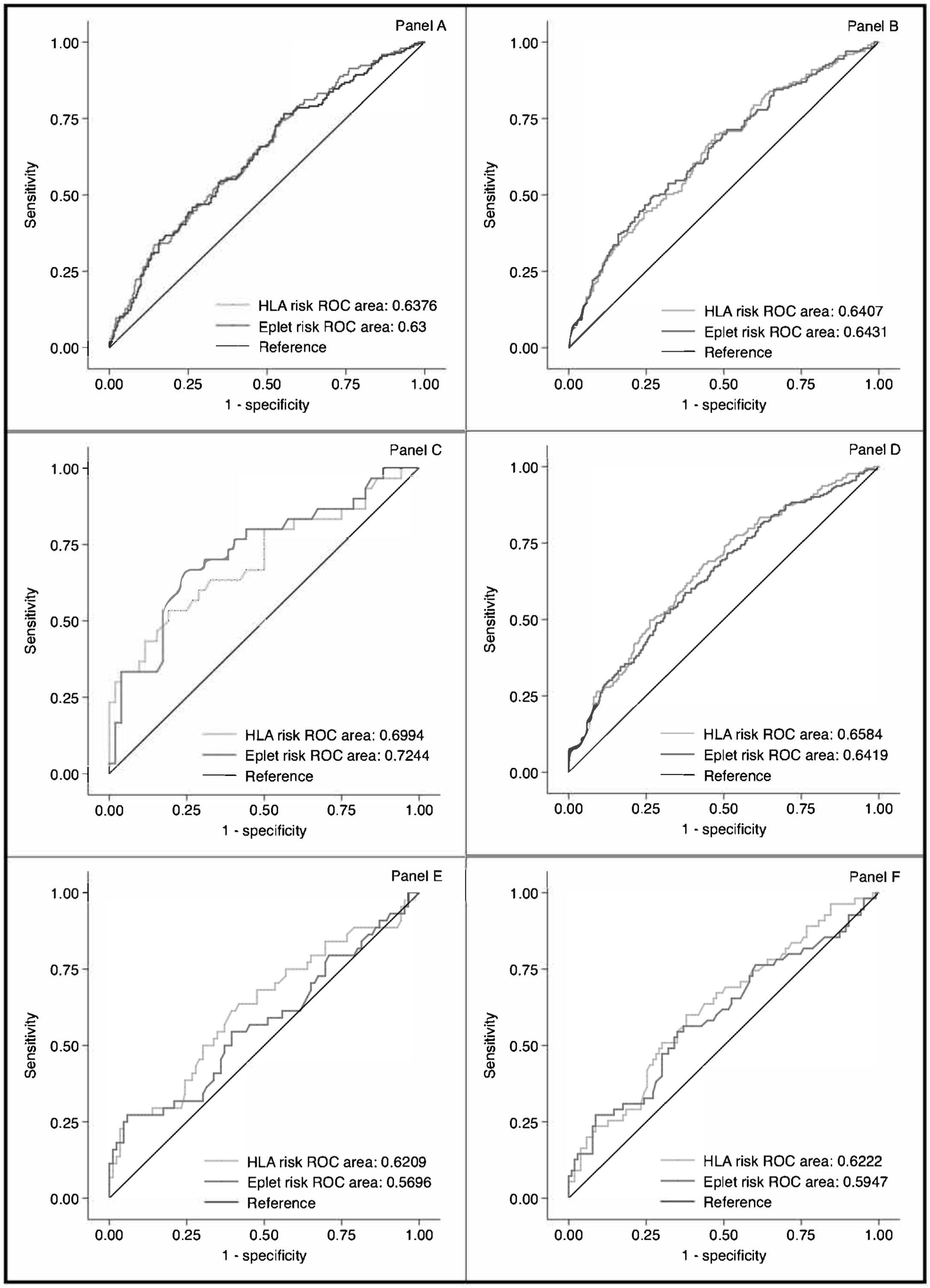

Panel A
Sensitivity
1 - specificity
HLA risk ROC area: 0.6376
Eplet risk ROC area: 0.63
Reference
Panel B
HLA risk ROC area: 0.6407
Eplet risk ROC area: 0.6431
Reference
Panel C
HLA risk ROC area: 0.6994
Eplet risk ROC area: 0.7244
Reference
Panel D
HLA risk ROC area: 0.6584
Eplet risk ROC area: 0.6419
Reference
Panel E
HLA risk ROC area: 0.6209
Eplet risk ROC area: 0.5696
Reference
Panel F
HLA risk ROC area: 0.6222
Eplet risk ROC area: 0.5947
Reference

Figure S2.

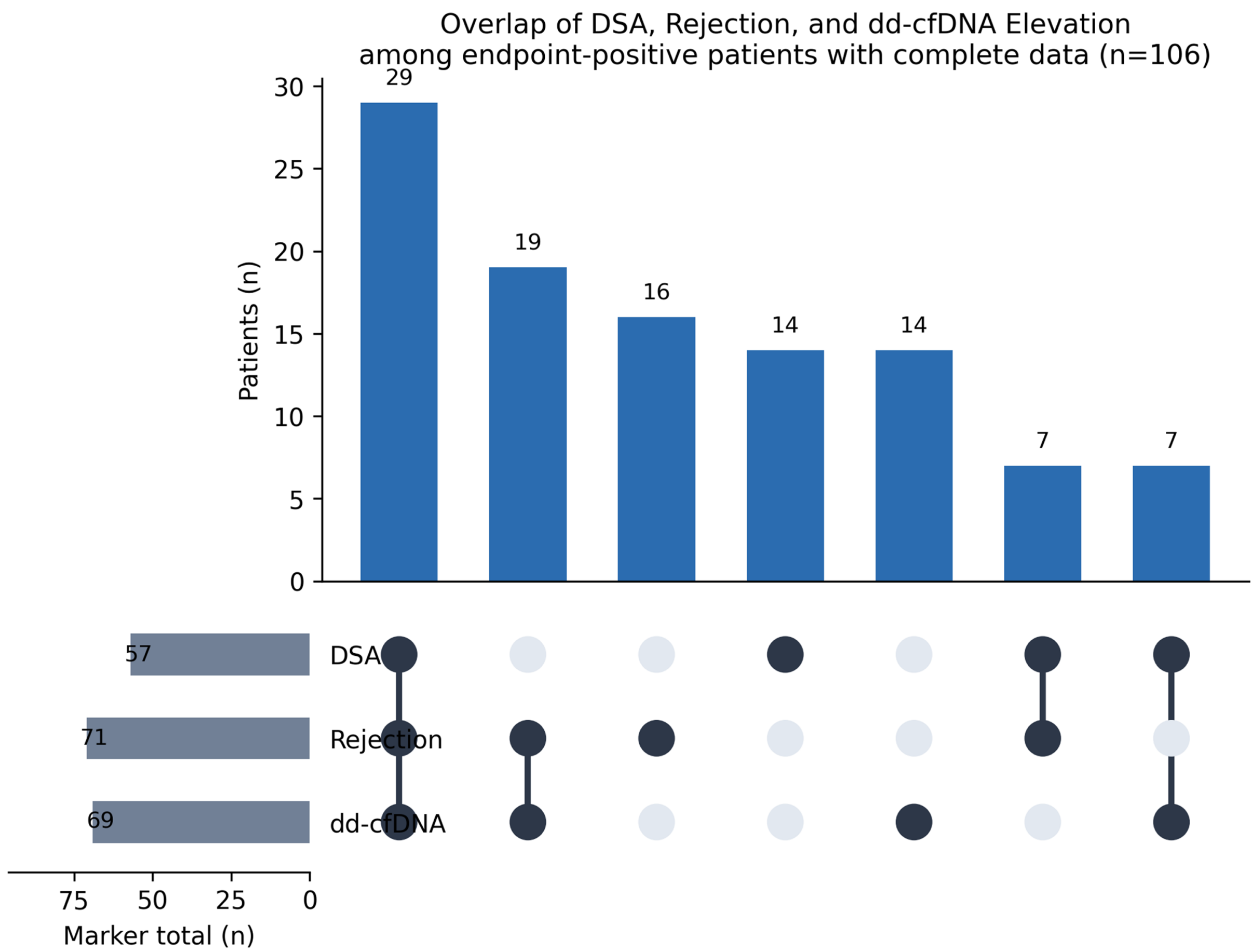

Top bars: number of patients in each exact combination. Left bars: total prevalence of each marker.

Figure S3.

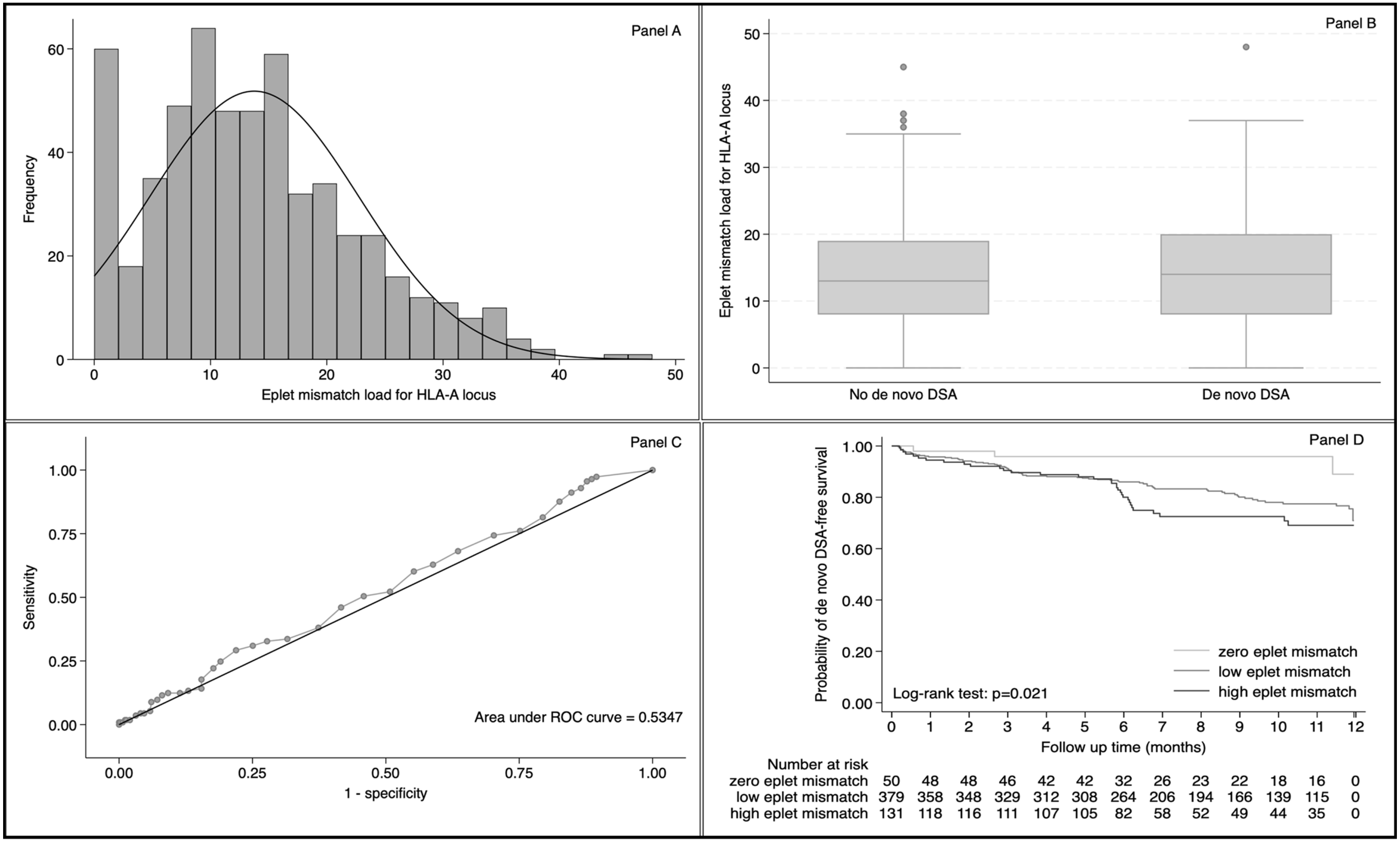
Panel A
Frequency
Eplet mismatch load for HLA-A locus
Panel B
Eplet mismatch load for HLA-A locus
No de novo DSA
De novo DSA
Panel C
Sensitivity
1 - specificity
Area under ROC curve = 0.5347
Panel D
Probability of de novo DSA-free survival
zero eplet mismatch
low eplet mismatch
high eplet mismatch
Log-rank test: p=0.021
Follow up time (months)
Number at risk
zero eplet mismatch 50 48 48 46 42 42 32 26 23 22 18 16 0
low eplet mismatch 379 358 348 329 312 308 264 206 194 166 139 115 0
high eplet mismatch 131 118 116 111 107 105 82 58 52 49 44 35 0

Figure S4.

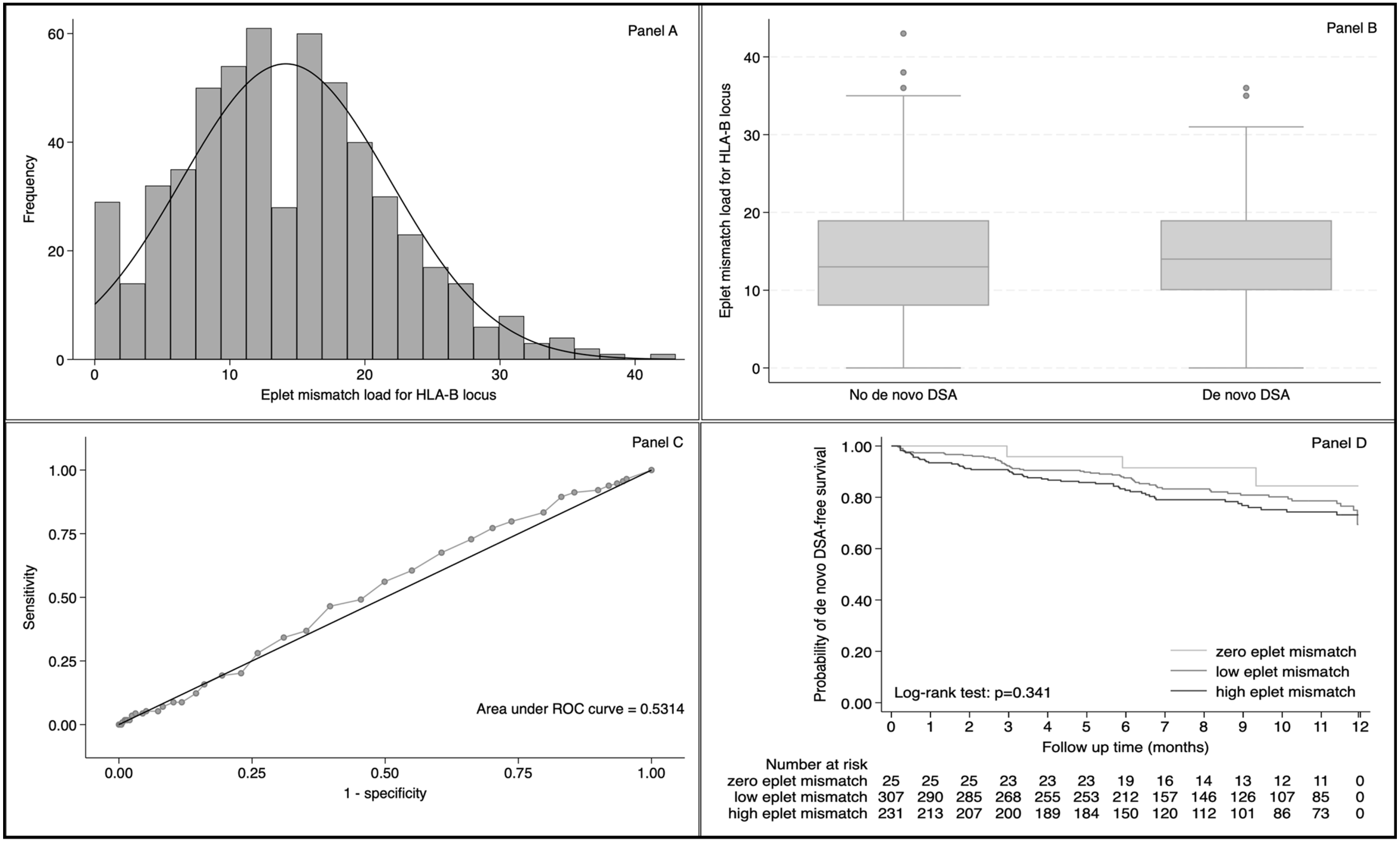
Panel A
Frequency
0
20
40
60
0
10
20
30
40
Eplet mismatch load for HLA-B locus
Panel B
Eplet mismatch load for HLA-B locus
0
10
20
30
40
No de novo DSA
De novo DSA
Panel C
Sensitivity
0.00
0.25
0.50
0.75
1.00
1 - specificity
Area under ROC curve = 0.5314
Panel D
Probability of de novo DSA-free survival
0.00
0.20
0.40
0.60
0.80
1.00
zero eplet mismatch
low eplet mismatch
high eplet mismatch
Log-rank test: p=0.341
0 1 2 3 4 5 6 7 8 9 10 11 12
Follow up time (months)
Number at risk
zero eplet mismatch 25 25 25 23 23 23 19 16 14 13 12 11 0
low eplet mismatch 307 290 285 268 255 253 212 157 146 126 107 85 0
high eplet mismatch 231 213 207 200 189 184 150 120 112 101 86 73 0

Figure S5.

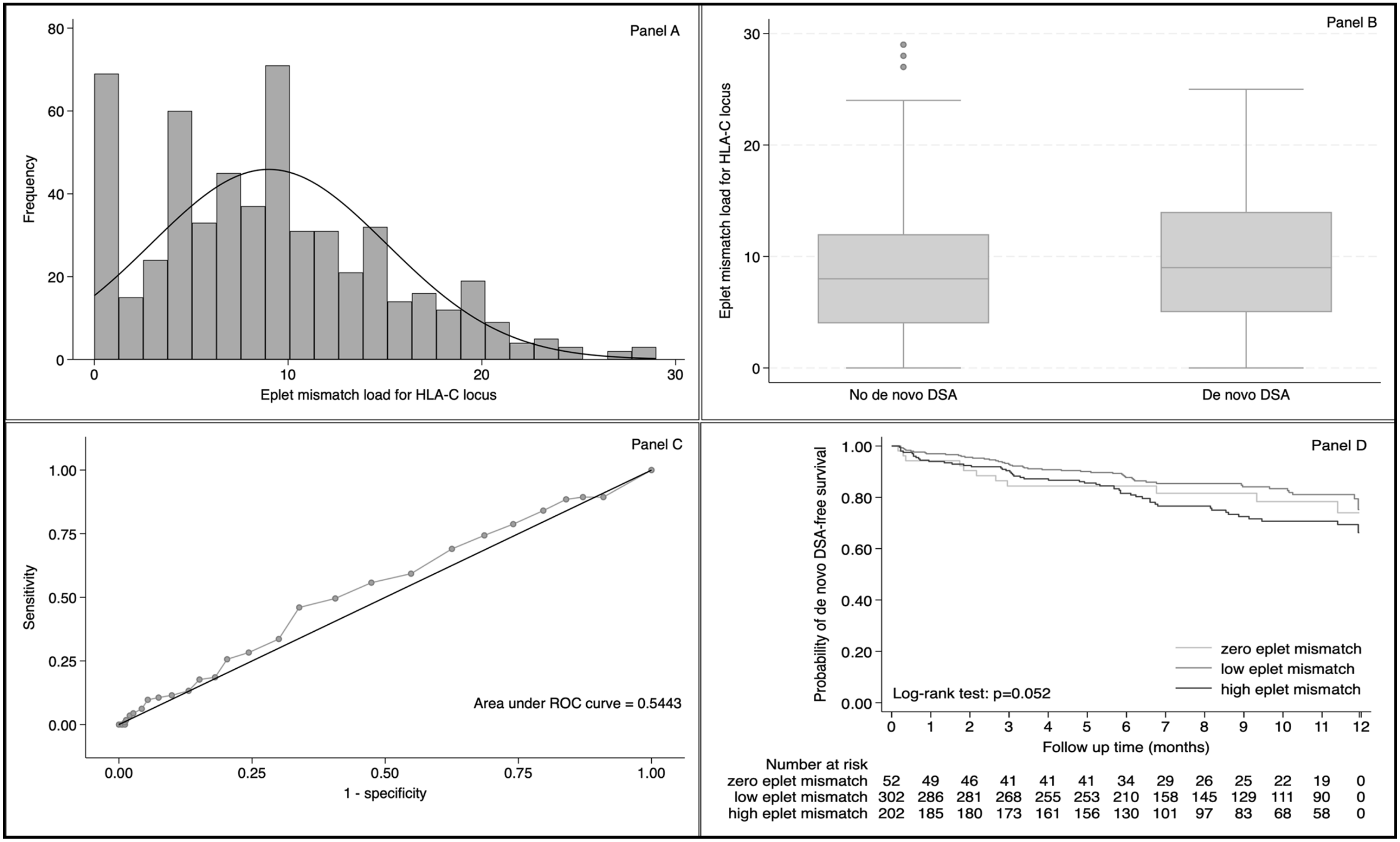
Panel A
Frequency
Eplet mismatch load for HLA-C locus
Panel B
Eplet mismatch load for HLA-C locus
No de novo DSA
De novo DSA
Panel C
Sensitivity
1 - specificity
Area under ROC curve = 0.5443
Panel D
Probability of de novo DSA-free survival
zero eplet mismatch
low eplet mismatch
high eplet mismatch
Log-rank test: p=0.052
Follow up time (months)
Number at risk
zero eplet mismatch 52 49 46 41 41 41 34 29 26 25 22 19 0
low eplet mismatch 302 286 281 268 255 253 210 158 145 129 111 90 0
high eplet mismatch 202 185 180 173 161 156 130 101 97 83 68 58 0

Figure S6.

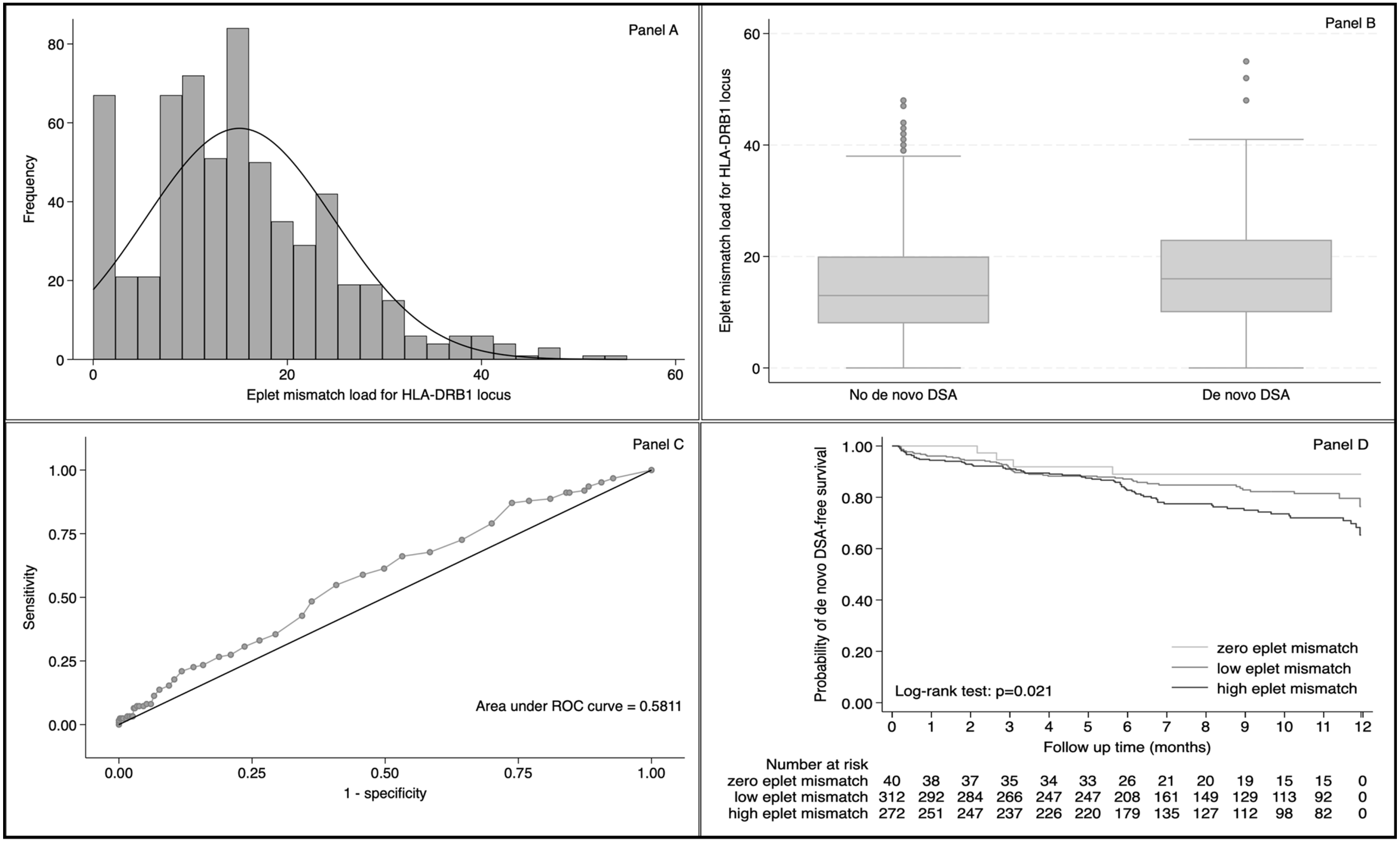

Panel A
Frequency
Eplet mismatch load for HLA-DRB1 locus
0
20
40
60
80
0
20
40
60
Panel B
Eplet mismatch load for HLA-DRB1 locus
0
20
40
60
No de novo DSA
De novo DSA
Panel C
Sensitivity
1 - specificity
0.00
0.25
0.50
0.75
1.00
Area under ROC curve = 0.5811
Panel D
Probability of de novo DSA-free survival
0.00
0.20
0.40
0.60
0.80
1.00
zero eplet mismatch
low eplet mismatch
high eplet mismatch
Log-rank test: p=0.021
0 1 2 3 4 5 6 7 8 9 10 11 12
Follow up time (months)
Number at risk
zero eplet mismatch 40 38 37 35 34 33 26 21 20 19 15 15 0
low eplet mismatch 312 292 284 266 247 247 208 161 149 129 113 92 0
high eplet mismatch 272 251 247 237 226 220 179 135 127 112 98 82 0

Figure S7.

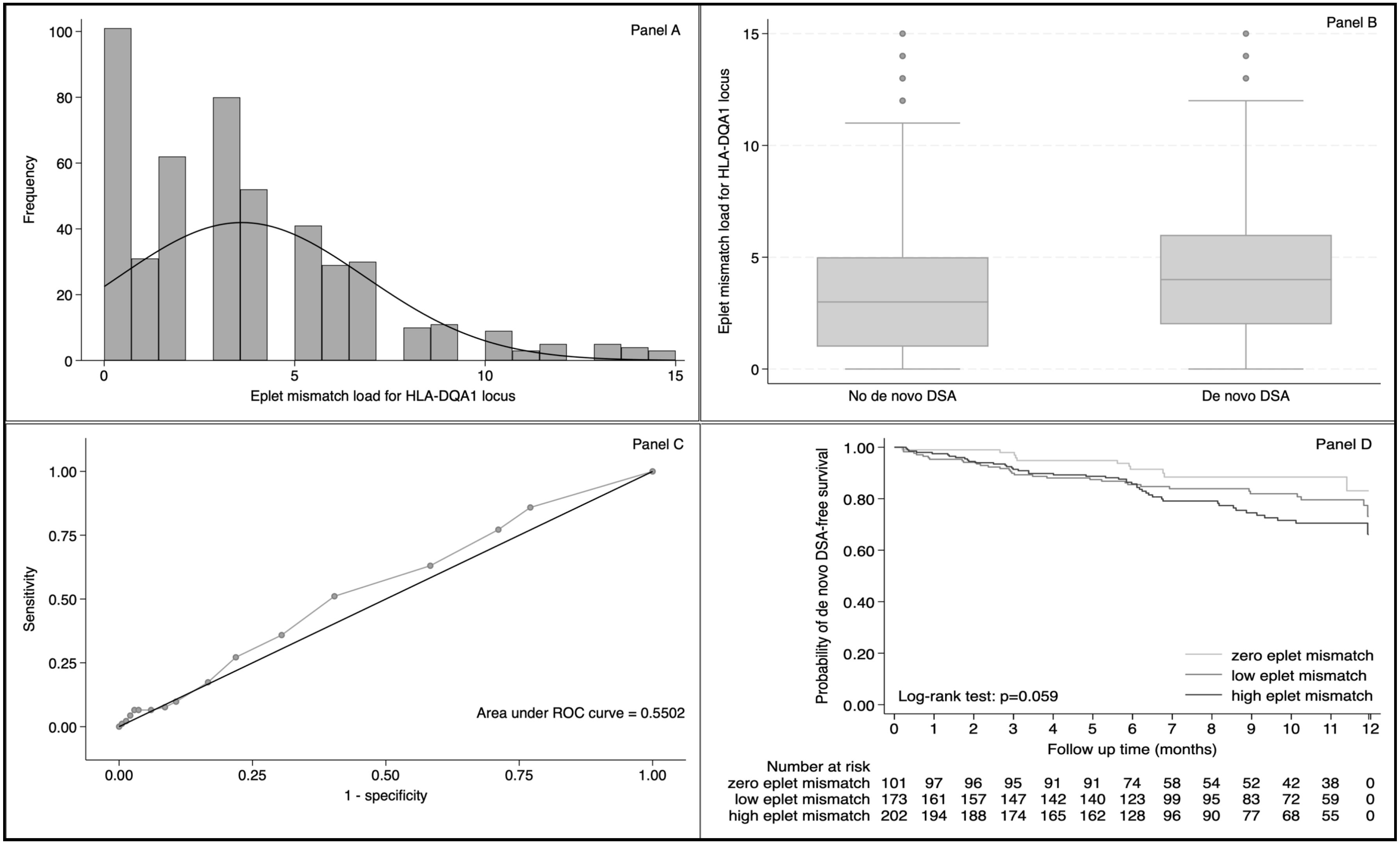
Panel A
Frequency
Eplet mismatch load for HLA-DQA1 locus
Panel B
Eplet mismatch load for HLA-DQA1 locus
No de novo DSA
De novo DSA
Panel C
Sensitivity
1 - specificity
Area under ROC curve = 0.5502
Panel D
Probability of de novo DSA-free survival
zero eplet mismatch
low eplet mismatch
high eplet mismatch
Log-rank test: p=0.059
Follow up time (months)
Number at risk
zero eplet mismatch 101 97 96 95 91 91 74 58 54 52 42 38 0
low eplet mismatch 173 161 157 147 142 140 123 99 95 83 72 59 0
high eplet mismatch 202 194 188 174 165 162 128 96 90 77 68 55 0

Figure S8.

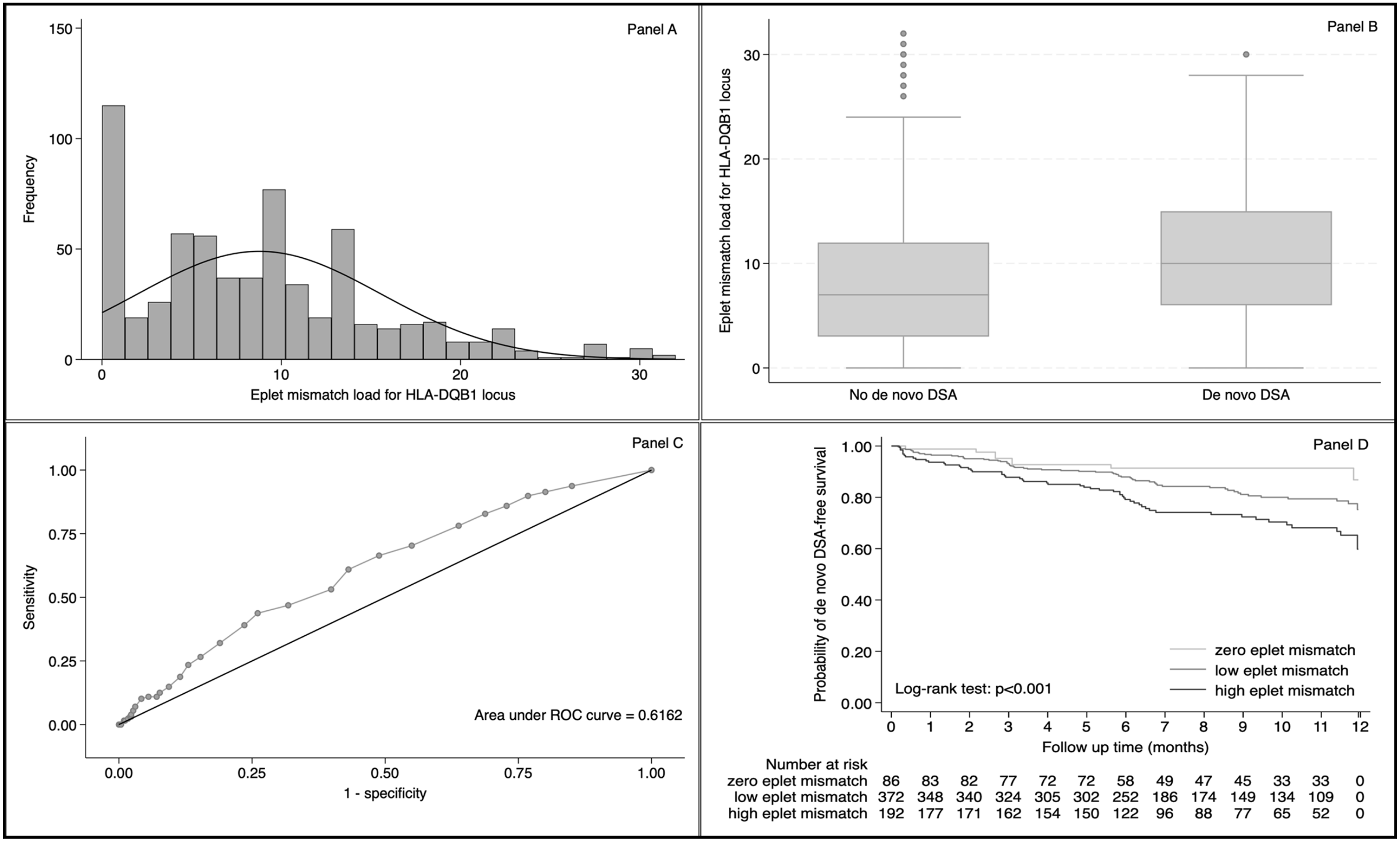

Panel A
150
100
50
0
Frequency
0
10
20
30
Eplet mismatch load for HLA-DQB1 locus
Panel B
30
20
10
0
Eplet mismatch load for HLA-DQB1 locus
No de novo DSA
De novo DSA
Panel C
1.00
0.75
0.50
0.25
0.00
Sensitivity
Area under ROC curve = 0.6162
0.00
0.25
0.50
0.75
1.00
1 - specificity
Panel D
1.00
0.80
0.60
0.40
0.20
0.00
Probability of de novo DSA-free survival
zero eplet mismatch
low eplet mismatch
high eplet mismatch
Log-rank test: p<0.001
0 1 2 3 4 5 6 7 8 9 10 11 12
Follow up time (months)
Number at risk
zero eplet mismatch 86 83 82 77 72 72 58 49 47 45 33 33 0
low eplet mismatch 372 348 340 324 305 302 252 186 174 149 134 109 0
high eplet mismatch 192 177 171 162 154 150 122 96 88 77 65 52 0

Figure S9.

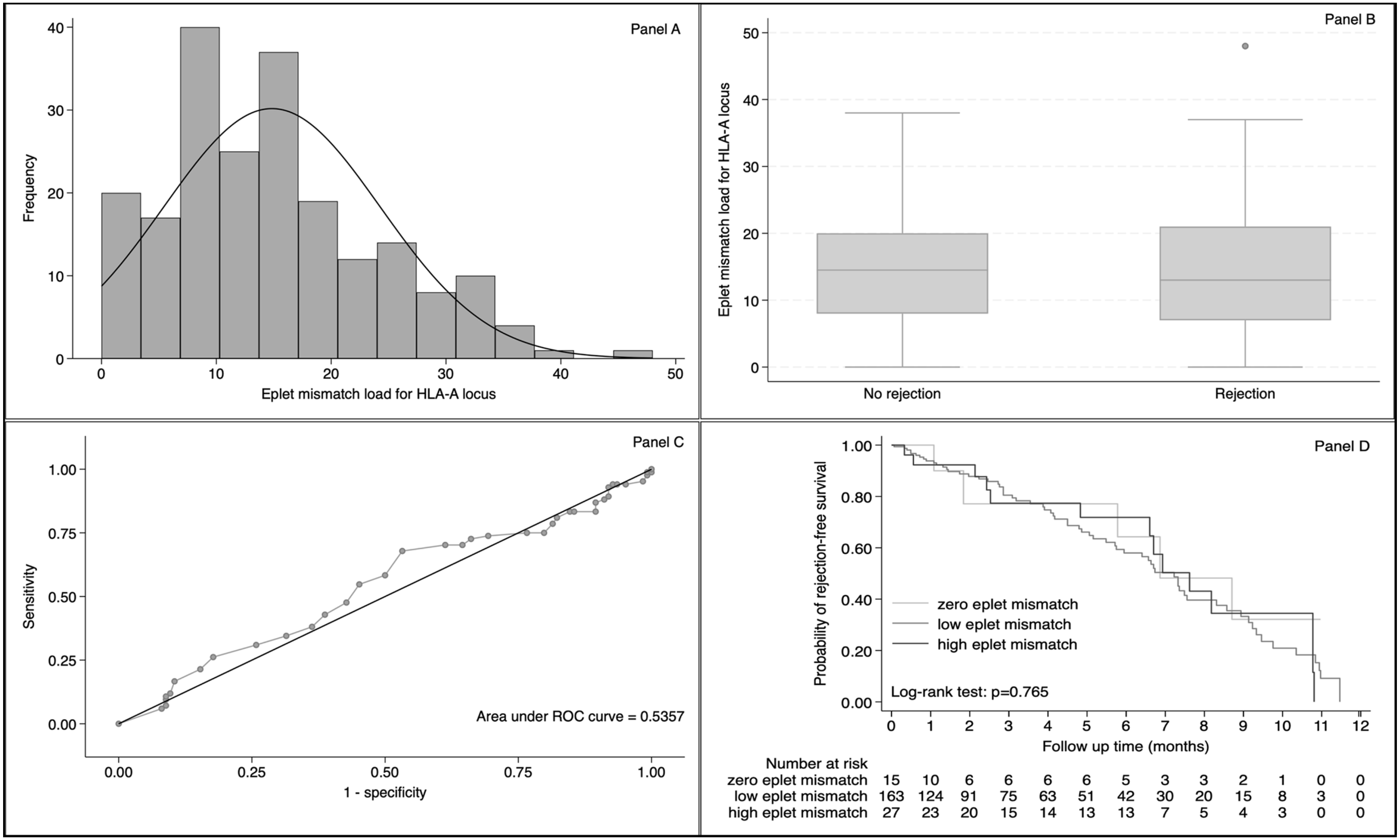

Panel A
Frequency
Eplet mismatch load for HLA-A locus
Panel B
Eplet mismatch load for HLA-A locus
No rejection
Rejection
Panel C
Sensitivity
1 - specificity
Area under ROC curve = 0.5357
Panel D
Probability of rejection-free survival
zero eplet mismatch
low eplet mismatch
high eplet mismatch
Log-rank test: p=0.765
Follow up time (months)
Number at risk
zero eplet mismatch 15 10 6 6 6 6 5 3 3 2 1 0 0
low eplet mismatch 163 124 91 75 63 51 42 30 20 15 8 3 0
high eplet mismatch 27 23 20 15 14 13 13 7 5 4 3 0 0

Figure S10.

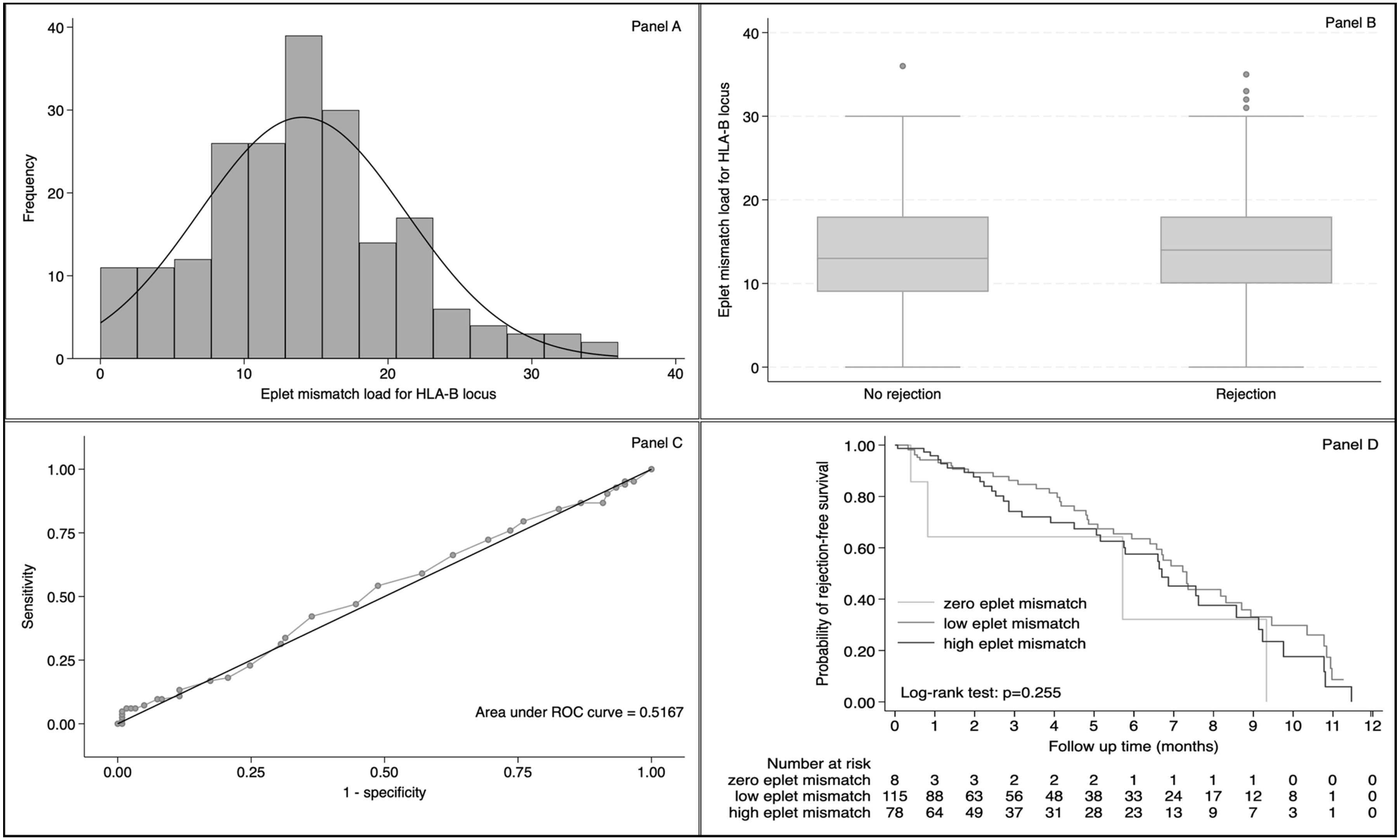
Panel A
Frequency
Eplet mismatch load for HLA-B locus
Panel B
Eplet mismatch load for HLA-B locus
No rejection
Rejection
Panel C
Sensitivity
1 - specificity
Area under ROC curve = 0.5167
Panel D
Probability of rejection-free survival
zero eplet mismatch
low eplet mismatch
high eplet mismatch
Log-rank test: p=0.255
Follow up time (months)
Number at risk
zero eplet mismatch 8 3 3 2 2 2 1 1 1 1 0 0 0
low eplet mismatch 115 88 63 56 48 38 33 24 17 12 8 1 0
high eplet mismatch 78 64 49 37 31 28 23 13 9 7 3 1 0

Figure S11.

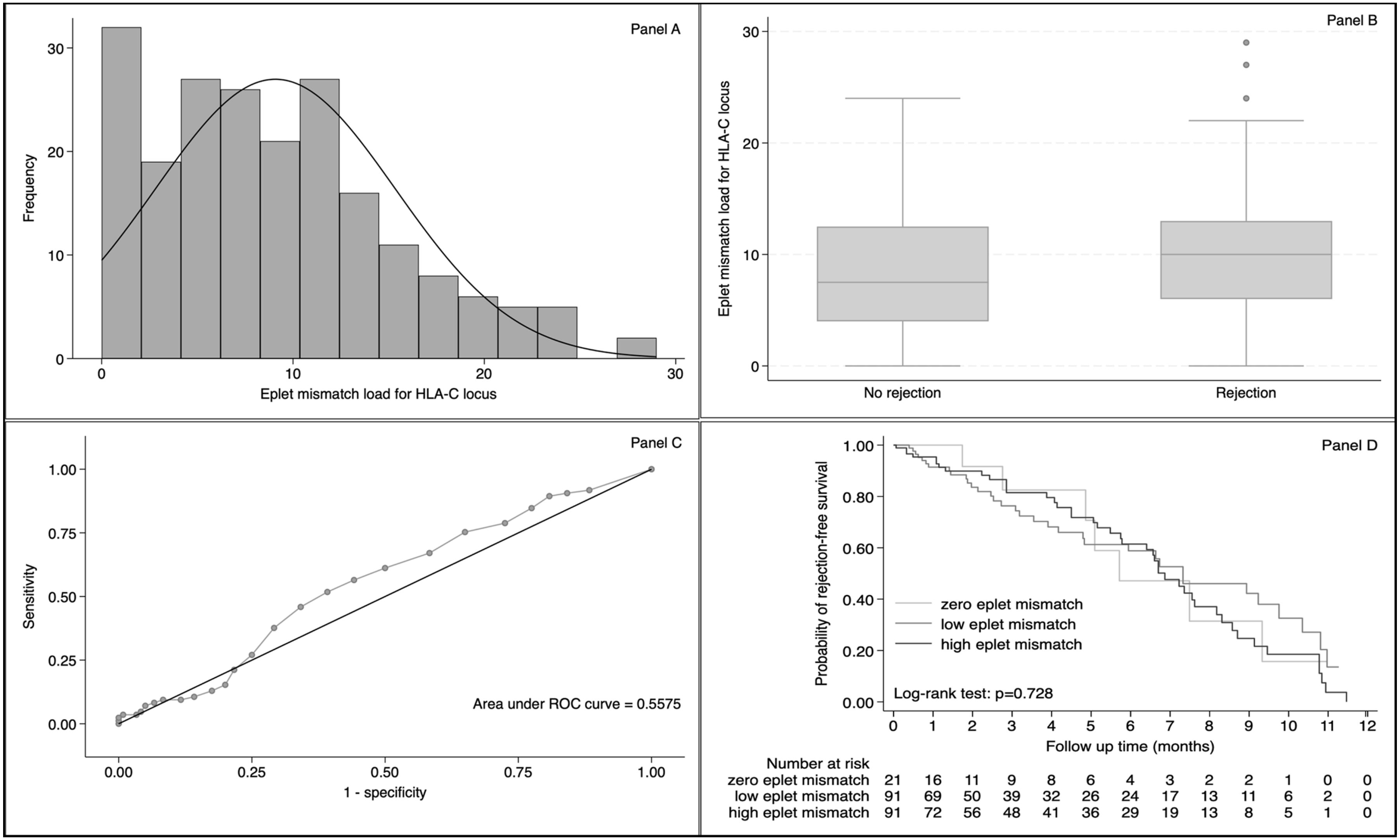

Panel A
Frequency
0
10
20
30
Eplet mismatch load for HLA-C locus
Panel B
Eplet mismatch load for HLA-C locus
No rejection
Rejection
Panel C
Sensitivity
0.00
0.25
0.50
0.75
1.00
1 - specificity
Area under ROC curve = 0.5575
Panel D
Probability of rejection-free survival
0.00
0.20
0.40
0.60
0.80
1.00
zero eplet mismatch
low eplet mismatch
high eplet mismatch
Log-rank test: p=0.728
0 1 2 3 4 5 6 7 8 9 10 11 12
Follow up time (months)
Number at risk
zero eplet mismatch 21 16 11 9 8 6 4 3 2 2 1 0 0
low eplet mismatch 91 69 50 39 32 26 24 17 13 11 6 2 0
high eplet mismatch 91 72 56 48 41 36 29 19 13 8 5 1 0

Figure S12.

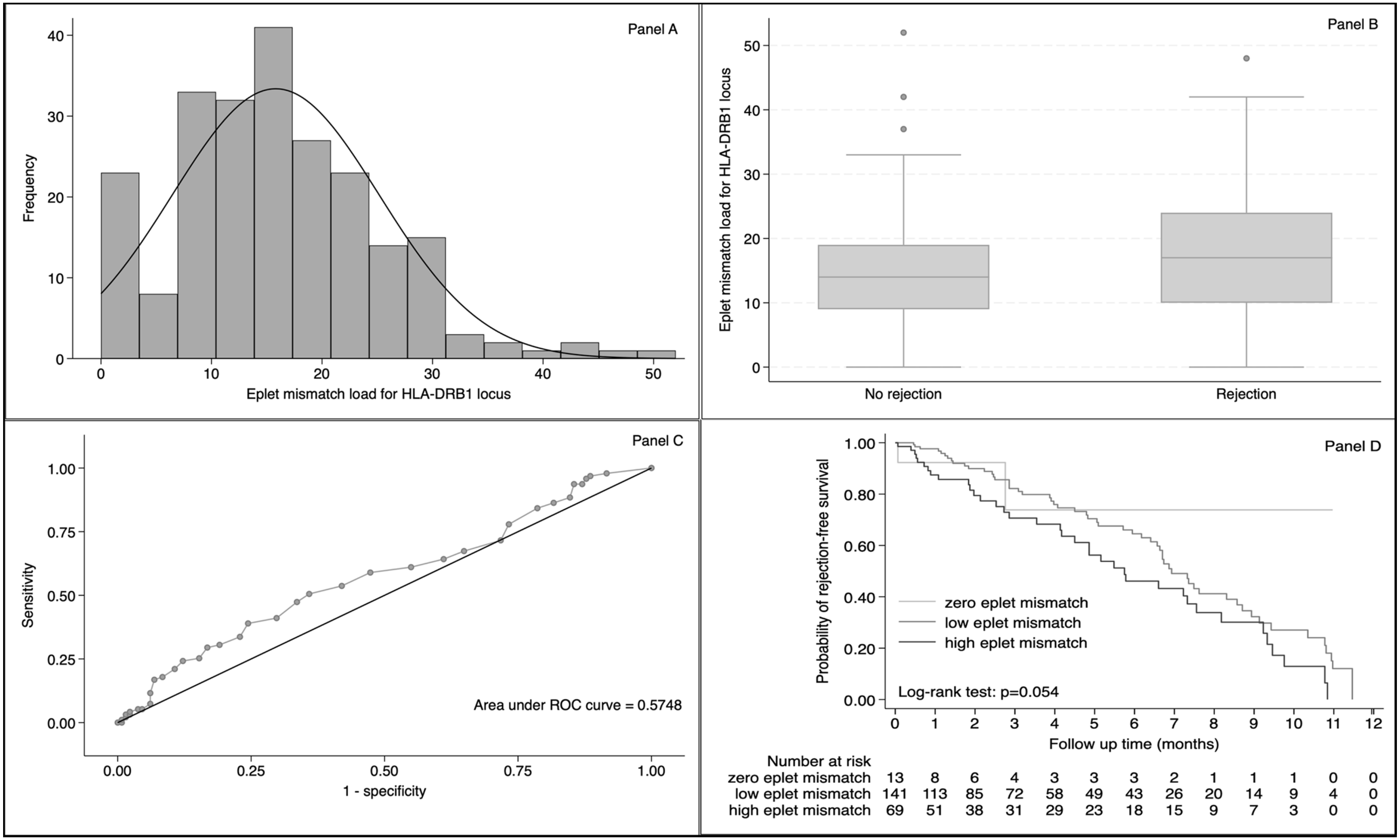
Panel A
Frequency
0
10
20
30
40
0
10
20
30
40
50
Eplet mismatch load for HLA-DRB1 locus
Panel B
Eplet mismatch load for HLA-DRB1 locus
0
10
20
30
40
50
No rejection
Rejection
Panel C
Sensitivity
0.00
0.25
0.50
0.75
1.00
0.00
0.25
0.50
0.75
1.00
1 - specificity
Area under ROC curve = 0.5748
Panel D
Probability of rejection-free survival
0.00
0.20
0.40
0.60
0.80
1.00
zero eplet mismatch
low eplet mismatch
high eplet mismatch
Log-rank test: p=0.054
0 1 2 3 4 5 6 7 8 9 10 11 12
Follow up time (months)
Number at risk
zero eplet mismatch 13 8 6 4 3 3 3 2 1 1 1 0 0
low eplet mismatch 141 113 85 72 58 49 43 26 20 14 9 4 0
high eplet mismatch 69 51 38 31 29 23 18 15 9 7 3 0 0

Figure S13.

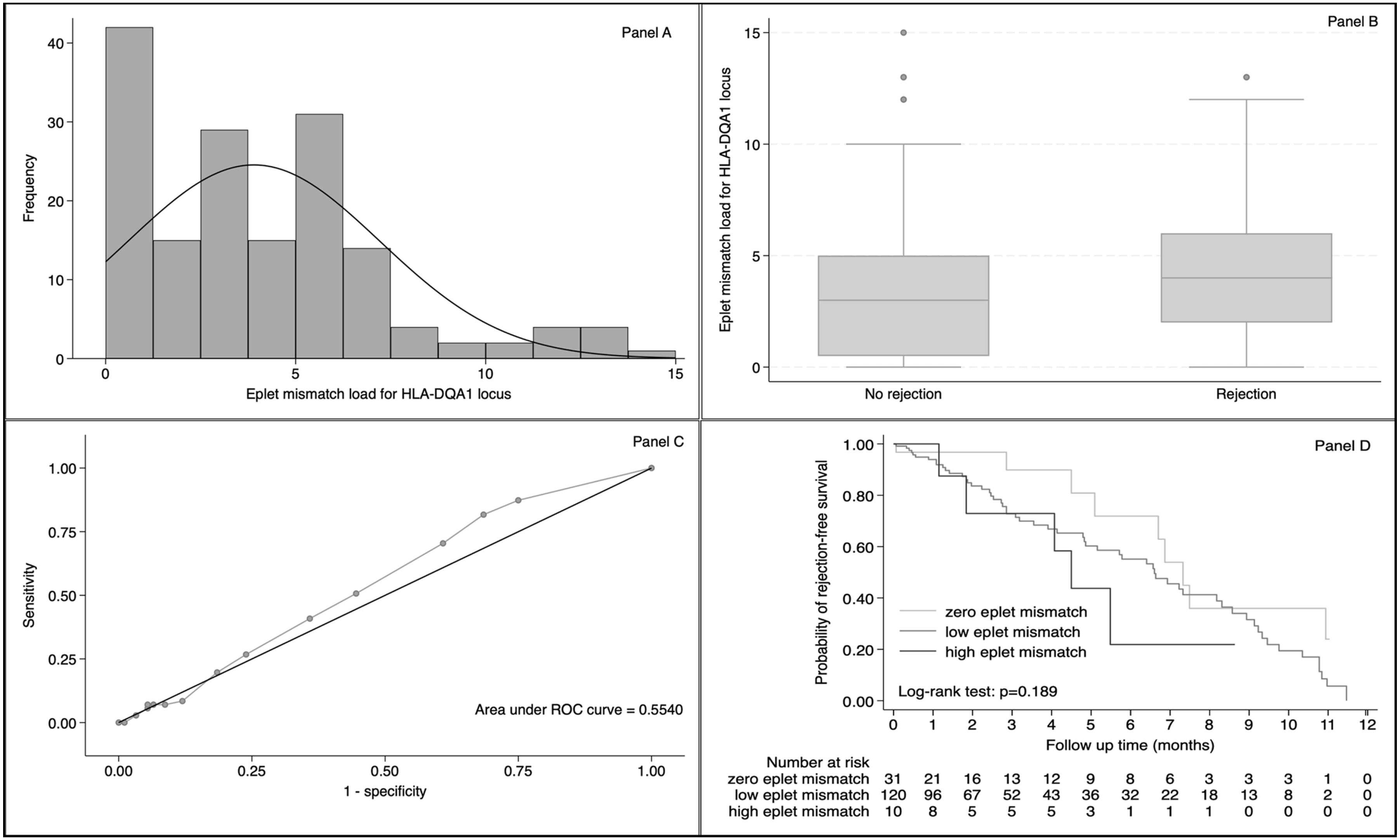

Panel A
Frequency
40
30
20
10
0
0
5
10
15
Eplet mismatch load for HLA-DQA1 locus
Panel B
Eplet mismatch load for HLA-DQA1 locus
15
10
5
0
No rejection
Rejection
Panel C
Sensitivity
1.00
0.75
0.50
0.25
0.00
Area under ROC curve = 0.5540
0.00
0.25
0.50
0.75
1.00
1 - specificity
Panel D
Probability of rejection-free survival
1.00
0.80
0.60
0.40
0.20
0.00
zero eplet mismatch
low eplet mismatch
high eplet mismatch
Log-rank test: p=0.189
0 1 2 3 4 5 6 7 8 9 10 11 12
Follow up time (months)
Number at risk
zero eplet mismatch 31 21 16 13 12 9 8 6 3 3 3 1 0
low eplet mismatch 120 96 67 52 43 36 32 22 18 13 8 2 0
high eplet mismatch 10 8 5 5 5 3 1 1 1 0 0 0 0

Figure S14.

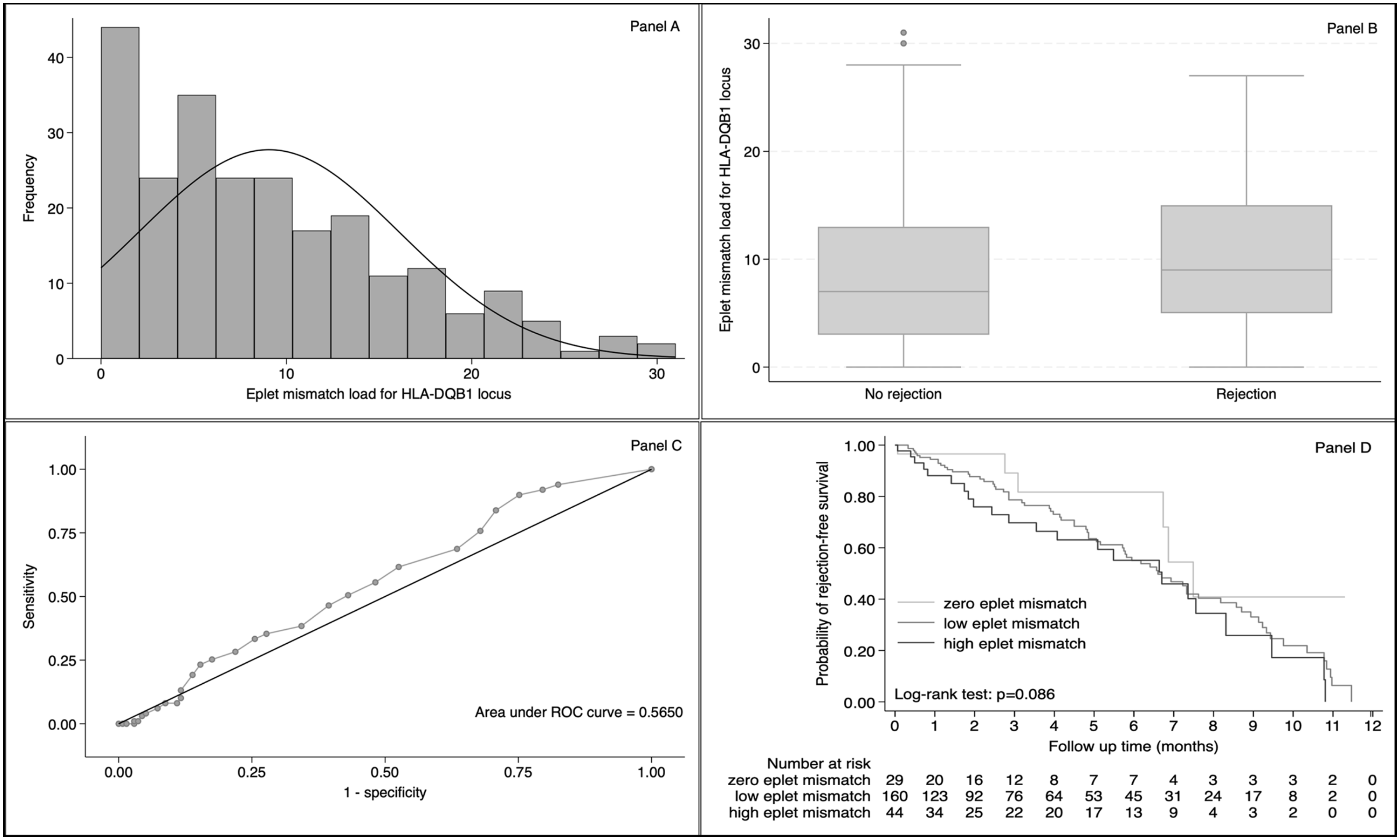

Panel A
Frequency
Eplet mismatch load for HLA-DQB1 locus
Panel B
Eplet mismatch load for HLA-DQB1 locus
No rejection
Rejection
Panel C
Sensitivity
1 - specificity
Area under ROC curve = 0.5650
Panel D
Probability of rejection-free survival
zero eplet mismatch
low eplet mismatch
high eplet mismatch
Log-rank test: p=0.086
Follow up time (months)
Number at risk
zero eplet mismatch 29 20 16 12 8 7 7 4 3 3 3 2 0
low eplet mismatch 160 123 92 76 64 53 45 31 24 17 8 2 0
high eplet mismatch 44 34 25 22 20 17 13 9 4 3 2 0 0

Figure S15.

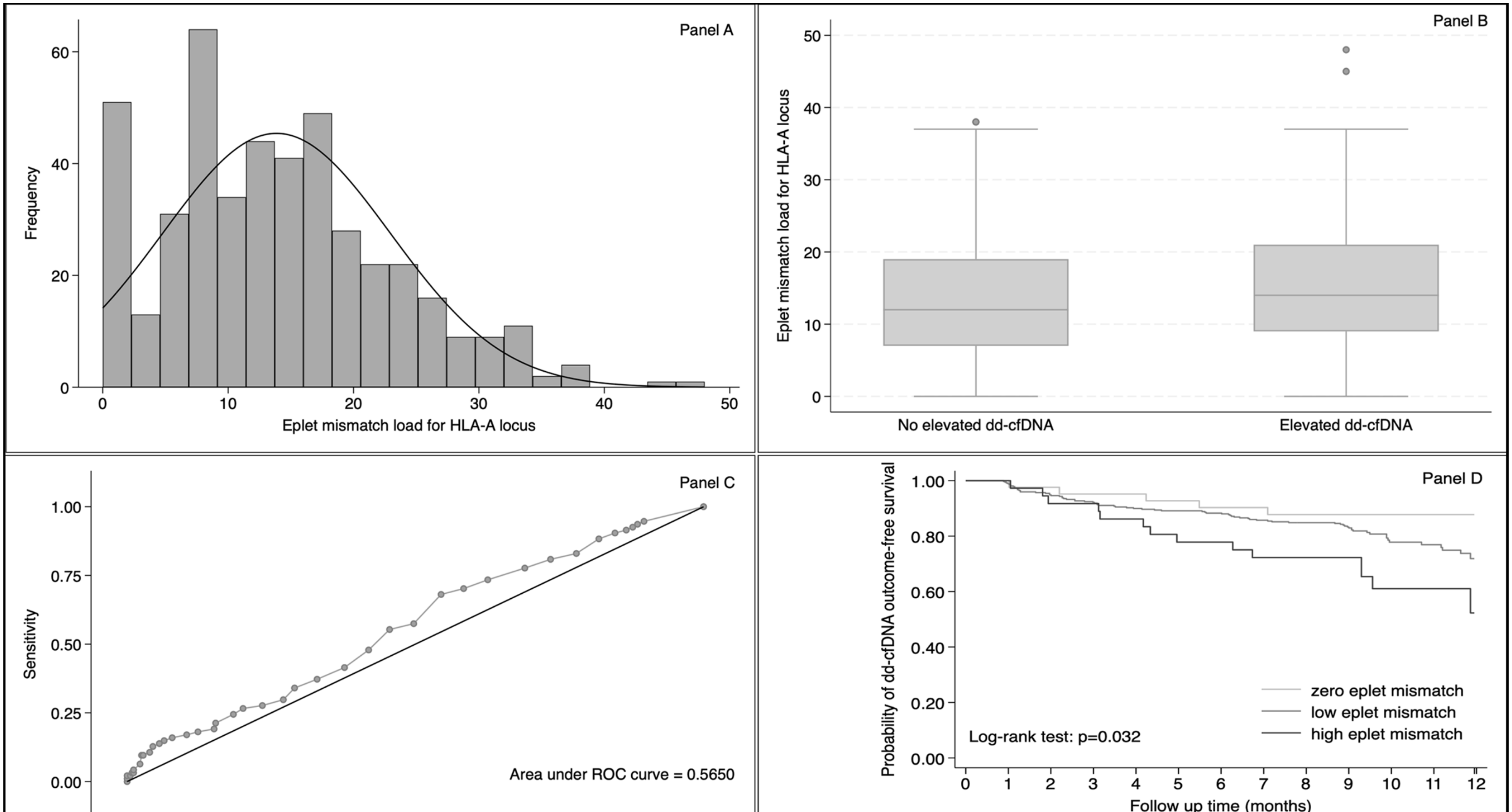

Number at risk	0	1	2	3	4	5	6	7	8	9	10	11	12
zero eplet mismatch	42	42	40	39	39	38	36	36	35	32	16	9	0
low eplet mismatch	373	368	351	337	327	323	312	297	292	243	103	82	0
high eplet mismatch	37	37	33	33	31	28	28	26	26	25	13	11	0

Figure S16.

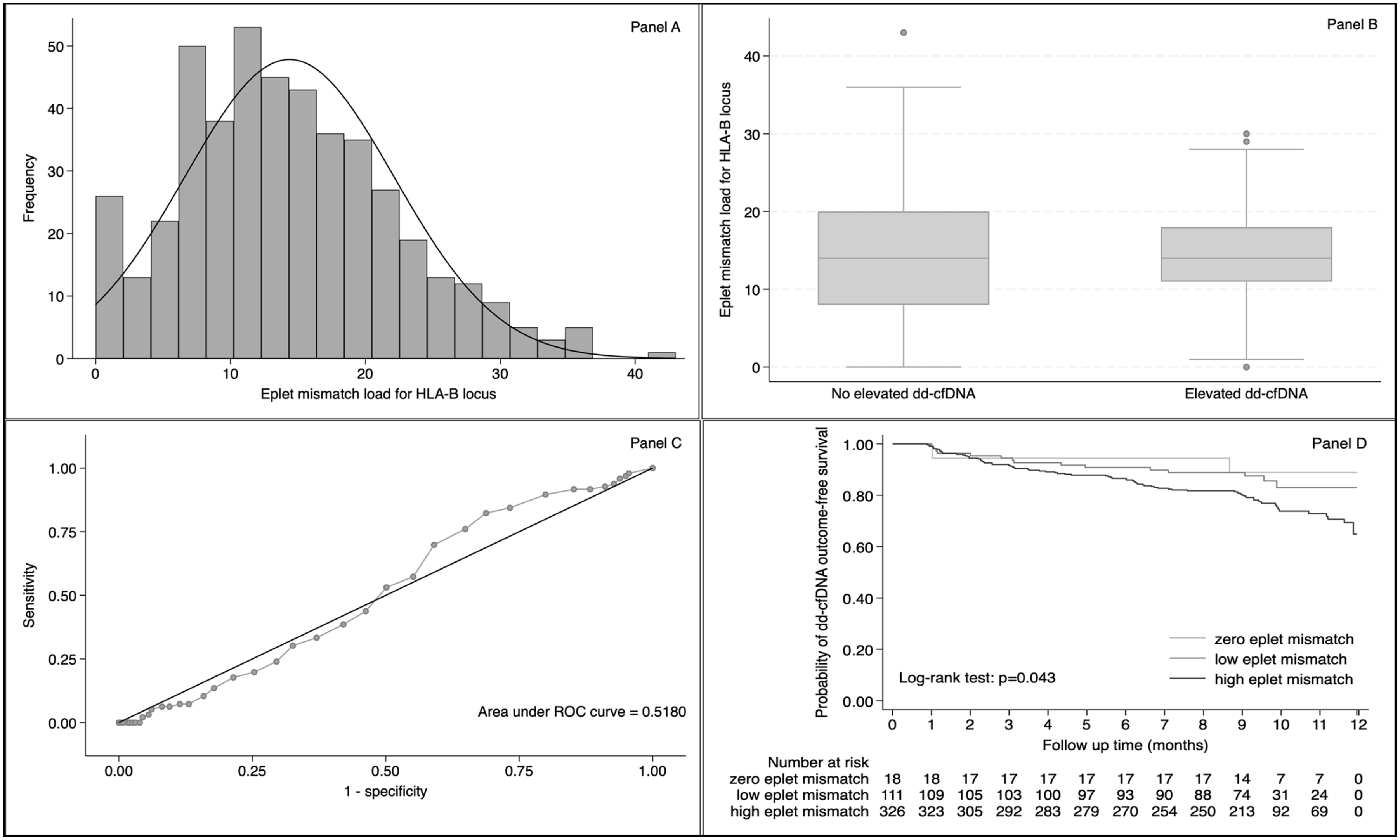

Panel A
Frequency
0
10
20
30
40
50
0
10
20
30
40
Eplet mismatch load for HLA-B locus
Panel B
Eplet mismatch load for HLA-B locus
0
10
20
30
40
No elevated dd-cfDNA
Elevated dd-cfDNA
Panel C
Sensitivity
0.00
0.25
0.50
0.75
1.00
1 - specificity
Area under ROC curve = 0.5180
Panel D
Probability of dd-cfDNA outcome-free survival
0.00
0.20
0.40
0.60
0.80
1.00
0 1 2 3 4 5 6 7 8 9 10 11 12
Follow up time (months)
zero eplet mismatch
low eplet mismatch
high eplet mismatch
Log-rank test: p=0.043
Number at risk
zero eplet mismatch 18 18 17 17 17 17 17 17 17 14 7 7 0
low eplet mismatch 111 109 105 103 100 97 93 90 88 74 31 24 0
high eplet mismatch 326 323 305 292 283 279 270 254 250 213 92 69 0

Figure S17.

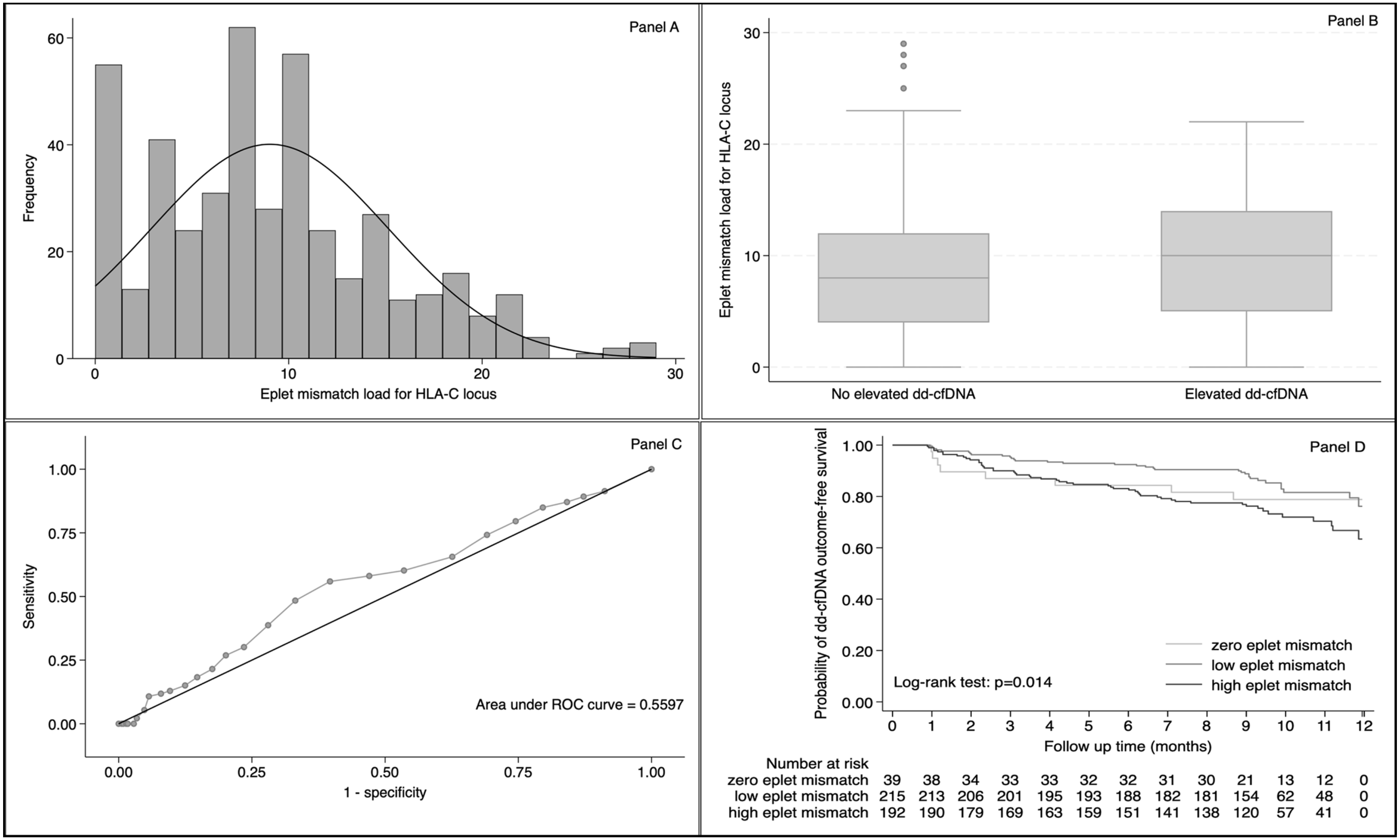

Panel A
Frequency
Eplet mismatch load for HLA-C locus
Panel B
Eplet mismatch load for HLA-C locus
No elevated dd-cfDNA
Elevated dd-cfDNA
Panel C
Sensitivity
1 - specificity
Area under ROC curve = 0.5597
Panel D
Probability of dd-cfDNA outcome-free survival
zero eplet mismatch
low eplet mismatch
high eplet mismatch
Log-rank test: p=0.014
Follow up time (months)
Number at risk
zero eplet mismatch 39 38 34 33 33 32 32 31 30 21 13 12 0
low eplet mismatch 215 213 206 201 195 193 188 182 181 154 62 48 0
high eplet mismatch 192 190 179 169 163 159 151 141 138 120 57 41 0

Figure S18.

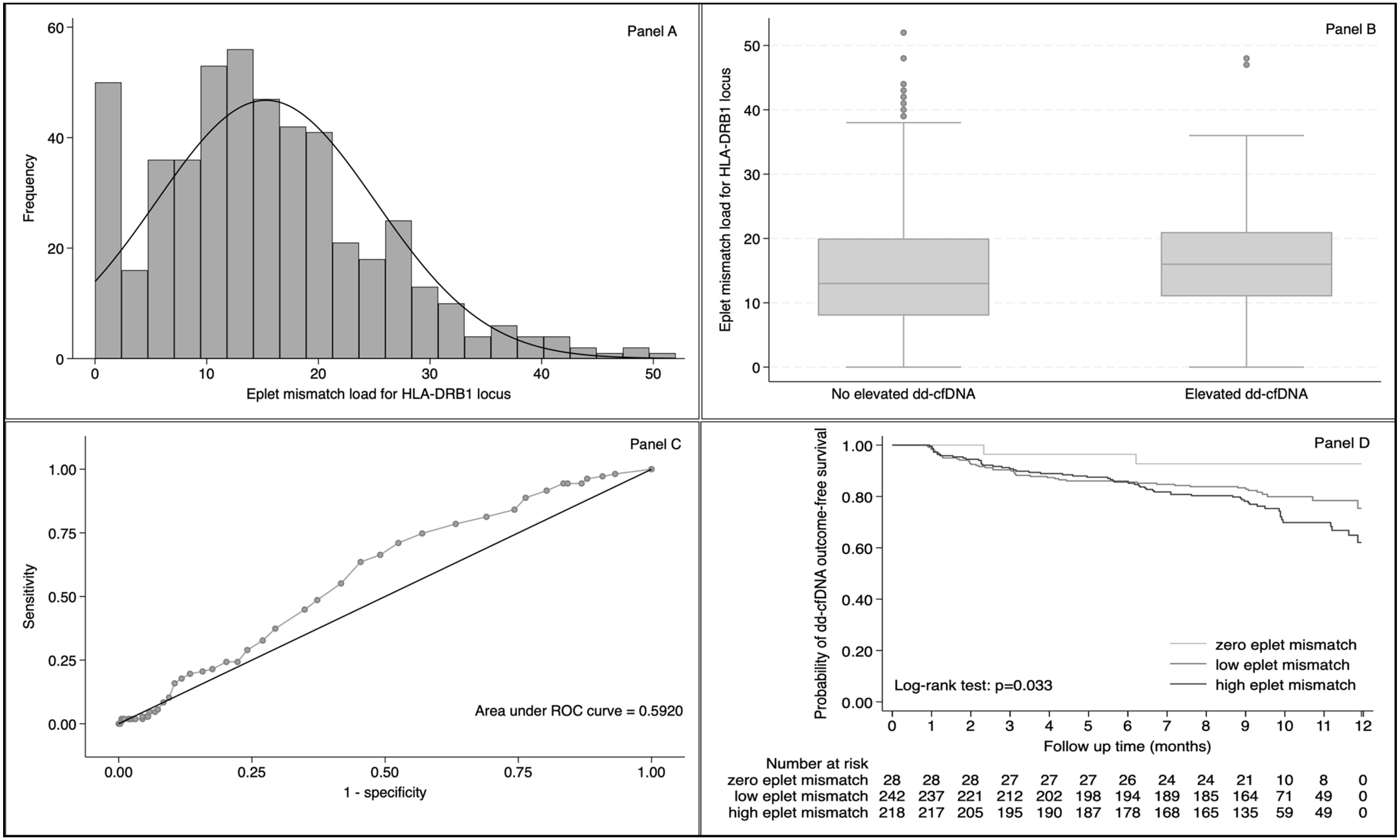
Panel A
60
40
20
0
Frequency
0
10
20
30
40
50
Eplet mismatch load for HLA-DRB1 locus
Panel B
50
40
30
20
10
0
Eplet mismatch load for HLA-DRB1 locus
No elevated dd-cfDNA
Elevated dd-cfDNA
Panel C
1.00
0.75
0.50
0.25
0.00
Sensitivity
0.00
0.25
0.50
0.75
1.00
1 - specificity
Area under ROC curve = 0.5920
Panel D
1.00
0.80
0.60
0.40
0.20
0.00
Probability of dd-cfDNA outcome-free survival
zero eplet mismatch
low eplet mismatch
high eplet mismatch
Log-rank test: p=0.033
0 1 2 3 4 5 6 7 8 9 10 11 12
Follow up time (months)
Number at risk
zero eplet mismatch 28 28 28 27 27 27 26 24 24 21 10 8 0
low eplet mismatch 242 237 221 212 202 198 194 189 185 164 71 49 0
high eplet mismatch 218 217 205 195 190 187 178 168 165 135 59 49 0

Figure S19.

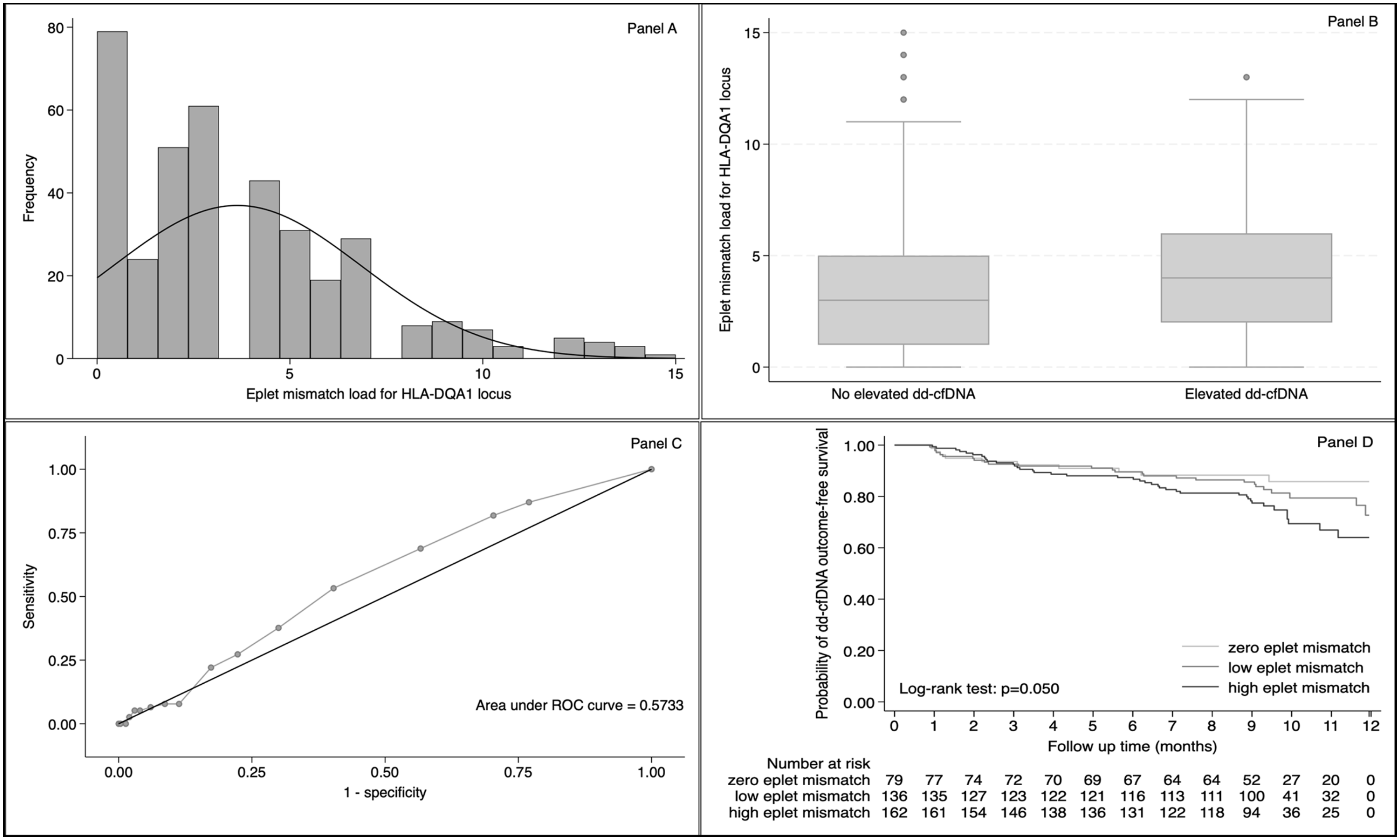
Panel A
Frequency
0
20
40
60
80
0
5
10
15
Eplet mismatch load for HLA-DQA1 locus
Panel B
Eplet mismatch load for HLA-DQA1 locus
0
5
10
15
No elevated dd-cfDNA
Elevated dd-cfDNA
Panel C
Sensitivity
0.00
0.25
0.50
0.75
1.00
Area under ROC curve = 0.5733
1 - specificity
Panel D
Probability of dd-cfDNA outcome-free survival
0.00
0.20
0.40
0.60
0.80
1.00
zero eplet mismatch
low eplet mismatch
high eplet mismatch
Log-rank test: p=0.050
0 1 2 3 4 5 6 7 8 9 10 11 12
Follow up time (months)
Number at risk
zero eplet mismatch 79 77 74 72 70 69 67 64 64 52 27 20 0
low eplet mismatch 136 135 127 123 122 121 116 113 111 100 41 32 0
high eplet mismatch 162 161 154 146 138 136 131 122 118 94 36 25 0

Figure S20.

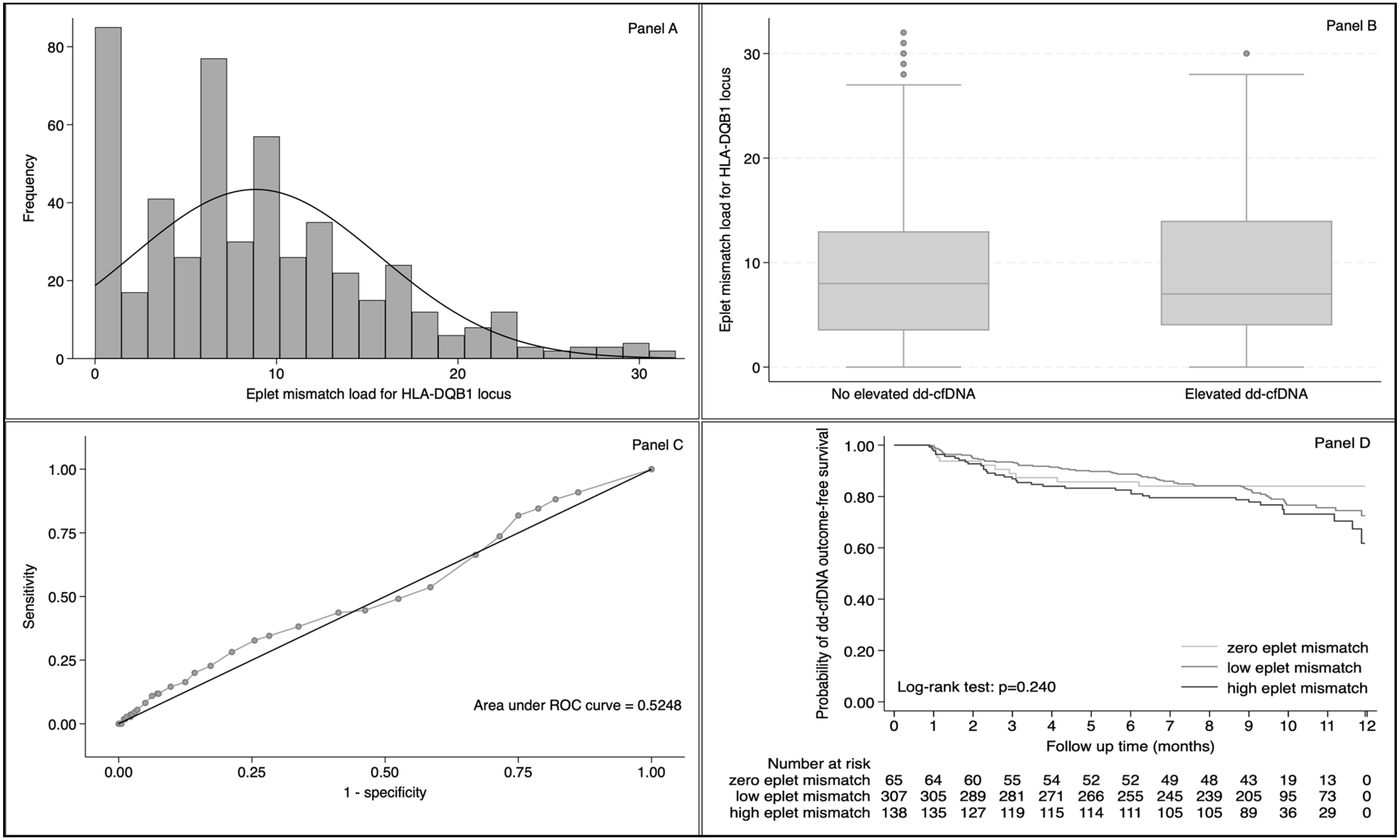
Panel A
Frequency
0
20
40
60
80
0
10
20
30
Eplet mismatch load for HLA-DQB1 locus
Panel B
Eplet mismatch load for HLA-DQB1 locus
0
10
20
30
No elevated dd-cfDNA
Elevated dd-cfDNA
Panel C
Sensitivity
0.00
0.25
0.50
0.75
1.00
1 - specificity
Area under ROC curve = 0.5248
Panel D
Probability of dd-cfDNA outcome-free survival
0.00
0.20
0.40
0.60
0.80
1.00
zero eplet mismatch
low eplet mismatch
high eplet mismatch
Log-rank test: p=0.240
0 1 2 3 4 5 6 7 8 9 10 11 12
Follow up time (months)
Number at risk
zero eplet mismatch 65 64 60 55 54 52 52 49 48 43 19 13 0
low eplet mismatch 307 305 289 281 271 266 255 245 239 205 95 73 0
high eplet mismatch 138 135 127 119 115 114 111 105 105 89 36 29 0

Table S1: Patient characteristics based on eplet mismatch load for HLA-A locus for primary outcome

	Zero Eplet Mismatch Group	Low Eplet Mismatch Group	High Eplet Mismatch Group	p-value
**N**	52	351	161	
**<u>Recipients' characteristics</u>**				
**Age (years), mean (SD)**	55.8 (14.6)	49.4 (15.1)	48.8 (15.1)	0.011
**Sex (N (%))**				0.32
male	34 (65.4%)	210 (59.8%)	107 (66.5%)	
female	18 (34.6%)	141 (40.2%)	54 (33.5%)	
**Race/ethnicity (N (%))**				0.12
White	43 (86.0%)	240 (70.4%)	96 (63.6%)	
Hispanic	6 (12.0%)	70 (20.5%)	31 (20.5%)	
African American	0 (0.0%)	7 (2.1%)	4 (2.6%)	
Asian	1 (2.0%)	12 (3.5%)	8 (5.3%)	
Other	0 (0.0%)	11 (3.2%)	12 (7.9%)	
Unknown	0 (0.0%)	1 (0.3%)	0 (0.0%)	
**Body Mass Index (kg/m2), mean (SD)**	28.6 (5.2)	28.8 (5.9)	29.0 (5.6)	0.89
**Dialysis vintage (months), median (IQR)**	27.1 (14.4-43.6)	29.7 (13.9-48.2)	34.3 (14.6-56.2)	0.51
**Cause of kidney failure (N (%))**				0.769
Diabetes mellitus	22 (42.3%)	109 (31.1%)	54 (33.5%)	
Hypertension/Vascular disease	6 (11.5%)	43 (12.2%)	17 (10.6%)	
Cystic kidney disease	2 (3.9%)	30 (8.5%)	11 (6.8%)	
Glomerulonephritis	12 (23.1%)	87 (24.8%)	36 (22.4%)	
Other/unknown	10 (19.2%)	82 (23.4%)	43 (26.7%)	
**Prior kidney transplant (N (%))**				0.085
No	47 (90.4%)	323 (92.0%)	156 (96.9%)	
Yes	5 (9.6%)	28 (8.0%)	5 (3.1%)	
**Multi-organ transplantation (N (%))**				0.69
Liver + kidney	1 (1.9%)	9 (2.6%)	3 (1.9%)	
Pancreas + kidney	0 (0.0%)	12 (3.4%)	5 (3.1%)	
Heart + kidney	2 (3.8%)	7 (2.0%)	2 (1.2%)	
None	49 (94.2%)	322 (91.7%)	149 (92.5%)	

Liver +Heart+Kidney	0 (0.0%)	1 (0.3%)	2 (1.2%)	
**CMV risk categories (N (%))**				0.049
Low	12 (23.1%)	82 (23.4%)	45 (28.0%)	
Intermediate	23 (44.2%)	166 (47.3%)	64 (39.8%)	
High	12 (23.1%)	95 (27.1%)	49 (30.4%)	
Unknown	5 (9.6%)	8 (2.3%)	3 (1.9%)	
**Was the donor organ pumped? (N (%))**				0.042
No	21 (45.7%)	101 (30.9%)	40 (26.1%)	
Yes	25 (54.3%)	226 (69.1%)	113 (73.9%)	
**En block kidney (N (%))**	0 (0%)	10 (15%)	3 (12%)	0.48
**Dual kidney (N (%))**	1 (12%)	8 (12%)	3 (12%)	1.00
**Transplantation characteristics**				
**Cold ischemic time (hours), median (IQR)**	10.7 (3.2-17.9)	12.5 (4.4-20.0)	13.4 (6.0-18.5)	0.27
**cPRA (%), median (IQR)**	0 (0-17)	0 (0-0)	0 (0-0)	0.061
**Donors' characteristics**				
**Age (years), mean (SD)**	40.3 (14.2)	37.4 (15.7)	35.6 (15.0)	0.14
**Sex (N (%))**				0.14
male	36 (69.2%)	196 (55.8%)	87 (54.0%)	
female	16 (30.8%)	155 (44.2%)	74 (46.0%)	
**Race/ethnicity**				0.36
White	41 (78.8%)	285 (81.2%)	131 (81.4%)	
African American	1 (1.9%)	12 (3.4%)	4 (2.5%)	
Asian	1 (1.9%)	6 (1.7%)	4 (2.5%)	
Other	3 (5.8%)	4 (1.1%)	1 (0.6%)	
Unknown	6 (11.5%)	44 (12.5%)	21 (13.0%)	
**Donor type (N (%))**				0.020
Living	25 (48.1%)	120 (34.2%)	44 (27.3%)	
Deceased	27 (51.9%)	231 (65.8%)	117 (72.7%)	
**Donor DCD (N (%))**				0.80
No	14 (51.9%)	135 (58.4%)	67 (57.3%)	
Yes	13 (48.1%)	96 (41.6%)	50 (42.7%)	
**KDPI, median (IQR)**	26 (12-59)	26 (12-54)	24 (10-46)	0.39
**Donor cause of death (N (%))**				0.38
Anoxia	16 (59.3%)	110 (47.6%)	55 (47.0%)	
Cerebrovascular/stroke	5 (18.5%)	31 (13.4%)	15 (12.8%)	
Head trauma	5 (18.5%)	81 (35.1%)	44 (37.6%)	

Central nervous system tumor	1 (3.7%)	1 (0.4%)	1 (0.9%)	
Other	0 (0.0%)	8 (3.5%)	2 (1.7%)	
**Donor terminal creatinine (mg/dl), median (IQR)**	0.86 (0.66-1.03)	0.81 (0.67-1.02)	0.79 (0.61-0.98)	0.32
**Donor diabetes (N (%))**				0.67
No	51 (98.1%)	331 (95.4%)	153 (95.6%)	
Yes	1 (1.9%)	16 (4.6%)	7 (4.4%)	
**Donor hypertension (N (%))**				0.34
No	48 (92.3%)	292 (84.6%)	135 (85.4%)	
Yes	4 (7.7%)	53 (15.4%)	23 (14.6%)	
**Donor malignancy (N (%))**				0.43
No	52 (100.0%)	343 (97.7%)	156 (96.9%)	
Yes	0 (0.0%)	8 (2.3%)	5 (3.1%)	
**Immunological characteristics**				
**Number of HLA mismatches (HLA A, B and DR) (N (%))**				<0.001
1	10 (19.2%)	5 (1.4%)	0 (0.0%)	
2	11 (21.2%)	23 (6.6%)	1 (0.6%)	
3	22 (42.3%)	73 (20.8%)	8 (5.0%)	
4	9 (17.3%)	102 (29.1%)	27 (16.8%)	
5	0 (0.0%)	101 (28.8%)	74 (46.0%)	
6	0 (0.0%)	47 (13.4%)	51 (31.7%)	
**HLA mismatches A (N (%))**				<0.001
0	15 (100%)	2 (2%)	0 (0%)	
1	0 (0%)	63 (68%)	4 (11%)	
2	0 (0%)	27 (29%)	33 (89%)	
**HLA mismatches B (N (%))**				0.61
0	1 (7%)	5 (5%)	0 (0%)	
1	5 (33%)	28 (30%)	10 (27%)	
2	9 (60%)	59 (64%)	27 (73%)	
**HLA mismatches DR (N (%))**				0.009
0	2 (6.7%)	24 (13.0%)	9 (10.7%)	
1	23 (76.7%)	96 (52.2%)	34 (40.5%)	
2	5 (16.7%)	64 (34.8%)	41 (48.8%)	

**HLA mismatches DQ (N (%))**				0.42
0	2 (13%)	16 (17%)	6 (17%)	
1	9 (60%)	50 (54%)	14 (39%)	
2	4 (27%)	26 (28%)	16 (44%)	
**Current flow B cell (N (%))**				0.19
< 2 SD compatible	26 (83.9%)	176 (93.6%)	80 (93.0%)	
2-3 SD compatible	4 (12.9%)	5 (2.7%)	3 (3.5%)	
weak positive	0 (0.0%)	4 (2.1%)	1 (1.2%)	
positive	1 (3.2%)	3 (1.6%)	2 (2.3%)	
**Current flow T cell (N (%))**				0.41
< 2 SD compatible	27 (87.1%)	173 (92.0%)	83 (96.5%)	
2-3 SD compatible	3 (9.7%)	7 (3.7%)	1 (1.2%)	
weak positive	0 (0.0%)	3 (1.6%)	1 (1.2%)	
positive	1 (3.2%)	5 (2.7%)	1 (1.2%)	
**Induction treatment (N (%))**				
No Induction	0 (0.0%)	0 (0.0%)	0 (0.0%)	NA
Basiliximab	8 (15.4%)	38 (10.8%)	24 (14.9%)	0.34
Thymoglobulin	42 (80.8%)	297 (84.6%)	131 (81.4%)	0.57
Steroid	52 (100.0%)	351 (100.0%)	160 (99.4%)	0.29
Alemtuzumab	1 (1.9%)	9 (2.6%)	4 (2.5%)	0.96
**Outcomes**				
**Proportion of Antibody Mediated Rejection of the subset of patients who underwent biopsy (N (%))**				0.39
No	13 (86.7%)	90 (72.6%)	54 (78.3%)	
Yes	2 (13.3%)	34 (27.4%)	15 (21.7%)	
**Proportion of rejection of the subset of patients who underwent biopsy (N (%))**				0.52
No	10 (66.7%)	70 (56.5%)	44 (63.8%)	
Yes	5 (33.3%)	54 (43.5%)	25 (36.2%)	
**Proportion of T-cell Mediated Rejection of the subset of patients who underwent biopsy (N (%))**				0.76

No	11 (73.3%)	79 (63.7%)	45 (65.2%)	
Yes	4 (26.7%)	45 (36.3%)	24 (34.8%)	
**Post-Transplant de novo Donor Specific Antibodies (N (%))**				0.028
No	47 (94.0%)	276 (79.1%)	124 (77.0%)	
Yes	3 (6.0%)	73 (20.9%)	37 (23.0%)	
**Dd-cf-DNA elevation (N (%))**				0.27
No	37 (88.1%)	217 (79.2%)	104 (76.5%)	
Yes	5 (11.9%)	57 (20.8%)	32 (23.5%)	
**Primary outcome (N (%))**				0.017
No	42 (80.8%)	219 (62.4%)	95 (59.0%)	
Yes	10 (19.2%)	132 (37.6%)	66 (41.0%)	
**Delayed Graft Function (N (%))**				0.59
No	49 (94.2%)	323 (92.0%)	145 (90.1%)	
Yes	3 (5.8%)	28 (8.0%)	16 (9.9%)	
**Death (N (%))**				0.36
No	49 (94.2%)	341 (97.2%)	153 (95.0%)	
Yes	3 (5.8%)	10 (2.8%)	8 (5.0%)	
**Graft Loss (N (%))**				0.53
No	52 (100.0%)	343 (97.7%)	157 (97.5%)	
Yes	0 (0.0%)	8 (2.3%)	4 (2.5%)	

Values are expressed as mean (standard deviation), median (interquartile range), or number (%). Continuous variables were compared via t-tests or Mann-Whitney U tests. Categorical variables were compared via Chi-square test

Table S2: Patient characteristics based on eplet mismatch load for HLA-B locus for primary outcome

	Zero Eplet Mismatch Group	Low Eplet Mismatch Group	High Eplet Mismatch Group	p-value
**N**	25	309	232	
**Recipients' characteristics**				
**Age (years), mean (SD)**	52.7 (14.5)	50.0 (15.3)	49.3 (15.0)	0.55
**Sex (N (%))**				0.30
male	13 (52.0%)	188 (60.8%)	152 (65.5%)	
female	12 (48.0%)	121 (39.2%)	80 (34.5%)	
**Race/ethnicity (N (%))**				0.30
White	22 (88.0%)	203 (68.4%)	155 (69.8%)	
Hispanic	0 (0.0%)	67 (22.6%)	40 (18.0%)	
African American	0 (0.0%)	6 (2.0%)	6 (2.7%)	
Asian	1 (4.0%)	9 (3.0%)	11 (5.0%)	
Other	2 (8.0%)	11 (3.7%)	10 (4.5%)	
Unknown	0 (0.0%)	1 (0.3%)	0 (0.0%)	
**Body Mass Index (kg/m2), mean (SD)**	29.5 (5.8)	28.8 (5.6)	28.8 (5.8)	0.80
**Dialysis vintage (months), median (IQR)**	27.5 (21.0-47.9)	29.9 (14.0-49.0)	33.9 (14.7-55.7)	0.52
**Cause of kidney failure (N (%))**				0.888
Diabetes mellitus	8 (32.0%)	96 (31.1%)	78 (33.6%)	
Hypertension/Vascular disease	2 (8.0%)	39 (12.6%)	27 (11.7%)	
Cystic kidney disease	2 (8.0%)	22 (7.1%)	20 (8.6%)	
Glomerulonephritis	4 (16.0%)	78 (25.2%)	53 (22.8%)	
Other/unknown	9 (36.0%)	74 (24.0%)	54 (23.3%)	
**Prior kidney transplant (N (%))**				0.009
No	20 (80.0%)	285 (92.2%)	222 (95.7%)	
Yes	5 (20.0%)	24 (7.8%)	10 (4.3%)	
**Multi-organ transplantation (N (%))**				0.61
Liver + kidney	0 (0.0%)	7 (2.3%)	7 (3.0%)	
Pancreas + kidney	0 (0.0%)	8 (2.6%)	9 (3.9%)	
Heart + kidney	1 (4.0%)	3 (1.0%)	6 (2.6%)	
None	24 (96.0%)	290 (93.9%)	208 (89.7%)	

Liver +Heart+Kidney	0 (0.0%)	1 (0.3%)	2 (0.9%)	
**CMV risk categories (N (%))**				0.005
Low	5 (20.0%)	84 (27.2%)	51 (22.0%)	
Intermediate	10 (40.0%)	130 (42.1%)	112 (48.3%)	
High	6 (24.0%)	86 (27.8%)	65 (28.0%)	
Unknown	4 (16.0%)	9 (2.9%)	4 (1.7%)	
**Was the donor organ pumped? (N (%))**				0.021
No	12 (52.2%)	93 (32.0%)	55 (25.7%)	
Yes	11 (47.8%)	198 (68.0%)	159 (74.3%)	
**<u>Transplantation characteristics</u>**				
**Cold ischemic time (hours), median (IQR)**	7.6 (3.2-21.7)	12.5 (4.2-19.8)	13.7 (7.2-19.0)	0.34
**cPRA (%), median (IQR)**	0 (0-83)	0 (0-0)	0 (0-0)	<0.001
**<u>Donors' characteristics</u>**				
**Age (years), mean (SD)**	42.2 (10.9)	38.2 (15.6)	35.5 (15.2)	0.034
**Sex (N (%))**				0.29
male	11 (44.0%)	172 (55.7%)	138 (59.5%)	
female	14 (56.0%)	137 (44.3%)	94 (40.5%)	
**Race/ethnicity**				0.037
White	24 (96.0%)	236 (76.4%)	197 (84.9%)	
African American	0 (0.0%)	11 (3.6%)	7 (3.0%)	
Asian	1 (4.0%)	7 (2.3%)	4 (1.7%)	
Other	0 (0.0%)	3 (1.0%)	5 (2.2%)	
Unknown	0 (0.0%)	52 (16.8%)	19 (8.2%)	
**Donor type (N (%))**				0.023
Living	14 (56.0%)	105 (34.0%)	68 (29.3%)	
Deceased	11 (44.0%)	204 (66.0%)	164 (70.7%)	
**Donor DCD (N (%))**				0.57
No	5 (45.5%)	117 (57.4%)	99 (60.4%)	
Yes	6 (54.5%)	87 (42.6%)	65 (39.6%)	
**KDPI, median (IQR)**	26 (14-42)	27 (11-55)	24 (11-50)	0.68
**Donor cause of death (N (%))**				0.92
Anoxia	7 (63.6%)	98 (48.0%)	78 (47.6%)	
Cerebrovascular/stroke	1 (9.1%)	31 (15.2%)	20 (12.2%)	
Head trauma	3 (27.3%)	69 (33.8%)	59 (36.0%)	
Central nervous system tumor	0 (0.0%)	2 (1.0%)	1 (0.6%)	

Other	0 (0.0%)	4 (2.0%)	6 (3.7%)	
**Donor terminal creatinine (mg/dl), median (IQR)**	0.93 (0.72-1.04)	0.805 (0.66-1.0)	0.83 (0.63-1.05)	0.38
**Donor diabetes (N (%))**				0.57
No	24 (96.0%)	292 (95.1%)	222 (96.9%)	
Yes	1 (4.0%)	15 (4.9%)	7 (3.1%)	
**Donor hypertension (N (%))**				0.30
No	24 (96.0%)	258 (84.9%)	193 (84.6%)	
Yes	1 (4.0%)	46 (15.1%)	35 (15.4%)	
**Donor malignancy (N (%))**				0.39
No	24 (96.0%)	300 (97.1%)	229 (98.7%)	
Yes	1 (4.0%)	9 (2.9%)	3 (1.3%)	
**Immunological characteristics**				
**Number of HLA mismatches (HLA A, B and DR) (N (%))**				<0.001
1	8 (32.0%)	6 (1.9%)	0 (0.0%)	
2	7 (28.0%)	24 (7.8%)	4 (1.7%)	
3	5 (20.0%)	77 (24.9%)	17 (7.3%)	
4	3 (12.0%)	78 (25.2%)	56 (24.1%)	
5	2 (8.0%)	81 (26.2%)	100 (43.1%)	
6	0 (0.0%)	43 (13.9%)	55 (23.7%)	
**HLA mismatches A (N (%))**				0.22
0	2 (33%)	9 (13%)	6 (9%)	
1	3 (50%)	35 (49%)	29 (41%)	
2	1 (17%)	27 (38%)	35 (50%)	
**HLA mismatches B (N (%))**				<0.001
0	4 (67%)	2 (3%)	0 (0%)	
1	2 (33%)	35 (49%)	6 (9%)	
2	0 (0%)	34 (48%)	64 (91%)	
**HLA mismatches DR (N (%))**				0.017
0	3 (17.6%)	22 (14.9%)	10 (7.7%)	
1	12 (70.6%)	81 (54.7%)	62 (47.7%)	
2	2 (11.8%)	45 (30.4%)	58 (44.6%)	
**HLA mismatches DQ (N (%))**				0.54

0	0 (0%)	13 (18%)	11 (16%)	
1	5 (83%)	37 (52%)	34 (49%)	
2	1 (17%)	21 (30%)	24 (35%)	
**Current flow B cell (N (%))**				0.69
< 2 SD compatible	15 (88.2%)	138 (91.4%)	126 (93.3%)	
2-3 SD compatible	1 (5.9%)	5 (3.3%)	6 (4.4%)	
weak positive	0 (0.0%)	3 (2.0%)	2 (1.5%)	
positive	1 (5.9%)	5 (3.3%)	1 (0.7%)	
**Current flow T cell (N (%))**				0.47
< 2 SD compatible	15 (88.2%)	138 (91.4%)	128 (94.8%)	
2-3 SD compatible	2 (11.8%)	6 (4.0%)	3 (2.2%)	
weak positive	0 (0.0%)	3 (2.0%)	1 (0.7%)	
positive	0 (0.0%)	4 (2.6%)	3 (2.2%)	
**Induction treatment (N (%))**				
No Induction	0 (0.0%)	0 (0.0%)	0 (0.0%)	NA
Basiliximab	3 (12.0%)	37 (12.0%)	32 (13.8%)	0.82
Thymoglobulin	22 (88.0%)	258 (83.5%)	189 (81.5%)	0.65
Steroid	25 (100.0%)	308 (99.7%)	232 (100.0%)	0.66
Alemtuzumab	0 (0.0%)	8 (2.6%)	7 (3.0%)	0.67
**Outcomes**				
**Proportion of Antibody Mediated Rejection of the subset of patients who underwent biopsy (N (%))**				0.21
No	5 (62.5%)	93 (79.5%)	55 (69.6%)	
Yes	3 (37.5%)	24 (20.5%)	24 (30.4%)	
**Proportion of rejection of the subset of patients who underwent biopsy (N (%))**				0.56
No	4 (50.0%)	73 (62.4%)	44 (55.7%)	
Yes	4 (50.0%)	44 (37.6%)	35 (44.3%)	
**Proportion of T-cell Mediated Rejection of the subset of patients who underwent biopsy (N (%))**				0.49
No	4 (50.0%)	79 (67.5%)	49 (62.0%)	

Yes	4 (50.0%)	38 (32.5%)	30 (38.0%)	
**Post-Transplant de novo Donor Specific Antibodies (N (%))**				0.40
No	21 (84.0%)	250 (81.4%)	178 (77.1%)	
Yes	4 (16.0%)	57 (18.6%)	53 (22.9%)	
**Dd-cf-DNA elevation (N (%))**				0.41
No	16 (88.9%)	192 (77.1%)	151 (80.3%)	
Yes	2 (11.1%)	57 (22.9%)	37 (19.7%)	
**Primary outcome (N (%))**				0.24
No	16 (64.0%)	203 (65.7%)	136 (58.6%)	
Yes	9 (36.0%)	106 (34.3%)	96 (41.4%)	
**Delayed Graft Function (N (%))**				0.22
No	25 (100.0%)	280 (90.6%)	215 (92.7%)	
Yes	0 (0.0%)	29 (9.4%)	17 (7.3%)	
**Death (N (%))**				0.23
No	25 (100.0%)	294 (95.1%)	226 (97.4%)	
Yes	0 (0.0%)	15 (4.9%)	6 (2.6%)	
**Graft Loss (N (%))**				<0.001
No	22 (88.0%)	304 (98.4%)	229 (98.7%)	
Yes	3 (12.0%)	5 (1.6%)	3 (1.3%)	

Values are expressed as mean (standard deviation), median (interquartile range), or number (%). Continuous variables were compared via t-tests or Mann-Whitney U tests. Categorical variables were compared via Chi-square test

Table S3: Patient characteristics based on eplet mismatch load for HLA-C locus for primary outcome

	Zero Eplet Mismatch Group	Low Eplet Mismatch Group	High Eplet Mismatch Group	p-value
**N**	52	304	203	
**Recipients' characteristics**				
**Age (years), mean (SD)**	47.4 (14.9)	50.3 (15.8)	48.9 (14.2)	0.34
**Sex (N (%))**				0.046
male	24 (46.2%)	191 (62.8%)	131 (64.5%)	
female	28 (53.8%)	113 (37.2%)	72 (35.5%)	
**Race/ethnicity (N (%))**				0.40
White	42 (82.4%)	207 (70.4%)	126 (64.6%)	
Hispanic	5 (9.8%)	58 (19.7%)	44 (22.6%)	
African American	1 (2.0%)	6 (2.0%)	5 (2.6%)	
Asian	1 (2.0%)	13 (4.4%)	7 (3.6%)	
Other	2 (3.9%)	9 (3.1%)	13 (6.7%)	
Unknown	0 (0.0%)	1 (0.3%)	0 (0.0%)	
**Body Mass Index (kg/m2), mean (SD)**	28.0 (5.4)	28.6 (5.5)	29.1 (6.2)	0.40
**Dialysis vintage (months), median (IQR)**	30.3 (18.3-48.8)	29.0 (13.3-51.3)	33.4 (13.6-54.3)	0.58
**Cause of kidney failure (N (%))**				0.266
Diabetes mellitus	10 (19.2%)	94 (30.9%)	76 (37.4%)	
Hypertension/Vascular disease	7 (13.5%)	42 (13.9%)	18 (8.9%)	
Cystic kidney disease	3 (5.8%)	24 (7.9%)	15 (7.4%)	
Glomerulonephritis	15 (28.8%)	74 (24.3%)	45 (22.2%)	
Other/unknown	17 (32.7%)	70 (23.0%)	49 (24.1%)	
**Prior kidney transplant (N (%))**				0.001
No	42 (80.8%)	284 (93.4%)	193 (95.1%)	
Yes	10 (19.2%)	20 (6.6%)	10 (4.9%)	
**Multi-organ transplantation (N (%))**				0.29
Liver + kidney	0 (0.0%)	9 (3.0%)	3 (1.5%)	
Pancreas + kidney	1 (1.9%)	7 (2.3%)	8 (3.9%)	
Heart + kidney	1 (1.9%)	5 (1.6%)	5 (2.5%)	
None	50 (96.2%)	283 (93.1%)	184 (90.6%)	

Liver +Heart+Kidney	0 (0.0%)	0 (0.0%)	3 (1.5%)	
**CMV risk categories (N (%))**				0.023
Low	8 (15.4%)	82 (27.0%)	44 (21.7%)	
Intermediate	25 (48.1%)	134 (44.1%)	91 (44.8%)	
High	15 (28.8%)	77 (25.3%)	67 (33.0%)	
Unknown	4 (7.7%)	11 (3.6%)	1 (0.5%)	
**Was the donor organ pumped? (N (%))**				0.091
No	18 (39.1%)	93 (33.0%)	49 (25.4%)	
Yes	28 (60.9%)	189 (67.0%)	144 (74.6%)	
**En block kidney (N (%))**	0 (0%)	9 (17%)	3 (8%)	0.29
**Dual kidney (N (%))**	0 (0%)	6 (11%)	7 (18%)	0.37
**Transplantation characteristics**				
**Cold ischemic time (hours), median (IQR)**	11.1 (3.2-21.4)	12.1 (4.2-19.5)	14.8 (6.6-19.8)	0.17
**cPRA (%), median (IQR)**	0 (0-65)	0 (0-0)	0 (0-0)	0.006
**Donors' characteristics**				
**Age (years), mean (SD)**	38.9 (13.4)	37.7 (15.7)	36.0 (15.7)	0.32
**Sex (N (%))**				0.24
male	25 (48.1%)	171 (56.2%)	123 (60.6%)	
female	27 (51.9%)	133 (43.8%)	80 (39.4%)	
**Race/ethnicity**				0.080
White	43 (82.7%)	253 (83.2%)	154 (75.9%)	
African American	1 (1.9%)	8 (2.6%)	10 (4.9%)	
Asian	1 (1.9%)	6 (2.0%)	4 (2.0%)	
Other	3 (5.8%)	2 (0.7%)	3 (1.5%)	
Unknown	4 (7.7%)	35 (11.5%)	32 (15.8%)	
**Donor type (N (%))**				0.027
Living	23 (44.2%)	109 (35.9%)	55 (27.1%)	
Deceased	29 (55.8%)	195 (64.1%)	148 (72.9%)	
**Donor DCD (N (%))**				0.99
No	17 (58.6%)	115 (59.0%)	86 (58.1%)	
Yes	12 (41.4%)	80 (41.0%)	62 (41.9%)	
**KDPI, median (IQR)**	21 (12-40)	27 (12-53)	24 (10-54)	0.58
**Donor cause of death (N (%))**				0.41
Anoxia	13 (44.8%)	90 (46.2%)	74 (50.0%)	
Cerebrovascular/stroke	4 (13.8%)	31 (15.9%)	17 (11.5%)	
Head trauma	11 (37.9%)	65 (33.3%)	55 (37.2%)	

Central nervous system tumor	1 (3.4%)	2 (1.0%)	0 (0.0%)	
Other	0 (0.0%)	7 (3.6%)	2 (1.4%)	
**Donor terminal creatinine (mg/dl), median (IQR)**	0.84 (0.72-1.02)	0.82 (0.66-1.0)	0.79 (0.61-1.01)	0.30
**Donor diabetes (N (%))**				0.71
No	51 (98.1%)	288 (96.0%)	193 (95.5%)	
Yes	1 (1.9%)	12 (4.0%)	9 (4.5%)	
**Donor hypertension (N (%))**				0.007
No	50 (96.2%)	258 (86.9%)	161 (80.1%)	
Yes	2 (3.8%)	39 (13.1%)	40 (19.9%)	
**Donor malignancy (N (%))**				0.54
No	51 (98.1%)	295 (97.0%)	200 (98.5%)	
Yes	1 (1.9%)	9 (3.0%)	3 (1.5%)	
**<u>Immunological characteristics</u>**				
**Number of HLA mismatches (HLA A, B and DR) (N (%))**				<0.001
1	9 (17.3%)	6 (2.0%)	0 (0.0%)	
2	14 (26.9%)	18 (5.9%)	3 (1.5%)	
3	13 (25.0%)	69 (22.7%)	19 (9.4%)	
4	8 (15.4%)	68 (22.4%)	60 (29.6%)	
5	8 (15.4%)	89 (29.3%)	77 (37.9%)	
6	0 (0.0%)	54 (17.8%)	44 (21.7%)	
**HLA mismatches A (N (%))**				0.059
0	4 (29%)	8 (10%)	5 (10%)	
1	9 (64%)	36 (45%)	21 (44%)	
2	1 (7%)	36 (45%)	22 (46%)	
**HLA mismatches B (N (%))**				<0.001
0	4 (29%)	2 (2%)	0 (0%)	
1	9 (64%)	28 (35%)	6 (12%)	
2	1 (7%)	50 (62%)	42 (88%)	
**HLA mismatches DR (N (%))**				0.16
0	6 (19.4%)	19 (11.5%)	11 (11.3%)	
1	19 (61.3%)	88 (53.3%)	44 (45.4%)	
2	6 (19.4%)	58 (35.2%)	42 (43.3%)	

**HLA mismatches DQ (N (%))**				0.089
0	3 (21%)	13 (16%)	9 (19%)	
1	9 (64%)	46 (57%)	17 (36%)	
2	2 (14%)	21 (26%)	21 (45%)	
**Current flow B cell (N (%))**				0.69
< 2 SD compatible	28 (87.5%)	156 (93.4%)	92 (91.1%)	
2-3 SD compatible	3 (9.4%)	5 (3.0%)	4 (4.0%)	
weak positive	0 (0.0%)	3 (1.8%)	2 (2.0%)	
positive	1 (3.1%)	3 (1.8%)	3 (3.0%)	
**Current flow T cell (N (%))**				0.063
< 2 SD compatible	27 (84.4%)	157 (94.0%)	94 (93.1%)	
2-3 SD compatible	3 (9.4%)	4 (2.4%)	4 (4.0%)	
weak positive	2 (6.2%)	2 (1.2%)	0 (0.0%)	
positive	0 (0.0%)	4 (2.4%)	3 (3.0%)	
**Induction treatment (N (%))**				
No Induction	0 (0.0%)	0 (0.0%)	0 (0.0%)	NA
Basiliximab	6 (11.5%)	46 (15.1%)	19 (9.4%)	0.16
Thymoglobulin	44 (84.6%)	247 (81.2%)	173 (85.2%)	0.48
Steroid	52 (100.0%)	304 (100.0%)	202 (99.5%)	0.42
Alemtuzumab	1 (1.9%)	5 (1.6%)	10 (4.9%)	0.086
**<u>Outcomes</u>**				
**Proportion of Antibody Mediated Rejection of the subset of patients who underwent biopsy (N (%))**				0.83
No	15 (71.4%)	79 (76.0%)	58 (72.5%)	
Yes	6 (28.6%)	25 (24.0%)	22 (27.5%)	
**Proportion of rejection of the subset of patients who underwent biopsy (N (%))**				0.22
No	14 (66.7%)	65 (62.5%)	41 (51.2%)	
Yes	7 (33.3%)	39 (37.5%)	39 (48.8%)	
**Proportion of T-cell Mediated Rejection of the subset of patients who underwent biopsy (N (%))**				0.40

No	14 (66.7%)	71 (68.3%)	47 (58.8%)	
Yes	7 (33.3%)	33 (31.7%)	33 (41.2%)	
**Post-Transplant de novo Donor Specific Antibodies (N (%))**				0.030
No	40 (76.9%)	253 (83.8%)	150 (74.3%)	
Yes	12 (23.1%)	49 (16.2%)	52 (25.7%)	
**Dd-cf-DNA elevation (N (%))**				0.021
No	31 (79.5%)	205 (83.7%)	117 (72.2%)	
Yes	8 (20.5%)	40 (16.3%)	45 (27.8%)	
**Primary outcome (N (%))**				0.003
No	32 (61.5%)	209 (68.8%)	109 (53.7%)	
Yes	20 (38.5%)	95 (31.2%)	94 (46.3%)	
**Delayed Graft Function (N (%))**				0.23
No	50 (96.2%)	282 (92.8%)	182 (89.7%)	
Yes	2 (3.8%)	22 (7.2%)	21 (10.3%)	
**Death (N (%))**				0.32
No	52 (100.0%)	291 (95.7%)	195 (96.1%)	
Yes	0 (0.0%)	13 (4.3%)	8 (3.9%)	
**Graft Loss (N (%))**				0.47
No	50 (96.2%)	299 (98.4%)	197 (97.0%)	
Yes	2 (3.8%)	5 (1.6%)	6 (3.0%)	

Values are expressed as mean (standard deviation), median (interquartile range), or number (%). Continuous variables were compared via t-tests or Mann-Whitney U tests. Categorical variables were compared via Chi-square test

Table S4: Patient characteristics based on eplet mismatch load for HLA-DQA1 locus for primary outcome

	Zero Eplet Mismatch Group	Low Eplet Mismatch Group	High Eplet Mismatch Group	p-value
N	101	173	204	
**<u>Recipients' characteristics</u>**				
**Age (years), mean (SD)**	51.3 (15.5)	49.8 (15.0)	49.0 (15.2)	0.45
**Sex (N (%))**				0.18
male	58 (57.4%)	102 (59.0%)	136 (66.7%)	
female	43 (42.6%)	71 (41.0%)	68 (33.3%)	
**Race/ethnicity (N (%))**				0.004
White	77 (77.8%)	118 (71.1%)	134 (68.4%)	
Hispanic	15 (15.2%)	36 (21.7%)	34 (17.3%)	
African American	4 (4.0%)	3 (1.8%)	4 (2.0%)	
Asian	2 (2.0%)	9 (5.4%)	7 (3.6%)	
Other	1 (1.0%)	0 (0.0%)	16 (8.2%)	
Unknown	0 (0.0%)	0 (0.0%)	1 (0.5%)	
**Body Mass Index (kg/m2), mean (SD)**	28.4 (5.9)	28.7 (5.7)	28.7 (5.4)	0.91
**Dialysis vintage (months), median (IQR)**	23.0 (12.0-42.3)	33.8 (14.2-54.6)	32.9 (14.7-53.7)	0.072
**Cause of kidney failure (N (%))**				0.601
Diabetes mellitus	23 (22.8%)	61 (35.3%)	62 (30.4%)	
Hypertension/Vascular disease	13 (12.9%)	18 (10.4%)	26 (12.7%)	
Cystic kidney disease	10 (9.9%)	15 (8.7%)	14 (6.9%)	
Glomerulonephritis	28 (27.7%)	35 (20.2%)	51 (25.0%)	
Other/unknown	27 (26.7%)	44 (25.4%)	51 (25.0%)	
**Prior kidney transplant (N (%))**				0.002
No	83 (82.2%)	158 (91.3%)	193 (94.6%)	
Yes	18 (17.8%)	15 (8.7%)	11 (5.4%)	
**Multi-organ transplantation (N (%))**				0.20
Liver + kidney	2 (2.0%)	2 (1.2%)	8 (3.9%)	
Pancreas + kidney	0 (0.0%)	9 (5.2%)	7 (3.4%)	
Heart + kidney	1 (1.0%)	3 (1.7%)	6 (2.9%)	
None	98 (97.0%)	158 (91.3%)	181 (88.7%)	

Liver +Heart+Kidney	0 (0.0%)	1 (0.6%)	2 (1.0%)	
**CMV risk categories (N (%))**				0.81
Low	25 (24.8%)	46 (26.6%)	47 (23.0%)	
Intermediate	45 (44.6%)	74 (42.8%)	96 (47.1%)	
High	29 (28.7%)	45 (26.0%)	56 (27.5%)	
Unknown	2 (2.0%)	8 (4.6%)	5 (2.5%)	
**Was the donor organ pumped? (N (%))**				0.45
No	35 (38.0%)	64 (40.8%)	65 (34.2%)	
Yes	57 (62.0%)	93 (59.2%)	125 (65.8%)	
**En block kidney (N (%))**	0 (0%)	4 (16%)	7 (20%)	0.18
**Dual kidney (N (%))**	0 (0%)	4 (16%)	3 (9%)	0.24
**<u>Transplantation characteristics</u>**				
**Cold ischemic time (hours), median (IQR)**	11.6 (3.6-20.4)	10.8 (3.3-18.4)	13.4 (4.3-20.4)	0.23
**cPRA (%), median (IQR)**	0 (0-23)	0 (0-0)	0 (0-0)	0.012
**<u>Donors' characteristics</u>**				
**Age (years), mean (SD)**	36.6 (13.7)	38.3 (15.4)	36.6 (16.1)	0.49
**Sex (N (%))**				0.013
male	43 (42.6%)	105 (60.7%)	114 (55.9%)	
female	58 (57.4%)	68 (39.3%)	90 (44.1%)	
**Race/ethnicity**				0.63
White	81 (80.2%)	138 (79.8%)	152 (74.5%)	
African American	3 (3.0%)	5 (2.9%)	11 (5.4%)	
Asian	2 (2.0%)	1 (0.6%)	6 (2.9%)	
Other	2 (2.0%)	3 (1.7%)	2 (1.0%)	
Unknown	13 (12.9%)	26 (15.0%)	33 (16.2%)	
**Donor type (N (%))**				0.35
Living	42 (41.6%)	76 (43.9%)	75 (36.8%)	
Deceased	59 (58.4%)	97 (56.1%)	129 (63.2%)	
**Donor DCD (N (%))**				0.90
No	36 (61.0%)	58 (59.8%)	81 (62.8%)	
Yes	23 (39.0%)	39 (40.2%)	48 (37.2%)	
**KDPI, median (IQR)**	25 (10-45)	23 (12-53)	22 (9-54)	0.85
**Donor cause of death (N (%))**				0.42
Anoxia	30 (50.8%)	37 (38.1%)	59 (45.7%)	
Cerebrovascular/stroke	11 (18.6%)	17 (17.5%)	14 (10.9%)	
Head trauma	16 (27.1%)	40 (41.2%)	49 (38.0%)	

Central nervous system tumor	1 (1.7%)	0 (0.0%)	2 (1.6%)	
Other	1 (1.7%)	3 (3.1%)	5 (3.9%)	
**Donor terminal creatinine (mg/dl), median (IQR)**	0.79 (0.69-0.96)	0.84 (0.70-1.03)	0.81 (0.62-1.0)	0.46
**Donor diabetes (N (%))**				0.47
No	95 (94.1%)	164 (95.9%)	195 (97.0%)	
Yes	6 (5.9%)	7 (4.1%)	6 (3.0%)	
**Donor hypertension (N (%))**				0.34
No	93 (93.0%)	152 (88.9%)	174 (87.4%)	
Yes	7 (7.0%)	19 (11.1%)	25 (12.6%)	
**Donor malignancy (N (%))**				0.18
No	101 (100.0%)	168 (97.1%)	197 (96.6%)	
Yes	0 (0.0%)	5 (2.9%)	7 (3.4%)	
**<u>Immunological characteristics</u>**				
**Number of HLA mismatches (HLA A, B and DR) (N (%))**				<0.001
1	10 (9.9%)	4 (2.3%)	2 (1.0%)	
2	21 (20.8%)	10 (5.8%)	4 (2.0%)	
3	28 (27.7%)	35 (20.2%)	17 (8.3%)	
4	21 (20.8%)	50 (28.9%)	48 (23.5%)	
5	16 (15.8%)	49 (28.3%)	78 (38.2%)	
6	5 (5.0%)	25 (14.5%)	55 (27.0%)	
**HLA mismatches A (N (%))**				0.39
0	6 (22%)	5 (10%)	4 (7%)	
1	11 (41%)	23 (47%)	27 (49%)	
2	10 (37%)	21 (43%)	24 (44%)	
**HLA mismatches B (N (%))**				0.24
0	0 (0%)	4 (8%)	3 (5%)	
1	9 (33%)	19 (39%)	13 (24%)	
2	18 (67%)	26 (53%)	39 (71%)	
**HLA mismatches DR (N (%))**				<0.001
0	23 (39.0%)	4 (4.0%)	4 (3.4%)	
1	30 (50.8%)	71 (71.7%)	48 (40.7%)	
2	6 (10.2%)	24 (24.2%)	66 (55.9%)	

**HLA mismatches DQ (N (%))**				<0.001
0	14 (52%)	6 (12%)	2 (4%)	
1	12 (44%)	25 (51%)	32 (59%)	
2	1 (4%)	18 (37%)	20 (37%)	
**Current flow B cell (N (%))**				0.98
< 2 SD compatible	54 (91.5%)	94 (94.0%)	117 (92.9%)	
2-3 SD compatible	3 (5.1%)	4 (4.0%)	5 (4.0%)	
weak positive	1 (1.7%)	1 (1.0%)	1 (0.8%)	
positive	1 (1.7%)	1 (1.0%)	3 (2.4%)	
**Current flow T cell (N (%))**				0.66
< 2 SD compatible	53 (89.8%)	96 (96.0%)	117 (92.9%)	
2-3 SD compatible	4 (6.8%)	2 (2.0%)	6 (4.8%)	
weak positive	0 (0.0%)	1 (1.0%)	1 (0.8%)	
positive	2 (3.4%)	1 (1.0%)	2 (1.6%)	
**Induction treatment (N (%))**				
No Induction	0 (0.0%)	0 (0.0%)	0 (0.0%)	NA
Basiliximab	13 (12.9%)	25 (14.5%)	22 (10.8%)	0.56
Thymoglobulin	81 (80.2%)	135 (78.0%)	175 (85.8%)	0.14
Steroid	101 (100.0%)	172 (99.4%)	204 (100.0%)	0.41
Alemtuzumab	3 (3.0%)	11 (6.4%)	3 (1.5%)	0.036
**<u>Outcomes</u>**				
**Proportion of Antibody Mediated Rejection of the subset of patients who underwent biopsy (N (%))**				0.14
No	27 (84%)	36 (67%)	51 (66%)	
Yes	5 (16%)	18 (33%)	26 (34%)	
**Proportion of rejection of the subset of patients who underwent biopsy (N (%))**				0.14
No	23 (72%)	28 (52%)	41 (53%)	
Yes	9 (28%)	26 (48%)	36 (47%)	
**Proportion of T-cell Mediated Rejection of the subset of patients who underwent biopsy (N (%))**				0.17

No	25 (78%)	33 (61%)	46 (60%)	
Yes	7 (22%)	21 (39%)	31 (40%)	
**Post-Transplant de novo Donor Specific Antibodies (N (%))**				0.091
No	88 (87.1%)	141 (81.5%)	155 (76.7%)	
Yes	13 (12.9%)	32 (18.5%)	47 (23.3%)	
**Dd-cf-DNA elevation (N (%))**				0.065
No	69 (87.3%)	110 (80.9%)	121 (74.7%)	
Yes	10 (12.7%)	26 (19.1%)	41 (25.3%)	
**Primary outcome (N (%))**				0.036
No	75 (74.3%)	113 (65.3%)	121 (59.3%)	
Yes	26 (25.7%)	60 (34.7%)	83 (40.7%)	
**Delayed Graft Function (N (%))**				0.12
No	96 (95.0%)	161 (93.1%)	181 (88.7%)	
Yes	5 (5.0%)	12 (6.9%)	23 (11.3%)	
**Death (N (%))**				0.048
No	99 (98.0%)	171 (98.8%)	193 (94.6%)	
Yes	2 (2.0%)	2 (1.2%)	11 (5.4%)	
**Graft Loss (N (%))**				0.38
No	100 (99.0%)	167 (96.5%)	200 (98.0%)	
Yes	1 (1.0%)	6 (3.5%)	4 (2.0%)	

Values are expressed as mean (standard deviation), median (interquartile range), or number (%). Continuous variables were compared via t-tests or Mann-Whitney U tests. Categorical variables were compared via Chi-square test

Table S5: Patient characteristics based on eplet mismatch load for HLA-DQB1 locus for primary outcome

	**Zero Eplet Mismatch Group**	**Low Eplet Mismatch Group**	**High Eplet Mismatch Group**	**p-value**
**N**	86	426	141	
**<u>Recipients' characteristics</u>**				
**Age (years), mean (SD)**	50.6 (16.1)	49.8 (15.0)	50.5 (14.6)	0.82
**Sex (N (%))**				0.44
male	48 (55.8%)	266 (62.4%)	90 (63.8%)	
female	38 (44.2%)	160 (37.6%)	51 (36.2%)	
**Race/ethnicity (N (%))**				0.051
White	67 (77.9%)	287 (71.0%)	82 (59.4%)	
Hispanic	17 (19.8%)	71 (17.6%)	34 (24.6%)	
African American	0 (0.0%)	10 (2.5%)	4 (2.9%)	
Asian	1 (1.2%)	19 (4.7%)	6 (4.3%)	
Other	1 (1.2%)	16 (4.0%)	12 (8.7%)	
Unknown	0 (0.0%)	1 (0.2%)	0 (0.0%)	
**Body Mass Index (kg/m2), mean (SD)**	28.3 (5.6)	28.6 (5.7)	29.5 (5.5)	0.18
**Dialysis vintage (months), median (IQR)**	26.4 (12.1-45.8)	33.8 (15.0-56.2)	35.7 (22.2-59.9)	0.046
**Cause of kidney failure (N (%))**				0.621
Diabetes mellitus	22 (25.6%)	146 (34.3%)	40 (28.4%)	
Hypertension/Vascular disease	13 (15.1%)	49 (11.5%)	21 (14.9%)	
Cystic kidney disease	6 (7.0%)	33 (7.7%)	12 (8.5%)	
Glomerulonephritis	18 (20.9%)	98 (23.0%)	35 (24.8%)	
Other/unknown	27 (31.4%)	100 (23.5%)	33 (23.4%)	
**Prior kidney transplant (N (%))**				<0.001
No	69 (80.2%)	387 (90.8%)	137 (97.2%)	
Yes	17 (19.8%)	39 (9.2%)	4 (2.8%)	
**Multi-organ transplantation (N (%))**				0.74
Liver + kidney	1 (1.2%)	9 (2.1%)	5 (3.5%)	
Pancreas + kidney	1 (1.2%)	15 (3.5%)	2 (1.4%)	
Heart + kidney	1 (1.2%)	8 (1.9%)	2 (1.4%)	
None	83 (96.5%)	392 (92.0%)	131 (92.9%)	

Liver +Heart+Kidney	0 (0.0%)	2 (0.5%)	1 (0.7%)	
**CMV risk categories (N (%))**				0.32
Low	17 (19.8%)	120 (28.2%)	28 (19.9%)	
Intermediate	42 (48.8%)	175 (41.1%)	66 (46.8%)	
High	25 (29.1%)	116 (27.2%)	44 (31.2%)	
Unknown	2 (2.3%)	15 (3.5%)	3 (2.1%)	
**Was the donor organ pumped? (N (%))**				0.028
No	23 (29.5%)	124 (31.0%)	26 (19.1%)	
Yes	55 (70.5%)	276 (69.0%)	110 (80.9%)	
**En block kidney (N (%))**	0 (0%)	10 (14%)	4 (15%)	0.26
**Dual kidney (N (%))**	1 (6%)	9 (13%)	4 (15%)	0.68
**Transplantation characteristics**				
**Cold ischemic time (hours), median (IQR)**	12.6 (4.9-20.4)	12.3 (4.4-19.9)	14.8 (9.2-19.9)	0.12
**cPRA (%), median (IQR)**	0 (0-75)	0 (0-0)	0 (0-0)	<0.001
**Donors' characteristics**				
**Age (years), mean (SD)**	40.1 (14.8)	37.1 (15.7)	36.2 (16.3)	0.18
**Sex (N (%))**				0.22
male	42 (48.8%)	245 (57.5%)	85 (60.3%)	
female	44 (51.2%)	181 (42.5%)	56 (39.7%)	
**Race/ethnicity**				0.22
White	62 (72.1%)	354 (83.1%)	113 (80.1%)	
African American	3 (3.5%)	13 (3.1%)	4 (2.8%)	
Asian	1 (1.2%)	8 (1.9%)	4 (2.8%)	
Other	1 (1.2%)	7 (1.6%)	1 (0.7%)	
Unknown	19 (22.1%)	44 (10.3%)	19 (13.5%)	
**Donor type (N (%))**				0.007
Living	29 (33.7%)	143 (33.6%)	28 (19.9%)	
Deceased	57 (66.3%)	283 (66.4%)	113 (80.1%)	
**Donor DCD (N (%))**				0.070
No	31 (54.4%)	173 (61.1%)	55 (48.7%)	
Yes	26 (45.6%)	110 (38.9%)	58 (51.3%)	
**KDPI, median (IQR)**	32 (16-56)	25 (11-53)	28 (13-52)	0.30
**Donor cause of death (N (%))**				0.62
Anoxia	23 (40.4%)	137 (48.4%)	60 (53.1%)	
Cerebrovascular/stroke	8 (14.0%)	38 (13.4%)	19 (16.8%)	
Head trauma	23 (40.4%)	98 (34.6%)	31 (27.4%)	

Central nervous system tumor	1 (1.8%)	1 (0.4%)	1 (0.9%)	
Other	2 (3.5%)	9 (3.2%)	2 (1.8%)	
**Donor terminal creatinine (mg/dl), median (IQR)**	0.79 (0.69-1.07)	0.81 (0.64-1.0)	0.88 (0.68-1.03)	0.54
**Donor diabetes (N (%))**				0.49
No	80 (93.0%)	403 (96.0%)	133 (95.0%)	
Yes	6 (7.0%)	17 (4.0%)	7 (5.0%)	
**Donor hypertension (N (%))**				0.42
No	75 (88.2%)	357 (85.6%)	115 (82.1%)	
Yes	10 (11.8%)	60 (14.4%)	25 (17.9%)	
**Donor malignancy (N (%))**				0.43
No	83 (96.5%)	418 (98.1%)	136 (96.5%)	
Yes	3 (3.5%)	8 (1.9%)	5 (3.5%)	
**Immunological characteristics**				
**Number of HLA mismatches (HLA A, B and DR) (N (%))**				<0.001
1	12 (14.0%)	5 (1.2%)	0 (0.0%)	
2	14 (16.3%)	23 (5.4%)	3 (2.1%)	
3	33 (38.4%)	75 (17.6%)	14 (9.9%)	
4	16 (18.6%)	112 (26.3%)	32 (22.7%)	
5	10 (11.6%)	147 (34.5%)	55 (39.0%)	
6	1 (1.2%)	64 (15.0%)	37 (26.2%)	
**HLA mismatches A (N (%))**				0.63
0	3 (11.5%)	14 (13.7%)	2 (6.5%)	
1	14 (53.8%)	47 (46.1%)	13 (41.9%)	
2	9 (34.6%)	41 (40.2%)	16 (51.6%)	
**HLA mismatches B (N (%))**				0.59
0	0 (0.0%)	7 (6.9%)	1 (3.2%)	
1	9 (34.6%)	27 (26.5%)	10 (32.3%)	
2	17 (65.4%)	68 (66.7%)	20 (64.5%)	
**HLA mismatches DR (N (%))**				<0.001
0	27 (57.4%)	9 (4.3%)	3 (4.2%)	
1	15 (31.9%)	130 (62.2%)	22 (31.0%)	
2	5 (10.6%)	70 (33.5%)	46 (64.8%)	

**HLA mismatches DQ (N (%))**				<0.001
0	23 (88.5%)	6 (5.9%)	0 (0.0%)	
1	3 (11.5%)	71 (70.3%)	5 (16.1%)	
2	0 (0.0%)	24 (23.8%)	26 (83.9%)	
**Current flow B cell (N (%))**				0.96
< 2 SD compatible	44 (93.6%)	198 (91.2%)	69 (93.2%)	
2-3 SD compatible	1 (2.1%)	11 (5.1%)	2 (2.7%)	
weak positive	1 (2.1%)	3 (1.4%)	1 (1.4%)	
positive	1 (2.1%)	5 (2.3%)	2 (2.7%)	
**Current flow T cell (N (%))**				0.71
< 2 SD compatible	43 (91.5%)	202 (93.1%)	70 (94.6%)	
2-3 SD compatible	3 (6.4%)	7 (3.2%)	2 (2.7%)	
weak positive	1 (2.1%)	3 (1.4%)	0 (0.0%)	
positive	0 (0.0%)	5 (2.3%)	2 (2.7%)	
**Induction treatment (N (%))**				
No Induction	0 (0.0%)	0 (0.0%)	0 (0.0%)	NA
Basiliximab	11 (12.8%)	53 (12.4%)	11 (7.8%)	0.30
Thymoglobulin	69 (80.2%)	351 (82.4%)	128 (90.8%)	0.038
Steroid	86 (100.0%)	426 (100.0%)	140 (99.3%)	0.16
Alemtuzumab	4 (4.7%)	14 (3.3%)	1 (0.7%)	0.17
**<u>Outcomes</u>**				
**Proportion of Antibody Mediated Rejection of the subset of patients who underwent biopsy (N (%))**				0.23
No	26 (86.7%)	108 (73.0%)	41 (70.7%)	
Yes	4 (13.3%)	40 (27.0%)	17 (29.3%)	
**Proportion of rejection of the subset of patients who underwent biopsy (N (%))**				0.028
No	24 (80.0%)	83 (56.1%)	30 (51.7%)	
Yes	6 (20.0%)	65 (43.9%)	28 (48.3%)	
**Proportion of T-cell Mediated Rejection of the subset of patients who underwent biopsy (N (%))**				0.063

No	25 (83.3%)	95 (64.2%)	34 (58.6%)	
Yes	5 (16.7%)	53 (35.8%)	24 (41.4%)	
**Post-Transplant de novo Donor Specific Antibodies (N (%))**				<0.001
No	78 (90.7%)	345 (81.4%)	99 (70.7%)	
Yes	8 (9.3%)	79 (18.6%)	41 (29.3%)	
**Dd-cf-DNA elevation (N (%))**				0.19
No	55 (84.6%)	260 (79.0%)	85 (73.3%)	
Yes	10 (15.4%)	69 (21.0%)	31 (26.7%)	
**Primary outcome (N (%))**				<0.001
No	66 (76.7%)	276 (64.8%)	66 (46.8%)	
Yes	20 (23.3%)	150 (35.2%)	75 (53.2%)	
**Delayed Graft Function (N (%))**				0.84
No	80 (93.0%)	390 (91.5%)	128 (90.8%)	
Yes	6 (7.0%)	36 (8.5%)	13 (9.2%)	
**Death (N (%))**				0.84
No	82 (95.3%)	406 (95.3%)	136 (96.5%)	
Yes	4 (4.7%)	20 (4.7%)	5 (3.5%)	
**Graft Loss (N (%))**				0.66
No	85 (98.8%)	416 (97.7%)	139 (98.6%)	
Yes	1 (1.2%)	10 (2.3%)	2 (1.4%)	

Values are expressed as mean (standard deviation), median (interquartile range), or number (%). Continuous variables were compared via t-tests or Mann-Whitney U tests. Categorical variables were compared via Chi-square test

Table S6: Patient characteristics based on eplet mismatch load for HLA-DRB1 locus for primary outcome

	**Zero Eplet Mismatch Group**	**Low Eplet Mismatch Group**	**High Eplet Mismatch Group**	**p-value**
**N**	40	284	303	
**<u>Recipients' characteristics</u>**				
**Age (years), mean (SD)**	49.0 (16.4)	51.4 (15.0)	49.2 (15.0)	0.18
**Sex (N (%))**				0.46
male	21 (52.5%)	178 (62.7%)	188 (62.0%)	
female	19 (47.5%)	106 (37.3%)	115 (38.0%)	
**Race/ethnicity (N (%))**				0.11
White	32 (80.0%)	183 (66.5%)	205 (71.4%)	
Hispanic	7 (17.5%)	63 (22.9%)	42 (14.6%)	
African American	0 (0.0%)	8 (2.9%)	6 (2.1%)	
Asian	1 (2.5%)	11 (4.0%)	14 (4.9%)	
Other	0 (0.0%)	9 (3.3%)	20 (7.0%)	
Unknown	0 (0.0%)	1 (0.4%)	0 (0.0%)	
**Body Mass Index (kg/m2), mean (SD)**	28.0 (5.9)	28.6 (5.5)	28.9 (5.9)	0.56
**Dialysis vintage (months), median (IQR)**	24.4 (11.9-36.0)	34.7 (16.7-57.1)	34.3 (15.0-56.5)	0.087
**Cause of kidney failure (N (%))**				0.481
Diabetes mellitus	9 (22.5%)	86 (30.3%)	105 (34.6%)	
Hypertension/Vascular disease	5 (12.5%)	41 (14.4%)	34 (11.2%)	
Cystic kidney disease	3 (7.5%)	19 (6.7%)	27 (8.9%)	
Glomerulonephritis	9 (22.5%)	64 (22.5%)	72 (23.8%)	
Other/unknown	14 (35.0%)	74 (26.1%)	65 (21.5%)	
**Prior kidney transplant (N (%))**				<0.001
No	29 (72.5%)	252 (88.7%)	286 (94.4%)	
Yes	11 (27.5%)	32 (11.3%)	17 (5.6%)	
**Multi-organ transplantation (N (%))**				0.14
Liver + kidney	1 (2.5%)	7 (2.5%)	7 (2.3%)	
Pancreas + kidney	0 (0.0%)	2 (0.7%)	15 (5.0%)	
Heart + kidney	1 (2.5%)	4 (1.4%)	6 (2.0%)	
None	38 (95.0%)	270 (95.1%)	273 (90.1%)	

Liver +Heart+Kidney	0 (0.0%)	1 (0.4%)	2 (0.7%)	
**CMV risk categories (N (%))**				0.58
Low	8 (20.0%)	67 (23.6%)	84 (27.7%)	
Intermediate	18 (45.0%)	124 (43.7%)	133 (43.9%)	
High	14 (35.0%)	82 (28.9%)	77 (25.4%)	
Unknown	0 (0.0%)	11 (3.9%)	9 (3.0%)	
**Was the donor organ pumped? (N (%))**				0.98
No	11 (30.6%)	77 (29.1%)	84 (29.3%)	
Yes	25 (69.4%)	188 (70.9%)	203 (70.7%)	
**En block kidney (N (%))**	0 (0%)	7 (14%)	7 (15%)	0.47
**Dual kidney (N (%))**	0 (0%)	5 (10%)	6 (13%)	0.52
**Transplantation characteristics**				
**Cold ischemic time (hours), median (IQR)**	17.1 (4.2-22.2)	13.3 (4.9-19.4)	12.9 (5.3-20.0)	0.57
**cPRA (%), median (IQR)**	0 (0-97)	0 (0-4)	0 (0-0)	0.002
**Donors' characteristics**				
**Age (years), mean (SD)**	36.6 (15.6)	37.2 (15.1)	37.5 (16.3)	0.93
**Sex (N (%))**				0.65
male	20 (50.0%)	162 (57.0%)	175 (57.8%)	
female	20 (50.0%)	122 (43.0%)	128 (42.2%)	
**Race/ethnicity**				0.89
White	33 (82.5%)	232 (81.7%)	240 (79.2%)	
African American	0 (0.0%)	10 (3.5%)	11 (3.6%)	
Asian	0 (0.0%)	6 (2.1%)	7 (2.3%)	
Other	1 (2.5%)	3 (1.1%)	5 (1.7%)	
Unknown	6 (15.0%)	33 (11.6%)	40 (13.2%)	
**Donor type (N (%))**				0.87
Living	14 (35.0%)	91 (32.0%)	94 (31.0%)	
Deceased	26 (65.0%)	193 (68.0%)	209 (69.0%)	
**Donor DCD (N (%))**				0.063
No	18 (69.2%)	100 (51.8%)	129 (61.7%)	
Yes	8 (30.8%)	93 (48.2%)	80 (38.3%)	
**KDPI, median (IQR)**	20 (11-47)	28 (13-52)	28 (12-57)	0.55
**Donor cause of death (N (%))**				0.072
Anoxia	10 (38.5%)	90 (46.6%)	107 (51.2%)	
Cerebrovascular/stroke	7 (26.9%)	26 (13.5%)	26 (12.4%)	
Head trauma	8 (30.8%)	68 (35.2%)	71 (34.0%)	

Central nervous system tumor	1 (3.8%)	0 (0.0%)	2 (1.0%)	
Other	0 (0.0%)	9 (4.7%)	3 (1.4%)	
**Donor terminal creatinine (mg/dl), median (IQR)**	0.8 (0.68-1.05)	0.84 (0.65-1.03)	0.82 (0.66-1.03)	0.99
**Donor diabetes (N (%))**				0.35
No	40 (100.0%)	268 (95.4%)	284 (95.0%)	
Yes	0 (0.0%)	13 (4.6%)	15 (5.0%)	
**Donor hypertension (N (%))**				0.44
No	35 (89.7%)	245 (87.2%)	249 (84.1%)	
Yes	4 (10.3%)	36 (12.8%)	47 (15.9%)	
**Donor malignancy (N (%))**				0.28
No	40 (100.0%)	279 (98.2%)	293 (96.7%)	
Yes	0 (0.0%)	5 (1.8%)	10 (3.3%)	
**<u>Immunological characteristics</u>**				
**Number of HLA mismatches (HLA A, B and DR) (N (%))**				<0.001
1	10 (25.0%)	6 (2.1%)	1 (0.3%)	
2	11 (27.5%)	25 (8.8%)	4 (1.3%)	
3	10 (25.0%)	79 (27.8%)	23 (7.6%)	
4	7 (17.5%)	78 (27.5%)	70 (23.1%)	
5	2 (5.0%)	77 (27.1%)	122 (40.3%)	
6	0 (0.0%)	19 (6.7%)	83 (27.4%)	
**HLA mismatches A (N (%))**				0.29
0	2 (17%)	11 (14%)	6 (9%)	
1	4 (33%)	42 (53%)	27 (42%)	
2	6 (50%)	26 (33%)	32 (49%)	
**HLA mismatches B (N (%))**				0.23
0	0 (0%)	5 (6%)	2 (3%)	
1	3 (25%)	28 (35%)	14 (22%)	
2	9 (75%)	46 (58%)	49 (75%)	
**HLA mismatches DR (N (%))**				<0.001
0	17 (81.0%)	20 (12.9%)	1 (0.7%)	
1	3 (14.3%)	112 (72.3%)	51 (35.2%)	
2	1 (4.8%)	23 (14.8%)	93 (64.1%)	

**HLA mismatches DQ (N (%))**				<0.001
0	10 (83%)	16 (20%)	3 (5%)	
1	1 (8%)	49 (62%)	29 (45%)	
2	1 (8%)	14 (18%)	32 (50%)	
**Current flow B cell (N (%))**				0.71
< 2 SD compatible	20 (90.9%)	143 (90.5%)	142 (93.4%)	
2-3 SD compatible	1 (4.5%)	8 (5.1%)	6 (3.9%)	
weak positive	1 (4.5%)	2 (1.3%)	1 (0.7%)	
positive	0 (0.0%)	5 (3.2%)	3 (2.0%)	
**Current flow T cell (N (%))**				0.23
< 2 SD compatible	20 (90.9%)	147 (93.0%)	142 (93.4%)	
2-3 SD compatible	2 (9.1%)	5 (3.2%)	5 (3.3%)	
weak positive	0 (0.0%)	0 (0.0%)	3 (2.0%)	
positive	0 (0.0%)	6 (3.8%)	2 (1.3%)	
**Induction treatment (N (%))**				
No Induction	0 (0.0%)	0 (0.0%)	0 (0.0%)	NA
Basiliximab	2 (5.0%)	45 (15.8%)	27 (8.9%)	0.013
Thymoglobulin	34 (85.0%)	226 (79.6%)	263 (86.8%)	0.061
Steroid	40 (100.0%)	284 (100.0%)	302 (99.7%)	0.59
Alemtuzumab	2 (5.0%)	7 (2.5%)	10 (3.3%)	0.63
**Outcomes**				
**Proportion of Antibody Mediated Rejection of the subset of patients who underwent biopsy (N (%))**				0.044
No	11 (84.6%)	77 (81.1%)	79 (66.9%)	
Yes	2 (15.4%)	18 (18.9%)	39 (33.1%)	
**Proportion of rejection of the subset of patients who underwent biopsy (N (%))**				0.061
No	11 (84.6%)	58 (61.1%)	62 (52.5%)	
Yes	2 (15.4%)	37 (38.9%)	56 (47.5%)	
**Proportion of T-cell Mediated Rejection of the subset of patients who underwent biopsy (N (%))**				0.10

No	12 (92.3%)	61 (64.2%)	74 (62.7%)	
Yes	1 (7.7%)	34 (35.8%)	44 (37.3%)	
**Post-Transplant de novo Donor Specific Antibodies (N (%))**				0.020
No	36 (90.0%)	235 (83.3%)	229 (75.8%)	
Yes	4 (10.0%)	47 (16.7%)	73 (24.2%)	
**Dd-cf-DNA elevation (N (%))**				0.002
No	26 (92.9%)	182 (83.1%)	173 (71.8%)	
Yes	2 (7.1%)	37 (16.9%)	68 (28.2%)	
**Primary outcome (N (%))**				0.003
No	30 (75.0%)	192 (67.6%)	169 (55.8%)	
Yes	10 (25.0%)	92 (32.4%)	134 (44.2%)	
**Delayed Graft Function (N (%))**				0.27
No	38 (95.0%)	265 (93.3%)	273 (90.1%)	
Yes	2 (5.0%)	19 (6.7%)	30 (9.9%)	
**Death (N (%))**				0.034
No	40 (100.0%)	265 (93.3%)	294 (97.0%)	
Yes	0 (0.0%)	19 (6.7%)	9 (3.0%)	
**Graft Loss (N (%))**				0.26
No	40 (100.0%)	280 (98.6%)	294 (97.0%)	
Yes	0 (0.0%)	4 (1.4%)	9 (3.0%)	

Values are expressed as mean (standard deviation), median (interquartile range), or number (%). Continuous variables were compared via t-tests or Mann-Whitney U tests. Categorical variables were compared via Chi-square test

Table S7. Performance characteristics table of eplet mismatch load for HLA-A locus for primary outcome

Cutpoint	Sensitivity	Specificity	Overall % correctly classified	LR+	LR-
(>= 1)	100.00%	0.00%	38.67%	1	
(>= 2)	98.99%	0.96%	38.87%	0.9994	1.0572
(>= 3)	97.47%	1.59%	38.67%	0.9905	1.5859
(>= 4)	95.96%	3.18%	39.06%	0.9912	1.2687
(>= 5)	94.95%	5.73%	40.23%	1.0072	0.881
(>= 6)	91.92%	8.28%	40.62%	1.0022	0.9759
(>= 7)	85.86%	11.15%	40.04%	0.9663	1.2687
(>= 8)	80.30%	15.61%	40.63%	0.9515	1.2622
(>= 9)	75.76%	20.70%	41.99%	0.9553	1.1711
(>= 10)	70.71%	29.62%	45.51%	1.0046	0.989
(>= 11)	65.66%	35.03%	46.88%	1.0106	0.9803
(>= 12)	61.62%	38.54%	47.46%	1.0025	0.9961
(>= 13)	55.05%	43.63%	48.05%	0.9766	1.0302
(>= 14)	51.52%	49.04%	50.00%	1.011	0.9886
(>= 15)	45.96%	53.18%	50.39%	0.9817	1.0161
(>= 16)	39.39%	57.96%	50.78%	0.9371	1.0456
(>= 17)	35.35%	65.29%	53.71%	1.0184	0.9902
(>= 18)	33.33%	69.75%	55.66%	1.1018	0.9559
(>= 19)	30.30%	72.29%	56.05%	1.0937	0.9641
(>= 20)	28.28%	76.11%	57.62%	1.1841	0.9422
(>= 21)	24.75%	79.62%	58.40%	1.2142	0.9452
(>= 22)	22.73%	81.21%	58.59%	1.2096	0.9515
(>= 23)	19.19%	83.76%	58.79%	1.1816	0.9648
(>= 24)	17.17%	83.76%	58.01%	1.0572	0.9889
(>= 25)	14.65%	85.99%	58.40%	1.0452	0.9926
(>= 26)	13.64%	87.90%	59.18%	1.1268	0.9825
(>= 27)	12.63%	90.45%	60.35%	1.3215	0.966
(>= 28)	11.11%	91.40%	60.35%	1.2922	0.9725
(>= 29)	10.10%	92.68%	60.74%	1.379	0.97
(>= 30)	8.59%	93.63%	60.74%	1.348	0.9763
(>= 31)	6.57%	93.95%	60.16%	1.0851	0.9945
(>= 32)	5.56%	95.22%	60.55%	1.163	0.9918
(>= 33)	5.56%	96.18%	61.13%	1.4537	0.982
(>= 34)	4.04%	96.82%	60.94%	1.2687	0.9912

(>= 35)	2.53%	98.09%	61.13%	1.3215	0.9937
(>= 36)	1.52%	98.41%	60.94%	0.9515	1.0008
(>= 37)	1.52%	98.73%	61.13%	1.1894	0.9976
(>= 38)	1.01%	99.36%	61.33%	1.5859	0.9962
(>= 45)	1.01%	100.00%	61.72%	0.9899	
(>= 48)	0.51%	100.00%	61.52%	0.9949	
(> 48)	0.00%	100.00%	61.33%	1	

Table S8. Performance characteristics table of eplet mismatch load for HLA-B locus for primary outcome

Cutpoint	Sensitivity	Specificity	Overall % correctly classified	LR+	LR-
(>= 1)	100.00%	0.00%	37.34%	1	
(>= 2)	98.51%	0.29%	36.97%	0.9881	5.0347
(>= 3)	98.02%	1.77%	37.71%	0.9979	1.1188
(>= 4)	96.53%	3.24%	38.08%	0.9977	1.068
(>= 5)	94.55%	5.31%	38.63%	0.9986	1.0256
(>= 6)	92.57%	10.32%	41.04%	1.0323	0.7192
(>= 7)	91.09%	13.27%	42.33%	1.0503	0.6713
(>= 8)	87.62%	17.70%	43.81%	1.0647	0.6993
(>= 9)	84.16%	24.78%	46.95%	1.1188	0.6393
(>= 10)	80.69%	28.91%	48.24%	1.1351	0.6679
(>= 11)	75.74%	32.74%	48.80%	1.1262	0.7408
(>= 12)	70.30%	38.64%	50.46%	1.1457	0.7686
(>= 13)	61.88%	43.36%	50.28%	1.0926	0.8791
(>= 14)	57.43%	48.97%	52.13%	1.1253	0.8694
(>= 15)	51.98%	53.98%	53.23%	1.1296	0.8895
(>= 16)	47.52%	59.88%	55.27%	1.1846	0.8763
(>= 17)	40.59%	64.90%	55.82%	1.1564	0.9154
(>= 18)	37.62%	69.62%	57.67%	1.2383	0.896
(>= 19)	31.19%	74.34%	58.23%	1.2153	0.9257
(>= 20)	25.74%	77.88%	58.41%	1.1636	0.9535
(>= 21)	23.76%	81.71%	60.07%	1.2993	0.933
(>= 22)	18.81%	84.37%	59.89%	1.2033	0.9623
(>= 23)	15.84%	85.84%	59.70%	1.1188	0.9804
(>= 24)	11.88%	88.20%	59.70%	1.0069	0.9991
(>= 25)	10.89%	89.68%	60.26%	1.0549	0.9937
(>= 26)	8.91%	91.74%	60.81%	1.0789	0.9929
(>= 27)	7.43%	92.63%	60.81%	1.0069	0.9994
(>= 28)	6.44%	94.99%	61.92%	1.2833	0.985
(>= 29)	4.95%	95.28%	61.55%	1.0489	0.9976
(>= 30)	4.46%	96.76%	62.29%	1.3731	0.9875
(>= 31)	3.47%	97.35%	62.29%	1.3053	0.9917
(>= 32)	2.48%	97.94%	62.29%	1.1987	0.9958
(>= 33)	1.98%	98.23%	62.29%	1.1188	0.9979
(>= 34)	1.49%	98.53%	62.29%	1.0069	0.9999

(>= 35)	1.49%	98.82%	62.48%	1.2587	0.9969
(>= 36)	0.50%	99.12%	62.29%	0.5594	1.0039
(>= 38)	0.00%	99.41%	62.29%	0	1.0059
(>= 43)	0.00%	99.71%	62.48%	0	1.003
(> 43)	0.00%	100.00%	62.66%	1	

LR: Likelihood ratio

Table S9: Performance characteristics table of eplet mismatch load for HLA-C locus for primary outcome

Cutpoint	Sensitivity	Specificity	Overall % correctly classified	LR+	LR-
(>= 1)	100.00%	0.00%	37.28%	1	
(>= 2)	98.94%	4.72%	39.84%	1.0384	0.2243
(>= 3)	96.83%	8.18%	41.22%	1.0545	0.3883
(>= 4)	91.53%	12.58%	42.01%	1.047	0.673
(>= 5)	86.24%	19.18%	44.18%	1.0671	0.7171
(>= 6)	80.95%	25.47%	46.15%	1.0862	0.7478
(>= 7)	74.07%	31.76%	47.53%	1.0855	0.8163
(>= 8)	66.14%	41.19%	50.49%	1.1247	0.822
(>= 9)	60.32%	49.37%	53.45%	1.1914	0.8038
(>= 10)	55.56%	58.49%	57.40%	1.3384	0.7599
(>= 11)	49.74%	65.72%	59.76%	1.451	0.7648
(>= 12)	39.15%	69.18%	57.99%	1.2705	0.8795
(>= 13)	31.22%	74.21%	58.19%	1.2106	0.9268
(>= 14)	27.51%	78.62%	59.57%	1.2866	0.922
(>= 15)	22.22%	81.13%	59.17%	1.1778	0.9587
(>= 16)	20.11%	84.28%	60.36%	1.2787	0.948
(>= 17)	15.87%	86.16%	59.96%	1.1472	0.9764
(>= 18)	13.23%	89.62%	61.14%	1.2747	0.9682
(>= 19)	11.64%	92.45%	62.33%	1.5423	0.9557
(>= 20)	10.05%	94.65%	63.12%	1.8805	0.9503
(>= 21)	6.88%	95.60%	62.52%	1.5624	0.9741
(>= 22)	4.76%	97.48%	62.92%	1.8929	0.977
(>= 23)	3.70%	98.11%	62.92%	1.963	0.9815
(>= 24)	2.65%	99.06%	63.12%	2.8042	0.9828
(>= 25)	1.59%	99.06%	62.72%	1.6825	0.9935
(>= 27)	1.06%	99.06%	62.52%	1.1217	0.9988
(>= 28)	0.53%	99.37%	62.52%	0.8413	1.001
(>= 29)	0.53%	99.69%	62.72%	1.6825	0.9978
(> 29)	0.00%	100.00%	62.72%	1	

LR: Likelihood ratio

Table S10: Performance characteristics table of eplet mismatch load for HLA-DQA1 locus for primary outcome

Cutpoint	Sensitivity	Specificity	Overall % correctly classified	LR+	LR-
(>= 1)	100.00%	0.00%	37.93%	1	
(>= 2)	92.31%	8.55%	40.32%	1.0093	0.9
(>= 3)	77.62%	26.07%	45.62%	1.0499	0.8584
(>= 4)	58.04%	48.29%	51.99%	1.1225	0.8689
(>= 5)	42.66%	61.11%	54.11%	1.0969	0.9383
(>= 6)	31.47%	71.79%	56.50%	1.1157	0.9545
(>= 7)	22.38%	78.63%	57.29%	1.0473	0.9872
(>= 8)	11.89%	85.47%	57.56%	0.8182	1.0309
(>= 9)	10.49%	88.89%	59.15%	0.9441	1.007
(>= 10)	7.69%	91.88%	59.95%	0.9474	1.0047
(>= 11)	6.29%	94.87%	61.27%	1.2273	0.9877
(>= 12)	5.59%	95.73%	61.54%	1.3091	0.9862
(>= 13)	3.50%	96.58%	61.27%	1.0227	0.9992
(>= 14)	1.40%	97.86%	61.27%	0.6545	1.0075
(>= 15)	0.70%	99.15%	61.80%	0.8182	1.0016
(> 15)	0.00%	100.00%	62.07%	1	

LR: Likelihood ratio

Table S11: Performance characteristics table of eplet mismatch load for HLA-DQB1 locus for primary outcome

Cutpoint	Sensitivity	Specificity	Overall % correctly classified	LR+	LR-
(>= 1)	100.00%	0.00%	39.68%	1	
(>= 2)	97.33%	6.73%	42.68%	1.0435	0.3965
(>= 3)	95.11%	10.82%	44.27%	1.0665	0.4519
(>= 4)	90.67%	15.50%	45.33%	1.0729	0.6023
(>= 5)	85.78%	19.59%	45.86%	1.0668	0.726
(>= 6)	79.56%	24.85%	46.56%	1.0587	0.8226
(>= 7)	69.33%	34.50%	48.32%	1.0586	0.8888
(>= 8)	65.78%	43.27%	52.20%	1.1596	0.7908
(>= 9)	60.00%	50.29%	54.14%	1.2071	0.7953
(>= 10)	53.33%	53.80%	53.62%	1.1544	0.8674
(>= 11)	46.22%	63.74%	56.79%	1.2748	0.8437
(>= 12)	41.78%	70.76%	59.26%	1.4288	0.8228
(>= 13)	38.67%	74.27%	60.14%	1.5027	0.8258
(>= 14)	33.33%	80.70%	61.90%	1.7273	0.8261
(>= 15)	27.56%	84.50%	61.90%	1.7781	0.8573
(>= 16)	24.00%	87.13%	62.08%	1.8655	0.8722
(>= 17)	20.44%	88.89%	61.73%	1.84	0.895
(>= 18)	15.56%	90.35%	60.67%	1.6121	0.9346
(>= 19)	13.33%	92.40%	61.02%	1.7538	0.938
(>= 20)	12.00%	92.98%	60.85%	1.71	0.9464
(>= 21)	11.56%	95.03%	61.90%	2.3247	0.9307
(>= 22)	9.33%	95.91%	61.55%	2.28	0.9454
(>= 23)	7.11%	97.37%	61.55%	2.7022	0.954
(>= 24)	5.78%	97.66%	61.20%	2.47	0.9648
(>= 25)	4.44%	97.95%	60.85%	2.1714	0.9755
(>= 26)	4.00%	97.95%	60.67%	1.9543	0.9801
(>= 27)	3.56%	97.95%	60.49%	1.7371	0.9846
(>= 28)	2.67%	98.25%	60.32%	1.52	0.9907
(>= 29)	1.78%	98.83%	60.32%	1.52	0.9938
(>= 30)	1.78%	99.12%	60.49%	2.0267	0.9909
(>= 31)	0.00%	99.42%	59.96%	0	1.0059
(>= 32)	0.00%	99.71%	60.14%	0	1.0029
(> 32)	0.00%	100.00%	60.32%	1	

LR: Likelihood ratio

Table S12: Performance characteristics table of eplet mismatch load for HLA-DRB1 locus for primary outcome

Cutpoint	Sensitivity	Specificity	Overall % correctly classified	LR+	LR-
(>= 1)	100.00%	0.00%	38.50%	1	
(>= 2)	98.67%	2.77%	39.69%	1.0148	0.4792
(>= 3)	97.35%	5.82%	41.06%	1.0336	0.4564
(>= 4)	95.58%	6.37%	40.72%	1.0208	0.6945
(>= 5)	95.13%	10.25%	42.93%	1.06	0.4749
(>= 6)	95.13%	11.08%	43.44%	1.0699	0.4393
(>= 7)	91.59%	13.85%	43.78%	1.0632	0.607
(>= 8)	89.82%	18.84%	46.17%	1.1067	0.5403
(>= 9)	86.73%	21.61%	46.68%	1.1063	0.6144
(>= 10)	81.42%	26.32%	47.53%	1.1049	0.7062
(>= 11)	75.22%	32.41%	48.89%	1.1129	0.7645
(>= 12)	69.91%	39.34%	51.11%	1.1524	0.7649
(>= 13)	66.81%	45.15%	53.49%	1.2182	0.735
(>= 14)	62.39%	48.75%	54.00%	1.2174	0.7714
(>= 15)	59.29%	53.19%	55.54%	1.2665	0.7654
(>= 16)	51.77%	56.79%	54.86%	1.198	0.8493
(>= 17)	47.35%	62.60%	56.73%	1.266	0.8411
(>= 18)	43.36%	64.54%	56.39%	1.223	0.8775
(>= 19)	37.17%	70.08%	57.41%	1.2424	0.8965
(>= 20)	34.07%	73.13%	58.09%	1.268	0.9015
(>= 21)	30.53%	75.90%	58.43%	1.2669	0.9153
(>= 22)	26.11%	77.84%	57.92%	1.178	0.9493
(>= 23)	25.22%	80.61%	59.28%	1.3007	0.9277
(>= 24)	22.57%	84.21%	60.48%	1.4292	0.9195
(>= 25)	21.68%	86.43%	61.50%	1.5973	0.9062
(>= 26)	19.03%	88.37%	61.67%	1.6354	0.9163
(>= 27)	17.26%	90.30%	62.18%	1.7799	0.9163
(>= 28)	15.49%	91.41%	62.18%	1.8035	0.9245
(>= 29)	11.95%	92.24%	61.33%	1.5403	0.9546
(>= 30)	9.73%	93.07%	60.99%	1.4057	0.9698
(>= 31)	7.96%	93.91%	60.82%	1.3069	0.9801
(>= 32)	7.52%	94.74%	61.16%	1.4292	0.9762
(>= 33)	7.08%	95.57%	61.50%	1.5973	0.9723
(>= 34)	6.19%	96.12%	61.50%	1.5973	0.9759

(>= 35)	6.19%	96.68%	61.84%	1.8636	0.9703
(>= 36)	5.31%	96.95%	61.67%	1.7426	0.9767
(>= 37)	4.87%	96.95%	61.50%	1.5973	0.9812
(>= 38)	3.10%	97.23%	60.99%	1.1181	0.9966
(>= 39)	3.10%	97.51%	61.16%	1.2424	0.9938
(>= 40)	3.10%	98.06%	61.50%	1.5973	0.9882
(>= 41)	3.10%	98.61%	61.84%	2.2363	0.9826
(>= 42)	2.65%	98.89%	61.84%	2.396	0.9844
(>= 43)	1.77%	99.17%	61.67%	2.1298	0.9905
(>= 44)	1.77%	99.45%	61.84%	3.1947	0.9878
(>= 47)	1.77%	99.72%	62.01%	6.3894	0.985
(>= 48)	1.33%	99.72%	61.84%	4.792	0.9895
(>= 52)	0.88%	100.00%	61.84%	0.9912	
(>= 55)	0.44%	100.00%	61.67%	0.9956	
(> 55)	0.00%	100.00%	61.50%	1	

LR: Likelihood ratio

Table S13. Eplet mismatch load, concordant vs. discordant patients, by locus

Locus	N (Disc./Conc.)	Mean eplet MM (Disc. vs Conc.)	t-test p	Rank-sum p	Adjusted OR (95% CI)	Adjusted p
HLA-A	41 / 55	16.10 vs 15.40	0.741	0.493	1.00 (0.94–1.05)	0.873
HLA-B	41 / 55	16.39 vs 13.35	0.047	0.139	0.93 (0.87–1.00)	0.061
HLA-DRB1	42 / 60	19.57 vs 17.92	0.371	0.481	0.98 (0.92–1.03)	0.398
HLA-C[a]	41 / 54	10.51 vs 9.76	0.584	0.985	1.00 (0.93–1.07)	0.939
HLA-DQA1[ab]	28 / 45	5.11 vs 4.13	0.221	0.250	0.92 (0.79–1.07)	0.275
HLA-DQB1[a]	44 / 62	11.89 vs 9.45	0.078	0.114	0.96 (0.90–1.01)	0.144

[a] *Reduced covariate set (age at transplant, donor type, cPRA) used for the adjusted model given smaller available N*

[b] *HLA-DQA1 antigen-level typing is not routinely performed at high resolution in this cohort; this does not affect the eplet-only concordant/discordant comparison shown here, but interpret cautiously given the small N.*

Table S14: Patient characteristics of the de novo DSA cohort based on eplet mismatch load for HLA-A locus

	Zero Eplet Mismatch Group	Low Eplet Mismatch Group	High Eplet Mismatch Group	p-value
**N**	50	379	131	
**<u>Recipients' characteristics</u>**				
**Age (years), mean (SD)**	56.4 (14.4)	49.6 (15.2)	48.5 (14.8)	0.005
**Sex (N (%))**				0.48
male	33 (66.0%)	229 (60.4%)	86 (65.6%)	
female	17 (34.0%)	150 (39.6%)	45 (34.4%)	
**Race/ethnicity (N (%))**				0.12
White	42 (87.5%)	256 (69.8%)	78 (63.4%)	
Hispanic	5 (10.4%)	75 (20.4%)	26 (21.1%)	
African American	0 (0.0%)	9 (2.5%)	2 (1.6%)	
Asian	1 (2.1%)	13 (3.5%)	7 (5.7%)	
Other	0 (0.0%)	13 (3.5%)	10 (8.1%)	
Unknown	0 (0.0%)	1 (0.3%)	0 (0.0%)	
**Body Mass Index (kg/m2), mean (SD)**	28.7 (5.2)	28.7 (5.9)	29.3 (5.6)	0.51
**Dialysis vintage (months), median (IQR)**	27.1 (14.4-43.6)	29.7 (13.8-48.9)	35.4 (14.7-56.2)	0.390
**Cause of kidney failure (N (%))**				0.646
Diabetes mellitus	22 (44.0%)	119 (31.4%)	44 (33.6%)	
Hypertension/Vascular disease	5 (10.0%)	45 (11.9%)	15 (11.5%)	
Cystic kidney disease	2 (4.0%)	31 (8.2%)	10 (7.6%)	
Glomerulonephritis	12 (24.0%)	96 (25.3%)	26 (19.8%)	
Other/unknown	9 (18.0%)	88 (23.2%)	36 (27.5%)	
**Prior kidney transplant (N (%))**				0.13
No	45 (90.0%)	350 (92.3%)	127 (96.9%)	
Yes	5 (10.0%)	29 (7.7%)	4 (3.1%)	
**Multi-organ transplantation (N (%))**				0.47
Liver + kidney	1 (2.0%)	10 (2.6%)	2 (1.5%)	
Pancreas + kidney	0 (0.0%)	12 (3.2%)	5 (3.8%)	
Heart + kidney	2 (4.0%)	8 (2.1%)	1 (0.8%)	
None	47 (94.0%)	348 (91.8%)	121 (92.4%)	

Liver +Heart+Kidney	0 (0.0%)	1 (0.3%)	2 (1.5%)	
**CMV risk categories (N (%))**				0.089
Low	10 (20.0%)	93 (24.5%)	34 (26.0%)	
Intermediate	23 (46.0%)	172 (45.4%)	57 (43.5%)	
High	12 (24.0%)	105 (27.7%)	38 (29.0%)	
Unknown	5 (10.0%)	9 (2.4%)	2 (1.5%)	
**Was the donor organ pumped? (N (%))**				0.046
No	21 (46.7%)	106 (30.0%)	34 (27.2%)	
Yes	24 (53.3%)	247 (70.0%)	91 (72.8%)	
**En block kidney (N (%))**	0 (0%)	11 (15%)	2 (11%)	0.48
**Dual kidney (N (%))**	1 (14%)	8 (11%)	3 (16%)	0.85
**Transplantation characteristics**				
**Cold ischemic time (hours), median (IQR)**	9.6 (3.1-17.8)	12.6 (4.6-20.1)	13.4 (5.9-18.4)	0.23
**cPRA (%), median (IQR)**	0 (0-8)	0 (0-0)	0 (0-0)	0.21
**Donors' characteristics**				
**Age (years), mean (SD)**	40.9 (14.2)	37.4 (15.9)	35.3 (14.5)	0.088
**Sex (N (%))**				0.073
male	35 (70.0%)	215 (56.7%)	67 (51.1%)	
female	15 (30.0%)	164 (43.3%)	64 (48.9%)	
**Race/ethnicity**				0.39
White	39 (78.0%)	308 (81.3%)	106 (80.9%)	
African American	1 (2.0%)	12 (3.2%)	4 (3.1%)	
Asian	1 (2.0%)	7 (1.8%)	3 (2.3%)	
Other	3 (6.0%)	4 (1.1%)	1 (0.8%)	
Unknown	6 (12.0%)	48 (12.7%)	17 (13.0%)	
**Donor type (N (%))**				0.042
Living	24 (48.0%)	126 (33.2%)	37 (28.2%)	
Deceased	26 (52.0%)	253 (66.8%)	94 (71.8%)	
**Donor DCD (N (%))**				0.92
No	14 (53.8%)	147 (58.1%)	54 (57.4%)	
Yes	12 (46.2%)	106 (41.9%)	40 (42.6%)	
**KDPI, median (IQR)**	27 (12-59)	28 (12-54)	23 (9-43)	0.12
**Donor cause of death (N (%))**				0.33
Anoxia	15 (57.7%)	121 (47.8%)	43 (45.7%)	
Cerebrovascular/stroke	5 (19.2%)	36 (14.2%)	10 (10.6%)	
Head trauma	5 (19.2%)	87 (34.4%)	38 (40.4%)	

Central nervous system tumor	1 (3.8%)	1 (0.4%)	1 (1.1%)	
Other	0 (0.0%)	8 (3.2%)	2 (2.1%)	
**Donor terminal creatinine (mg/dl), median (IQR)**	0.85 (0.66-1.03)	0.81 (0.65-1.02)	0.79 (0.61-0.97)	0.40
**Donor diabetes (N (%))**				0.44
No	49 (98.0%)	356 (94.9%)	126 (96.9%)	
Yes	1 (2.0%)	19 (5.1%)	4 (3.1%)	
**Donor hypertension (N (%))**				0.17
No	46 (92.0%)	312 (83.6%)	113 (88.3%)	
Yes	4 (8.0%)	61 (16.4%)	15 (11.7%)	
**Donor malignancy (N (%))**				0.28
No	50 (100.0%)	371 (97.9%)	126 (96.2%)	
Yes	0 (0.0%)	8 (2.1%)	5 (3.8%)	
**<u>Immunological characteristics</u>**				
**Number of HLA mismatches (HLA A, B and DR) (N (%))**				<0.001
1	10 (20.0%)	5 (1.3%)	0 (0.0%)	
2	11 (22.0%)	23 (6.1%)	1 (0.8%)	
3	21 (42.0%)	75 (19.8%)	6 (4.6%)	
4	8 (16.0%)	109 (28.8%)	19 (14.5%)	
5	0 (0.0%)	110 (29.0%)	64 (48.9%)	
6	0 (0.0%)	57 (15.0%)	41 (31.3%)	
**HLA mismatches A (N (%))**				<0.001
0	13 (100.0%)	2 (2.0%)	0 (0.0%)	
1	0 (0.0%)	67 (66.3%)	0 (0.0%)	
2	0 (0.0%)	32 (31.7%)	28 (100.0%)	
**HLA mismatches B (N (%))**				0.57
0	1 (7.7%)	5 (5.0%)	0 (0.0%)	
1	5 (38.5%)	31 (30.7%)	7 (25.0%)	
2	7 (53.8%)	65 (64.4%)	21 (75.0%)	
**HLA mismatches DR (N (%))**				0.008
0	2 (7.1%)	27 (13.4%)	6 (9.2%)	
1	23 (82.1%)	101 (50.2%)	29 (44.6%)	
2	3 (10.7%)	73 (36.3%)	30 (46.2%)	

**HLA mismatches DQ (N (%))**				0.13
0	2 (15.4%)	19 (19.0%)	3 (10.7%)	
1	9 (69.2%)	53 (53.0%)	11 (39.3%)	
2	2 (15.4%)	28 (28.0%)	14 (50.0%)	
**Current flow B cell (N (%))**				0.15
< 2 SD compatible	24 (82.8%)	193 (94.1%)	62 (92.5%)	
2-3 SD compatible	4 (13.8%)	6 (2.9%)	2 (3.0%)	
weak positive	0 (0.0%)	3 (1.5%)	1 (1.5%)	
positive	1 (3.4%)	3 (1.5%)	2 (3.0%)	
**Current flow T cell (N (%))**				0.33
< 2 SD compatible	25 (86.2%)	189 (92.2%)	65 (97.0%)	
2-3 SD compatible	3 (10.3%)	7 (3.4%)	1 (1.5%)	
weak positive	0 (0.0%)	4 (2.0%)	0 (0.0%)	
positive	1 (3.4%)	5 (2.4%)	1 (1.5%)	
**Induction treatment (N (%))**				
No Induction	0 (0.0%)	0 (0.0%)	0 (0.0%)	NA
Basiliximab	8 (16.0%)	43 (11.3%)	19 (14.5%)	0.47
Thymoglobulin	40 (80.0%)	318 (83.9%)	108 (82.4%)	0.76
Steroid	50 (100.0%)	379 (100.0%)	130 (99.2%)	0.19
Alemtuzumab	1 (2.0%)	10 (2.6%)	3 (2.3%)	0.95
**Outcomes**				
**Proportion of Antibody Mediated Rejection of the subset of patients who underwent biopsy (N (%))**				0.42
No	13 (86.7%)	97 (72.9%)	47 (78.3%)	
Yes	2 (13.3%)	36 (27.1%)	13 (21.7%)	
**Proportion of rejection of the subset of patients who underwent biopsy (N (%))**				0.61
No	10 (66.7%)	76 (57.1%)	38 (63.3%)	
Yes	5 (33.3%)	57 (42.9%)	22 (36.7%)	
**Proportion of T-cell Mediated Rejection of the subset of patients who underwent biopsy (N (%))**				0.77

No	11 (73.3%)	85 (63.9%)	39 (65.0%)	
Yes	4 (26.7%)	48 (36.1%)	21 (35.0%)	
**Post-Transplant de novo Donor Specific Antibodies (N (%))**				0.016
No	47 (94.0%)	302 (79.7%)	98 (74.8%)	
Yes	3 (6.0%)	77 (20.3%)	33 (25.2%)	
**Dd-cf-DNA elevation (N (%))**				0.33
No	35 (87.5%)	235 (78.9%)	84 (76.4%)	
Yes	5 (12.5%)	63 (21.1%)	26 (23.6%)	
**Delayed Graft Function (N (%))**				0.79
No	47 (94.0%)	347 (91.6%)	119 (90.8%)	
Yes	3 (6.0%)	32 (8.4%)	12 (9.2%)	
**Death (N (%))**				0.13
No	47 (94.0%)	369 (97.4%)	123 (93.9%)	
Yes	3 (6.0%)	10 (2.6%)	8 (6.1%)	
**Graft Loss (N (%))**				0.45
No	50 (100.0%)	371 (97.9%)	127 (96.9%)	
Yes	0 (0.0%)	8 (2.1%)	4 (3.1%)	

Values are expressed as mean (standard deviation), median (interquartile range), or number (%). Continuous variables were compared via t-tests or Mann-Whitney U tests. Categorical variables were compared via Chi-square tests.

Table S15: Patient characteristics of the de novo DSA cohort based on eplet mismatch load for HLA-B locus

	Zero Eplet Mismatch Group	Low Eplet Mismatch Group	High Eplet Mismatch Group	p-value
**N**	25	307	231	
**<u>Recipients' characteristics</u>**				
**Age (years), mean (SD)**	52.7 (14.5)	50.0 (15.3)	49.4 (15.0)	0.56
**Sex (N (%))**				0.32
male	13 (52.0%)	187 (60.9%)	151 (65.4%)	
female	12 (48.0%)	120 (39.1%)	80 (34.6%)	
**Race/ethnicity (N (%))**				0.28
White	22 (88.0%)	201 (68.1%)	155 (70.1%)	
Hispanic	0 (0.0%)	67 (22.7%)	39 (17.6%)	
African American	0 (0.0%)	6 (2.0%)	6 (2.7%)	
Asian	1 (4.0%)	9 (3.1%)	11 (5.0%)	
Other	2 (8.0%)	11 (3.7%)	10 (4.5%)	
Unknown	0 (0.0%)	1 (0.3%)	0 (0.0%)	
**Body Mass Index (kg/m2), mean (SD)**	29.5 (5.8)	28.8 (5.6)	28.8 (5.8)	0.81
**Dialysis vintage (months), median (IQR)**	27.5 (21.0-47.9)	29.9 (14.1-48.4)	33.8 (14.7-55.7)	0.535
**Cause of kidney failure (N (%))**				0.874
Diabetes mellitus	8 (32.0%)	96 (31.3%)	78 (33.7%)	
Hypertension/Vascular disease	2 (8.0%)	39 (12.7%)	26 (11.3%)	
Cystic kidney disease	2 (8.0%)	22 (7.2%)	20 (8.7%)	
Glomerulonephritis	4 (16.0%)	78 (25.4%)	53 (22.9%)	
Other/unknown	9 (36.0%)	72 (23.4%)	54 (23.4%)	
**Prior kidney transplant (N (%))**				0.009
No	20 (80.0%)	283 (92.2%)	221 (95.7%)	
Yes	5 (20.0%)	24 (7.8%)	10 (4.3%)	
**Multi-organ transplantation (N (%))**				0.61
Liver + kidney	0 (0.0%)	7 (2.3%)	7 (3.0%)	
Pancreas + kidney	0 (0.0%)	8 (2.6%)	9 (3.9%)	
Heart + kidney	1 (4.0%)	3 (1.0%)	6 (2.6%)	
None	24 (96.0%)	288 (93.8%)	207 (89.6%)	

Liver +Heart+Kidney	0 (0.0%)	1 (0.3%)	2 (0.9%)	
**CMV risk categories (N (%))**				0.005
Low	5 (20.0%)	83 (27.0%)	50 (21.6%)	
Intermediate	10 (40.0%)	129 (42.0%)	112 (48.5%)	
High	6 (24.0%)	86 (28.0%)	65 (28.1%)	
Unknown	4 (16.0%)	9 (2.9%)	4 (1.7%)	
**Was the donor organ pumped? (N (%))**				0.022
No	12 (52.2%)	93 (32.1%)	55 (25.8%)	
Yes	11 (47.8%)	197 (67.9%)	158 (74.2%)	
**Transplantation characteristics**				
**Cold ischemic time (hours), median (IQR)**	7.6 (3.2-21.7)	12.1 (4.2-19.7)	13.7 (7.2-19.1)	0.30
**cPRA (%), median (IQR)**	0 (0-83)	0 (0-0)	0 (0-0)	<0.001
**Donors' characteristics**				
**Age (years), mean (SD)**	42.2 (10.9)	38.3 (15.6)	35.6 (15.3)	0.036
**Sex (N (%))**				0.30
male	11 (44.0%)	171 (55.7%)	137 (59.3%)	
female	14 (56.0%)	136 (44.3%)	94 (40.7%)	
**Race/ethnicity**				0.036
White	24 (96.0%)	234 (76.2%)	196 (84.8%)	
African American	0 (0.0%)	11 (3.6%)	7 (3.0%)	
Asian	1 (4.0%)	7 (2.3%)	4 (1.7%)	
Other	0 (0.0%)	3 (1.0%)	5 (2.2%)	
Unknown	0 (0.0%)	52 (16.9%)	19 (8.2%)	
**Donor type (N (%))**				0.025
Living	14 (56.0%)	104 (33.9%)	68 (29.4%)	
Deceased	11 (44.0%)	203 (66.1%)	163 (70.6%)	
**Donor DCD (N (%))**				0.53
No	5 (45.5%)	116 (57.1%)	99 (60.7%)	
Yes	6 (54.5%)	87 (42.9%)	64 (39.3%)	
**KDPI, median (IQR)**	26 (14-42)	27 (11-56)	24 (11-51)	0.70
**Donor cause of death (N (%))**				0.92
Anoxia	7 (63.6%)	97 (47.8%)	77 (47.2%)	
Cerebrovascular/stroke	1 (9.1%)	31 (15.3%)	20 (12.3%)	
Head trauma	3 (27.3%)	69 (34.0%)	59 (36.2%)	
Central nervous system tumor	0 (0.0%)	2 (1.0%)	1 (0.6%)	

Other	0 (0.0%)	4 (2.0%)	6 (3.7%)	
**Donor terminal creatinine (mg/dl), median (IQR)**	0.93 (0.72-1.04)	0.81 (0.66-1.0)	0.83 (0.63-1.05)	0.39
**Donor diabetes (N (%))**				0.57
No	24 (96.0%)	290 (95.1%)	221 (96.9%)	
Yes	1 (4.0%)	15 (4.9%)	7 (3.1%)	
**Donor hypertension (N (%))**				0.30
No	24 (96.0%)	256 (84.8%)	192 (84.6%)	
Yes	1 (4.0%)	46 (15.2%)	35 (15.4%)	
**Donor malignancy (N (%))**				0.39
No	24 (96.0%)	298 (97.1%)	228 (98.7%)	
Yes	1 (4.0%)	9 (2.9%)	3 (1.3%)	
**Immunological characteristics**				
**Number of HLA mismatches (HLA A, B and DR) (N (%))**				<0.001
1	8 (32.0%)	6 (2.0%)	0 (0.0%)	
2	7 (28.0%)	24 (7.8%)	4 (1.7%)	
3	5 (20.0%)	77 (25.1%)	16 (6.9%)	
4	3 (12.0%)	77 (25.1%)	56 (24.2%)	
5	2 (8.0%)	80 (26.1%)	100 (43.3%)	
6	0 (0.0%)	43 (14.0%)	55 (23.8%)	
**HLA mismatches A (N (%))**				0.16
0	2 (33%)	8 (11%)	5 (7%)	
1	3 (50%)	35 (50%)	29 (42%)	
2	1 (17%)	27 (39%)	35 (51%)	
**HLA mismatches B (N (%))**				<0.001
0	4 (67%)	2 (3%)	0 (0%)	
1	2 (33%)	35 (50%)	6 (9%)	
2	0 (0%)	33 (47%)	63 (91%)	
**HLA mismatches DR (N (%))**				0.015
0	3 (17.6%)	22 (15.1%)	10 (7.8%)	
1	12 (70.6%)	81 (55.5%)	62 (48.1%)	
2	2 (11.8%)	43 (29.5%)	57 (44.2%)	
**HLA mismatches DQ (N (%))**				0.55

0	0 (0%)	13 (19%)	11 (16%)	
1	5 (83%)	37 (53%)	34 (50%)	
2	1 (17%)	20 (29%)	23 (34%)	
**Current flow B cell (N (%))**				0.72
< 2 SD compatible	15 (88.2%)	137 (91.9%)	125 (93.3%)	
2-3 SD compatible	1 (5.9%)	5 (3.4%)	6 (4.5%)	
weak positive	0 (0.0%)	2 (1.3%)	2 (1.5%)	
positive	1 (5.9%)	5 (3.4%)	1 (0.7%)	
**Current flow T cell (N (%))**				0.48
< 2 SD compatible	15 (88.2%)	136 (91.3%)	127 (94.8%)	
2-3 SD compatible	2 (11.8%)	6 (4.0%)	3 (2.2%)	
weak positive	0 (0.0%)	3 (2.0%)	1 (0.7%)	
positive	0 (0.0%)	4 (2.7%)	3 (2.2%)	
**Induction treatment (N (%))**				
No Induction	0 (0.0%)	0 (0.0%)	0 (0.0%)	NA
Basiliximab	3 (12.0%)	37 (12.1%)	32 (13.9%)	0.82
Thymoglobulin	22 (88.0%)	256 (83.4%)	188 (81.4%)	0.65
Steroid	25 (100.0%)	306 (99.7%)	231 (100.0%)	0.66
Alemtuzumab	0 (0.0%)	8 (2.6%)	7 (3.0%)	0.67
**<u>Outcomes</u>**				
**Proportion of Antibody Mediated Rejection of the subset of patients who underwent biopsy (N (%))**				0.21
No	5 (62.5%)	93 (79.5%)	55 (69.6%)	
Yes	3 (37.5%)	24 (20.5%)	24 (30.4%)	
**Proportion of rejection of the subset of patients who underwent biopsy (N (%))**				0.56
No	4 (50.0%)	73 (62.4%)	44 (55.7%)	
Yes	4 (50.0%)	44 (37.6%)	35 (44.3%)	
**Proportion of T-cell Mediated Rejection of the subset of patients who underwent biopsy (N (%))**				0.49
No	4 (50.0%)	79 (67.5%)	49 (62.0%)	

Yes	4 (50.0%)	38 (32.5%)	30 (38.0%)	
**Post-Transplant de novo Donor Specific Antibodies (N (%))**				0.40
No	21 (84.0%)	250 (81.4%)	178 (77.1%)	
Yes	4 (16.0%)	57 (18.6%)	53 (22.9%)	
**Dd-cf-DNA elevation (N (%))**				0.40
No	16 (88.9%)	190 (76.9%)	150 (80.2%)	
Yes	2 (11.1%)	57 (23.1%)	37 (19.8%)	
**Delayed Graft Function (N (%))**				0.21
No	25 (100.0%)	278 (90.6%)	214 (92.6%)	
Yes	0 (0.0%)	29 (9.4%)	17 (7.4%)	
**Death (N (%))**				0.23
No	25 (100.0%)	292 (95.1%)	225 (97.4%)	
Yes	0 (0.0%)	15 (4.9%)	6 (2.6%)	
**Graft Loss (N (%))**				<0.001
No	22 (88.0%)	302 (98.4%)	228 (98.7%)	
Yes	3 (12.0%)	5 (1.6%)	3 (1.3%)	

Values are expressed as mean (standard deviation), median (interquartile range), or number (%). Continuous variables were compared via t-tests or Mann-Whitney U tests. Categorical variables were compared via Chi-square tests.

Table S16: Patient characteristics for the de novo DSA cohort based on eplet mismatch load for HLA-C locus

	**Zero Eplet Mismatch Group**	**Low Eplet Mismatch Group**	**High Eplet Mismatch Group**	**p-value**
**N**	52	302	202	
**Recipients' characteristics**				
**Age (years), mean (SD)**	47.4 (14.9)	50.4 (15.9)	48.9 (14.2)	0.33
**Sex (N (%))**				0.047
male	24 (46.2%)	190 (62.9%)	130 (64.4%)	
female	28 (53.8%)	112 (37.1%)	72 (35.6%)	
**Race/ethnicity (N (%))**				0.43
White	42 (82.4%)	205 (70.2%)	126 (64.9%)	
Hispanic	5 (9.8%)	58 (19.9%)	43 (22.2%)	
African American	1 (2.0%)	6 (2.1%)	5 (2.6%)	
Asian	1 (2.0%)	13 (4.5%)	7 (3.6%)	
Other	2 (3.9%)	9 (3.1%)	13 (6.7%)	
Unknown	0 (0.0%)	1 (0.3%)	0 (0.0%)	
**Body Mass Index (kg/m2), mean (SD)**	28.0 (5.4)	28.6 (5.5)	29.1 (6.1)	0.37
**Dialysis vintage (months), median (IQR)**	30.3 (18.3-48.9)	29.0 (13.5-51.1)	33.1 (13.6-54.3)	0.58
**Cause of kidney failure (N (%))**				0.212
Diabetes mellitus	10 (19.2%)	94 (31.1%)	76 (37.6%)	
Hypertension/Vascular disease	7 (13.5%)	42 (13.9%)	17 (8.4%)	
Cystic kidney disease	3 (5.8%)	24 (8.0%)	15 (7.4%)	
Glomerulonephritis	15 (28.8%)	74 (24.5%)	45 (22.3%)	
Other/unknown	17 (32.7%)	68 (22.5%)	49 (24.3%)	
**Prior kidney transplant (N (%))**				0.002
No	42 (80.8%)	282 (93.4%)	192 (95.0%)	
Yes	10 (19.2%)	20 (6.6%)	10 (5.0%)	
**Multi-organ transplantation (N (%))**				0.29
Liver + kidney	0 (0.0%)	9 (3.0%)	3 (1.5%)	
Pancreas + kidney	1 (1.9%)	7 (2.3%)	8 (4.0%)	
Heart + kidney	1 (1.9%)	5 (1.7%)	5 (2.5%)	
None	50 (96.2%)	281 (93.0%)	183 (90.6%)	

Liver +Heart+Kidney	0 (0.0%)	0 (0.0%)	3 (1.5%)	
**CMV risk categories (N (%))**				0.023
Low	8 (15.4%)	81 (26.8%)	43 (21.3%)	
Intermediate	25 (48.1%)	133 (44.0%)	91 (45.0%)	
High	15 (28.8%)	77 (25.5%)	67 (33.2%)	
Unknown	4 (7.7%)	11 (3.6%)	1 (0.5%)	
**Was the donor organ pumped? (N (%))**				0.095
No	18 (39.1%)	93 (33.1%)	49 (25.5%)	
Yes	28 (60.9%)	188 (66.9%)	143 (74.5%)	
**En block kidney (N (%))**	0 (0%)	9 (17%)	3 (8%)	0.30
**Dual kidney (N (%))**	0 (0%)	6 (11%)	7 (19%)	0.34
**Transplantation characteristics**				
**Cold ischemic time (hours), median (IQR)**	11.1 (3.2-21.4)	12.0 (4.2-19.2)	14.9 (6.6-19.8)	0.13
**cPRA (%), median (IQR)**	0 (0-65)	0 (0-0)	0 (0-0)	0.005
**Donors' characteristics**				
**Age (years), mean (SD)**	38.9 (13.4)	37.8 (15.7)	36.0 (15.7)	0.34
**Sex (N (%))**				0.26
male	25 (48.1%)	170 (56.3%)	122 (60.4%)	
female	27 (51.9%)	132 (43.7%)	80 (39.6%)	
**Race/ethnicity**				0.081
White	43 (82.7%)	251 (83.1%)	153 (75.7%)	
African American	1 (1.9%)	8 (2.6%)	10 (5.0%)	
Asian	1 (1.9%)	6 (2.0%)	4 (2.0%)	
Other	3 (5.8%)	2 (0.7%)	3 (1.5%)	
Unknown	4 (7.7%)	35 (11.6%)	32 (15.8%)	
**Donor type (N (%))**				0.031
Living	23 (44.2%)	108 (35.8%)	55 (27.2%)	
Deceased	29 (55.8%)	194 (64.2%)	147 (72.8%)	
**Donor DCD (N (%))**				1.00
No	17 (58.6%)	114 (58.8%)	86 (58.5%)	
Yes	12 (41.4%)	80 (41.2%)	61 (41.5%)	
**KDPI, median (IQR)**	21 (12-40)	27 (12-53)	24 (10-54)	0.59
**Donor cause of death (N (%))**				0.42
Anoxia	13 (44.8%)	89 (45.9%)	73 (49.7%)	
Cerebrovascular/stroke	4 (13.8%)	31 (16.0%)	17 (11.6%)	
Head trauma	11 (37.9%)	65 (33.5%)	55 (37.4%)	

Central nervous system tumor	1 (3.4%)	2 (1.0%)	0 (0.0%)	
Other	0 (0.0%)	7 (3.6%)	2 (1.4%)	
**Donor terminal creatinine (mg/dl), median (IQR)**	0.84 (0.72-1.02)	0.82 (0.65-1.0)	0.79 (0.61-1.02)	0.28
**Donor diabetes (N (%))**				0.70
No	51 (98.1%)	286 (96.0%)	192 (95.5%)	
Yes	1 (1.9%)	12 (4.0%)	9 (4.5%)	
**Donor hypertension (N (%))**				0.007
No	50 (96.2%)	256 (86.8%)	160 (80.0%)	
Yes	2 (3.8%)	39 (13.2%)	40 (20.0%)	
**Donor malignancy (N (%))**				0.54
No	51 (98.1%)	293 (97.0%)	199 (98.5%)	
Yes	1 (1.9%)	9 (3.0%)	3 (1.5%)	
**Immunological characteristics**				
**Number of HLA mismatches (HLA A, B and DR) (N (%))**				<0.001
1	9 (17.3%)	6 (2.0%)	0 (0.0%)	
2	14 (26.9%)	18 (6.0%)	3 (1.5%)	
3	13 (25.0%)	69 (22.8%)	18 (8.9%)	
4	8 (15.4%)	67 (22.2%)	60 (29.7%)	
5	8 (15.4%)	88 (29.1%)	77 (38.1%)	
6	0 (0.0%)	54 (17.9%)	44 (21.8%)	
**HLA mismatches A (N (%))**				0.038
0	4 (29%)	7 (9%)	4 (9%)	
1	9 (64%)	36 (46%)	21 (45%)	
2	1 (7%)	36 (46%)	22 (47%)	
**HLA mismatches B (N (%))**				<0.001
0	4 (29%)	2 (3%)	0 (0%)	
1	9 (64%)	28 (35%)	6 (13%)	
2	1 (7%)	49 (62%)	41 (87%)	
**HLA mismatches DR (N (%))**				0.17
0	6 (19.4%)	19 (11.7%)	11 (11.5%)	
1	19 (61.3%)	88 (54.0%)	44 (45.8%)	
2	6 (19.4%)	56 (34.4%)	41 (42.7%)	

**HLA mismatches DQ (N (%))**				0.10
0	3 (21%)	13 (16%)	9 (20%)	
1	9 (64%)	46 (58%)	17 (37%)	
2	2 (14%)	20 (25%)	20 (43%)	
**Current flow B cell (N (%))**				0.67
< 2 SD compatible	28 (87.5%)	155 (93.9%)	91 (91.0%)	
2-3 SD compatible	3 (9.4%)	5 (3.0%)	4 (4.0%)	
weak positive	0 (0.0%)	2 (1.2%)	2 (2.0%)	
positive	1 (3.1%)	3 (1.8%)	3 (3.0%)	
**Current flow T cell (N (%))**				0.066
< 2 SD compatible	27 (84.4%)	155 (93.9%)	93 (93.0%)	
2-3 SD compatible	3 (9.4%)	4 (2.4%)	4 (4.0%)	
weak positive	2 (6.2%)	2 (1.2%)	0 (0.0%)	
positive	0 (0.0%)	4 (2.4%)	3 (3.0%)	
**Induction treatment (N (%))**				
No Induction	0 (0.0%)	0 (0.0%)	0 (0.0%)	NA
Basiliximab	6 (11.5%)	46 (15.2%)	19 (9.4%)	0.15
Thymoglobulin	44 (84.6%)	245 (81.1%)	172 (85.1%)	0.47
Steroid	52 (100.0%)	302 (100.0%)	201 (99.5%)	0.42
Alemtuzumab	1 (1.9%)	5 (1.7%)	10 (5.0%)	0.087
**<u>Outcomes</u>**				
**Proportion of Antibody Mediated Rejection of the subset of patients who underwent biopsy (N (%))**				0.83
No	15 (71.4%)	79 (76.0%)	58 (72.5%)	
Yes	6 (28.6%)	25 (24.0%)	22 (27.5%)	
**Proportion of rejection of the subset of patients who underwent biopsy (N (%))**				0.22
No	14 (66.7%)	65 (62.5%)	41 (51.2%)	
Yes	7 (33.3%)	39 (37.5%)	39 (48.8%)	
**Proportion of T-cell Mediated Rejection of the subset of patients who underwent biopsy (N (%))**				0.40

No	14 (66.7%)	71 (68.3%)	47 (58.8%)	
Yes	7 (33.3%)	33 (31.7%)	33 (41.2%)	
**Post-Transplant de novo Donor Specific Antibodies (N (%))**				0.030
No	40 (76.9%)	253 (83.8%)	150 (74.3%)	
Yes	12 (23.1%)	49 (16.2%)	52 (25.7%)	
**Dd-cf-DNA elevation (N (%))**				0.021
No	31 (79.5%)	203 (83.5%)	116 (72.0%)	
Yes	8 (20.5%)	40 (16.5%)	45 (28.0%)	
**Delayed Graft Function (N (%))**				0.23
No	50 (96.2%)	280 (92.7%)	181 (89.6%)	
Yes	2 (3.8%)	22 (7.3%)	21 (10.4%)	
**Death (N (%))**				0.32
No	52 (100.0%)	289 (95.7%)	194 (96.0%)	
Yes	0 (0.0%)	13 (4.3%)	8 (4.0%)	
**Graft Loss (N (%))**				0.48
No	50 (96.2%)	297 (98.3%)	196 (97.0%)	
Yes	2 (3.8%)	5 (1.7%)	6 (3.0%)	

Values are expressed as mean (standard deviation), median (interquartile range), or number (%). Continuous variables were compared via t-tests or Mann-Whitney U tests. Categorical variables were compared via Chi-square tests.

Table S17: Patient characteristics for the de novo DSA cohort based on eplet mismatch load for HLA-DQA1 locus

	Zero Eplet Mismatch Group	Low Eplet Mismatch Group	High Eplet Mismatch Group	p-value
**N**	101	173	202	
**Recipients' characteristics**				
**Age (years), mean (SD)**	51.3 (15.5)	49.8 (15.0)	49.1 (15.2)	0.49
**Sex (N (%))**				0.17
male	58 (57.4%)	102 (59.0%)	135 (66.8%)	
female	43 (42.6%)	71 (41.0%)	67 (33.2%)	
**Race/ethnicity (N (%))**				0.004
White	77 (77.8%)	118 (71.1%)	132 (68.0%)	
Hispanic	15 (15.2%)	36 (21.7%)	34 (17.5%)	
African American	4 (4.0%)	3 (1.8%)	4 (2.1%)	
Asian	2 (2.0%)	9 (5.4%)	7 (3.6%)	
Other	1 (1.0%)	0 (0.0%)	16 (8.2%)	
Unknown	0 (0.0%)	0 (0.0%)	1 (0.5%)	
**Body Mass Index (kg/m2), mean (SD)**	28.4 (5.9)	28.7 (5.7)	28.8 (5.4)	0.89
**Dialysis vintage (months), median (IQR)**	23.0 (12.0-42.3)	33.8 (14.2-54.6)	33.2 (14.7-54.3)	0.066
**Cause of kidney failure (N (%))**				0.61
Diabetes mellitus	23 (22.8%)	61 (35.3%)	62 (30.7%)	
Hypertension/Vascular disease	13 (12.9%)	18 (10.4%)	26 (12.8%)	
Cystic kidney disease	10 (9.9%)	15 (8.7%)	14 (6.9%)	
Glomerulonephritis	28 (27.7%)	35 (20.2%)	50 (24.8%)	
Other/unknown	27 (26.7%)	44 (25.4%)	50 (24.8%)	
**Prior kidney transplant (N (%))**				0.002
No	83 (82.2%)	158 (91.3%)	191 (94.6%)	
Yes	18 (17.8%)	15 (8.7%)	11 (5.4%)	
**Multi-organ transplantation (N (%))**				0.19
Liver + kidney	2 (2.0%)	2 (1.2%)	8 (4.0%)	
Pancreas + kidney	0 (0.0%)	9 (5.2%)	7 (3.5%)	
Heart + kidney	1 (1.0%)	3 (1.7%)	6 (3.0%)	
None	98 (97.0%)	158 (91.3%)	179 (88.6%)	

Liver +Heart+Kidney	0 (0.0%)	1 (0.6%)	2 (1.0%)	
**CMV risk categories (N (%))**				0.79
Low	25 (24.8%)	46 (26.6%)	46 (22.8%)	
Intermediate	45 (44.6%)	74 (42.8%)	96 (47.5%)	
High	29 (28.7%)	45 (26.0%)	55 (27.2%)	
Unknown	2 (2.0%)	8 (4.6%)	5 (2.5%)	
**Was the donor organ pumped? (N (%))**				0.41
No	35 (38.0%)	64 (40.8%)	64 (33.9%)	
Yes	57 (62.0%)	93 (59.2%)	125 (66.1%)	
**En block kidney (N (%))**	0 (0%)	4 (16%)	7 (20%)	0.18
**Dual kidney (N (%))**	0 (0%)	4 (16%)	3 (9%)	0.24
**Transplantation characteristics**				
**Cold ischemic time (hours), median (IQR)**	11.6 (3.6-20.4)	10.8 (3.3-18.4)	13.4 (4.3-20.2)	0.24
**cPRA (%), median (IQR)**	0 (0-23)	0 (0-0)	0 (0-0)	0.010
**Donors' characteristics**				
**Age (years), mean (SD)**	36.6 (13.7)	38.3 (15.4)	36.7 (16.1)	0.52
**Sex (N (%))**				0.013
male	43 (42.6%)	105 (60.7%)	114 (56.4%)	
female	58 (57.4%)	68 (39.3%)	88 (43.6%)	
**Race/ethnicity**				0.62
White	81 (80.2%)	138 (79.8%)	150 (74.3%)	
African American	3 (3.0%)	5 (2.9%)	11 (5.4%)	
Asian	2 (2.0%)	1 (0.6%)	6 (3.0%)	
Other	2 (2.0%)	3 (1.7%)	2 (1.0%)	
Unknown	13 (12.9%)	26 (15.0%)	33 (16.3%)	
**Donor type (N (%))**				0.29
Living	42 (41.6%)	76 (43.9%)	73 (36.1%)	
Deceased	59 (58.4%)	97 (56.1%)	129 (63.9%)	
**Donor DCD (N (%))**				0.90
No	36 (61.0%)	58 (59.8%)	81 (62.8%)	
Yes	23 (39.0%)	39 (40.2%)	48 (37.2%)	
**KDPI, median (IQR)**	25 (10-45)	23 (12-53)	22 (9-54)	0.85
**Donor cause of death (N (%))**				0.42
Anoxia	30 (50.8%)	37 (38.1%)	59 (45.7%)	
Cerebrovascular/stroke	11 (18.6%)	17 (17.5%)	14 (10.9%)	
Head trauma	16 (27.1%)	40 (41.2%)	49 (38.0%)	

Central nervous system tumor	1 (1.7%)	0 (0.0%)	2 (1.6%)	
Other	1 (1.7%)	3 (3.1%)	5 (3.9%)	
**Donor terminal creatinine (mg/dl), median (IQR)**	0.79 (0.69-0.96)	0.84 (0.70-1.03)	0.81 (0.62-1.0)	0.44
**Donor diabetes (N (%))**				0.48
No	95 (94.1%)	164 (95.9%)	193 (97.0%)	
Yes	6 (5.9%)	7 (4.1%)	6 (3.0%)	
**Donor hypertension (N (%))**				0.33
No	93 (93.0%)	152 (88.9%)	172 (87.3%)	
Yes	7 (7.0%)	19 (11.1%)	25 (12.7%)	
**Donor malignancy (N (%))**				0.18
No	101 (100.0%)	168 (97.1%)	195 (96.5%)	
Yes	0 (0.0%)	5 (2.9%)	7 (3.5%)	
**Immunological characteristics**				
**Number of HLA mismatches (HLA A, B and DR) (N (%))**				<0.001
1	10 (9.9%)	4 (2.3%)	2 (1.0%)	
2	21 (20.8%)	10 (5.8%)	4 (2.0%)	
3	28 (27.7%)	35 (20.2%)	17 (8.4%)	
4	21 (20.8%)	50 (28.9%)	46 (22.8%)	
5	16 (15.8%)	49 (28.3%)	78 (38.6%)	
6	5 (5.0%)	25 (14.5%)	55 (27.2%)	
**HLA mismatches A (N (%))**				0.26
0	6 (22%)	5 (10%)	3 (6%)	
1	11 (41%)	23 (47%)	27 (50%)	
2	10 (37%)	21 (43%)	24 (44%)	
**HLA mismatches B (N (%))**				0.26
0	0 (0%)	4 (8%)	3 (6%)	
1	9 (33%)	19 (39%)	13 (24%)	
2	18 (67%)	26 (53%)	38 (70%)	
**HLA mismatches DR (N (%))**				<0.001
0	23 (39.0%)	4 (4.0%)	4 (3.4%)	
1	30 (50.8%)	71 (71.7%)	48 (41.4%)	
2	6 (10.2%)	24 (24.2%)	64 (55.2%)	

**HLA mismatches DQ (N (%))**				<0.001
0	14 (52%)	6 (12%)	2 (4%)	
1	12 (44%)	25 (51%)	32 (60%)	
2	1 (4%)	18 (37%)	19 (36%)	
**Current flow B cell (N (%))**				0.98
< 2 SD compatible	54 (91.5%)	94 (94.0%)	115 (92.7%)	
2-3 SD compatible	3 (5.1%)	4 (4.0%)	5 (4.0%)	
weak positive	1 (1.7%)	1 (1.0%)	1 (0.8%)	
positive	1 (1.7%)	1 (1.0%)	3 (2.4%)	
**Current flow T cell (N (%))**				0.66
< 2 SD compatible	53 (89.8%)	96 (96.0%)	115 (92.7%)	
2-3 SD compatible	4 (6.8%)	2 (2.0%)	6 (4.8%)	
weak positive	0 (0.0%)	1 (1.0%)	1 (0.8%)	
positive	2 (3.4%)	1 (1.0%)	2 (1.6%)	
**Induction treatment (N (%))**				
No Induction	0 (0.0%)	0 (0.0%)	0 (0.0%)	NA
Basiliximab	13 (12.9%)	25 (14.5%)	22 (10.9%)	0.58
Thymoglobulin	81 (80.2%)	135 (78.0%)	173 (85.6%)	0.15
Steroid	101 (100.0%)	172 (99.4%)	202 (100.0%)	0.42
Alemtuzumab	3 (3.0%)	11 (6.4%)	3 (1.5%)	0.038
**<u>Outcomes</u>**				
**Proportion of Antibody Mediated Rejection of the subset of patients who underwent biopsy (N (%))**				0.14
No	27 (84%)	36 (67%)	51 (66%)	
Yes	5 (16%)	18 (33%)	26 (34%)	
**Proportion of rejection of the subset of patients who underwent biopsy (N (%))**				0.14
No	23 (72%)	28 (52%)	41 (53%)	
Yes	9 (28%)	26 (48%)	36 (47%)	
**Proportion of T-cell Mediated Rejection of the subset of patients who underwent biopsy (N (%))**				0.17

No	25 (78%)	33 (61%)	46 (60%)	
Yes	7 (22%)	21 (39%)	31 (40%)	
**Post-Transplant de novo Donor Specific Antibodies (N (%))**				0.091
No	88 (87.1%)	141 (81.5%)	155 (76.7%)	
Yes	13 (12.9%)	32 (18.5%)	47 (23.3%)	
**Dd-cf-DNA elevation (N (%))**				0.058
No	69 (87.3%)	110 (80.9%)	119 (74.4%)	
Yes	10 (12.7%)	26 (19.1%)	41 (25.6%)	
**Delayed Graft Function (N (%))**				0.11
No	96 (95.0%)	161 (93.1%)	179 (88.6%)	
Yes	5 (5.0%)	12 (6.9%)	23 (11.4%)	
**Death (N (%))**				0.045
No	99 (98.0%)	171 (98.8%)	191 (94.6%)	
Yes	2 (2.0%)	2 (1.2%)	11 (5.4%)	
**Graft Loss (N (%))**				0.39
No	100 (99.0%)	167 (96.5%)	198 (98.0%)	
Yes	1 (1.0%)	6 (3.5%)	4 (2.0%)	

Values are expressed as mean (standard deviation), median (interquartile range), or number (%). Continuous variables were compared via t-tests or Mann-Whitney U tests. Categorical variables were compared via Chi-square tests.

Table S18: Patient characteristics for the de novo DSA cohort based on eplet mismatch load for HLA-DQB1 locus

	Zero Eplet Mismatch Group	Low Eplet Mismatch Group	High Eplet Mismatch Group	p-value
**N**	86	372	192	
**Recipients' characteristics**				
**Age (years), mean (SD)**	50.6 (16.1)	49.9 (14.9)	50.3 (14.8)	0.90
**Sex (N (%))**				0.33
male	48 (55.8%)	229 (61.6%)	125 (65.1%)	
female	38 (44.2%)	143 (38.4%)	67 (34.9%)	
**Race/ethnicity (N (%))**				0.090
White	67 (77.9%)	252 (71.6%)	115 (61.5%)	
Hispanic	17 (19.8%)	61 (17.3%)	43 (23.0%)	
African American	0 (0.0%)	8 (2.3%)	6 (3.2%)	
Asian	1 (1.2%)	16 (4.5%)	9 (4.8%)	
Other	1 (1.2%)	14 (4.0%)	14 (7.5%)	
Unknown	0 (0.0%)	1 (0.3%)	0 (0.0%)	
**Body Mass Index (kg/m2), mean (SD)**	28.3 (5.6)	28.6 (5.7)	29.1 (5.7)	0.45
**Dialysis vintage (months), median (IQR)**	26.4 (12.1-45.8)	33.5 (15.5-55.7)	35.1 (18.9-59.8)	0.059
**Cause of kidney failure (N (%))**				0.73
Diabetes mellitus	22 (25.6%)	123 (33.0%)	63 (32.8%)	
Hypertension/Vascular disease	13 (15.1%)	45 (12.1%)	24 (12.5%)	
Cystic kidney disease	6 (7.0%)	32 (8.6%)	13 (6.8%)	
Glomerulonephritis	18 (20.9%)	84 (22.6%)	49 (25.5%)	
Other/unknown	27 (31.4%)	88 (23.7%)	43 (22.4%)	
**Prior kidney transplant (N (%))**				<0.001
No	69 (80.2%)	338 (90.9%)	183 (95.3%)	
Yes	17 (19.8%)	34 (9.1%)	9 (4.7%)	
**Multi-organ transplantation (N (%))**				0.81
Liver + kidney	1 (1.2%)	8 (2.2%)	6 (3.1%)	
Pancreas + kidney	1 (1.2%)	11 (3.0%)	6 (3.1%)	
Heart + kidney	1 (1.2%)	7 (1.9%)	3 (1.6%)	
None	83 (96.5%)	345 (92.7%)	175 (91.1%)	

Liver +Heart+Kidney	0 (0.0%)	1 (0.3%)	2 (1.0%)	
**CMV risk categories (N (%))**				0.44
Low	17 (19.8%)	103 (27.7%)	43 (22.4%)	
Intermediate	42 (48.8%)	156 (41.9%)	84 (43.8%)	
High	25 (29.1%)	99 (26.6%)	61 (31.8%)	
Unknown	2 (2.3%)	14 (3.8%)	4 (2.1%)	
**Was the donor organ pumped? (N (%))**				0.052
No	23 (29.5%)	110 (31.5%)	40 (21.6%)	
Yes	55 (70.5%)	239 (68.5%)	145 (78.4%)	
**En block kidney (N (%))**	0 (0%)	7 (12%)	7 (18%)	0.18
**Dual kidney (N (%))**	1 (6%)	7 (12%)	6 (16%)	0.63
**Transplantation characteristics**				
**Cold ischemic time (hours), median (IQR)**	12.6 (4.9-20.4)	12.1 (4.2-19.7)	14.8 (8.6-20.0)	0.073
**cPRA (%), median (IQR)**	0 (0-75)	0 (0-0)	0 (0-0)	<0.001
**Donors' characteristics**				
**Age (years), mean (SD)**	40.1 (14.8)	36.9 (15.5)	36.9 (16.5)	0.23
**Sex (N (%))**				0.24
male	42 (48.8%)	219 (58.9%)	109 (56.8%)	
female	44 (51.2%)	153 (41.1%)	83 (43.2%)	
**Race/ethnicity**				0.069
White	62 (72.1%)	313 (84.1%)	151 (78.6%)	
African American	3 (3.5%)	11 (3.0%)	6 (3.1%)	
Asian	1 (1.2%)	7 (1.9%)	5 (2.6%)	
Other	1 (1.2%)	7 (1.9%)	1 (0.5%)	
Unknown	19 (22.1%)	34 (9.1%)	29 (15.1%)	
**Donor type (N (%))**				0.022
Living	29 (33.7%)	126 (33.9%)	44 (22.9%)	
Deceased	57 (66.3%)	246 (66.1%)	148 (77.1%)	
**Donor DCD (N (%))**				0.49
No	31 (54.4%)	147 (59.8%)	80 (54.1%)	
Yes	26 (45.6%)	99 (40.2%)	68 (45.9%)	
**KDPI, median (IQR)**	32 (16-56)	24 (11-51)	32 (12-58)	0.16
**Donor cause of death (N (%))**				0.48
Anoxia	23 (40.4%)	119 (48.4%)	76 (51.4%)	
Cerebrovascular/stroke	8 (14.0%)	31 (12.6%)	26 (17.6%)	
Head trauma	23 (40.4%)	86 (35.0%)	43 (29.1%)	

Central nervous system tumor	1 (1.8%)	1 (0.4%)	1 (0.7%)	
Other	2 (3.5%)	9 (3.7%)	2 (1.4%)	
**Donor terminal creatinine (mg/dl), median (IQR)**	0.79 (0.69-1.07)	0.82 (0.64-1.0)	0.83 (0.66-1.03)	0.72
**Donor diabetes (N (%))**				0.41
No	80 (93.0%)	353 (96.2%)	180 (94.7%)	
Yes	6 (7.0%)	14 (3.8%)	10 (5.3%)	
**Donor hypertension (N (%))**				0.33
No	75 (88.2%)	313 (86.0%)	156 (82.1%)	
Yes	10 (11.8%)	51 (14.0%)	34 (17.9%)	
**Donor malignancy (N (%))**				0.098
No	83 (96.5%)	367 (98.7%)	184 (95.8%)	
Yes	3 (3.5%)	5 (1.3%)	8 (4.2%)	
**Immunological characteristics**				
**Number of HLA mismatches (HLA A, B and DR) (N (%))**				<0.001
1	12 (14.0%)	5 (1.3%)	0 (0.0%)	
2	14 (16.3%)	23 (6.2%)	3 (1.6%)	
3	33 (38.4%)	72 (19.4%)	16 (8.3%)	
4	16 (18.6%)	96 (25.8%)	47 (24.5%)	
5	10 (11.6%)	124 (33.3%)	77 (40.1%)	
6	1 (1.2%)	52 (14.0%)	49 (25.5%)	
**HLA mismatches A (N (%))**				0.048
0	3 (12%)	13 (15%)	1 (2%)	
1	14 (54%)	43 (49%)	17 (39%)	
2	9 (35%)	31 (36%)	26 (59%)	
**HLA mismatches B (N (%))**				0.40
0	0 (0%)	7 (8%)	1 (2%)	
1	9 (35%)	25 (29%)	12 (27%)	
2	17 (65%)	55 (63%)	31 (70%)	
**HLA mismatches DR (N (%))**				<0.001
0	27 (57.4%)	9 (4.9%)	3 (3.3%)	
1	15 (31.9%)	117 (63.2%)	35 (38.0%)	
2	5 (10.6%)	59 (31.9%)	54 (58.7%)	

**HLA mismatches DQ (N (%))**				<0.001
0	23 (88%)	6 (7%)	0 (0%)	
1	3 (12%)	65 (76%)	11 (25%)	
2	0 (0%)	15 (17%)	33 (75%)	
**Current flow B cell (N (%))**				0.98
< 2 SD compatible	44 (93.6%)	175 (91.6%)	90 (92.8%)	
2-3 SD compatible	1 (2.1%)	9 (4.7%)	4 (4.1%)	
weak positive	1 (2.1%)	2 (1.0%)	1 (1.0%)	
positive	1 (2.1%)	5 (2.6%)	2 (2.1%)	
**Current flow T cell (N (%))**				0.60
< 2 SD compatible	43 (91.5%)	178 (93.2%)	91 (93.8%)	
2-3 SD compatible	3 (6.4%)	5 (2.6%)	4 (4.1%)	
weak positive	1 (2.1%)	3 (1.6%)	0 (0.0%)	
positive	0 (0.0%)	5 (2.6%)	2 (2.1%)	
**Induction treatment (N (%))**				
No Induction	0 (0.0%)	0 (0.0%)	0 (0.0%)	NA
Basiliximab	11 (12.8%)	46 (12.4%)	18 (9.4%)	0.53
Thymoglobulin	69 (80.2%)	306 (82.3%)	170 (88.5%)	0.098
Steroid	86 (100.0%)	372 (100.0%)	191 (99.5%)	0.30
Alemtuzumab	4 (4.7%)	12 (3.2%)	3 (1.6%)	0.32
**Outcomes**				
**Proportion of Antibody Mediated Rejection of the subset of patients who underwent biopsy (N (%))**				0.16
No	26 (86.7%)	99 (74.4%)	50 (68.5%)	
Yes	4 (13.3%)	34 (25.6%)	23 (31.5%)	
**Proportion of rejection of the subset of patients who underwent biopsy (N (%))**				0.028
No	24 (80.0%)	75 (56.4%)	38 (52.1%)	
Yes	6 (20.0%)	58 (43.6%)	35 (47.9%)	
**Proportion of T-cell Mediated Rejection of the subset of patients who underwent biopsy (N (%))**				0.044

No	25 (83.3%)	87 (65.4%)	42 (57.5%)	
Yes	5 (16.7%)	46 (34.6%)	31 (42.5%)	
**Post-Transplant de novo Donor Specific Antibodies (N (%))**				<0.001
No	78 (90.7%)	308 (82.8%)	136 (70.8%)	
Yes	8 (9.3%)	64 (17.2%)	56 (29.2%)	
**Dd-cf-DNA elevation (N (%))**				0.24
No	55 (84.6%)	231 (78.8%)	111 (74.5%)	
Yes	10 (15.4%)	62 (21.2%)	38 (25.5%)	
**Delayed Graft Function (N (%))**				0.50
No	80 (93.0%)	343 (92.2%)	172 (89.6%)	
Yes	6 (7.0%)	29 (7.8%)	20 (10.4%)	
**Death (N (%))**				0.97
No	82 (95.3%)	355 (95.4%)	184 (95.8%)	
Yes	4 (4.7%)	17 (4.6%)	8 (4.2%)	
**Graft Loss (N (%))**				0.40
No	85 (98.8%)	366 (98.4%)	186 (96.9%)	
Yes	1 (1.2%)	6 (1.6%)	6 (3.1%)	

Values are expressed as mean (standard deviation), median (interquartile range), or number (%). Continuous variables were compared via t-tests or Mann-Whitney U tests. Categorical variables were compared via Chi-square tests.

Table S19: Patient characteristics for the de novo DSA cohort based on eplet mismatch load for HLA-DRB1 locus

	**Zero Eplet Mismatch Group**	**Low Eplet Mismatch Group**	**High Eplet Mismatch Group**	**p-value**
**N**	40	312	272	
**<u>Recipients' characteristics</u>**				
**Age (years), mean (SD)**	49.0 (16.4)	50.9 (15.0)	49.7 (15.1)	0.58
**Sex (N (%))**				0.39
male	21 (52.5%)	191 (61.2%)	173 (63.6%)	
female	19 (47.5%)	121 (38.8%)	99 (36.4%)	
**Race/ethnicity (N (%))**				0.25
White	32 (80.0%)	205 (67.7%)	181 (70.7%)	
Hispanic	7 (17.5%)	66 (21.8%)	38 (14.8%)	
African American	0 (0.0%)	8 (2.6%)	6 (2.3%)	
Asian	1 (2.5%)	12 (4.0%)	13 (5.1%)	
Other	0 (0.0%)	11 (3.6%)	18 (7.0%)	
Unknown	0 (0.0%)	1 (0.3%)	0 (0.0%)	
**Body Mass Index (kg/m2), mean (SD)**	28.0 (5.9)	28.6 (5.5)	28.9 (5.9)	0.57
**Dialysis vintage (months), median (IQR)**	24.4 (12.9-36.0)	33.8 (16.7-56.4)	34.9 (15.0-58.9)	0.10
**Cause of kidney failure (N (%))**				0.596
Diabetes mellitus	9 (22.5%)	94 (30.1%)	97 (35.7%)	
Hypertension/Vascular disease	5 (12.5%)	40 (12.8%)	34 (12.5%)	
Cystic kidney disease	3 (7.5%)	25 (8.0%)	21 (7.7%)	
Glomerulonephritis	9 (22.5%)	72 (23.1%)	64 (23.5%)	
Other/unknown	14 (35.0%)	81 (26.0%)	56 (20.6%)	
**Prior kidney transplant (N (%))**				<0.001
No	29 (72.5%)	277 (88.8%)	258 (94.9%)	
Yes	11 (27.5%)	35 (11.2%)	14 (5.1%)	
**Multi-organ transplantation (N (%))**				0.16
Liver + kidney	1 (2.5%)	8 (2.6%)	6 (2.2%)	
Pancreas + kidney	0 (0.0%)	3 (1.0%)	14 (5.1%)	
Heart + kidney	1 (2.5%)	5 (1.6%)	5 (1.8%)	
None	38 (95.0%)	295 (94.6%)	245 (90.1%)	

Liver +Heart+Kidney	0 (0.0%)	1 (0.3%)	2 (0.7%)	
**CMV risk categories (N (%))**				0.46
Low	8 (20.0%)	74 (23.7%)	75 (27.6%)	
Intermediate	18 (45.0%)	134 (42.9%)	122 (44.9%)	
High	14 (35.0%)	91 (29.2%)	68 (25.0%)	
Unknown	0 (0.0%)	13 (4.2%)	7 (2.6%)	
**Was the donor organ pumped? (N (%))**				0.66
No	11 (30.6%)	90 (30.9%)	71 (27.4%)	
Yes	25 (69.4%)	201 (69.1%)	188 (72.6%)	
**En block kidney (N (%))**	0 (0%)	7 (14%)	7 (16%)	0.45
**Dual kidney (N (%))**	0 (0%)	6 (12%)	5 (11%)	0.55
**Transplantation characteristics**				
**Cold ischemic time (hours), median (IQR)**	17.1 (4.2-22.2)	12.6 (4.6-19.3)	13.2 (5.8-20.2)	0.41
**cPRA (%), median (IQR)**	0 (0-97)	0 (0-3)	0 (0-0)	0.002
**Donors' characteristics**				
**Age (years), mean (SD)**	36.6 (15.6)	37.3 (15.2)	37.6 (16.4)	0.92
**Sex (N (%))**				0.65
male	20 (50.0%)	178 (57.1%)	157 (57.7%)	
female	20 (50.0%)	134 (42.9%)	115 (42.3%)	
**Race/ethnicity**				0.81
White	33 (82.5%)	255 (81.7%)	214 (78.7%)	
African American	0 (0.0%)	11 (3.5%)	10 (3.7%)	
Asian	0 (0.0%)	6 (1.9%)	7 (2.6%)	
Other	1 (2.5%)	5 (1.6%)	3 (1.1%)	
Unknown	6 (15.0%)	35 (11.2%)	38 (14.0%)	
**Donor type (N (%))**				0.54
Living	14 (35.0%)	104 (33.3%)	80 (29.4%)	
Deceased	26 (65.0%)	208 (66.7%)	192 (70.6%)	
**Donor DCD (N (%))**				0.072
No	18 (69.2%)	109 (52.4%)	119 (62.0%)	
Yes	8 (30.8%)	99 (47.6%)	73 (38.0%)	
**KDPI, median (IQR)**	20 (11-47)	28 (13-52)	28 (12-58)	0.45
**Donor cause of death (N (%))**				0.086
Anoxia	10 (38.5%)	97 (46.6%)	98 (51.0%)	
Cerebrovascular/stroke	7 (26.9%)	26 (12.5%)	26 (13.5%)	
Head trauma	8 (30.8%)	76 (36.5%)	63 (32.8%)	

Central nervous system tumor	1 (3.8%)	0 (0.0%)	2 (1.0%)	
Other	0 (0.0%)	9 (4.3%)	3 (1.6%)	
**Donor terminal creatinine (mg/dl), median (IQR)**	0.80 (0.68-1.05)	0.82 (0.65-1.03)	0.82 (0.65-1.03)	1.00
**Donor diabetes (N (%))**				0.36
No	40 (100.0%)	294 (95.1%)	255 (95.1%)	
Yes	0 (0.0%)	15 (4.9%)	13 (4.9%)	
**Donor hypertension (N (%))**				0.088
No	35 (89.7%)	273 (88.3%)	218 (82.3%)	
Yes	4 (10.3%)	36 (11.7%)	47 (17.7%)	
**Donor malignancy (N (%))**				0.59
No	40 (100.0%)	304 (97.4%)	265 (97.4%)	
Yes	0 (0.0%)	8 (2.6%)	7 (2.6%)	
**Immunological characteristics**				
**Number of HLA mismatches (HLA A, B and DR) (N (%))**				<0.001
1	10 (25.0%)	6 (1.9%)	1 (0.4%)	
2	11 (27.5%)	26 (8.3%)	3 (1.1%)	
3	10 (25.0%)	81 (26.0%)	20 (7.4%)	
4	7 (17.5%)	90 (28.8%)	57 (21.0%)	
5	2 (5.0%)	86 (27.6%)	112 (41.2%)	
6	0 (0.0%)	23 (7.4%)	79 (29.0%)	
**HLA mismatches A (N (%))**				0.19
0	2 (17%)	10 (12%)	5 (8%)	
1	4 (33%)	45 (55%)	24 (40%)	
2	6 (50%)	27 (33%)	31 (52%)	
**HLA mismatches B (N (%))**				0.16
0	0 (0%)	5 (6%)	2 (3%)	
1	3 (25%)	30 (37%)	12 (20%)	
2	9 (75%)	47 (57%)	46 (77%)	
**HLA mismatches DR (N (%))**				<0.001
0	17 (81.0%)	21 (12.7%)	0 (0.0%)	
1	3 (14.3%)	121 (72.9%)	42 (32.1%)	
2	1 (4.8%)	24 (14.5%)	89 (67.9%)	

**HLA mismatches DQ (N (%))**				<0.001
0	10 (83%)	16 (20%)	3 (5%)	
1	1 (8%)	52 (63%)	26 (44%)	
2	1 (8%)	14 (17%)	30 (51%)	
**Current flow B cell (N (%))**				0.65
< 2 SD compatible	20 (90.9%)	156 (91.8%)	127 (92.7%)	
2-3 SD compatible	1 (4.5%)	8 (4.7%)	6 (4.4%)	
weak positive	1 (4.5%)	1 (0.6%)	1 (0.7%)	
positive	0 (0.0%)	5 (2.9%)	3 (2.2%)	
**Current flow T cell (N (%))**				0.57
< 2 SD compatible	20 (90.9%)	158 (92.9%)	128 (93.4%)	
2-3 SD compatible	2 (9.1%)	5 (2.9%)	5 (3.6%)	
weak positive	0 (0.0%)	1 (0.6%)	2 (1.5%)	
positive	0 (0.0%)	6 (3.5%)	2 (1.5%)	
**Induction treatment (N (%))**				
No Induction	0 (0.0%)	0 (0.0%)	0 (0.0%)	NA
Basiliximab	2 (5.0%)	46 (14.7%)	26 (9.6%)	0.059
Thymoglobulin	34 (85.0%)	252 (80.8%)	234 (86.0%)	0.23
Steroid	40 (100.0%)	312 (100.0%)	271 (99.6%)	0.52
Alemtuzumab	2 (5.0%)	8 (2.6%)	9 (3.3%)	0.66
**Outcomes**				
**Proportion of Antibody Mediated Rejection of the subset of patients who underwent biopsy (N (%))**				0.078
No	11 (84.6%)	85 (79.4%)	71 (67.0%)	
Yes	2 (15.4%)	22 (20.6%)	35 (33.0%)	
**Proportion of rejection of the subset of patients who underwent biopsy (N (%))**				0.057
No	11 (84.6%)	65 (60.7%)	55 (51.9%)	
Yes	2 (15.4%)	42 (39.3%)	51 (48.1%)	
**Proportion of T-cell Mediated Rejection of the subset of patients who underwent biopsy (N (%))**				0.086

No	12 (92.3%)	70 (65.4%)	65 (61.3%)	
Yes	1 (7.7%)	37 (34.6%)	41 (38.7%)	
**Post-Transplant de novo Donor Specific Antibodies (N (%))**				0.011
No	36 (90.0%)	260 (83.3%)	204 (75.0%)	
Yes	4 (10.0%)	52 (16.7%)	68 (25.0%)	
**Dd-cf-DNA elevation (N (%))**				0.017
No	26 (92.9%)	194 (80.8%)	158 (72.8%)	
Yes	2 (7.1%)	46 (19.2%)	59 (27.2%)	
**Delayed Graft Function (N (%))**				0.47
No	38 (95.0%)	289 (92.6%)	246 (90.4%)	
Yes	2 (5.0%)	23 (7.4%)	26 (9.6%)	
**Death (N (%))**				0.048
No	40 (100.0%)	292 (93.6%)	264 (97.1%)	
Yes	0 (0.0%)	20 (6.4%)	8 (2.9%)	
**Graft Loss (N (%))**				0.63
No	40 (100.0%)	305 (97.8%)	266 (97.8%)	
Yes	0 (0.0%)	7 (2.2%)	6 (2.2%)	

Values are expressed as mean (standard deviation), median (interquartile range), or number (%). Continuous variables were compared via t-tests or Mann-Whitney U tests. Categorical variables were compared via Chi-square tests.

Table S20: Performance characteristics table of eplet mismatch load for HLA-A locus for de novo DSA

Cutpoint	Sensitivity	Specificity	Overall % correctly classified	LR+	LR-
(>= 1)	100.00%	0.00%	21.57%	1	
(>= 2)	99.09%	1.00%	22.16%	1.0009	0.9091
(>= 3)	98.18%	2.00%	22.75%	1.0019	0.9091
(>= 4)	95.45%	3.25%	23.14%	0.9866	1.3986
(>= 5)	93.64%	5.25%	24.31%	0.9882	1.2121
(>= 6)	90.00%	7.75%	25.49%	0.9756	1.2903
(>= 7)	83.64%	11.25%	26.86%	0.9424	1.4545
(>= 8)	78.18%	16.00%	29.41%	0.9307	1.3636
(>= 9)	76.36%	21.50%	33.33%	0.9728	1.0994
(>= 10)	70.00%	29.00%	37.84%	0.9859	1.0345
(>= 11)	64.55%	34.25%	40.78%	0.9817	1.0352
(>= 12)	61.82%	38.25%	43.33%	1.0011	0.9982
(>= 13)	53.64%	43.25%	45.49%	0.9451	1.072
(>= 14)	51.82%	48.75%	49.41%	1.0111	0.9883
(>= 15)	47.27%	53.50%	52.16%	1.0166	0.9856
(>= 16)	39.09%	58.25%	54.12%	0.9363	1.0456
(>= 17)	34.55%	64.75%	58.24%	0.98	1.0109
(>= 18)	33.64%	69.00%	61.37%	1.085	0.9618
(>= 19)	31.82%	72.00%	63.33%	1.1364	0.947
(>= 20)	30.00%	75.50%	65.69%	1.2245	0.9272
(>= 21)	25.45%	78.75%	67.25%	1.1979	0.9466
(>= 22)	22.73%	80.25%	67.84%	1.1507	0.9629
(>= 23)	18.18%	82.75%	68.82%	1.054	0.9887
(>= 24)	14.55%	82.75%	68.04%	0.8432	1.0327
(>= 25)	13.64%	85.50%	70.00%	0.9404	1.0101
(>= 26)	12.73%	87.25%	71.18%	0.9982	1.0003
(>= 27)	12.73%	89.75%	73.14%	1.2417	0.9724
(>= 28)	11.82%	91.00%	73.92%	1.3131	0.969
(>= 29)	10.00%	92.00%	74.31%	1.25	0.9783
(>= 30)	9.09%	93.25%	75.10%	1.3468	0.9749
(>= 31)	5.45%	93.50%	74.51%	0.8392	1.0112
(>= 32)	4.55%	94.75%	75.29%	0.8658	1.0074
(>= 33)	4.55%	95.50%	75.88%	1.0101	0.9995
(>= 34)	3.64%	96.50%	76.47%	1.039	0.9986

(>= 35)	1.82%	97.75%	77.06%	0.8081	1.0044
(>= 36)	1.82%	98.50%	77.65%	1.2121	0.9968
(>= 37)	1.82%	98.75%	77.84%	1.4545	0.9942
(>= 38)	0.91%	99.25%	78.04%	1.2121	0.9984
(>= 45)	0.91%	99.75%	78.43%	3.6364	0.9934
(>= 48)	0.91%	100.00%	78.63%	0.9909	
(> 48)	0.00%	100.00%	78.43%	1	

LR: Likelihood ratio

Table S21: Performance characteristics table of eplet mismatch load for HLA-B locus for de novo DSA

Cutpoint	Sensitivity	Specificity	Overall % correctly classified	LR+	LR-
(>= 1)	100.00%	0.00%	20.45%	1	
(>= 2)	99.09%	0.70%	20.82%	0.9979	1.297
(>= 3)	98.18%	1.87%	21.56%	1.0005	0.9727
(>= 4)	97.27%	3.50%	22.68%	1.0081	0.7782
(>= 5)	95.45%	5.61%	23.98%	1.0113	0.8106
(>= 6)	94.55%	10.28%	27.51%	1.0538	0.5306
(>= 7)	92.73%	12.85%	29.18%	1.064	0.566
(>= 8)	86.36%	16.36%	30.67%	1.0325	0.8338
(>= 9)	82.73%	22.66%	34.94%	1.0697	0.7621
(>= 10)	80.00%	26.40%	37.36%	1.087	0.7575
(>= 11)	75.45%	30.61%	39.78%	1.0874	0.8019
(>= 12)	70.00%	36.45%	43.31%	1.1015	0.8231
(>= 13)	62.73%	42.29%	46.47%	1.0869	0.8814
(>= 14)	58.18%	47.66%	49.81%	1.1117	0.8774
(>= 15)	50.91%	52.34%	52.04%	1.0681	0.938
(>= 16)	48.18%	58.41%	56.32%	1.1585	0.8871
(>= 17)	38.18%	63.08%	57.99%	1.0343	0.9799
(>= 18)	35.45%	67.52%	60.97%	1.0917	0.9559
(>= 19)	29.09%	72.66%	63.75%	1.0642	0.9759
(>= 20)	20.91%	75.93%	64.68%	0.8688	1.0416
(>= 21)	20.00%	79.67%	67.47%	0.9839	1.0041
(>= 22)	16.36%	83.18%	69.52%	0.9727	1.0055
(>= 23)	12.73%	84.81%	70.07%	0.838	1.029
(>= 24)	9.09%	87.62%	71.56%	0.7341	1.0376
(>= 25)	9.09%	89.25%	72.86%	0.8458	1.0186
(>= 26)	7.27%	91.36%	74.16%	0.8413	1.015
(>= 27)	5.45%	92.29%	74.54%	0.7074	1.0244
(>= 28)	5.45%	94.63%	76.39%	1.015	0.9991
(>= 29)	4.55%	95.33%	76.77%	0.9727	1.0013
(>= 30)	4.55%	96.73%	77.88%	1.3896	0.9868
(>= 31)	3.64%	97.43%	78.25%	1.4149	0.9891
(>= 32)	1.82%	97.90%	78.25%	0.8646	1.0029
(>= 33)	1.82%	98.36%	78.62%	1.1117	0.9981
(>= 34)	1.82%	98.60%	78.81%	1.297	0.9958

(>= 35)	1.82%	98.83%	79.00%	1.5564	0.9934
(>= 36)	0.91%	99.30%	79.18%	1.297	0.9979
(>= 38)	0.00%	99.53%	79.18%	0	1.0047
(>= 43)	0.00%	99.77%	79.37%	0	1.0023
(> 43)	0.00%	100.00%	79.55%	1	

LR: Likelihood ratio

Table S22: Performance characteristics table of eplet mismatch load for HLA-C locus for de novo DSA

Cutpoint	Sensitivity	Specificity	Overall % correctly classified	LR+	LR-
(>= 1)	100.00%	0.00%	20.04%	1	
(>= 2)	100.00%	4.22%	23.41%	1.044	0
(>= 3)	99.01%	7.69%	25.99%	1.0726	0.1287
(>= 4)	94.06%	12.41%	28.77%	1.0738	0.4788
(>= 5)	88.12%	18.61%	32.54%	1.0827	0.6384
(>= 6)	83.17%	24.57%	36.31%	1.1025	0.6852
(>= 7)	77.23%	31.27%	40.48%	1.1236	0.7284
(>= 8)	66.34%	39.70%	45.04%	1.1002	0.8479
(>= 9)	62.38%	47.89%	50.79%	1.197	0.7856
(>= 10)	55.45%	55.33%	55.36%	1.2414	0.8052
(>= 11)	51.49%	62.78%	60.52%	1.3832	0.7728
(>= 12)	37.62%	67.00%	61.11%	1.14	0.931
(>= 13)	31.68%	73.20%	64.88%	1.1823	0.9333
(>= 14)	28.71%	77.67%	67.86%	1.2857	0.9179
(>= 15)	20.79%	80.15%	68.25%	1.0474	0.9883
(>= 16)	19.80%	83.37%	70.63%	1.1911	0.9619
(>= 17)	14.85%	85.61%	71.43%	1.0319	0.9946
(>= 18)	12.87%	89.08%	73.81%	1.1789	0.9781
(>= 19)	11.88%	91.81%	75.79%	1.4509	0.9598
(>= 20)	10.89%	94.04%	77.38%	1.8288	0.9475
(>= 21)	6.93%	95.29%	77.58%	1.47	0.9767
(>= 22)	4.95%	97.02%	78.57%	1.6625	0.9797
(>= 23)	3.96%	97.77%	78.97%	1.7734	0.9823
(>= 24)	1.98%	98.51%	79.17%	1.33	0.995
(>= 25)	0.99%	98.76%	79.17%	0.798	1.0025
(>= 27)	0.00%	98.76%	78.97%	0	1.0126
(>= 28)	0.00%	99.26%	79.37%	0	1.0075
(>= 29)	0.00%	99.50%	79.56%	0	1.005
(> 29)	0.00%	100.00%	79.96%	1	

LR: Likelihood ratio

Table S23: Performance characteristics table of eplet mismatch load for HLA-DQA1 locus for de novo DSA

Cutpoint	Sensitivity	Specificity	Overall % correctly classified	LR+	LR-
(>= 1)	100.00%	0.00%	21.07%	1	
(>= 2)	89.87%	7.77%	25.07%	0.9745	1.3032
(>= 3)	73.42%	24.32%	34.67%	0.9702	1.0928
(>= 4)	59.49%	47.64%	50.13%	1.1361	0.8503
(>= 5)	41.77%	60.47%	56.53%	1.0568	0.9629
(>= 6)	31.65%	71.62%	63.20%	1.1151	0.9544
(>= 7)	20.25%	78.38%	66.13%	0.9367	1.0175
(>= 8)	11.39%	86.15%	70.40%	0.8225	1.0285
(>= 9)	8.86%	88.85%	72.00%	0.7948	1.0257
(>= 10)	7.59%	92.23%	74.40%	0.9774	1.0019
(>= 11)	7.59%	95.27%	76.80%	1.6058	0.9699
(>= 12)	7.59%	96.28%	77.60%	2.0437	0.9597
(>= 13)	5.06%	97.30%	77.87%	1.8734	0.9757
(>= 14)	2.53%	98.31%	78.13%	1.4987	0.9914
(>= 15)	1.27%	99.32%	78.67%	1.8734	0.9941
(> 15)	0.00%	100.00%	78.93%	1	

LR: Likelihood ratio

Table S24: Performance characteristics table of eplet mismatch load for HLA-DQB1 locus for de novo DSA

Cutpoint	Sensitivity	Specificity	Overall % correctly classified	LR+	LR-
(>= 1)	100.00%	0.00%	21.28%	1	
(>= 2)	97.50%	5.86%	25.35%	1.0356	0.4269
(>= 3)	95.83%	9.68%	28.01%	1.0611	0.4302
(>= 4)	91.67%	14.41%	30.85%	1.0711	0.5781
(>= 5)	88.33%	19.14%	33.87%	1.0925	0.6094
(>= 6)	83.33%	25.00%	37.41%	1.1111	0.6667
(>= 7)	75.00%	35.36%	43.79%	1.1603	0.707
(>= 8)	70.83%	42.57%	48.58%	1.2333	0.6852
(>= 9)	65.00%	49.32%	52.66%	1.2827	0.7096
(>= 10)	56.67%	53.15%	53.90%	1.2096	0.8153
(>= 11)	50.00%	62.61%	59.93%	1.3373	0.7986
(>= 12)	46.67%	69.37%	64.54%	1.5235	0.7688
(>= 13)	41.67%	72.30%	65.78%	1.5041	0.8069
(>= 14)	34.17%	77.70%	68.44%	1.5323	0.8472
(>= 15)	28.33%	81.98%	70.57%	1.5725	0.8742
(>= 16)	25.00%	84.68%	71.99%	1.6324	0.8856
(>= 17)	20.00%	86.49%	72.34%	1.48	0.925
(>= 18)	15.83%	88.96%	73.40%	1.4347	0.9461
(>= 19)	13.33%	90.99%	74.47%	1.48	0.9525
(>= 20)	11.67%	91.67%	74.65%	1.4	0.9636
(>= 21)	11.67%	93.47%	76.06%	1.7862	0.9451
(>= 22)	10.83%	95.05%	77.13%	2.1864	0.9382
(>= 23)	7.50%	96.40%	77.48%	2.0813	0.9596
(>= 24)	5.83%	96.85%	77.48%	1.85	0.9723
(>= 25)	4.17%	97.30%	77.48%	1.5417	0.985
(>= 26)	3.33%	97.30%	77.30%	1.2333	0.9935
(>= 27)	3.33%	97.52%	77.48%	1.3455	0.9912
(>= 28)	2.50%	97.97%	77.66%	1.2333	0.9952
(>= 29)	1.67%	98.65%	78.01%	1.2333	0.9968
(>= 30)	1.67%	98.87%	78.19%	1.48	0.9945
(>= 31)	0.00%	99.55%	78.37%	0	1.0045
(>= 32)	0.00%	99.77%	78.55%	0	1.0023
(> 32)	0.00%	100.00%	78.72%	1	

LR: Likelihood ratio

Table S25: Performance characteristics table of eplet mismatch load for HLA-DRB1 locus for de novo DSA

Cutpoint	Sensitivity	Specificity	Overall % correctly classified	LR+	LR-
(>= 1)	100.00%	0.00%	20.55%	1	
(>= 2)	98.33%	2.37%	22.09%	1.0072	0.703
(>= 3)	96.67%	4.96%	23.80%	1.0171	0.6725
(>= 4)	95.00%	5.82%	24.14%	1.0087	0.8593
(>= 5)	94.17%	8.84%	26.37%	1.0329	0.6602
(>= 6)	94.17%	9.48%	26.88%	1.0403	0.6152
(>= 7)	91.67%	12.72%	28.94%	1.0502	0.6554
(>= 8)	90.83%	17.03%	32.19%	1.0947	0.5384
(>= 9)	90.00%	20.47%	34.76%	1.1317	0.4884
(>= 10)	81.67%	24.57%	36.30%	1.0827	0.7462
(>= 11)	75.00%	30.60%	39.73%	1.0807	0.8169
(>= 12)	70.00%	37.07%	43.84%	1.1123	0.8093
(>= 13)	68.33%	42.67%	47.95%	1.192	0.7421
(>= 14)	63.33%	46.34%	49.83%	1.1802	0.7913
(>= 15)	60.83%	50.65%	52.74%	1.2326	0.7733
(>= 16)	56.67%	56.03%	56.16%	1.2889	0.7733
(>= 17)	50.00%	60.99%	58.73%	1.2818	0.8198
(>= 18)	44.17%	62.93%	59.08%	1.1915	0.8872
(>= 19)	36.67%	68.32%	61.82%	1.1574	0.927
(>= 20)	34.17%	71.55%	63.87%	1.201	0.9201
(>= 21)	31.67%	74.57%	65.75%	1.2452	0.9164
(>= 22)	28.33%	77.37%	67.29%	1.2521	0.9263
(>= 23)	27.50%	79.74%	69.01%	1.3574	0.9092
(>= 24)	24.17%	82.97%	70.89%	1.4194	0.9139
(>= 25)	23.33%	84.91%	72.26%	1.5467	0.9029
(>= 26)	21.67%	87.28%	73.80%	1.704	0.8974
(>= 27)	18.33%	88.79%	74.32%	1.6359	0.9197
(>= 28)	15.83%	89.87%	74.66%	1.5631	0.9365
(>= 29)	14.17%	91.81%	75.86%	1.7298	0.9349
(>= 30)	11.67%	92.89%	76.20%	1.6404	0.951
(>= 31)	8.33%	93.53%	76.03%	1.2889	0.98
(>= 32)	8.33%	94.40%	76.71%	1.4872	0.9711
(>= 33)	7.50%	95.04%	77.05%	1.513	0.9732
(>= 34)	7.50%	95.91%	77.74%	1.8316	0.9645

(>= 35)	7.50%	96.34%	78.08%	2.0471	0.9602
(>= 36)	6.67%	96.77%	78.25%	2.0622	0.9645
(>= 37)	6.67%	96.98%	78.42%	2.2095	0.9624
(>= 38)	3.33%	97.20%	77.91%	1.1897	0.9945
(>= 39)	3.33%	97.41%	78.08%	1.2889	0.9923
(>= 40)	3.33%	97.84%	78.42%	1.5467	0.988
(>= 41)	3.33%	98.28%	78.77%	1.9333	0.9836
(>= 42)	2.50%	98.49%	78.77%	1.6571	0.9899
(>= 43)	2.50%	99.14%	79.28%	2.9	0.9835
(>= 44)	2.50%	99.35%	79.45%	3.8667	0.9813
(>= 47)	2.50%	99.57%	79.62%	5.8	0.9792
(>= 48)	2.50%	99.78%	79.79%	11.6001	0.9771
(>= 52)	1.67%	100.00%	79.79%	0.9833	
(>= 55)	0.83%	100.00%	79.62%	0.9917	
(> 55)	0.00%	100.00%	79.45%	1	

LR: Likelihood ratio

Table S26: Association of class I HLA Eplet mismatch load and de novo DSA using Cox proportional regression models

Model		Hazard Ratio (HR)	95% Confidence Interval (CI) of HR	p-value		Hazard Ratio (HR)	95% Confidence Interval (CI) of HR	p-value		Hazard Ratio (HR)	95% Confidence Interval (CI) of HR	p-value
UNADJUSTED	**N=560**				**N=563**				**N=556**			
	**HLA-A** (+1 eplet increase)	1.013	0.993-1.033	0.193	**HLA-B** (+1 eplet increase)	1.014	0.990-1.038	0.254	**HLA-C** (+1 eplet increase)	1.021	0.991-1.052	0.173
	**HLA-A** (ref=Zero)				**HLA-B** (ref=Zero)				**HLA-C** (ref=Zero)			
	**Low**	3.451	1.089-10.937	0.035	**Low**	1.400	0.507-3.863	0.516	**Low**	0.723	0.384-1.361	0.315
	**High**	4.552	1.396-14.845	0.012	**High**	1.750	0.632-4.842	0.281	**High**	1.166	0.622-2.185	0.631
ADJUSTED*	**N=538**				**N=541**				**N=537**			
	**HLA-A** (+1 eplet increase)	1.006	0.985-1.027	0.604	**HLA-B** (+1 eplet increase)	1.018	0.993-1.044	0.157	**HLA-C** (+1 eplet increase)	1.024	0.992-1.057	0.140
	**HLA-A** (ref=Zero)				**HLA-B** (ref=Zero)				**HLA-C** (ref=Zero)			
	**Low**	4.616	1.117-19.078	0.035	**Low**	2.115	0.740-6.042	0.162	**Low**	0.865	0.438-1.707	0.676
	**High**	5.752	1.343-24.641	0.018	**High**	2.532	0.878-7.298	0.085	**High**	1.458	0.726-2.929	0.289

*adjusted for: recipient age, recipient sex, recipient race/ethnicity, recipient BMI, dialysis vintage, cause of end stage renal disease, preemptive kidney transplantation, history of prior kidney transplantation, multi-organ transplantation, type of induction therapy, donor age, donor sex, donor race/ethnicity, donor type [living vs. deceased], cold ischemic time, cytomegalovirus risk status, cPRA at allocation

Table S27: Association of class II HLA Eplet mismatch load and de novo DSA using Cox proportional regression models

Model		Hazard Ratio (HR)	95% Confidence Interval (CI) of HR	p-value		Hazard Ratio (HR)	95% Confidence Interval (CI) of HR	p-value		Hazard Ratio (HR)	95% Confidence Interval (CI) of HR	p-value
UNADJUSTED	**N=624**				**N=476**				**N=650**			
	**HLA-DRB1** (+1 eplet increase)	1.025	1.008-1.042	0.004	**HLA-DQA1** (+1 eplet increase)	1.054	0.993-1.120	0.086	**HLA-DQB1** (+1 eplet increase)	1.046	1.022-1.070	<0.001
	**HLA-DRB1** (ref=Zero)				**HLA-DQA1** (ref=Zero)				**HLA-DQB1** (ref=Zero)			
	**Low**	1.798	0.650-4.976	0.258	**Low**	1.493	0.783-2.844	0.223	**Low**	1.977	0.948-4.125	0.069
	**High**	2.705	0.986-7.422	0.053	**High**	2.020	1.093-3.735	0.025	**High**	3.461	1.650-7.263	0.001
ADJUSTED*	**N=599**				**N=459**				**N=625**			
	**HLA-DRB1** (+1 eplet increase)	1.027	1.009-1.046	0.003	**HLA-DQA1** (+1 eplet increase)	1.079	1.010-1.153	0.023	**HLA-DQB1** (+1 eplet increase)	1.043	1.018-1.068	0.001
	**HLA-DRB1** (ref=Zero)				**HLA-DQA1** (ref=Zero)				**HLA-DQB1** (ref=Zero)			
	**Low**	2.165	0.769-6.100	0.144	**Low**	1.608	0.798-3.242	0.184	**Low**	2.345	1.083-5.076	0.031
	**High**	3.157	1.114-8.946	0.031	**High**	2.335	1.194-4.566	0.013	**High**	3.739	1.719-8.133	0.001

*adjusted for: recipient age, recipient sex, recipient race/ethnicity, recipient BMI, dialysis vintage, cause of end stage renal disease, preemptive kidney transplantation, history of prior kidney transplantation, multi-organ transplantation, type of induction therapy, donor age, donor sex, donor race/ethnicity, donor type [living vs. deceased], cold ischemic time, cytomegalovirus risk status, cPRA at allocation

Table S28: Patient characteristics for the rejection cohort based on eplet mismatch load for HLA-A locus

	Zero Eplet Mismatch Group	Low Eplet Mismatch Group	High Eplet Mismatch Group	p-value
**N**	15	166	27	
**<u>Recipients' characteristics</u>**				
**Age (years), mean (SD)**	53.9 (15.9)	48.7 (15.1)	46.6 (13.7)	0.32
**Sex (N (%))**				0.60
male	10 (66.7%)	101 (60.8%)	19 (70.4%)	
female	5 (33.3%)	65 (39.2%)	8 (29.6%)	
**Race/ethnicity (N (%))**				0.11
White	12 (85.7%)	109 (68.6%)	11 (50.0%)	
Hispanic	2 (14.3%)	34 (21.4%)	5 (22.7%)	
African American	0 (0.0%)	3 (1.9%)	0 (0.0%)	
Asian	0 (0.0%)	7 (4.4%)	2 (9.1%)	
Other	0 (0.0%)	6 (3.8%)	4 (18.2%)	
Unknown	0 (0.0%)	0 (0.0%)	0 (0.0%)	
**Body Mass Index (kg/m2), mean (SD)**	27.7 (5.1)	29.3 (6.3)	30.1 (5.0)	0.31
**Dialysis vintage (months), median (IQR)**	19.5 (9.3-41.8)	33.1 (15.4-56.2)	45.1 (14.4-71.1)	0.19
**Cause of kidney failure (N (%))**				0.788
Diabetes mellitus	5 (33.3%)	46 (27.7%)	11 (40.7%)	
Hypertension/Vascular disease	2 (13.4%)	19 (11.5%)	2 (7.4%)	
Cystic kidney disease	0 (0.0%)	13 (7.8%)	1 (3.7%)	
Glomerulonephritis	3 (20.0%)	46 (27.7%)	8 (29.7%)	
Other/unknown	5 (33.3%)	42 (25.3%)	5 (18.5%)	
**Prior kidney transplant (N (%))**				0.081
No	12 (80.0%)	151 (91.0%)	27 (100.0%)	
Yes	3 (20.0%)	15 (9.0%)	0 (0.0%)	
**Multi-organ transplantation (N (%))**				0.85
Liver + kidney	0 (0.0%)	3 (1.8%)	0 (0.0%)	
Pancreas + kidney	0 (0.0%)	2 (1.2%)	1 (3.7%)	
Heart + kidney	0 (0.0%)	3 (1.8%)	0 (0.0%)	
None	15 (100.0%)	156 (94.0%)	25 (92.6%)	

Liver +Heart+Kidney	0 (0.0%)	2 (1.2%)	1 (3.7%)	
**CMV risk categories (N (%))**				0.64
Low	5 (33.3%)	37 (22.3%)	4 (14.8%)	
Intermediate	7 (46.7%)	69 (41.6%)	15 (55.6%)	
High	3 (20.0%)	57 (34.3%)	8 (29.6%)	
Unknown	0 (0.0%)	3 (1.8%)	0 (0.0%)	
**Was the donor organ pumped? (N (%))**				0.26
No	6 (42.9%)	40 (25.6%)	5 (19.2%)	
Yes	8 (57.1%)	116 (74.4%)	21 (80.8%)	
**En block kidney (N (%))**	0 (0%)	6 (24%)	1 (12%)	0.52
**Dual kidney (N (%))**	1 (33%)	2 (8%)	2 (25%)	0.29
**Transplantation characteristics**				
**Cold ischemic time (hours), median (IQR)**	12.5 (3.9-19.6)	13.6 (5.4-20.7)	17.7 (8.5-21.9)	0.37
**cPRA (%), median (IQR)**	0 (0-17)	0 (0-7)	0 (0-0)	0.28
**Donors' characteristics**				
**Age (years), mean (SD)**	45.7 (14.3)	38.4 (16.0)	34.9 (16.4)	0.11
**Sex (N (%))**				0.25
male	11 (73.3%)	89 (53.6%)	17 (63.0%)	
female	4 (26.7%)	77 (46.4%)	10 (37.0%)	
**Race/ethnicity**				<0.001
White	11 (73.3%)	133 (80.1%)	22 (81.5%)	
African American	0 (0.0%)	7 (4.2%)	0 (0.0%)	
Asian	0 (0.0%)	5 (3.0%)	1 (3.7%)	
Other	3 (20.0%)	1 (0.6%)	0 (0.0%)	
Unknown	1 (6.7%)	20 (12.0%)	4 (14.8%)	
**Donor type (N (%))**				0.32
Living	6 (40.0%)	46 (27.7%)	5 (18.5%)	
Deceased	9 (60.0%)	120 (72.3%)	22 (81.5%)	
**Donor DCD (N (%))**				0.42
No	4 (44.4%)	66 (55.0%)	9 (40.9%)	
Yes	5 (55.6%)	54 (45.0%)	13 (59.1%)	
**KDPI, median (IQR)**	40 (19-70)	33 (16-55)	33 (18-61)	0.83
**Donor cause of death (N (%))**				0.17
Anoxia	6 (66.7%)	57 (47.5%)	13 (59.1%)	
Cerebrovascular/stroke	1 (11.1%)	16 (13.3%)	3 (13.6%)	
Head trauma	1 (11.1%)	44 (36.7%)	5 (22.7%)	

Central nervous system tumor	1 (11.1%)	1 (0.8%)	0 (0.0%)	
Other	0 (0.0%)	2 (1.7%)	1 (4.5%)	
**Donor terminal creatinine (mg/dl), median (IQR)**	0.84 (0.53-1.04)	0.81 (0.65-1.0)	0.90 (0.60-1.14)	0.75
**Donor diabetes (N (%))**				0.36
No	14 (93.3%)	152 (92.7%)	26 (100.0%)	
Yes	1 (6.7%)	12 (7.3%)	0 (0.0%)	
**Donor hypertension (N (%))**				0.95
No	12 (80.0%)	133 (81.6%)	19 (79.2%)	
Yes	3 (20.0%)	30 (18.4%)	5 (20.8%)	
**Donor malignancy (N (%))**				0.080
No	15 (100.0%)	164 (98.8%)	25 (92.6%)	
Yes	0 (0.0%)	2 (1.2%)	2 (7.4%)	
**<u>Immunological characteristics</u>**				
**Number of HLA mismatches (HLA A, B and DR) (N (%))**				<0.001
1	2 (13.3%)	3 (1.8%)	0 (0.0%)	
2	4 (26.7%)	7 (4.2%)	0 (0.0%)	
3	7 (46.7%)	26 (15.7%)	1 (3.7%)	
4	2 (13.3%)	41 (24.7%)	3 (11.1%)	
5	0 (0.0%)	57 (34.3%)	12 (44.4%)	
6	0 (0.0%)	32 (19.3%)	11 (40.7%)	
**HLA mismatches A (N (%))**				<0.001
0	2 (100%)	1 (3%)	0 (0%)	
1	0 (0%)	16 (50%)	0 (0%)	
2	0 (0%)	15 (47%)	6 (100%)	
**HLA mismatches B (N (%))**				0.84
0	0 (0%)	1 (3%)	0 (0%)	
1	0 (0%)	9 (28%)	1 (17%)	
2	2 (100%)	22 (69%)	5 (83%)	
**HLA mismatches DR (N (%))**				0.36
0	1 (14%)	12 (16%)	1 (7%)	
1	5 (71%)	28 (37%)	7 (50%)	
2	1 (14%)	36 (47%)	6 (43%)	

**HLA mismatches DQ (N (%))**				0.81
0	0 (0%)	8 (25%)	1 (17%)	
1	1 (50%)	16 (50%)	4 (67%)	
2	1 (50%)	8 (25%)	1 (17%)	
**Current flow B cell (N (%))**				0.003
< 2 SD compatible	5 (71%)	76 (95%)	14 (93%)	
2-3 SD compatible	2 (29%)	1 (1%)	0 (0%)	
weak positive	0 (0%)	1 (1%)	1 (7%)	
positive	0 (0%)	2 (2%)	0 (0%)	
**Current flow T cell (N (%))**				0.026
< 2 SD compatible	4 (57%)	74 (92%)	15 (100%)	
2-3 SD compatible	2 (29%)	4 (5%)	0 (0%)	
weak positive	0 (0%)	1 (1%)	0 (0%)	
positive	1 (14%)	1 (1%)	0 (0%)	
**Induction treatment (N (%))**				
No Induction	0 (0%)	0 (0%)	0 (0%)	NA
Basiliximab	3 (20.0%)	19 (11.4%)	4 (14.8%)	0.58
Thymoglobulin	12 (80.0%)	143 (86.1%)	24 (88.9%)	0.73
Steroid	15 (100.0%)	166 (100.0%)	27 (100.0%)	NA
Alemtuzumab	0 (0.0%)	3 (1.8%)	0 (0.0%)	0.68
**Outcomes**				
**Proportion of Antibody Mediated Rejection of the subset of patients who underwent biopsy (N (%))**				0.53
No	13 (86.7%)	123 (74.1%)	21 (77.8%)	
Yes	2 (13.3%)	43 (25.9%)	6 (22.2%)	
**Proportion of rejection of the subset of patients who underwent biopsy (N (%))**				0.39
No	10 (66.7%)	101 (60.8%)	13 (48.1%)	
Yes	5 (33.3%)	65 (39.2%)	14 (51.9%)	
**Proportion of T-cell Mediated Rejection of the subset of patients who underwent biopsy (N (%))**				0.13

No	11 (73.3%)	111 (66.9%)	13 (48.1%)	
Yes	4 (26.7%)	55 (33.1%)	14 (51.9%)	
**Post-Transplant de novo Donor Specific Antibodies (N (%))**				0.056
No	13 (86.7%)	115 (69.3%)	14 (51.9%)	
Yes	2 (13.3%)	51 (30.7%)	13 (48.1%)	
**Dd-cf-DNA elevation (N (%))**				0.29
No	7 (63.6%)	82 (63.1%)	10 (45.5%)	
Yes	4 (36.4%)	48 (36.9%)	12 (54.5%)	
**Delayed Graft Function (N (%))**				0.57
No	14 (93.3%)	139 (83.7%)	22 (81.5%)	
Yes	1 (6.7%)	27 (16.3%)	5 (18.5%)	
**Death (N (%))**				0.91
No	14 (93.3%)	158 (95.2%)	26 (96.3%)	
Yes	1 (6.7%)	8 (4.8%)	1 (3.7%)	
**Graft Loss (N (%))**				0.30
No	15 (100.0%)	157 (94.6%)	27 (100.0%)	
Yes	0 (0.0%)	9 (5.4%)	0 (0.0%)	

Values are expressed as mean (standard deviation), median (interquartile range), or number (%). Continuous variables were compared via t-tests or Mann-Whitney U tests. Categorical variables were compared via Chi-square tests.

Table S29: Patient characteristics for the rejection cohort based on eplet mismatch load for HLA-B locus

	**Zero Eplet Mismatch Group**	**Low Eplet Mismatch Group**	**High Eplet Mismatch Group**	**p-value**
**N**	8	117	79	
**Recipients' characteristics**				
**Age (years), mean (SD)**	39.8 (17.6)	48.6 (15.5)	49.9 (13.4)	0.18
**Sex (N (%))**				0.60
male	6 (75.0%)	70 (59.8%)	51 (64.6%)	
female	2 (25.0%)	47 (40.2%)	28 (35.4%)	
**Race/ethnicity (N (%))**				0.39
White	8 (100.0%)	71 (64.5%)	51 (69.9%)	
Hispanic	0 (0.0%)	26 (23.6%)	13 (17.8%)	
African American	0 (0.0%)	2 (1.8%)	2 (2.7%)	
Asian	0 (0.0%)	3 (2.7%)	5 (6.8%)	
Other	0 (0.0%)	8 (7.3%)	2 (2.7%)	
Unknown	0 (0.0%)	0 (0.0%)	0 (0.0%)	
**Body Mass Index (kg/m2), mean (SD)**	28.6 (6.7)	29.8 (6.0)	28.9 (6.1)	0.57
**Dialysis vintage (months), median (IQR)**	25.5 (19.4-36.0)	33.8 (14.0-57.1)	34.5 (17.2-56.2)	0.674
**Cause of kidney failure (N (%))**				0.350
Diabetes mellitus	2 (25.0%)	31 (26.5%)	26 (32.9%)	
Hypertension/Vascular disease	0 (0.0%)	14 (12.0%)	9 (11.4%)	
Cystic kidney disease	0 (0.0%)	9 (7.7%)	5 (6.3%)	
Glomerulonephritis	1 (12.5%)	37 (31.6%)	19 (24.1%)	
Other/unknown	5 (62.5%)	26 (22.2%)	20 (25.3%)	
**Prior kidney transplant (N (%))**				0.25
No	6 (75.0%)	107 (91.5%)	73 (92.4%)	
Yes	2 (25.0%)	10 (8.5%)	6 (7.6%)	
**Multi-organ transplantation (N (%))**				0.51
Liver + kidney	0 (0.0%)	2 (1.7%)	1 (1.3%)	
Pancreas + kidney	0 (0.0%)	1 (0.9%)	2 (2.5%)	
Heart + kidney	0 (0.0%)	0 (0.0%)	3 (3.8%)	
None	8 (100.0%)	113 (96.6%)	71 (89.9%)	

Liver +Heart+Kidney	0 (0.0%)	1 (0.9%)	2 (2.5%)	
**CMV risk categories (N (%))**				0.65
Low	1 (12.5%)	29 (24.8%)	14 (17.7%)	
Intermediate	3 (37.5%)	45 (38.5%)	40 (50.6%)	
High	4 (50.0%)	41 (35.0%)	24 (30.4%)	
Unknown	0 (0.0%)	2 (1.7%)	1 (1.3%)	
**Was the donor organ pumped? (N (%))**				0.77
No	3 (37.5%)	29 (25.9%)	19 (26.4%)	
Yes	5 (62.5%)	83 (74.1%)	53 (73.6%)	
**Transplantation characteristics**				
**Cold ischemic time (hours), median (IQR)**	16.3 (3.7-23.6)	14.8 (5.8-20.7)	13.7 (6.8-20.2)	0.79
**cPRA (%), median (IQR)**	64 (0-99)	0 (0-0)	0 (0-0)	0.010
**Donors' characteristics**				
**Age (years), mean (SD)**	47.3 (9.5)	40.4 (15.7)	35.5 (16.1)	0.031
**Sex (N (%))**				0.037
male	1 (12.5%)	69 (59.0%)	45 (57.0%)	
female	7 (87.5%)	48 (41.0%)	34 (43.0%)	
**Race/ethnicity**				0.24
White	8 (100.0%)	88 (75.2%)	68 (86.1%)	
African American	0 (0.0%)	6 (5.1%)	1 (1.3%)	
Asian	0 (0.0%)	5 (4.3%)	1 (1.3%)	
Other	0 (0.0%)	1 (0.9%)	3 (3.8%)	
Unknown	0 (0.0%)	17 (14.5%)	6 (7.6%)	
**Donor type (N (%))**				0.64
Living	3 (37.5%)	30 (25.6%)	24 (30.4%)	
Deceased	5 (62.5%)	87 (74.4%)	55 (69.6%)	
**Donor DCD (N (%))**				0.80
No	2 (40%)	47 (54%)	28 (51%)	
Yes	3 (60%)	40 (46%)	27 (49%)	
**KDPI, median (IQR)**	42 (42-44)	28 (13-58)	33 (20-59)	0.74
**Donor cause of death (N (%))**				0.92
Anoxia	3 (60%)	44 (51%)	28 (51%)	
Cerebrovascular/stroke	1 (20%)	11 (13%)	6 (11%)	
Head trauma	1 (20%)	29 (33%)	19 (35%)	
Central nervous system tumor	0 (0%)	2 (2%)	0 (0%)	

Other	0 (0%)	1 (1%)	2 (4%)	
**Donor terminal creatinine (mg/dl), median (IQR)**	0.89 (0.75-1.13)	0.83 (0.66-1.04)	0.81 (0.56-1.05)	0.50
**Donor diabetes (N (%))**				0.53
No	8 (100.0%)	108 (93.1%)	74 (96.1%)	
Yes	0 (0.0%)	8 (6.9%)	3 (3.9%)	
**Donor hypertension (N (%))**				0.43
No	7 (87.5%)	90 (78.3%)	64 (85.3%)	
Yes	1 (12.5%)	25 (21.7%)	11 (14.7%)	
**Donor malignancy (N (%))**				0.040
No	7 (87.5%)	114 (97.4%)	79 (100.0%)	
Yes	1 (12.5%)	3 (2.6%)	0 (0.0%)	
**<u>Immunological characteristics</u>**				
**Number of HLA mismatches (HLA A, B and DR) (N (%))**				<0.001
1	2 (25.0%)	2 (1.7%)	0 (0.0%)	
2	1 (12.5%)	9 (7.7%)	1 (1.3%)	
3	2 (25.0%)	25 (21.4%)	5 (6.3%)	
4	2 (25.0%)	26 (22.2%)	18 (22.8%)	
5	1 (12.5%)	34 (29.1%)	33 (41.8%)	
6	0 (0.0%)	21 (17.9%)	22 (27.8%)	
**HLA mismatches A (N (%))**				0.76
0	0 (0%)	1 (4%)	2 (12%)	
1	0 (0%)	10 (43%)	6 (38%)	
2	1 (100%)	12 (52%)	8 (50%)	
**HLA mismatches B (N (%))**				0.082
0	0 (0%)	1 (4%)	0 (0%)	
1	1 (100%)	8 (35%)	1 (6%)	
2	0 (0%)	14 (61%)	15 (94%)	
**HLA mismatches DR (N (%))**				0.15
0	2 (50%)	8 (16%)	4 (10%)	
1	1 (25%)	24 (47%)	15 (37%)	
2	1 (25%)	19 (37%)	22 (54%)	
**HLA mismatches DQ (N (%))**				0.50

0	0 (0%)	6 (26%)	3 (19%)	
1	0 (0%)	12 (52%)	9 (56%)	
2	1 (100%)	5 (22%)	4 (25%)	
**Current flow B cell (N (%))**				0.74
< 2 SD compatible	4 (100%)	48 (91%)	41 (93%)	
2-3 SD compatible	0 (0%)	1 (2%)	2 (5%)	
weak positive	0 (0%)	1 (2%)	1 (2%)	
positive	0 (0%)	3 (6%)	0 (0%)	
**Current flow T cell (N (%))**				0.55
< 2 SD compatible	3 (75%)	47 (89%)	42 (95%)	
2-3 SD compatible	1 (25%)	4 (8%)	1 (2%)	
weak positive	0 (0%)	1 (2%)	0 (0%)	
positive	0 (0%)	1 (2%)	1 (2%)	
**Induction treatment (N (%))**				
No Induction	0 (0%)	0 (0%)	0 (0%)	NA
Basiliximab	1 (12.5%)	14 (12.0%)	12 (15.2%)	0.81
Thymoglobulin	7 (87.5%)	99 (84.6%)	67 (84.8%)	0.98
Steroid	8 (100.0%)	117 (100.0%)	79 (100.0%)	NA
Alemtuzumab	0 (0.0%)	3 (2.6%)	1 (1.3%)	0.75
**<u>Outcomes</u>**				
**Proportion of Antibody Mediated Rejection of the subset of patient underwent biopsy (N (%))**				0.21
No	5 (62.5%)	93 (79.5%)	55 (69.6%)	
Yes	3 (37.5%)	24 (20.5%)	24 (30.4%)	
**Proportion of rejection of the subset of patients who underwent biopsy (N (%))**				0.56
No	4 (50.0%)	73 (62.4%)	44 (55.7%)	
Yes	4 (50.0%)	44 (37.6%)	35 (44.3%)	
**Proportion of T-cell Mediated Rejection of the subset of patients who underwent biopsy (N (%))**				0.49
No	4 (50.0%)	79 (67.5%)	49 (62.0%)	

Yes	4 (50.0%)	38 (32.5%)	30 (38.0%)	
**Post-Transplant de novo Donor Specific Antibodies (N (%))**				0.45
No	7 (87.5%)	80 (68.4%)	52 (65.8%)	
Yes	1 (12.5%)	37 (31.6%)	27 (34.2%)	
**Dd-cf-DNA elevation (N (%))**				0.61
No	4 (80%)	57 (59%)	36 (62%)	
Yes	1 (20%)	40 (41%)	22 (38%)	
**Delayed Graft Function (N (%))**				0.28
No	8 (100.0%)	96 (82.1%)	69 (87.3%)	
Yes	0 (0.0%)	21 (17.9%)	10 (12.7%)	
**Death (N (%))**				0.32
No	8 (100.0%)	109 (93.2%)	77 (97.5%)	
Yes	0 (0.0%)	8 (6.8%)	2 (2.5%)	
**Graft Loss (N (%))**				0.013
No	6 (75.0%)	112 (95.7%)	77 (97.5%)	
Yes	2 (25.0%)	5 (4.3%)	2 (2.5%)	

Values are expressed as mean (standard deviation), median (interquartile range), or number (%). Continuous variables were compared via t-tests or Mann-Whitney U tests. Categorical variables were compared via Chi-square tests.

Table S30: Patient characteristics for the rejection cohort based on eplet mismatch load for HLA-C locus

	Zero Eplet Mismatch Group	Low Eplet Mismatch Group	High Eplet Mismatch Group	p-value
**N**	21	93	91	
**<u>Recipients' characteristics</u>**				
**Age (years), mean (SD)**	44.3 (14.0)	48.5 (16.5)	49.5 (13.5)	0.37
**Sex (N (%))**				0.86
male	12 (57%)	59 (63%)	57 (63%)	
female	9 (43%)	34 (37%)	34 (37%)	
**Race/ethnicity (N (%))**				0.23
White	16 (76%)	64 (71%)	50 (60%)	
Hispanic	2 (10%)	17 (19%)	23 (28%)	
African American	1 (5%)	3 (3%)	0 (0%)	
Asian	0 (0%)	3 (3%)	5 (6%)	
Other	2 (10%)	3 (3%)	5 (6%)	
Unknown	0 (0%)	0 (0%)	0 (0%)	
**Body Mass Index (kg/m2), mean (SD)**	26.5 (5.4)	29.1 (6.0)	30.0 (6.2)	0.059
**Dialysis vintage (months), median (IQR)**	41.8 (15.4-65.5)	25.9 (12.6-44.9)	37.6 (23.0-60.3)	0.040
**Cause of kidney failure (N (%))**				0.99
Diabetes mellitus	5 (23.8%)	27 (29.0%)	27 (29.6%)	
Hypertension/Vascular disease	2 (9.5%)	11 (11.8%)	10 (11.0%)	
Cystic kidney disease	1 (4.8%)	8 (8.6%)	5 (5.5%)	
Glomerulonephritis	7 (33.3%)	25 (26.9%)	25 (27.5%)	
Other/unknown	6 (28.6%)	22 (23.7%)	24 (26.4%)	
**Prior kidney transplant (N (%))**				0.005
No	15 (71%)	87 (94%)	84 (92%)	
Yes	6 (29%)	6 (6%)	7 (8%)	
**Multi-organ transplantation (N (%))**				0.35
Liver + kidney	0 (0%)	2 (2%)	1 (1%)	
Pancreas + kidney	0 (0%)	3 (3%)	0 (0%)	
Heart + kidney	0 (0%)	1 (1%)	2 (2%)	
None	21 (100%)	87 (94%)	85 (93%)	

Liver +Heart+Kidney	0 (0%)	0 (0%)	3 (3%)	
**CMV risk categories (N (%))**				0.58
Low	2 (10%)	20 (22%)	20 (22%)	
Intermediate	9 (43%)	38 (41%)	43 (47%)	
High	9 (43%)	34 (37%)	27 (30%)	
Unknown	1 (5%)	1 (1%)	1 (1%)	
**Was the donor organ pumped? (N (%))**				0.29
No	6 (29%)	27 (31%)	18 (21%)	
Yes	15 (71%)	59 (69%)	68 (79%)	
**En block kidney (N (%))**	0 (0%)	5 (26%)	1 (8%)	0.24
**Dual kidney (N (%))**	0 (0%)	2 (11%)	3 (23%)	0.42
**Transplantation characteristics**				
**Cold ischemic time (hours), median (IQR)**	15.4 (7.9-23.0)	12.2 (3.9-20.5)	16.2 (8.4-20.4)	0.24
**cPRA (%), median (IQR)**	16 (0-99)	0 (0-0)	0 (0-0)	0.002
**Donors' characteristics**				
**Age (years), mean (SD)**	41.2 (15.4)	37.8 (16.6)	38.5 (15.8)	0.69
**Sex (N (%))**				0.34
male	9 (43%)	52 (56%)	55 (60%)	
female	12 (57%)	41 (44%)	36 (40%)	
**Race/ethnicity**				0.78
White	19 (90%)	74 (80%)	70 (77%)	
African American	0 (0%)	3 (3%)	5 (5%)	
Asian	0 (0%)	3 (3%)	3 (3%)	
Other	1 (5%)	1 (1%)	2 (2%)	
Unknown	1 (5%)	12 (13%)	11 (12%)	
**Donor type (N (%))**				0.16
Living	5 (24%)	32 (34%)	20 (22%)	
Deceased	16 (76%)	61 (66%)	71 (78%)	
**Donor DCD (N (%))**				0.007
No	7 (44%)	42 (69%)	30 (42%)	
Yes	9 (56%)	19 (31%)	41 (58%)	
**KDPI, median (IQR)**	36 (20-41)	33 (15-53)	33 (16-64)	0.75
**Donor cause of death (N (%))**				0.28
Anoxia	8 (50%)	31 (51%)	34 (48%)	
Cerebrovascular/stroke	1 (6%)	8 (13%)	12 (17%)	
Head trauma	6 (38%)	18 (30%)	25 (35%)	

Central nervous system tumor	1 (6%)	1 (2%)	0 (0%)	
Other	0 (0%)	3 (5%)	0 (0%)	
**Donor terminal creatinine (mg/dl), median (IQR)**	0.83 (0.76-1.06)	0.84 (0.65-1.07)	0.81 (0.60-1.03)	0.36
**Donor diabetes (N (%))**				0.99
No	20 (95%)	85 (94%)	86 (95%)	
Yes	1 (5%)	5 (6%)	5 (5%)	
**Donor hypertension (N (%))**				0.072
No	19 (90%)	78 (87%)	66 (75%)	
Yes	2 (10%)	12 (13%)	22 (25%)	
**Donor malignancy (N (%))**				0.18
No	20 (95%)	90 (97%)	91 (100%)	
Yes	1 (5%)	3 (3%)	0 (0%)	
**Immunological characteristics**				
**Number of HLA mismatches (HLA A, B and DR) (N (%))**				<0.001
1	4 (19%)	1 (1%)	0 (0%)	
2	4 (19%)	6 (6%)	2 (2%)	
3	5 (24%)	21 (23%)	7 (8%)	
4	4 (19%)	19 (20%)	22 (24%)	
5	4 (19%)	25 (27%)	37 (41%)	
6	0 (0%)	21 (23%)	23 (25%)	
**HLA mismatches A (N (%))**				0.29
0	0 (0%)	1 (5%)	2 (13%)	
1	4 (80%)	9 (47%)	4 (27%)	
2	1 (20%)	9 (47%)	9 (60%)	
**HLA mismatches B (N (%))**				0.015
0	1 (20%)	0 (0%)	0 (0%)	
1	3 (60%)	5 (26%)	2 (13%)	
2	1 (20%)	14 (74%)	13 (87%)	
**HLA mismatches DR (N (%))**				0.43
0	3 (38%)	6 (13%)	6 (15%)	
1	3 (38%)	20 (44%)	15 (37%)	
2	2 (25%)	19 (42%)	20 (49%)	

**HLA mismatches DQ (N (%))**				0.90
0	2 (40%)	4 (21%)	4 (27%)	
1	2 (40%)	11 (58%)	7 (47%)	
2	1 (20%)	4 (21%)	4 (27%)	
**Current flow B cell (N (%))**				0.50
< 2 SD compatible	8 (89%)	43 (96%)	40 (89%)	
2-3 SD compatible	0 (0%)	1 (2%)	2 (4%)	
weak positive	0 (0%)	0 (0%)	2 (4%)	
positive	1 (11%)	1 (2%)	1 (2%)	
**Current flow T cell (N (%))**				0.088
< 2 SD compatible	7 (78%)	42 (93%)	41 (91%)	
2-3 SD compatible	2 (22%)	0 (0%)	4 (9%)	
weak positive	0 (0%)	1 (2%)	0 (0%)	
positive	0 (0%)	2 (4%)	0 (0%)	
**Induction treatment (N (%))**				
No Induction	0 (0%)	0 (0%)	0 (0%)	NA
Basiliximab	1 (5%)	18 (19%)	8 (9%)	0.051
Thymoglobulin	19 (90%)	74 (80%)	81 (89%)	0.15
Steroid	21 (100%)	93 (100%)	91 (100%)	NA
Alemtuzumab	1 (5%)	1 (1%)	2 (2%)	0.53
**<u>Outcomes</u>**				
**Proportion of Antibody Mediated Rejection of the subset of patients who underwent biopsy (N (%))**				0.80
No	15 (71%)	71 (76%)	66 (73%)	
Yes	6 (29%)	22 (24%)	25 (27%)	
**Proportion of rejection of the subset of patients who underwent biopsy (N (%))**				0.19
No	14 (67%)	59 (63%)	47 (52%)	
Yes	7 (33%)	34 (37%)	44 (48%)	
**Proportion of T-cell Mediated Rejection of the subset of patients who underwent biopsy (N (%))**				0.25

No	14 (67%)	65 (70%)	53 (58%)	
Yes	7 (33%)	28 (30%)	38 (42%)	
**Post-Transplant de novo Donor Specific Antibodies (N (%))**				0.39
No	14 (67%)	68 (73%)	58 (64%)	
Yes	7 (33%)	25 (27%)	33 (36%)	
**Dd-cf-DNA elevation (N (%))**				0.013
No	10 (67%)	52 (72%)	35 (49%)	
Yes	5 (33%)	20 (28%)	37 (51%)	
**Delayed Graft Function (N (%))**				0.009
No	19 (90%)	86 (92%)	70 (77%)	
Yes	2 (10%)	7 (8%)	21 (23%)	
**Death (N (%))**				0.45
No	21 (100%)	87 (94%)	87 (96%)	
Yes	0 (0%)	6 (6%)	4 (4%)	
**Graft Loss (N (%))**				0.45
No	19 (90%)	90 (97%)	86 (95%)	
Yes	2 (10%)	3 (3%)	5 (5%)	

Values are expressed as mean (standard deviation), median (interquartile range), or number (%). Continuous variables were compared via t-tests or Mann-Whitney U tests. Categorical variables were compared via Chi-square tests.

Table S31: Patient characteristics for the rejection cohort based on eplet mismatch load for HLA-DQA1 locus

	**Zero Eplet Mismatch Group**	**Low Eplet Mismatch Group**	**High Eplet Mismatch Group**	**p-value**
**N**	32	121	10	
**<u>Recipients' characteristics</u>**				
**Age (years), mean (SD)**	47.5 (14.6)	48.0 (15.5)	50 (15.1)	0.90
**Sex (N (%))**				0.97
male	20 (62.5%)	77 (63.6%)	6 (60.0%)	
female	12 (37.5%)	44 (36.4%)	4 (40.0%)	
**Race/ethnicity (N (%))**				0.48
White	20 (64.5%)	77 (69.4%)	7 (77.8%)	
Hispanic	8 (25.8%)	21 (18.9%)	2 (22.2%)	
African American	2 (6.5%)	1 (0.9%)	0 (0.0%)	
Asian	0 (0.0%)	6 (5.4%)	0 (0.0%)	
Other	1 (3.2%)	6 (5.4%)	0 (0.0%)	
Unknown	0 (0.0%)	0 (0.0%)	0 (0.0%)	
**Body Mass Index (kg/m2), mean (SD)**	28.8 (6.5)	29.1 (5.6)	29.3 (6.6)	0.95
**Dialysis vintage (months), median (IQR)**	23.3 (12.4-37.0)	37.6 (15.4-59.9)	34.3 (29.9-77.0)	0.073
**Cause of kidney failure (N (%))**				0.614
Diabetes mellitus	7 (21.9%)	33 (27.3%)	2 (20.0%)	
Hypertension/Vascular disease	2 (6.2%)	16 (13.2%)	2 (20.0%)	
Cystic kidney disease	1 (3.1%)	10 (8.2%)	0 (0.0%)	
Glomerulonephritis	12 (37.5%)	29 (24.0%)	4 (40.0%)	
Other/unknown	10 (31.3%)	33 (27.3%)	2 (20.0%)	
**Prior kidney transplant (N (%))**				0.074
No	24 (75.0%)	109 (90.1%)	9 (90.0%)	
Yes	8 (25.0%)	12 (9.9%)	1 (10.0%)	
**Multi-organ transplantation (N (%))**				0.20
Liver + kidney	1 (3.1%)	1 (0.8%)	0 (0.0%)	
Pancreas + kidney	0 (0.0%)	3 (2.5%)	0 (0.0%)	
Heart + kidney	0 (0.0%)	2 (1.7%)	1 (10.0%)	
None	31 (96.9%)	113 (93.4%)	8 (80.0%)	

Liver +Heart+Kidney	0 (0.0%)	2 (1.7%)	1 (10.0%)	
**CMV risk categories (N (%))**				0.93
Low	5 (15.6%)	27 (22.3%)	2 (20.0%)	
Intermediate	16 (50.0%)	56 (46.3%)	4 (40.0%)	
High	11 (34.4%)	36 (29.8%)	4 (40.0%)	
Unknown	0 (0.0%)	2 (1.7%)	0 (0.0%)	
**Was the donor organ pumped? (N (%))**				0.11
No	7 (25.0%)	45 (39.5%)	1 (11.1%)	
Yes	21 (75.0%)	69 (60.5%)	8 (88.9%)	
**Transplantation characteristics**				
**Cold ischemic time (hours), median (IQR)**	13.4 (9.5-20.2)	12.5 (3.3-21.2)	18.0 (14.8-21.8)	0.25
**cPRA (%), median (IQR)**	0 (0-59)	0 (0-0)	0 (0-32)	0.37
**Donors' characteristics**				
**Age (years), mean (SD)**	35.6 (15.0)	39.9 (16.4)	45.2 (11.0)	0.20
**Sex (N (%))**				0.18
male	13 (40.6%)	71 (58.7%)	5 (50.0%)	
female	19 (59.4%)	50 (41.3%)	5 (50.0%)	
**Race/ethnicity**				0.006
White	23 (71.9%)	96 (79.3%)	4 (40.0%)	
African American	2 (6.2%)	6 (5.0%)	0 (0.0%)	
Asian	1 (3.1%)	1 (0.8%)	2 (20.0%)	
Other	1 (3.1%)	3 (2.5%)	0 (0.0%)	
Unknown	5 (15.6%)	15 (12.4%)	4 (40.0%)	
**Donor type (N (%))**				0.40
Living	10 (31.2%)	47 (38.8%)	2 (20.0%)	
Deceased	22 (68.8%)	74 (61.2%)	8 (80.0%)	
**Donor DCD (N (%))**				0.80
No	14 (64%)	44 (59%)	4 (50%)	
Yes	8 (36%)	30 (41%)	4 (50%)	
**KDPI, median (IQR)**	25 (4-41)	35 (16-61)	30 (16-77)	0.14
**Donor cause of death (N (%))**				0.91
Anoxia	10 (45%)	35 (47%)	3 (38%)	
Cerebrovascular/stroke	2 (9%)	11 (15%)	2 (25%)	
Head trauma	8 (36%)	26 (35%)	3 (38%)	
Central nervous system tumor	1 (5%)	1 (1%)	0 (0%)	

Other	1 (5%)	1 (1%)	0 (0%)	
**Donor terminal creatinine (mg/dl), median (IQR)**	0.80 (0.60-0.95)	0.81 (0.64-1.0)	0.83 (0.6-1.05)	0.92
**Donor diabetes (N (%))**				0.043
No	27 (84.4%)	113 (95.8%)	10 (100.0%)	
Yes	5 (15.6%)	5 (4.2%)	0 (0.0%)	
**Donor hypertension (N (%))**				0.15
No	30 (93.8%)	97 (84.3%)	7 (70.0%)	
Yes	2 (6.2%)	18 (15.7%)	3 (30.0%)	
**Donor malignancy (N (%))**				0.41
No	32 (100.0%)	116 (95.9%)	10 (100.0%)	
Yes	0 (0.0%)	5 (4.1%)	0 (0.0%)	
**Immunological characteristics**				
**Number of HLA mismatches (HLA A, B and DR) (N (%))**				0.001
1	4 (12.5%)	2 (1.7%)	0 (0.0%)	
2	5 (15.6%)	6 (5.0%)	0 (0.0%)	
3	5 (15.6%)	14 (11.6%)	1 (10.0%)	
4	12 (37.5%)	28 (23.1%)	0 (0.0%)	
5	4 (12.5%)	40 (33.1%)	5 (50.0%)	
6	2 (6.2%)	31 (25.6%)	4 (40.0%)	
**HLA mismatches A (N (%))**				0.23
0	0 (0%)	3 (16%)	0 (0%)	
1	8 (73%)	6 (32%)	1 (50%)	
2	3 (27%)	10 (53%)	1 (50%)	
**HLA mismatches B (N (%))**				0.68
0	0 (0%)	2 (11%)	0 (0%)	
1	3 (27%)	5 (26%)	0 (0%)	
2	8 (73%)	12 (63%)	2 (100%)	
**HLA mismatches DR (N (%))**				<0.001
0	9 (43%)	1 (2%)	0 (0%)	
1	8 (38%)	30 (52%)	1 (14%)	
2	4 (19%)	27 (47%)	6 (86%)	
**HLA mismatches DQ (N (%))**				0.002

0	7 (64%)	0 (0%)	0 (0%)	
1	3 (27%)	13 (68%)	1 (50%)	
2	1 (9%)	6 (32%)	1 (50%)	
**Current flow B cell (N (%))**				0.94
< 2 SD compatible	19 (90%)	58 (94%)	7 (100%)	
2-3 SD compatible	1 (5%)	2 (3%)	0 (0%)	
weak positive	1 (5%)	1 (2%)	0 (0%)	
positive	0 (0%)	1 (2%)	0 (0%)	
**Current flow T cell (N (%))**				0.54
< 2 SD compatible	18 (86%)	58 (94%)	6 (86%)	
2-3 SD compatible	2 (10%)	3 (5%)	1 (14%)	
weak positive	0 (0%)	1 (2%)	0 (0%)	
positive	1 (5%)	0 (0%)	0 (0%)	
**Induction treatment (N (%))**				
No Induction	0 (0%)	0 (0%)	0 (0%)	NA
Basiliximab	4 (12.5%)	16 (13.2%)	1 (10.0%)	0.96
Thymoglobulin	26 (81.2%)	104 (86.0%)	9 (90.0%)	0.73
Steroid	32 (100.0%)	121 (100.0%)	10 (100.0%)	NA
Alemtuzumab	1 (3.1%)	2 (1.7%)	0 (0.0%)	0.78
**Outcomes**				
**Proportion of Antibody Mediated Rejection of the subset of patients who underwent biopsy (N (%))**				0.069
No	27 (84.4%)	82 (67.8%)	5 (50.0%)	
Yes	5 (15.6%)	39 (32.2%)	5 (50.0%)	
**Proportion of rejection of the subset of patients who underwent biopsy (N (%))**				0.14
No	23 (71.9%)	64 (52.9%)	5 (50.0%)	
Yes	9 (28.1%)	57 (47.1%)	5 (50.0%)	
**Proportion of T-cell Mediated Rejection of the subset of patients who underwent biopsy (N (%))**				0.17
No	25 (78.1%)	73 (60.3%)	6 (60.0%)	

Yes	7 (21.9%)	48 (39.7%)	4 (40.0%)	
**Post-Transplant de novo Donor Specific Antibodies (N (%))**				0.083
No	26 (81.2%)	76 (62.8%)	5 (50.0%)	
Yes	6 (18.8%)	45 (37.2%)	5 (50.0%)	
**Dd-cf-DNA elevation (N (%))**				0.032
No	19 (83%)	49 (53%)	5 (62%)	
Yes	4 (17%)	44 (47%)	3 (38%)	
**Delayed Graft Function (N (%))**				0.28
No	29 (90.6%)	101 (83.5%)	7 (70.0%)	
Yes	3 (9.4%)	20 (16.5%)	3 (30.0%)	
**Death (N (%))**				0.71
No	31 (96.9%)	115 (95.0%)	10 (100.0%)	
Yes	1 (3.1%)	6 (5.0%)	0 (0.0%)	
**Graft Loss (N (%))**				0.19
No	32 (100.0%)	112 (92.6%)	10 (100.0%)	
Yes	0 (0.0%)	9 (7.4%)	0 (0.0%)	

Values are expressed as mean (standard deviation), median (interquartile range), or number (%). Continuous variables were compared via t-tests or Mann-Whitney U tests. Categorical variables were compared via Chi-square tests.

Table S32: Patient characteristics for the rejection cohort based on eplet mismatch load for HLA-DQB1 locus

	Zero Eplet Mismatch Group	Low Eplet Mismatch Group	High Eplet Mismatch Group	p-value
**N**	30	162	44	
**<u>Recipients' characteristics</u>**				
**Age (years), mean (SD)**	47.3 (15.0)	48.9 (15.1)	49.9 (14.7)	0.76
**Sex (N (%))**				0.32
male	16 (53.3%)	102 (63.0%)	31 (70.5%)	
female	14 (46.7%)	60 (37.0%)	13 (29.5%)	
**Race/ethnicity (N (%))**				0.13
White	19 (63.3%)	107 (71.3%)	25 (59.5%)	
Hispanic	11 (36.7%)	25 (16.7%)	9 (21.4%)	
African American	0 (0.0%)	3 (2.0%)	2 (4.8%)	
Asian	0 (0.0%)	8 (5.3%)	2 (4.8%)	
Other	0 (0.0%)	7 (4.7%)	4 (9.5%)	
Unknown	0 (0.0%)	0 (0.0%)	0 (0.0%)	
**Body Mass Index (kg/m2), mean (SD)**	28.3 (6.6)	29.3 (6.1)	29.1 (5.6)	0.75
**Dialysis vintage (months), median (IQR)**	32.6 (11.4-57.7)	34.7 (15.4-57.3)	41.8 (29.9-60.3)	0.22
**Cause of kidney failure (N (%))**				0.74
Diabetes mellitus	8 (26.7%)	53 (32.7%)	8 (18.2%)	
Hypertension/Vascular disease	4 (13.3%)	18 (11.1%)	6 (13.6%)	
Cystic kidney disease	1 (3.4%)	10 (6.2%)	4 (9.1%)	
Glomerulonephritis	7 (23.3%)	41 (25.3%)	14 (31.8%)	
Other/unknown	10 (33.3%)	40 (24.7%)	12 (27.3%)	
**Prior kidney transplant (N (%))**				0.036
No	22 (73.3%)	145 (89.5%)	40 (90.9%)	
Yes	8 (26.7%)	17 (10.5%)	4 (9.1%)	
**Multi-organ transplantation (N (%))**				0.72
Liver + kidney	1 (3.3%)	1 (0.6%)	1 (2.3%)	
Pancreas + kidney	0 (0.0%)	3 (1.9%)	0 (0.0%)	
Heart + kidney	0 (0.0%)	3 (1.9%)	0 (0.0%)	
None	29 (96.7%)	153 (94.4%)	42 (95.5%)	

Liver +Heart+Kidney	0 (0.0%)	2 (1.2%)	1 (2.3%)	
**CMV risk categories (N (%))**				0.75
Low	4 (13.3%)	42 (25.9%)	10 (22.7%)	
Intermediate	14 (46.7%)	67 (41.4%)	17 (38.6%)	
High	12 (40.0%)	50 (30.9%)	16 (36.4%)	
Unknown	0 (0.0%)	3 (1.9%)	1 (2.3%)	
**Was the donor organ pumped? (N (%))**				0.92
No	6 (22.2%)	39 (25.3%)	10 (23.3%)	
Yes	21 (77.8%)	115 (74.7%)	33 (76.7%)	
**En block kidney (N (%))**	0 (0%)	5 (21%)	2 (25%)	0.33
**Dual kidney (N (%))**	0 (0%)	4 (17%)	1 (12%)	0.47
**Transplantation characteristics**				
**Cold ischemic time (hours), median (IQR)**	17.7 (9.5-22.4)	15.5 (5.8-21.0)	12.9 (7.4-19.6)	0.35
**cPRA (%), median (IQR)**	0 (0-98)	0 (0-0)	0 (0-0)	0.004
**Donors' characteristics**				
**Age (years), mean (SD)**	43.7 (14.7)	37.7 (16.9)	38.6 (14.2)	0.17
**Sex (N (%))**				0.86
male	16 (53.3%)	95 (58.6%)	25 (56.8%)	
female	14 (46.7%)	67 (41.4%)	19 (43.2%)	
**Race/ethnicity**				0.35
White	21 (70.0%)	134 (82.7%)	35 (79.5%)	
African American	2 (6.7%)	6 (3.7%)	0 (0.0%)	
Asian	0 (0.0%)	4 (2.5%)	2 (4.5%)	
Other	1 (3.3%)	3 (1.9%)	0 (0.0%)	
Unknown	6 (20.0%)	15 (9.3%)	7 (15.9%)	
**Donor type (N (%))**				0.78
Living	9 (30.0%)	42 (25.9%)	10 (22.7%)	
Deceased	21 (70.0%)	120 (74.1%)	34 (77.3%)	
**Donor DCD (N (%))**				0.86
No	12 (57.1%)	65 (54.2%)	17 (50.0%)	
Yes	9 (42.9%)	55 (45.8%)	17 (50.0%)	
**KDPI, median (IQR)**	42 (25-53)	33 (15-60)	32 (21-58)	0.65
**Donor cause of death (N (%))**				0.58
Anoxia	8 (38.1%)	66 (55.0%)	16 (47.1%)	
Cerebrovascular/stroke	5 (23.8%)	15 (12.5%)	6 (17.6%)	
Head trauma	7 (33.3%)	36 (30.0%)	11 (32.4%)	

Central nervous system tumor	1 (4.8%)	1 (0.8%)	0 (0.0%)	
Other	0 (0.0%)	2 (1.7%)	1 (2.9%)	
**Donor terminal creatinine (mg/dl), median (IQR)**	0.83 (0.70-1.05)	0.80 (0.61-1.03)	0.91 (0.77-1.12)	0.13
**Donor diabetes (N (%))**				0.25
No	26 (86.7%)	149 (94.3%)	42 (95.5%)	
Yes	4 (13.3%)	9 (5.7%)	2 (4.5%)	
**Donor hypertension (N (%))**				0.98
No	24 (80.0%)	128 (81.5%)	35 (81.4%)	
Yes	6 (20.0%)	29 (18.5%)	8 (18.6%)	
**Donor malignancy (N (%))**				0.30
No	28 (93.3%)	159 (98.1%)	43 (97.7%)	
Yes	2 (6.7%)	3 (1.9%)	1 (2.3%)	
**Immunological characteristics**				
**Number of HLA mismatches (HLA A, B and DR) (N (%))**				<0.001
1	5 (16.7%)	1 (0.6%)	0 (0.0%)	
2	5 (16.7%)	6 (3.7%)	2 (4.5%)	
3	9 (30.0%)	29 (17.9%)	2 (4.5%)	
4	8 (26.7%)	39 (24.1%)	9 (20.5%)	
5	2 (6.7%)	62 (38.3%)	13 (29.5%)	
6	1 (3.3%)	25 (15.4%)	18 (40.9%)	
**HLA mismatches A (N (%))**				0.30
0	0 (0%)	3 (11%)	1 (14%)	
1	7 (64%)	12 (43%)	1 (14%)	
2	4 (36%)	13 (46%)	5 (71%)	
**HLA mismatches B (N (%))**				0.57
0	0 (0%)	2 (7%)	1 (14%)	
1	4 (36%)	5 (18%)	2 (29%)	
2	7 (64%)	21 (75%)	4 (57%)	
**HLA mismatches DR (N (%))**				<0.001
0	12 (63%)	4 (6%)	0 (0%)	
1	4 (21%)	32 (50%)	7 (30%)	
2	3 (16%)	28 (44%)	16 (70%)	

**HLA mismatches DQ (N (%))**				<0.001
0	11 (100%)	1 (4%)	0 (0%)	
1	0 (0%)	20 (71%)	2 (29%)	
2	0 (0%)	7 (25%)	5 (71%)	
**Current flow B cell (N (%))**				0.44
< 2 SD compatible	17 (89%)	61 (90%)	26 (100%)	
2-3 SD compatible	0 (0%)	4 (6%)	0 (0%)	
weak positive	1 (5%)	1 (1%)	0 (0%)	
positive	1 (5%)	2 (3%)	0 (0%)	
**Current flow T cell (N (%))**				0.59
< 2 SD compatible	17 (89%)	61 (90%)	26 (100%)	
2-3 SD compatible	2 (11%)	4 (6%)	0 (0%)	
weak positive	0 (0%)	1 (1%)	0 (0%)	
positive	0 (0%)	2 (3%)	0 (0%)	
**Induction treatment (N (%))**				
No Induction	0 (0%)	0 (0%)	0 (0%)	NA
Basiliximab	3 (10.0%)	19 (11.7%)	6 (13.6%)	0.89
Thymoglobulin	24 (80.0%)	140 (86.4%)	39 (88.6%)	0.56
Steroid	30 (100.0%)	162 (100.0%)	44 (100.0%)	NA
Alemtuzumab	2 (6.7%)	3 (1.9%)	0 (0.0%)	0.14
**Outcomes**				
**Proportion of Antibody Mediated Rejection of the subset of patients who underwent biopsy (N (%))**				0.19
No	26 (86.7%)	119 (73.5%)	30 (68.2%)	
Yes	4 (13.3%)	43 (26.5%)	14 (31.8%)	
**Proportion of rejection of the subset of patients who underwent biopsy (N subset of patients who underwent biopsy (N (%))**				0.019
No	24 (80.0%)	92 (56.8%)	21 (47.7%)	
Yes	6 (20.0%)	70 (43.2%)	23 (52.3%)	
**Proportion of T-cell Mediated Rejection of the subset of patients who**				0.038

**underwent biopsy (N subset of patients who underwent biopsy (N (%))**				
No	25 (83.3%)	105 (64.8%)	24 (54.5%)	
Yes	5 (16.7%)	57 (35.2%)	20 (45.5%)	
**Post-Transplant de novo Donor Specific Antibodies (N (%))**				0.057
No	25 (83.3%)	110 (67.9%)	25 (56.8%)	
Yes	5 (16.7%)	52 (32.1%)	19 (43.2%)	
**Dd-cf-DNA elevation (N (%))**				0.044
No	18 (85.7%)	72 (59.0%)	19 (54.3%)	
Yes	3 (14.3%)	50 (41.0%)	16 (45.7%)	
**Delayed Graft Function (N (%))**				0.76
No	26 (86.7%)	134 (82.7%)	38 (86.4%)	
Yes	4 (13.3%)	28 (17.3%)	6 (13.6%)	
**Death (N (%))**				0.80
No	29 (96.7%)	152 (93.8%)	41 (93.2%)	
Yes	1 (3.3%)	10 (6.2%)	3 (6.8%)	
**Graft Loss (N (%))**				0.71
No	29 (96.7%)	154 (95.1%)	43 (97.7%)	
Yes	1 (3.3%)	8 (4.9%)	1 (2.3%)	

Values are expressed as mean (standard deviation), median (interquartile range), or number (%). Continuous variables were compared via t-tests or Mann-Whitney U tests. Categorical variables were compared via Chi-square tests.

Table S33: Patient characteristics for the rejection cohort based on eplet mismatch load for HLA-DRB1 locus

	Zero Eplet Mismatch Group	Low Eplet Mismatch Group	High Eplet Mismatch Group	p-value
**N**	13	144	69	
**<u>Recipients' characteristics</u>**				
**Age (years), mean (SD)**	47.5 (17.7)	48.4 (14.7)	50.4 (15.3)	0.61
**Sex (N (%))**				0.84
male	8 (61.5%)	88 (61.1%)	45 (65.2%)	
female	5 (38.5%)	56 (38.9%)	24 (34.8%)	
**Race/ethnicity (N (%))**				0.33
White	10 (76.9%)	92 (66.7%)	42 (68.9%)	
Hispanic	3 (23.1%)	32 (23.2%)	7 (11.5%)	
African American	0 (0.0%)	2 (1.4%)	3 (4.9%)	
Asian	0 (0.0%)	5 (3.6%)	5 (8.2%)	
Other	0 (0.0%)	7 (5.1%)	4 (6.6%)	
Unknown	0 (0.0%)	0 (0.0%)	0 (0.0%)	
**Body Mass Index (kg/m2), mean (SD)**	28.3 (7.0)	28.7 (5.7)	29.9 (6.9)	0.34
**Dialysis vintage (months), median (IQR)**	28.8 (14.4-36.0)	36.7 (16.1-65.5)	35.1 (24.0-55.2)	0.465
**Cause of kidney failure (N (%))**				0.621
Diabetes mellitus	3 (23.1%)	39 (27.1%)	23 (33.3%)	
Hypertension/Vascular disease	0 (0.0%)	18 (12.5%)	9 (13.1%)	
Cystic kidney disease	0 (0.0%)	11 (7.6%)	4 (5.8%)	
Glomerulonephritis	4 (30.8%)	39 (27.1%)	18 (26.1%)	
Other/unknown	6 (46.1%)	37 (25.7%)	15 (21.7%)	
**Prior kidney transplant (N (%))**				0.009
No	8 (61.5%)	124 (86.1%)	64 (92.8%)	
Yes	5 (38.5%)	20 (13.9%)	5 (7.2%)	
**Multi-organ transplantation (N (%))**				0.83
Liver + kidney	0 (0.0%)	2 (1.4%)	1 (1.4%)	
Pancreas + kidney	0 (0.0%)	1 (0.7%)	2 (2.9%)	
Heart + kidney	0 (0.0%)	2 (1.4%)	1 (1.4%)	
None	13 (100.0%)	138 (95.8%)	63 (91.3%)	

Liver +Heart+Kidney	0 (0.0%)	1 (0.7%)	2 (2.9%)	
**CMV risk categories (N (%))**				0.64
Low	2 (15.4%)	35 (24.3%)	17 (24.6%)	
Intermediate	5 (38.5%)	57 (39.6%)	32 (46.4%)	
High	6 (46.2%)	48 (33.3%)	20 (29.0%)	
Unknown	0 (0.0%)	4 (2.8%)	0 (0.0%)	
**Was the donor organ pumped? (N (%))**				0.70
No	4 (33.3%)	36 (26.5%)	15 (22.7%)	
Yes	8 (66.7%)	100 (73.5%)	51 (77.3%)	
**En block kidney (N (%))**	0 (0%)	5 (23%)	2 (20%)	0.50
**Dual kidney (N (%))**	0 (0%)	2 (9%)	1 (10%)	0.77
**Transplantation characteristics**				
**Cold ischemic time (hours), median (IQR)**	18.7 (9.5-21.6)	14.2 (5.9-20.2)	14.0 (7.8-21.8)	0.70
**cPRA (%), median (IQR)**	0 (0-98)	0 (0-10)	0 (0-0)	0.21
**Donors' characteristics**				
**Age (years), mean (SD)**	42.5 (16.1)	37.7 (16.4)	39.0 (15.8)	0.54
**Sex (N (%))**				0.67
male	6 (46.2%)	82 (56.9%)	41 (59.4%)	
female	7 (53.8%)	62 (43.1%)	28 (40.6%)	
**Race/ethnicity**				0.60
White	10 (76.9%)	118 (81.9%)	53 (76.8%)	
African American	0 (0.0%)	7 (4.9%)	2 (2.9%)	
Asian	0 (0.0%)	4 (2.8%)	2 (2.9%)	
Other	1 (7.7%)	2 (1.4%)	1 (1.4%)	
Unknown	2 (15.4%)	13 (9.0%)	11 (15.9%)	
**Donor type (N (%))**				0.59
Living	5 (38.5%)	39 (27.1%)	17 (24.6%)	
Deceased	8 (61.5%)	105 (72.9%)	52 (75.4%)	
**Donor DCD (N (%))**				0.27
No	6 (75.0%)	53 (50.5%)	31 (59.6%)	
Yes	2 (25.0%)	52 (49.5%)	21 (40.4%)	
**KDPI, median (IQR)**	34 (7-59)	31 (16-53)	36 (22-63)	0.38
**Donor cause of death (N (%))**				0.009
Anoxia	1 (12.5%)	57 (54.3%)	27 (51.9%)	
Cerebrovascular/stroke	4 (50.0%)	11 (10.5%)	8 (15.4%)	
Head trauma	2 (25.0%)	34 (32.4%)	16 (30.8%)	

Central nervous system tumor	1 (12.5%)	1 (1.0%)	0 (0.0%)	
Other	0 (0.0%)	2 (1.9%)	1 (1.9%)	
**Donor terminal creatinine (mg/dl), median (IQR)**	0.75 (0.68-0.90)	0.81 (0.63-1.03)	0.90 (0.69-1.12)	0.25
**Donor diabetes (N (%))**				0.49
No	13 (100.0%)	132 (93.6%)	62 (91.2%)	
Yes	0 (0.0%)	9 (6.4%)	6 (8.8%)	
**Donor hypertension (N (%))**				0.25
No	11 (84.6%)	120 (85.1%)	50 (75.8%)	
Yes	2 (15.4%)	21 (14.9%)	16 (24.2%)	
**Donor malignancy (N (%))**				0.71
No	13 (100.0%)	140 (97.2%)	68 (98.6%)	
Yes	0 (0.0%)	4 (2.8%)	1 (1.4%)	
**Immunological characteristics**				
**Number of HLA mismatches (HLA A, B and DR) (N (%))**				<0.001
1	5 (38.5%)	1 (0.7%)	0 (0.0%)	
2	1 (7.7%)	12 (8.3%)	0 (0.0%)	
3	3 (23.1%)	28 (19.4%)	5 (7.2%)	
4	4 (30.8%)	41 (28.5%)	11 (15.9%)	
5	0 (0.0%)	46 (31.9%)	25 (36.2%)	
6	0 (0.0%)	16 (11.1%)	28 (40.6%)	
**HLA mismatches A (N (%))**				0.36
0	0 (0%)	3 (12%)	1 (8%)	
1	3 (43%)	14 (54%)	3 (25%)	
2	4 (57%)	9 (35%)	8 (67%)	
**HLA mismatches B (N (%))**				0.37
0	0 (0%)	2 (8%)	0 (0%)	
1	2 (29%)	8 (31%)	1 (8%)	
2	5 (71%)	16 (62%)	11 (92%)	
**HLA mismatches DR (N (%))**				<0.001
0	9 (100%)	7 (11%)	0 (0%)	
1	0 (0%)	36 (56%)	6 (19%)	
2	0 (0%)	21 (33%)	25 (81%)	

**HLA mismatches DQ (N (%))**				<0.001
0	7 (100%)	5 (19%)	0 (0%)	
1	0 (0%)	18 (69%)	4 (33%)	
2	0 (0%)	3 (12%)	8 (67%)	
**Current flow B cell (N (%))**				0.042
< 2 SD compatible	9 (90%)	65 (94%)	28 (88%)	
2-3 SD compatible	0 (0%)	3 (4%)	2 (6%)	
weak positive	1 (10%)	0 (0%)	0 (0%)	
positive	0 (0%)	1 (1%)	2 (6%)	
**Current flow T cell (N (%))**				0.24
< 2 SD compatible	8 (80%)	65 (94%)	28 (88%)	
2-3 SD compatible	2 (20%)	1 (1%)	3 (9%)	
weak positive	0 (0%)	1 (1%)	0 (0%)	
positive	0 (0%)	2 (3%)	1 (3%)	
**Induction treatment (N (%))**				
No Induction	0 (0%)	0 (0%)	0 (0%)	NA
Basiliximab	2 (15.4%)	19 (13.2%)	6 (8.7%)	0.59
Thymoglobulin	10 (76.9%)	122 (84.7%)	62 (89.9%)	0.38
Steroid	13 (100.0%)	144 (100.0%)	69 (100.0%)	NA
Alemtuzumab	1 (7.7%)	3 (2.1%)	1 (1.4%)	0.37
**Outcomes**				
**Proportion of Antibody Mediated Rejection of the subset of patients who underwent biopsy (N subset of patients who underwent biopsy (N (%))**				0.12
No	11 (84.6%)	111 (77.1%)	45 (65.2%)	
Yes	2 (15.4%)	33 (22.9%)	24 (34.8%)	
**Proportion of rejection of the subset of patients who underwent biopsy (N subset of patients who underwent biopsy (N (%))**				0.017
No	11 (84.6%)	88 (61.1%)	32 (46.4%)	
Yes	2 (15.4%)	56 (38.9%)	37 (53.6%)	

**Proportion of T-cell Mediated Rejection of the subset of patients who underwent biopsy (N subset of patients who underwent biopsy (N (%))**				0.037
No	12 (92.3%)	96 (66.7%)	39 (56.5%)	
Yes	1 (7.7%)	48 (33.3%)	30 (43.5%)	
**Post-Transplant de novo Donor Specific Antibodies (N (%))**				0.34
No	11 (84.6%)	96 (66.7%)	44 (63.8%)	
Yes	2 (15.4%)	48 (33.3%)	25 (36.2%)	
**Dd-cf-DNA elevation (N (%))**				0.028
No	8 (100.0%)	66 (61.1%)	27 (50.9%)	
Yes	0 (0.0%)	42 (38.9%)	26 (49.1%)	
**Delayed Graft Function (N (%))**				0.99
No	11 (84.6%)	122 (84.7%)	58 (84.1%)	
Yes	2 (15.4%)	22 (15.3%)	11 (15.9%)	
**Death (N (%))**				0.25
No	13 (100.0%)	133 (92.4%)	67 (97.1%)	
Yes	0 (0.0%)	11 (7.6%)	2 (2.9%)	
**Graft Loss (N (%))**				0.33
No	13 (100.0%)	139 (96.5%)	64 (92.8%)	
Yes	0 (0.0%)	5 (3.5%)	5 (7.2%)	

Values are expressed as mean (standard deviation), median (interquartile range), or number (%). Continuous variables were compared via t-tests or Mann-Whitney U tests. Categorical variables were compared via Chi-square tests.

Table S34: Performance characteristics table of eplet mismatch load for HLA-A locus for rejection

Cutpoint	Sensitivity	Specificity	Overall % correctly classified	LR+	LR-
(>= 1)	100.00%	0.00%	40.93%	1	
(>= 2)	98.73%	0.88%	40.93%	0.9961	1.443
(>= 3)	96.20%	0.88%	39.90%	0.9705	4.3291
(>= 4)	94.94%	0.88%	39.38%	0.9578	5.7721
(>= 5)	93.67%	1.75%	39.38%	0.9534	3.6076
(>= 6)	88.61%	2.63%	37.82%	0.91	4.3291
(>= 7)	83.54%	7.89%	38.86%	0.9071	2.0844
(>= 8)	78.48%	10.53%	38.34%	0.8771	2.0443
(>= 9)	73.42%	19.30%	41.45%	0.9097	1.3774
(>= 10)	69.62%	25.44%	43.52%	0.9337	1.1942
(>= 11)	65.82%	30.70%	45.08%	0.9498	1.1132
(>= 12)	60.76%	33.33%	44.56%	0.9114	1.1772
(>= 13)	55.70%	37.72%	45.08%	0.8943	1.1746
(>= 14)	48.10%	40.35%	43.52%	0.8064	1.2862
(>= 15)	44.30%	45.61%	45.08%	0.8146	1.221
(>= 16)	34.18%	49.12%	43.01%	0.6718	1.34
(>= 17)	31.65%	57.89%	47.15%	0.7516	1.1807
(>= 18)	31.65%	61.40%	49.22%	0.8199	1.1132
(>= 19)	29.11%	63.16%	49.22%	0.7902	1.1224
(>= 20)	27.85%	66.67%	50.78%	0.8354	1.0823
(>= 21)	26.58%	74.56%	54.92%	1.045	0.9847
(>= 22)	26.58%	78.07%	56.99%	1.2122	0.9404
(>= 23)	22.78%	79.82%	56.48%	1.1293	0.9673
(>= 24)	20.25%	80.70%	55.96%	1.0495	0.9882
(>= 25)	17.72%	83.33%	56.48%	1.0633	0.9873
(>= 26)	17.72%	84.21%	56.99%	1.1224	0.9771
(>= 27)	17.72%	88.60%	59.59%	1.554	0.9287
(>= 28)	13.92%	88.60%	58.03%	1.221	0.9716
(>= 29)	12.66%	90.35%	58.55%	1.3119	0.9667
(>= 30)	11.39%	91.23%	58.55%	1.2987	0.9713
(>= 31)	7.59%	91.23%	56.99%	0.8658	1.0129
(>= 32)	6.33%	92.11%	56.99%	0.8017	1.017
(>= 33)	6.33%	92.98%	57.51%	0.9019	1.0074
(>= 34)	6.33%	94.74%	58.55%	1.2025	0.9887

(>= 35)	5.06%	98.25%	60.10%	2.8861	0.9663
(>= 37)	2.53%	99.12%	59.59%	2.8861	0.9833
(>= 38)	1.27%	99.12%	59.07%	1.443	0.9961
(>= 48)	1.27%	100.00%	59.59%	0.9873	
(> 48)	0.00%	100.00%	59.07%	1	

LR: Likelihood rati**o**

Table S35: Performance characteristics table of eplet mismatch load for HLA-B locus for rejection

Cutpoint	Sensitivity	Specificity	Overall % correctly classified	LR+	LR-
(>= 1)	100.00%	0.00%	40.31%	1	
(>= 2)	100.00%	1.71%	41.33%	1.0174	0
(>= 3)	98.73%	1.71%	40.82%	1.0045	0.7405
(>= 4)	97.47%	3.42%	41.33%	1.0092	0.7405
(>= 5)	94.94%	5.13%	41.33%	1.0007	0.9873
(>= 6)	91.14%	5.98%	40.31%	0.9694	1.481
(>= 7)	91.14%	10.26%	42.86%	1.0156	0.8639
(>= 8)	88.61%	14.53%	44.39%	1.0367	0.7841
(>= 9)	83.54%	21.37%	46.43%	1.0625	0.7701
(>= 10)	79.75%	23.93%	46.43%	1.0484	0.8463
(>= 11)	75.95%	28.21%	47.45%	1.0579	0.8527
(>= 12)	69.62%	35.04%	48.98%	1.0718	0.8669
(>= 13)	62.03%	41.03%	49.49%	1.0517	0.9256
(>= 14)	56.96%	49.57%	52.55%	1.1296	0.8682
(>= 15)	49.37%	53.85%	52.04%	1.0696	0.9403
(>= 16)	44.30%	62.39%	55.10%	1.1781	0.8927
(>= 17)	35.44%	67.52%	54.59%	1.0913	0.9561
(>= 18)	32.91%	68.38%	54.08%	1.0407	0.9812
(>= 19)	24.05%	74.36%	54.08%	0.938	1.0214
(>= 20)	18.99%	78.63%	54.59%	0.8886	1.0303
(>= 21)	17.72%	82.05%	56.12%	0.9873	1.0028
(>= 22)	13.92%	88.03%	58.16%	1.1637	0.9778
(>= 23)	11.39%	88.03%	57.14%	0.9521	1.0065
(>= 24)	10.13%	91.45%	58.67%	1.1848	0.9827
(>= 25)	10.13%	92.31%	59.18%	1.3165	0.9736
(>= 27)	7.59%	94.87%	59.69%	1.481	0.974
(>= 28)	6.33%	96.58%	60.20%	1.8513	0.9699
(>= 29)	6.33%	97.44%	60.71%	2.4684	0.9614
(>= 30)	6.33%	98.29%	61.22%	3.7025	0.953
(>= 31)	5.06%	99.15%	61.22%	5.9241	0.9576
(>= 32)	3.80%	99.15%	60.71%	4.4431	0.9703
(>= 33)	2.53%	99.15%	60.20%	2.962	0.9831
(>= 35)	1.27%	99.15%	59.69%	1.481	0.9959
(>= 36)	0.00%	99.15%	59.18%	0	1.0086

(> 36)	0.00%	100.00%	59.69%	1	

LR: Likelihood ratio

Table S36: Performance characteristics table of eplet mismatch load for HLA-C locus for rejection

Cutpoint	Sensitivity	Specificity	Overall % correctly classified	LR+	LR-
(>= 1)	100.00%	0.00%	42.39%	1	
(>= 2)	98.72%	4.72%	44.57%	1.036	0.2718
(>= 3)	97.44%	8.49%	46.20%	1.0648	0.302
(>= 4)	92.31%	12.26%	46.20%	1.0521	0.6272
(>= 5)	85.90%	17.92%	46.74%	1.0466	0.7868
(>= 6)	82.05%	26.42%	50.00%	1.1151	0.6795
(>= 7)	73.08%	33.96%	50.54%	1.1066	0.7927
(>= 8)	66.67%	43.40%	53.26%	1.1778	0.7681
(>= 9)	61.54%	50.00%	54.89%	1.2308	0.7692
(>= 10)	56.41%	55.66%	55.98%	1.2722	0.7831
(>= 11)	50.00%	61.32%	56.52%	1.2927	0.8154
(>= 12)	41.03%	66.98%	55.98%	1.2425	0.8805
(>= 13)	29.49%	71.70%	53.80%	1.0419	0.9835
(>= 14)	23.08%	75.47%	53.26%	0.9408	1.0192
(>= 15)	16.67%	77.36%	51.63%	0.7361	1.0772
(>= 16)	14.10%	80.19%	52.17%	0.7118	1.0712
(>= 17)	11.54%	83.96%	53.26%	0.7195	1.0536
(>= 18)	10.26%	86.79%	54.35%	0.7766	1.034
(>= 19)	10.26%	90.57%	56.52%	1.0872	0.9909
(>= 20)	8.97%	92.45%	57.07%	1.1891	0.9846
(>= 21)	7.69%	94.34%	57.61%	1.359	0.9785
(>= 22)	5.13%	95.28%	57.07%	1.0872	0.9957
(>= 23)	3.85%	96.23%	57.07%	1.0192	0.9992
(>= 24)	3.85%	99.06%	58.70%	4.0769	0.9707
(>= 27)	2.56%	100.00%	58.70%	0.9744	
(>= 29)	1.28%	100.00%	58.15%	0.9872	
(> 29)	0.00%	100.00%	57.61%	1	

LR: Likelihood ratio

Table S37: Performance characteristics table of eplet mismatch load for HLA-DQA1 locus for rejection

Cutpoint	Sensitivity	Specificity	Overall % correctly classified	LR+	LR-
(>= 1)	100.00%	0.00%	47.33%	1	
(>= 2)	93.55%	8.70%	48.85%	1.0246	0.7419
(>= 3)	80.65%	18.84%	48.09%	0.9937	1.0273
(>= 4)	58.06%	40.58%	48.85%	0.9772	1.0334
(>= 5)	46.77%	52.17%	49.62%	0.978	1.0202
(>= 6)	30.65%	68.12%	50.38%	0.9611	1.0182
(>= 7)	22.58%	75.36%	50.38%	0.9165	1.0273
(>= 8)	9.68%	84.06%	48.85%	0.607	1.0745
(>= 9)	8.06%	88.41%	50.38%	0.6956	1.0399
(>= 10)	8.06%	91.30%	51.91%	0.9274	1.0069
(>= 11)	8.06%	92.75%	52.67%	1.1129	0.9912
(>= 12)	6.45%	92.75%	51.91%	0.8903	1.0086
(>= 13)	3.23%	95.65%	51.91%	0.7419	1.0117
(>= 15)	0.00%	98.55%	51.91%	0	1.0147
(> 15)	0.00%	100.00%	52.67%	1	

LR: Likelihood ratio

Table S38: Performance characteristics table of eplet mismatch load for HLA-DQB1 locus for rejection

Cutpoint	Sensitivity	Specificity	Overall % correctly classified	LR+	LR-
(>= 1)	100.00%	0.00%	45.15%	1	
(>= 2)	97.85%	3.54%	46.12%	1.0144	0.6075
(>= 3)	95.70%	8.85%	48.06%	1.0499	0.486
(>= 4)	89.25%	14.16%	48.06%	1.0397	0.7594
(>= 5)	80.65%	17.70%	46.12%	0.9799	1.0935
(>= 6)	73.12%	23.01%	45.63%	0.9497	1.1683
(>= 7)	65.59%	36.28%	49.51%	1.0294	0.9483
(>= 8)	59.14%	41.59%	49.51%	1.0125	0.9824
(>= 9)	53.76%	47.79%	50.49%	1.0297	0.9675
(>= 10)	49.46%	52.21%	50.97%	1.035	0.9679
(>= 11)	40.86%	58.41%	50.49%	0.9824	1.0125
(>= 12)	37.63%	66.37%	53.40%	1.1191	0.9396
(>= 13)	35.48%	69.03%	53.88%	1.1456	0.9347
(>= 14)	30.11%	73.45%	53.88%	1.1341	0.9515
(>= 15)	26.88%	78.76%	55.34%	1.2657	0.9284
(>= 16)	24.73%	81.42%	55.83%	1.3308	0.9245
(>= 17)	20.43%	83.19%	54.85%	1.2151	0.9565
(>= 18)	13.98%	85.84%	53.40%	0.9872	1.0021
(>= 19)	10.75%	85.84%	51.94%	0.7594	1.0397
(>= 20)	8.60%	86.73%	51.46%	0.648	1.0539
(>= 21)	8.60%	89.38%	52.91%	0.81	1.0226
(>= 22)	6.45%	91.15%	52.91%	0.729	1.0263
(>= 23)	4.30%	93.81%	53.40%	0.6943	1.0202
(>= 24)	3.23%	94.69%	53.40%	0.6075	1.022
(>= 25)	1.08%	95.58%	52.91%	0.243	1.035
(>= 27)	1.08%	96.46%	53.40%	0.3038	1.0255
(>= 28)	0.00%	96.46%	52.91%	0	1.0367
(>= 30)	0.00%	98.23%	53.88%	0	1.018
(>= 31)	0.00%	99.12%	54.37%	0	1.0089
(> 31)	0.00%	100.00%	54.85%	1	

LR: Likelihood ratio

Table S39: Performance characteristics table of eplet mismatch load for HLA-DRB1 locus for rejection

Cutpoint	Sensitivity	Specificity	Overall % correctly classified	LR+	LR-
(>= 1)	100.00%	0.00%	43.66%	1	
(>= 2)	98.92%	3.33%	45.07%	1.0234	0.3226
(>= 3)	97.85%	4.17%	45.07%	1.021	0.5161
(>= 4)	95.70%	5.00%	44.60%	1.0074	0.8602
(>= 6)	95.70%	6.67%	45.54%	1.0253	0.6452
(>= 7)	90.32%	7.50%	43.66%	0.9765	1.2903
(>= 8)	88.17%	10.83%	44.60%	0.9888	1.0918
(>= 9)	86.02%	14.17%	45.54%	1.0022	0.9867
(>= 10)	79.57%	20.00%	46.01%	0.9946	1.0215
(>= 11)	73.12%	21.67%	44.13%	0.9334	1.2407
(>= 12)	68.82%	29.17%	46.48%	0.9715	1.0691
(>= 13)	65.59%	33.33%	47.42%	0.9839	1.0323
(>= 14)	62.37%	40.00%	49.77%	1.0394	0.9409
(>= 15)	60.22%	48.33%	53.52%	1.1655	0.8231
(>= 16)	54.84%	54.17%	54.46%	1.1965	0.8337
(>= 17)	51.61%	60.83%	56.81%	1.3178	0.7954
(>= 18)	48.39%	63.33%	56.81%	1.3196	0.8149
(>= 19)	41.94%	67.50%	56.34%	1.2903	0.8602
(>= 20)	39.78%	73.33%	58.69%	1.4919	0.8211
(>= 21)	34.41%	75.00%	57.28%	1.3763	0.8746
(>= 22)	31.18%	79.17%	58.22%	1.4968	0.8693
(>= 23)	30.11%	81.67%	59.15%	1.6422	0.8558
(>= 24)	25.81%	83.33%	58.22%	1.5484	0.8903
(>= 25)	24.73%	86.67%	59.62%	1.8548	0.8685
(>= 26)	21.51%	88.33%	59.15%	1.8433	0.8886
(>= 27)	18.28%	90.83%	59.15%	1.9941	0.8997
(>= 28)	17.20%	92.50%	59.62%	2.2939	0.8951
(>= 29)	11.83%	93.33%	57.75%	1.7742	0.9447
(>= 30)	7.53%	93.33%	55.87%	1.129	0.9908
(>= 31)	5.38%	95.00%	55.87%	1.0753	0.996
(>= 33)	5.38%	95.83%	56.34%	1.2903	0.9874
(>= 35)	4.30%	97.50%	56.81%	1.7204	0.9815
(>= 37)	3.23%	97.50%	56.34%	1.2903	0.9926
(>= 41)	3.23%	98.33%	56.81%	1.9355	0.9841

(>= 42)	2.15%	98.33%	56.34%	1.2903	0.9951
(>= 48)	1.08%	99.17%	56.34%	1.2903	0.9976
(>= 52)	0.00%	99.17%	55.87%	0	1.0084
(> 52)	0.00%	100.00%	56.34%	1	

LR: Likelihood ratio

Table S40: Association of class I HLA Eplet mismatch load and rejection using Cox proportional regression models

Model		Hazard Ratio (HR)	95% Confidence Interval (CI) of HR	p-value		Hazard Ratio (HR)	95% Confidence Interval (CI) of HR	p-value		Hazard Ratio (HR)	95% Confidence Interval (CI) of HR	p-value
UNADJUSTED	**N=205**				**N=201**				**N=203**			
	**HLA-A** (+1 eplet increase)	1.006	0.986-1.026	0.558	**HLA-B** (+1 eplet increase)	1.001	0.970-1.033	0.929	**HLA-C** (+1 eplet increase)	1.004	0.971-1.037	0.828
	**HLA-A** (ref=Zero)				**HLA-B** (ref=Zero)				**HLA-C** (ref=Zero)			
	**Low**	1.401	0.562-3.490	0.470	**Low**	0.457	0.163-1.280	0.136	**Low**	1.062	0.470-2.399	0.884
	**High**	1.341	0.481-3.7420	0.575	**High**	0.565	0.200-1.601	0.283	**High**	1.248	0.560-2.782	0.588
ADJUSTED*	**N=192**				**N=188**				**N=192**			
	**HLA-A** (+1 eplet increase)	1.011	0.983-1.039	0.448	**HLA-B** (+1 eplet increase)	1.026	0.987-1.067	0.200	**HLA-C** (+1 eplet increase)	1.020	0.980-1.062	0.328
	**HLA-A** (ref=Zero)				**HLA-B** (ref=Zero)				**HLA-C** (ref=Zero)			
	**Low**	1.845	0.590-5.767	0.292	**Low**	0.346	0.098-1.223	0.099	**Low**	1.511	0.544-4.197	0.428
	**High**	1.706	0.461-6.320	0.424	**High**	0.600	0.166-2.165	0.435	**High**	2.033	0.747-5.534	0.165

*adjusted for: recipient age, recipient sex, recipient race/ethnicity, recipient BMI, dialysis vintage, cause of end stage renal disease, preemptive kidney transplantation, history of prior kidney transplantation, multi-organ transplantation, type of induction therapy, donor age, donor sex, donor race/ethnicity, donor type [living vs. deceased], cold ischemic time, cytomegalovirus risk status, cPRA at allocation

Table S41: Association of class II HLA Eplet mismatch load and rejection using Cox proportional regression models

Model		Hazard Ratio (HR)	95% Confidence Interval (CI) of HR	p-value		Hazard Ratio (HR)	95% Confidence Interval (CI) of HR	p-value		Hazard Ratio (HR)	95% Confidence Interval (CI) of HR	p-value
UNADJUSTED	**N=223**				**N=161**				**N=233**			
	**HLA-DRB1** (+1 eplet increase)	1.023	1.002-1.045	0.028	**HLA-DQA1** (+1 eplet increase)	1.044	0.975-1.119	0.216	**HLA-DQB1** (+1 eplet increase)	1.017	0.990-1.044	0.221
	**HLA-DRB1** (ref=Zero)				**HLA-DQA1** (ref=Zero)				**HLA-DQB1** (ref=Zero)			
	**Low**	2.121	0.516-8.721	0.297	**Low**	1.746	0.858-3.551	0.124	**Low**	2.196	0.943-5.114	0.068
	**High**	3.256	0.780-13.605	0.106	**High**	2.508	0.826-7.614	0.105	**High**	2.733	1.095-6.827	0.031
ADJUSTED*	**N=209**				**N=149**				**N=219**			
	**HLA-DRB1** (+1 eplet increase)	1.035	1.009-1.062	0.011	**HLA-DQA1** (+1 eplet increase)	1.061	0.979-1.150	0.147	**HLA-DQB1** (+1 eplet increase)	1.024	0.992-1.058	0.141
	**HLA-DRB1** (ref=Zero)				**HLA-DQA1** (ref=Zero)				**HLA-DQB1** (ref=Zero)			
	**Low**	1.999	0.444-8.987	0.367	**Low**	1.995	0.878-4.536	0.099	**Low**	3.081	1.196-7.935	0.020
	**High**	3.745	0.812-17.270	0.090	**High**	3.452	0.949-12.551	0.060	**High**	4.368	1.520-12.554	0.006

*adjusted for: recipient age, recipient sex, recipient race/ethnicity, recipient BMI, dialysis vintage, cause of end stage renal disease, preemptive kidney transplantation, history of prior kidney transplantation, multi-organ transplantation, type of induction therapy, donor age, donor sex, donor race/ethnicity, donor type [living vs. deceased], cold ischemic time, cytomegalovirus risk status, cPRA at allocation

Table S42: Patient characteristics for the donor-derived cell-free DNA cohort based on eplet mismatch load for HLA-A locus

	**Zero Eplet Mismatch Group**	**Low Eplet Mismatch Group**	**High Eplet Mismatch Group**	**p-value**
**N**	42	373	37	
**<u>Recipients' characteristics</u>**				
**Age (years), mean (SD)**	56.6 (14.9)	50.1 (15.2)	46.7 (13.4)	0.009
**Sex (N (%))**				0.091
male	28 (66.7%)	237 (63.5%)	17 (45.9%)	
female	14 (33.3%)	136 (36.5%)	20 (54.1%)	
**Race/ethnicity (N (%))**				0.019
White	34 (85.0%)	248 (69.3%)	15 (45.5%)	
Hispanic	5 (12.5%)	74 (20.7%)	9 (27.3%)	
African American	0 (0.0%)	9 (2.5%)	1 (3.0%)	
Asian	1 (2.5%)	13 (3.6%)	3 (9.1%)	
Other	0 (0.0%)	13 (3.6%)	5 (15.2%)	
Unknown	0 (0.0%)	1 (0.3%)	0 (0.0%)	
**Body Mass Index (kg/m2), mean (SD)**	29.1 (4.8)	29.1 (5.7)	29.6 (5.4)	0.87
**Dialysis vintage (months), median (IQR)**	25.9 (11.7-41.1)	32.2 (14.7-52.9)	36.0 (19.1-62.4)	0.21
**Cause of kidney failure (N (%))**				0.57
Diabetes mellitus	20 (47.6%)	116 (31.1%)	13 (35.2%)	
Hypertension/Vascular disease	6 (14.3%)	47 (12.6%)	5 (13.5%)	
Cystic kidney disease	2 (4.8%)	36 (9.7%)	2 (5.4%)	
Glomerulonephritis	9 (21.4%)	97 (26.0%)	9 (24.3%)	
Other/unknown	5 (11.9%)	77 (20.6%)	8 (21.6%)	
**Prior kidney transplant (N (%))**				0.81
No	42 (100.0%)	371 (99.5%)	37 (100.0%)	
Yes	0 (0.0%)	2 (0.5%)	0 (0.0%)	
**Multi-organ transplantation (N (%))**				0.81
Liver + kidney	0 (0.0%)	0 (0.0%)	0 (0.0%)	
Pancreas + kidney	0 (0.0%)	2 (0.5%)	0 (0.0%)	
Heart + kidney	0 (0.0%)	0 (0.0%)	0 (0.0%)	
None	42 (100.0%)	371 (99.5%)	37 (100.0%)	

Liver +Heart+Kidney	0 (0.0%)	0 (0.0%)	0 (0.0%)	
**CMV risk categories (N (%))**				0.10
Low	9 (21.4%)	94 (25.2%)	9 (24.3%)	
Intermediate	19 (45.2%)	171 (45.8%)	18 (48.6%)	
High	9 (21.4%)	97 (26.0%)	10 (27.0%)	
Unknown	5 (11.9%)	11 (2.9%)	0 (0.0%)	
**Was the donor organ pumped? (N (%))**				0.010
No	21 (56.8%)	119 (34.3%)	9 (25.0%)	
Yes	16 (43.2%)	228 (65.7%)	27 (75.0%)	
**En block kidney (N (%))**	0 (0.0%)	0 (0.0%)	0 (0.0%)	NA
**Dual kidney (N (%))**	0 (0.0%)	0 (0.0%)	0 (0.0%)	NA
**Transplantation characteristics**				
**Cold ischemic time (hours), median (IQR)**	7.6 (2.6-16.4)	12.5 (4.0-19.5)	15.8 (8.3-18.4)	0.091
**cPRA (%), median (IQR)**	0 (0-6)	0 (0-0)	0 (0-6)	0.24
**Donors' characteristics**				
**Age (years), mean (SD)**	41.8 (13.9)	38.5 (14.3)	34.2 (14.4)	0.062
**Sex (N (%))**				0.45
male	26 (61.9%)	193 (51.7%)	19 (51.4%)	
female	16 (38.1%)	180 (48.3%)	18 (48.6%)	
**Race/ethnicity**				0.13
White	32 (76.2%)	308 (82.6%)	29 (78.4%)	
African American	1 (2.4%)	10 (2.7%)	0 (0.0%)	
Asian	1 (2.4%)	6 (1.6%)	1 (2.7%)	
Other	3 (7.1%)	4 (1.1%)	0 (0.0%)	
Unknown	5 (11.9%)	45 (12.1%)	7 (18.9%)	
**Donor type (N (%))**				0.010
Living	24 (57.1%)	142 (38.1%)	9 (24.3%)	
Deceased	18 (42.9%)	231 (61.9%)	28 (75.7%)	
**Donor DCD (N (%))**				0.99
No	10 (55.6%)	125 (54.1%)	15 (53.6%)	
Yes	8 (44.4%)	106 (45.9%)	13 (46.4%)	
**KDPI, median (IQR)**	28 (13-63)	27 (14-49)	24 (10-41)	0.50
**Donor cause of death (N (%))**				0.58
Anoxia	12 (66.7%)	112 (48.5%)	17 (60.7%)	
Cerebrovascular/stroke	4 (22.2%)	36 (15.6%)	3 (10.7%)	
Head trauma	2 (11.1%)	76 (32.9%)	7 (25.0%)	

Central nervous system tumor	0 (0.0%)	2 (0.9%)	0 (0.0%)	
Other	0 (0.0%)	5 (2.2%)	1 (3.6%)	
**Donor terminal creatinine (mg/dl), median (IQR)**	0.85 (0.65-1.0)	0.81 (0.67-1.0)	0.80 (0.61-1.06)	0.92
**Donor diabetes (N (%))**				0.32
No	41 (97.6%)	352 (95.1%)	36 (100.0%)	
Yes	1 (2.4%)	18 (4.9%)	0 (0.0%)	
**Donor hypertension (N (%))**				0.45
No	38 (90.5%)	309 (84.2%)	32 (88.9%)	
Yes	4 (9.5%)	58 (15.8%)	4 (11.1%)	
**Donor malignancy (N (%))**				0.029
No	42 (100.0%)	366 (98.1%)	34 (91.9%)	
Yes	0 (0.0%)	7 (1.9%)	3 (8.1%)	
**Immunological characteristics**				
**Number of HLA mismatches (HLA A, B and DR) (N (%))**				<0.001
1	7 (16.7%)	3 (0.8%)	0 (0.0%)	
2	8 (19.0%)	18 (4.8%)	0 (0.0%)	
3	18 (42.9%)	63 (16.9%)	2 (5.4%)	
4	9 (21.4%)	99 (26.5%)	3 (8.1%)	
5	0 (0.0%)	123 (33.0%)	19 (51.4%)	
6	0 (0.0%)	67 (18.0%)	13 (35.1%)	
**HLA mismatches A (N (%))**				<0.001
0	13 (100%)	1 (1%)	0 (0%)	
1	0 (0%)	50 (51%)	0 (0%)	
2	0 (0%)	47 (48%)	9 (100%)	
**HLA mismatches B (N (%))**				0.72
0	1 (8%)	2 (2%)	0 (0%)	
1	4 (31%)	29 (30%)	2 (22%)	
2	8 (62%)	67 (68%)	7 (78%)	
**HLA mismatches DR (N (%))**				0.18
0	2 (7.4%)	22 (11.1%)	3 (16.7%)	
1	20 (74.1%)	100 (50.3%)	8 (44.4%)	
2	5 (18.5%)	77 (38.7%)	7 (38.9%)	

**HLA mismatches DQ (N (%))**				0.92
0	2 (15%)	17 (18%)	2 (22%)	
1	7 (54%)	45 (46%)	3 (33%)	
2	4 (31%)	35 (36%)	4 (44%)	
**Current flow B cell (N (%))**				0.65
< 2 SD compatible	24 (88.9%)	192 (94.6%)	18 (94.7%)	
2-3 SD compatible	2 (7.4%)	5 (2.5%)	0 (0.0%)	
weak positive	0 (0.0%)	2 (1.0%)	0 (0.0%)	
positive	1 (3.7%)	4 (2.0%)	1 (5.3%)	
**Current flow T cell (N (%))**				0.88
< 2 SD compatible	26 (96.3%)	193 (95.1%)	18 (94.7%)	
2-3 SD compatible	1 (3.7%)	5 (2.5%)	0 (0.0%)	
weak positive	0 (0.0%)	1 (0.5%)	0 (0.0%)	
positive	0 (0.0%)	4 (2.0%)	1 (5.3%)	
**Induction treatment (N (%))**				
No Induction	0 (0.0%)	0 (0.0%)	0 (0.0%)	NA
Basiliximab	8 (19.0%)	45 (12.1%)	4 (10.8%)	0.41
Thymoglobulin	33 (78.6%)	319 (85.5%)	34 (91.9%)	0.24
Steroid	42 (100.0%)	372 (99.7%)	37 (100.0%)	0.90
Alemtuzumab	1 (2.4%)	5 (1.3%)	0 (0.0%)	0.65
**Outcomes**				
**Proportion of Antibody Mediated Rejection of the subset of patients who underwent biopsy (N (%))**				0.84
No	9 (81.8%)	100 (75.2%)	15 (78.9%)	
Yes	2 (18.2%)	33 (24.8%)	4 (21.1%)	
**Proportion of rejection of the subset of patients who underwent biopsy (N (%))**				0.74
No	7 (63.6%)	82 (61.7%)	10 (52.6%)	
Yes	4 (36.4%)	51 (38.3%)	9 (47.4%)	
**Proportion of T-cell Mediated Rejection of the subset of patients who underwent biopsy (N (%))**				0.41

No	8 (72.7%)	89 (66.9%)	10 (52.6%)	
Yes	3 (27.3%)	44 (33.1%)	9 (47.4%)	
**Post-Transplant de novo Donor Specific Antibodies (N (%))**				0.019
No	38 (95.0%)	297 (80.1%)	26 (70.3%)	
Yes	2 (5.0%)	74 (19.9%)	11 (29.7%)	
**Dd-cf-DNA elevation (N (%))**				0.013
No	37 (88.1%)	298 (79.9%)	23 (62.2%)	
Yes	5 (11.9%)	75 (20.1%)	14 (37.8%)	
**Delayed Graft Function (N (%))**				0.82
No	40 (95.2%)	347 (93.0%)	34 (91.9%)	
Yes	2 (4.8%)	26 (7.0%)	3 (8.1%)	
**Death (N (%))**				0.63
No	40 (95.2%)	362 (97.1%)	35 (94.6%)	
Yes	2 (4.8%)	11 (2.9%)	2 (5.4%)	
**Graft Loss (N (%))**				0.59
No	42 (100.0%)	368 (98.7%)	37 (100.0%)	
Yes	0 (0.0%)	5 (1.3%)	0 (0.0%)	

Values are expressed as mean (standard deviation), median (interquartile range), or number (%). Continuous variables were compared via t-tests or Mann-Whitney U tests. Categorical variables were compared via Chi-square tests.

Table S43: Patient characteristics for the donor-derived cell-free DNA cohort based on eplet mismatch load for HLA-B locus

	Zero Eplet Mismatch Group	Low Eplet Mismatch Group	High Eplet Mismatch Group	p-value
**N**	18	111	326	
**Recipients' characteristics**				
**Age (years), mean (SD)**	56.4 (9.2)	50.6 (16.2)	50.2 (15.1)	0.24
**Sex (N (%))**				0.25
male	8 (44.4%)	71 (64.0%)	208 (63.8%)	
female	10 (55.6%)	40 (36.0%)	118 (36.2%)	
**Race/ethnicity (N (%))**				0.55
White	15 (83.3%)	74 (69.8%)	208 (67.1%)	
Hispanic	0 (0.0%)	23 (21.7%)	66 (21.3%)	
African American	0 (0.0%)	3 (2.8%)	9 (2.9%)	
Asian	1 (5.6%)	3 (2.8%)	14 (4.5%)	
Other	2 (11.1%)	3 (2.8%)	12 (3.9%)	
Unknown	0 (0.0%)	0 (0.0%)	1 (0.3%)	
**Body Mass Index (kg/m2), mean (SD)**	31.3 (4.8)	28.5 (5.4)	29.2 (5.7)	0.12
**Dialysis vintage (months), median (IQR)**	44.5 (26.5-64.0)	25.9 (13.7-43.5)	34.3 (16.1-43.5)	0.14
**Cause of kidney failure (N (%))**				0.49
Diabetes mellitus	7 (38.9%)	28 (25.2%)	112 (34.4%)	
Hypertension/Vascular disease	2 (11.1%)	17 (15.3%)	41 (12.6%)	
Cystic kidney disease	2 (11.1%)	7 (6.3%)	32 (9.8%)	
Glomerulonephritis	3 (16.7%)	30 (27.0%)	82 (25.1%)	
Other/unknown	4 (22.2%)	29 (26.2%)	59 (18.1%)	
**Prior kidney transplant (N (%))**				0.67
No	18 (100.0%)	111 (100.0%)	324 (99.4%)	
Yes	0 (0.0%)	0 (0.0%)	2 (0.6%)	
**Multi-organ transplantation (N (%))**				0.67
Liver + kidney	0 (0.0%)	0 (0.0%)	0 (0.0%)	
Pancreas + kidney	0 (0.0%)	0 (0.0%)	2 (0.6%)	
Heart + kidney	0 (0.0%)	0 (0.0%)	0 (0.0%)	
None	18 (100.0%)	111 (100.0%)	324 (99.4%)	

Liver +Heart+Kidney	0 (0.0%)	0 (0.0%)	0 (0.0%)	
**CMV risk categories (N (%))**				<0.001
Low	2 (11.1%)	33 (29.7%)	79 (24.2%)	
Intermediate	7 (38.9%)	43 (38.7%)	157 (48.2%)	
High	5 (27.8%)	30 (27.0%)	82 (25.2%)	
Unknown	4 (22.2%)	5 (4.5%)	8 (2.5%)	
**Was the donor organ pumped? (N (%))**				0.007
No	9 (52.9%)	46 (45.1%)	92 (30.3%)	
Yes	8 (47.1%)	56 (54.9%)	212 (69.7%)	
**Transplantation characteristics**				
**Cold ischemic time (hours), median (IQR)**	5.9 (3.1-21.7)	9.0 (3.0-19.6)	13.6 (5.4-19.4)	0.095
**cPRA (%), median (IQR)**	0 (0-59)	0 (0-0)	0 (0-0)	0.013
**Donors' characteristics**				
**Age (years), mean (SD)**	42.9 (11.7)	40.3 (13.3)	37.7 (14.7)	0.11
**Sex (N (%))**				0.53
male	8 (44.4%)	55 (49.5%)	177 (54.3%)	
female	10 (55.6%)	56 (50.5%)	149 (45.7%)	
**Race/ethnicity**				0.64
White	17 (94.4%)	89 (80.2%)	265 (81.3%)	
African American	0 (0.0%)	2 (1.8%)	9 (2.8%)	
Asian	1 (5.6%)	2 (1.8%)	6 (1.8%)	
Other	0 (0.0%)	1 (0.9%)	6 (1.8%)	
Unknown	0 (0.0%)	17 (15.3%)	40 (12.3%)	
**Donor type (N (%))**				0.003
Living	10 (55.6%)	55 (49.5%)	108 (33.1%)	
Deceased	8 (44.4%)	56 (50.5%)	218 (66.9%)	
**Donor DCD (N (%))**				0.59
No	3 (37.5%)	31 (55.4%)	122 (56.0%)	
Yes	5 (62.5%)	25 (44.6%)	96 (44.0%)	
**KDPI, median (IQR)**	32 (21-43)	27 (13-55)	26 (12-49)	0.77
**Donor cause of death (N (%))**				0.49
Anoxia	6 (75.0%)	25 (44.6%)	112 (51.4%)	
Cerebrovascular/stroke	1 (12.5%)	13 (23.2%)	31 (14.2%)	
Head trauma	1 (12.5%)	17 (30.4%)	68 (31.2%)	
Central nervous system tumor	0 (0.0%)	1 (1.8%)	1 (0.5%)	

Other	0 (0.0%)	0 (0.0%)	6 (2.8%)	
**Donor terminal creatinine (mg/dl), median (IQR)**	0.91 (0.77-1.04)	0.83 (0.70-1.07)	0.82 (0.63-1.0)	0.42
**Donor diabetes (N (%))**				0.050
No	17 (94.4%)	109 (100.0%)	307 (94.8%)	
Yes	1 (5.6%)	0 (0.0%)	17 (5.2%)	
**Donor hypertension (N (%))**				0.14
No	18 (100.0%)	94 (87.0%)	270 (83.9%)	
Yes	0 (0.0%)	14 (13.0%)	52 (16.1%)	
**Donor malignancy (N (%))**				0.53
No	17 (94.4%)	108 (97.3%)	320 (98.2%)	
Yes	1 (5.6%)	3 (2.7%)	6 (1.8%)	
**Immunological characteristics**				
**Number of HLA mismatches (HLA A, B and DR) (N (%))**				<0.001
1	6 (33.3%)	3 (2.7%)	1 (0.3%)	
2	6 (33.3%)	10 (9.0%)	9 (2.8%)	
3	3 (16.7%)	37 (33.3%)	40 (12.3%)	
4	2 (11.1%)	28 (25.2%)	81 (24.8%)	
5	1 (5.6%)	24 (21.6%)	124 (38.0%)	
6	0 (0.0%)	9 (8.1%)	71 (21.8%)	
**HLA mismatches A (N (%))**				0.12
0	2 (50%)	3 (12%)	9 (10%)	
1	1 (25%)	13 (50%)	35 (38%)	
2	1 (25%)	10 (38%)	47 (52%)	
**HLA mismatches B (N (%))**				<0.001
0	2 (50%)	1 (4%)	0 (0%)	
1	2 (50%)	16 (62%)	16 (18%)	
2	0 (0%)	9 (35%)	75 (82%)	
**HLA mismatches DR (N (%))**				0.011
0	2 (16.7%)	8 (14.5%)	16 (9.2%)	
1	8 (66.7%)	37 (67.3%)	84 (48.3%)	
2	2 (16.7%)	10 (18.2%)	74 (42.5%)	
**HLA mismatches DQ (N (%))**				0.21

0	0 (0%)	8 (31%)	12 (13%)	
1	3 (75%)	10 (38%)	44 (49%)	
2	1 (25%)	8 (31%)	34 (38%)	
**Current flow B cell (N (%))**				0.78
< 2 SD compatible	11 (91.7%)	51 (92.7%)	170 (94.4%)	
2-3 SD compatible	0 (0.0%)	2 (3.6%)	5 (2.8%)	
weak positive	0 (0.0%)	1 (1.8%)	1 (0.6%)	
positive	1 (8.3%)	1 (1.8%)	4 (2.2%)	
**Current flow T cell (N (%))**				0.87
< 2 SD compatible	11 (91.7%)	53 (96.4%)	171 (95.0%)	
2-3 SD compatible	1 (8.3%)	1 (1.8%)	4 (2.2%)	
weak positive	0 (0.0%)	0 (0.0%)	1 (0.6%)	
positive	0 (0.0%)	1 (1.8%)	4 (2.2%)	
**Induction treatment (N (%))**				
No Induction	0 (0.0%)	0 (0.0%)	0 (0.0%)	NA
Basiliximab	3 (16.7%)	17 (15.3%)	39 (12.0%)	0.59
Thymoglobulin	15 (83.3%)	91 (82.0%)	282 (86.5%)	0.50
Steroid	18 (100.0%)	111 (100.0%)	325 (99.7%)	0.82
Alemtuzumab	0 (0.0%)	1 (0.9%)	4 (1.2%)	0.87
**Outcomes**				
**Proportion of Antibody Mediated Rejection of the subset of patients who underwent biopsy (N (%))**				0.71
No	3 (60.0%)	27 (77.1%)	90 (75.0%)	
Yes	2 (40.0%)	8 (22.9%)	30 (25.0%)	
**Proportion of rejection of the subset of patients who underwent biopsy (N (%))**				0.74
No	3 (60.0%)	23 (65.7%)	70 (58.3%)	
Yes	2 (40.0%)	12 (34.3%)	50 (41.7%)	
**Proportion of T-cell Mediated Rejection of the subset of patients who underwent biopsy (N (%))**				0.97
No	3 (60.0%)	23 (65.7%)	78 (65.0%)	

Yes	2 (40.0%)	12 (34.3%)	42 (35.0%)	
**Post-Transplant de novo Donor Specific Antibodies (N (%))**				0.69
No	15 (83.3%)	90 (82.6%)	257 (79.1%)	
Yes	3 (16.7%)	19 (17.4%)	68 (20.9%)	
**Dd-cf-DNA elevation (N (%))**				0.033
No	16 (88.9%)	96 (86.5%)	247 (75.8%)	
Yes	2 (11.1%)	15 (13.5%)	79 (24.2%)	
**Delayed Graft Function (N (%))**				0.51
No	18 (100.0%)	103 (92.8%)	305 (93.6%)	
Yes	0 (0.0%)	8 (7.2%)	21 (6.4%)	
**Death (N (%))**				0.009
No	18 (100.0%)	102 (91.9%)	319 (97.9%)	
Yes	0 (0.0%)	9 (8.1%)	7 (2.1%)	
**Graft Loss (N (%))**				0.10
No	17 (94.4%)	111 (100.0%)	322 (98.8%)	
Yes	1 (5.6%)	0 (0.0%)	4 (1.2%)	

Values are expressed as mean (standard deviation), median (interquartile range), or number (%). Continuous variables were compared via t-tests or Mann-Whitney U tests. Categorical variables were compared via Chi-square tests.

Table S44: Patient characteristics for the donor-derived cell-free DNA cohort based on eplet mismatch load for HLA-C locus

	Zero Eplet Mismatch Group	Low Eplet Mismatch Group	High Eplet Mismatch Group	p-value
**N**	39	215	192	
**<u>Recipients' characteristics</u>**				
**Age (years), mean (SD)**	49.2 (14.7)	51.1 (15.8)	49.5 (14.4)	0.52
**Sex (N (%))**				0.29
male	20 (51.3%)	136 (63.3%)	124 (64.6%)	
female	19 (48.7%)	79 (36.7%)	68 (35.4%)	
**Race/ethnicity (N (%))**				0.32
White	31 (81.6%)	147 (70.3%)	114 (63.0%)	
Hispanic	3 (7.9%)	39 (18.7%)	46 (25.4%)	
African American	1 (2.6%)	6 (2.9%)	4 (2.2%)	
Asian	1 (2.6%)	10 (4.8%)	6 (3.3%)	
Other	2 (5.3%)	6 (2.9%)	11 (6.1%)	
Unknown	0 (0.0%)	1 (0.5%)	0 (0.0%)	
**Body Mass Index (kg/m2), mean (SD)**	28.8 (5.1)	29.3 (5.5)	29.1 (5.8)	0.85
**Dialysis vintage (months), median (IQR)**	28.7 (19.3-48.9)	28.9 (12.1-45.1)	34.4 (18.0-59.9)	0.041
**Cause of kidney failure (N (%))**				0.45
Diabetes mellitus	7 (18.0%)	66 (30.7%)	71 (37.0%)	
Hypertension/Vascular disease	7 (18.0%)	32 (14.9%)	20 (10.4%)	
Cystic kidney disease	3 (7.7%)	19 (8.8%)	17 (8.9%)	
Glomerulonephritis	13 (33.3%)	53 (24.7%)	49 (25.5%)	
Other/unknown	9 (23.0%)	45 (20.9%)	35 (18.2%)	
**Prior kidney transplant (N (%))**				0.26
No	39 (100.0%)	215 (100.0%)	190 (99.0%)	
Yes	0 (0.0%)	0 (0.0%)	2 (1.0%)	
**Multi-organ transplantation (N (%))**				0.91
Liver + kidney	0 (0.0%)	0 (0.0%)	0 (0.0%)	
Pancreas + kidney	0 (0.0%)	1 (0.5%)	1 (0.5%)	
Heart + kidney	0 (0.0%)	0 (0.0%)	0 (0.0%)	
None	39 (100.0%)	214 (99.5%)	191 (99.5%)	

Liver +Heart+Kidney	0 (0.0%)	0 (0.0%)	0 (0.0%)	
**CMV risk categories (N (%))**				0.040
Low	5 (12.8%)	61 (28.4%)	43 (22.4%)	
Intermediate	16 (41.0%)	98 (45.6%)	90 (46.9%)	
High	14 (35.9%)	48 (22.3%)	55 (28.6%)	
Unknown	4 (10.3%)	8 (3.7%)	4 (2.1%)	
**Was the donor organ pumped? (N (%))**				0.24
No	16 (44.4%)	74 (37.6%)	57 (31.5%)	
Yes	20 (55.6%)	123 (62.4%)	124 (68.5%)	
**En block kidney (N (%))**	0 (0.0%)	0 (0.0%)	0 (0.0%)	NA
**Dual kidney (N (%))**	0 (0.0%)	0 (0.0%)	0 (0.0%)	NA
**Transplantation characteristics**				
**Cold ischemic time (hours), median (IQR)**	9.0 (2.8-21.2)	12.0 (3.3-19.1)	13.4 (5.6-19.0)	0.42
**cPRA (%), median (IQR)**	0 (0-46)	0 (0-0)	0 (0-0)	0.10
**Donors' characteristics**				
**Age (years), mean (SD)**	40.3 (13.8)	38.6 (14.3)	37.7 (14.5)	0.53
**Sex (N (%))**				0.26
male	16 (41.0%)	119 (55.3%)	103 (53.6%)	
female	23 (59.0%)	96 (44.7%)	89 (46.4%)	
**Race/ethnicity**				0.19
White	34 (87.2%)	178 (82.8%)	152 (79.2%)	
African American	1 (2.6%)	3 (1.4%)	7 (3.6%)	
Asian	1 (2.6%)	4 (1.9%)	3 (1.6%)	
Other	2 (5.1%)	1 (0.5%)	4 (2.1%)	
Unknown	1 (2.6%)	29 (13.5%)	26 (13.5%)	
**Donor type (N (%))**				0.12
Living	19 (48.7%)	89 (41.4%)	65 (33.9%)	
Deceased	20 (51.3%)	126 (58.6%)	127 (66.1%)	
**Donor DCD (N (%))**				0.78
No	11 (55.0%)	73 (57.9%)	68 (53.5%)	
Yes	9 (45.0%)	53 (42.1%)	59 (46.5%)	
**KDPI, median (IQR)**	24 (14-40)	25 (12-47)	26 (11-49)	0.63
**Donor cause of death (N (%))**				0.93
Anoxia	10 (50.0%)	60 (47.6%)	68 (53.5%)	
Cerebrovascular/stroke	3 (15.0%)	24 (19.0%)	16 (12.6%)	
Head trauma	7 (35.0%)	38 (30.2%)	40 (31.5%)	

Central nervous system tumor	0 (0.0%)	1 (0.8%)	1 (0.8%)	
Other	0 (0.0%)	3 (2.4%)	2 (1.6%)	
**Donor terminal creatinine (mg/dl), median (IQR)**	0.83 (0.72-0.98)	0.83 (0.69-1.0)	0.80 (0.62-1.01)	0.41
**Donor diabetes (N (%))**				0.89
No	38 (97.4%)	203 (96.2%)	184 (95.8%)	
Yes	1 (2.6%)	8 (3.8%)	8 (4.2%)	
**Donor hypertension (N (%))**				0.004
No	39 (100.0%)	184 (87.6%)	153 (80.5%)	
Yes	0 (0.0%)	26 (12.4%)	37 (19.5%)	
**Donor malignancy (N (%))**				0.87
No	38 (97.4%)	211 (98.1%)	187 (97.4%)	
Yes	1 (2.6%)	4 (1.9%)	5 (2.6%)	
**Immunological characteristics**				
**Number of HLA mismatches (HLA A, B and DR) (N (%))**				<0.001
1	7 (17.9%)	3 (1.4%)	0 (0.0%)	
2	8 (20.5%)	14 (6.5%)	4 (2.1%)	
3	9 (23.1%)	56 (26.0%)	16 (8.3%)	
4	8 (20.5%)	45 (20.9%)	55 (28.6%)	
5	7 (17.9%)	62 (28.8%)	72 (37.5%)	
6	0 (0.0%)	35 (16.3%)	45 (23.4%)	
**HLA mismatches A (N (%))**				0.23
0	2 (25%)	5 (8%)	7 (15%)	
1	5 (62%)	26 (42%)	17 (37%)	
2	1 (12%)	31 (50%)	22 (48%)	
**HLA mismatches B (N (%))**				<0.001
0	2 (25%)	1 (2%)	0 (0%)	
1	5 (62%)	23 (37%)	6 (13%)	
2	1 (12%)	38 (61%)	40 (87%)	
**HLA mismatches DR (N (%))**				0.32
0	5 (21.7%)	14 (11.2%)	8 (8.8%)	
1	13 (56.5%)	66 (52.8%)	46 (50.5%)	
2	5 (21.7%)	45 (36.0%)	37 (40.7%)	

**HLA mismatches DQ (N (%))**				0.40
0	2 (25%)	13 (21%)	6 (13%)	
1	5 (62%)	29 (47%)	19 (42%)	
2	1 (12%)	20 (32%)	20 (44%)	
**Current flow B cell (N (%))**				0.25
< 2 SD compatible	21 (91.3%)	118 (95.2%)	90 (92.8%)	
2-3 SD compatible	2 (8.7%)	1 (0.8%)	4 (4.1%)	
weak positive	0 (0.0%)	2 (1.6%)	0 (0.0%)	
positive	0 (0.0%)	3 (2.4%)	3 (3.1%)	
**Current flow T cell (N (%))**				0.013
< 2 SD compatible	20 (87.0%)	121 (97.6%)	91 (93.8%)	
2-3 SD compatible	2 (8.7%)	1 (0.8%)	3 (3.1%)	
weak positive	1 (4.3%)	0 (0.0%)	0 (0.0%)	
positive	0 (0.0%)	2 (1.6%)	3 (3.1%)	
**Induction treatment (N (%))**				
No Induction	0 (0.0%)	0 (0.0%)	0 (0.0%)	NA
Basiliximab	5 (12.8%)	35 (16.3%)	18 (9.4%)	0.12
Thymoglobulin	33 (84.6%)	177 (82.3%)	169 (88.0%)	0.28
Steroid	39 (100.0%)	215 (100.0%)	191 (99.5%)	0.52
Alemtuzumab	0 (0.0%)	0 (0.0%)	6 (3.1%)	0.018
**<u>Outcomes</u>**				
**Proportion of Antibody Mediated Rejection of the subset of patients who underwent biopsy (N (%))**				0.74
No	11 (73%)	56 (78%)	52 (72%)	
Yes	4 (27%)	16 (22%)	20 (28%)	
**Proportion of rejection of the subset of patients who underwent biopsy (N (%))**				0.033
No	10 (67%)	50 (69%)	35 (49%)	
Yes	5 (33%)	22 (31%)	37 (51%)	
**Proportion of T-cell Mediated Rejection of the subset of patients who underwent biopsy (N (%))**				0.032

No	10 (67%)	54 (75%)	39 (54%)	
Yes	5 (33%)	18 (25%)	33 (46%)	
**Post-Transplant de novo Donor Specific Antibodies (N (%))**				0.043
No	29 (74.4%)	182 (85.4%)	146 (76.4%)	
Yes	10 (25.6%)	31 (14.6%)	45 (23.6%)	
**Dd-cf-DNA elevation (N (%))**				0.015
No	31 (79.5%)	182 (84.7%)	140 (72.9%)	
Yes	8 (20.5%)	33 (15.3%)	52 (27.1%)	
**Delayed Graft Function (N (%))**				0.072
No	39 (100.0%)	204 (94.9%)	175 (91.1%)	
Yes	0 (0.0%)	11 (5.1%)	17 (8.9%)	
**Death (N (%))**				0.47
No	39 (100.0%)	207 (96.3%)	185 (96.4%)	
Yes	0 (0.0%)	8 (3.7%)	7 (3.6%)	
**Graft Loss (N (%))**				0.092
No	38 (97.4%)	215 (100.0%)	188 (97.9%)	
Yes	1 (2.6%)	0 (0.0%)	4 (2.1%)	

Values are expressed as mean (standard deviation), median (interquartile range), or number (%). Continuous variables were compared via t-tests or Mann-Whitney U tests. Categorical variables were compared via Chi-square tests.

Table S45: Patient characteristics for the donor-derived cell-free DNA cohort based on eplet mismatch load for HLA-DQA1 locus

	Zero Eplet Mismatch Group	Low Eplet Mismatch Group	High Eplet Mismatch Group	p-value
**N**	79	136	162	
**<u>Recipients' characteristics</u>**				
**Age (years), mean (SD)**	53.8 (15.1)	51.0 (14.7)	48.8 (15.5)	0.052
**Sex (N (%))**				0.046
male	46 (58.2%)	76 (55.9%)	112 (69.1%)	
female	33 (41.8%)	60 (44.1%)	50 (30.9%)	
**Race/ethnicity (N (%))**				0.017
White	60 (77.9%)	90 (69.8%)	101 (65.6%)	
Hispanic	10 (13.0%)	31 (24.0%)	29 (18.8%)	
African American	4 (5.2%)	2 (1.6%)	4 (2.6%)	
Asian	2 (2.6%)	6 (4.7%)	7 (4.5%)	
Other	1 (1.3%)	0 (0.0%)	12 (7.8%)	
Unknown	0 (0.0%)	0 (0.0%)	1 (0.6%)	
**Body Mass Index (kg/m2), mean (SD)**	28.3 (5.9)	29.2 (5.7)	29.2 (5.5)	0.49
**Dialysis vintage (months), median (IQR)**	22.0 (11.5-43.8)	32.1 (15.6-52.9)	34.5 (15.9-56.2)	0.081
**Cause of kidney failure (N (%))**				0.59
Diabetes mellitus	20 (25.3%)	49 (36.0%)	50 (30.9%)	
Hypertension/Vascular disease	12 (15.2%)	14 (10.3%)	22 (13.6%)	
Cystic kidney disease	10 (12.7%)	14 (10.3%)	13 (8.0%)	
Glomerulonephritis	21 (26.6%)	28 (20.6%)	46 (28.4%)	
Other/unknown	16 (20.2%)	31 (22.8%)	31 (19.1%)	
**Prior kidney transplant (N (%))**				0.13
No	79 (100.0%)	136 (100.0%)	159 (98.1%)	
Yes	0 (0.0%)	0 (0.0%)	3 (1.9%)	
**Multi-organ transplantation (N (%))**				0.76
Liver + kidney	0 (0.0%)	0 (0.0%)	0 (0.0%)	
Pancreas + kidney	0 (0.0%)	1 (0.7%)	1 (0.6%)	
Heart + kidney	0 (0.0%)	0 (0.0%)	0 (0.0%)	
None	79 (100.0%)	135 (99.3%)	161 (99.4%)	

Liver +Heart+Kidney	0 (0.0%)	0 (0.0%)	0 (0.0%)	
**CMV risk categories (N (%))**				0.55
Low	19 (24.1%)	35 (25.7%)	37 (22.8%)	
Intermediate	33 (41.8%)	59 (43.4%)	82 (50.6%)	
High	25 (31.6%)	34 (25.0%)	38 (23.5%)	
Unknown	2 (2.5%)	8 (5.9%)	5 (3.1%)	
**Was the donor organ pumped? (N (%))**				0.28
No	31 (42.5%)	58 (47.2%)	56 (37.6%)	
Yes	42 (57.5%)	65 (52.8%)	93 (62.4%)	
**En block kidney (N (%))**	0 (0.0%)	0 (0.0%)	0 (0.0%)	NA
**Dual kidney (N (%))**	0 (0.0%)	0 (0.0%)	0 (0.0%)	NA
**Transplantation characteristics**				
**Cold ischemic time (hours), median (IQR)**	10.2 (2.8-17.9)	8.5 (3.2-17.75)	13.4 (3.7-20.2)	0.14
**cPRA (%), median (IQR)**	0 (0-0)	0 (0-0)	0 (0-0)	0.41
**Donors' characteristics**				
**Age (years), mean (SD)**	36.5 (13.5)	39.3 (14.2)	38.7 (14.5)	0.37
**Sex (N (%))**				0.027
male	30 (38.0%)	77 (56.6%)	85 (52.5%)	
female	49 (62.0%)	59 (43.4%)	77 (47.5%)	
**Race/ethnicity**				0.72
White	65 (82.3%)	113 (83.1%)	123 (75.9%)	
African American	1 (1.3%)	3 (2.2%)	6 (3.7%)	
Asian	2 (2.5%)	1 (0.7%)	4 (2.5%)	
Other	2 (2.5%)	2 (1.5%)	2 (1.2%)	
Unknown	9 (11.4%)	17 (12.5%)	27 (16.7%)	
**Donor type (N (%))**				0.32
Living	36 (45.6%)	69 (50.7%)	68 (42.0%)	
Deceased	43 (54.4%)	67 (49.3%)	94 (58.0%)	
**Donor DCD (N (%))**				0.79
No	22 (51%)	37 (55%)	54 (57%)	
Yes	21 (49%)	30 (45%)	40 (43%)	
**KDPI, median (IQR)**	28 (11-45)	25 (15-45)	22 (10-49)	0.96
**Donor cause of death (N (%))**				0.55
Anoxia	24 (56%)	27 (40%)	46 (49%)	
Cerebrovascular/stroke	7 (16%)	13 (19%)	12 (13%)	
Head trauma	12 (28%)	25 (37%)	31 (33%)	

Central nervous system tumor	0 (0%)	0 (0%)	2 (2%)	
Other	0 (0%)	2 (3%)	3 (3%)	
**Donor terminal creatinine (mg/dl), median (IQR)**	0.79 (0.67-0.96)	0.84 (0.7-1.03)	0.82 (0.66-1.02)	0.45
**Donor diabetes (N (%))**				0.40
No	74 (93.7%)	130 (97.0%)	155 (96.9%)	
Yes	5 (6.3%)	4 (3.0%)	5 (3.1%)	
**Donor hypertension (N (%))**				0.18
No	74 (93.7%)	121 (90.3%)	136 (86.1%)	
Yes	5 (6.3%)	13 (9.7%)	22 (13.9%)	
**Donor malignancy (N (%))**				0.14
No	79 (100.0%)	134 (98.5%)	156 (96.3%)	
Yes	0 (0.0%)	2 (1.5%)	6 (3.7%)	
**Immunological characteristics**				
**Number of HLA mismatches (HLA A, B and DR) (N (%))**				<0.001
1	7 (8.9%)	3 (2.2%)	0 (0.0%)	
2	16 (20.3%)	8 (5.9%)	2 (1.2%)	
3	21 (26.6%)	28 (20.6%)	15 (9.3%)	
4	16 (20.3%)	37 (27.2%)	42 (25.9%)	
5	14 (17.7%)	39 (28.7%)	60 (37.0%)	
6	5 (6.3%)	21 (15.4%)	43 (26.5%)	
**HLA mismatches A (N (%))**				0.53
0	4 (20%)	4 (11%)	3 (7%)	
1	7 (35%)	15 (39%)	22 (48%)	
2	9 (45%)	19 (50%)	21 (46%)	
**HLA mismatches B (N (%))**				0.13
0	0 (0%)	3 (8%)	0 (0%)	
1	6 (30%)	14 (37%)	12 (26%)	
2	14 (70%)	21 (55%)	34 (74%)	
**HLA mismatches DR (N (%))**				<0.001
0	15 (35%)	4 (5%)	4 (4%)	
1	24 (56%)	56 (71%)	40 (42%)	
2	4 (9%)	19 (24%)	52 (54%)	

**HLA mismatches DQ (N (%))**				<0.001
0	9 (45%)	6 (16%)	2 (4%)	
1	10 (50%)	17 (45%)	24 (53%)	
2	1 (5%)	15 (39%)	19 (42%)	
**Current flow B cell (N (%))**				0.35
< 2 SD compatible	41 (97.6%)	74 (93.7%)	98 (96.1%)	
2-3 SD compatible	1 (2.4%)	4 (5.1%)	1 (1.0%)	
weak positive	0 (0.0%)	0 (0.0%)	0 (0.0%)	
positive	0 (0.0%)	1 (1.3%)	3 (2.9%)	
**Current flow T cell (N (%))**				0.85
< 2 SD compatible	39 (92.9%)	76 (96.2%)	99 (97.1%)	
2-3 SD compatible	2 (4.8%)	2 (2.5%)	2 (2.0%)	
weak positive	0 (0.0%)	0 (0.0%)	0 (0.0%)	
positive	1 (2.4%)	1 (1.3%)	1 (1.0%)	
**Induction treatment (N (%))**				
No Induction	0 (0.0%)	0 (0.0%)	0 (0.0%)	NA
Basiliximab	12 (15.2%)	21 (15.4%)	16 (9.9%)	0.29
Thymoglobulin	63 (79.7%)	111 (81.6%)	145 (89.5%)	0.069
Steroid	79 (100.0%)	135 (99.3%)	162 (100.0%)	0.41
Alemtuzumab	1 (1.3%)	3 (2.2%)	2 (1.2%)	0.77
**Outcomes**				
**Proportion of Antibody Mediated Rejection of the subset of patients who underwent biopsy (N (%))**				0.17
No	20 (87%)	27 (68%)	41 (67%)	
Yes	3 (13%)	13 (32%)	20 (33%)	
**Proportion of rejection of the subset of patients who underwent biopsy (N (%))**				0.50
No	16 (70%)	22 (55%)	35 (57%)	
Yes	7 (30%)	18 (45%)	26 (43%)	
**Proportion of T-cell Mediated Rejection of the subset of patients who underwent biopsy (N (%))**				0.49

No	17 (74%)	27 (68%)	37 (61%)	
Yes	6 (26%)	13 (32%)	24 (39%)	
**Post-Transplant de novo Donor Specific Antibodies (N (%))**				0.10
No	71 (89.9%)	110 (80.9%)	126 (78.8%)	
Yes	8 (10.1%)	26 (19.1%)	34 (21.2%)	
**Dd-cf-DNA elevation (N (%))**				0.065
No	69 (87.3%)	110 (80.9%)	121 (74.7%)	
Yes	10 (12.7%)	26 (19.1%)	41 (25.3%)	
**Delayed Graft Function (N (%))**				0.046
No	77 (97.5%)	131 (96.3%)	147 (90.7%)	
Yes	2 (2.5%)	5 (3.7%)	15 (9.3%)	
**Death (N (%))**				0.058
No	77 (97.5%)	135 (99.3%)	153 (94.4%)	
Yes	2 (2.5%)	1 (0.7%)	9 (5.6%)	
**Graft Loss (N (%))**				0.39
No	79 (100.0%)	133 (97.8%)	160 (98.8%)	
Yes	0 (0.0%)	3 (2.2%)	2 (1.2%)	

Values are expressed as mean (standard deviation), median (interquartile range), or number (%). Continuous variables were compared via t-tests or Mann-Whitney U tests. Categorical variables were compared via Chi-square tests.

Table S46: Patient characteristics for the donor-derived cell-free DNA cohort based on eplet mismatch load for HLA-DQB1 locus

	Zero Eplet Mismatch Group	Low Eplet Mismatch Group	High Eplet Mismatch Group	p-value
**N**	65	307	138	
**<u>Recipients' characteristics</u>**				
**Age (years), mean (SD)**	54.0 (15.6)	50.2 (15.1)	50.9 (14.5)	0.18
**Sex (N (%))**				0.60
male	38 (58.5%)	188 (61.2%)	90 (65.2%)	
female	27 (41.5%)	119 (38.8%)	48 (34.8%)	
**Race/ethnicity (N (%))**				0.29
White	52 (80.0%)	198 (69.0%)	80 (59.7%)	
Hispanic	11 (16.9%)	56 (19.5%)	34 (25.4%)	
African American	0 (0.0%)	7 (2.4%)	5 (3.7%)	
Asian	1 (1.5%)	13 (4.5%)	6 (4.5%)	
Other	1 (1.5%)	12 (4.2%)	9 (6.7%)	
Unknown	0 (0.0%)	1 (0.3%)	0 (0.0%)	
**Body Mass Index (kg/m2), mean (SD)**	28.5 (5.6)	28.8 (5.4)	30.0 (5.6)	0.057
**Dialysis vintage (months), median (IQR)**	26.5 (12.1-51.8)	33.8 (16.4-56.4)	38.6 (21.5-64.8)	0.074
**Cause of kidney failure (N (%))**				0.72
Diabetes mellitus	17 (26.2%)	99 (32.2%)	50 (36.2%)	
Hypertension/Vascular disease	12 (18.4%)	39 (12.7%)	19 (13.8%)	
Cystic kidney disease	6 (9.2%)	29 (9.5%)	12 (8.7%)	
Glomerulonephritis	13 (20.0%)	77 (25.1%)	34 (24.6%)	
Other/unknown	17 (26.2%)	63 (20.5%)	23 (16.7%)	
**Prior kidney transplant (N (%))**				0.76
No	64 (98.5%)	305 (99.3%)	137 (99.3%)	
Yes	1 (1.5%)	2 (0.7%)	1 (0.7%)	
**Multi-organ transplantation (N (%))**				0.51
Liver + kidney	0 (0.0%)	0 (0.0%)	0 (0.0%)	
Pancreas + kidney	0 (0.0%)	2 (0.7%)	0 (0.0%)	
Heart + kidney	0 (0.0%)	0 (0.0%)	0 (0.0%)	
None	65 (100.0%)	305 (99.3%)	138 (100.0%)	

Liver +Heart+Kidney	0 (0.0%)	0 (0.0%)	0 (0.0%)	
**CMV risk categories (N (%))**				0.72
Low	14 (21.5%)	85 (27.7%)	30 (21.7%)	
Intermediate	29 (44.6%)	129 (42.0%)	67 (48.6%)	
High	20 (30.8%)	80 (26.1%)	37 (26.8%)	
Unknown	2 (3.1%)	13 (4.2%)	4 (2.9%)	
**Was the donor organ pumped? (N (%))**				0.056
No	20 (34.5%)	103 (35.5%)	31 (23.8%)	
Yes	38 (65.5%)	187 (64.5%)	99 (76.2%)	
**En block kidney (N (%))**	0 (0.0%)	0 (0.0%)	0 (0.0%)	NA
**Dual kidney (N (%))**	0 (0.0%)	0 (0.0%)	0 (0.0%)	NA
**<u>Transplantation characteristics</u>**				
**Cold ischemic time (hours), median (IQR)**	12.5 (3.3-19.5)	11.7 (3.9-19.6)	14.7 (7.7-19.9)	0.17
**cPRA (%), median (IQR)**	0 (0-0)	0 (0-0)	0 (0-0)	0.67
**<u>Donors' characteristics</u>**				
**Age (years), mean (SD)**	40.4 (15.0)	37.9 (14.2)	38.3 (15.0)	0.44
**Sex (N (%))**				0.20
male	28 (43.1%)	163 (53.1%)	78 (56.5%)	
female	37 (56.9%)	144 (46.9%)	60 (43.5%)	
**Race/ethnicity**				0.25
White	48 (73.8%)	259 (84.4%)	112 (81.2%)	
African American	2 (3.1%)	8 (2.6%)	1 (0.7%)	
Asian	1 (1.5%)	6 (2.0%)	3 (2.2%)	
Other	1 (1.5%)	6 (2.0%)	1 (0.7%)	
Unknown	13 (20.0%)	28 (9.1%)	21 (15.2%)	
**Donor type (N (%))**				0.078
Living	26 (40.0%)	116 (37.8%)	38 (27.5%)	
Deceased	39 (60.0%)	191 (62.2%)	100 (72.5%)	
**Donor DCD (N (%))**				0.077
No	18 (46.2%)	112 (58.6%)	46 (46.0%)	
Yes	21 (53.8%)	79 (41.4%)	54 (54.0%)	
**KDPI, median (IQR)**	34 (21-57)	26 (13-49)	27 (15-47)	0.15
**Donor cause of death (N (%))**				0.38
Anoxia	19 (48.7%)	97 (50.8%)	51 (51.0%)	
Cerebrovascular/stroke	3 (7.7%)	29 (15.2%)	20 (20.0%)	
Head trauma	15 (38.5%)	57 (29.8%)	28 (28.0%)	

Central nervous system tumor	0 (0.0%)	1 (0.5%)	1 (1.0%)	
Other	2 (5.1%)	7 (3.7%)	0 (0.0%)	
**Donor terminal creatinine (mg/dl), median (IQR)**	0.79 (0.65-1.10)	0.81 (0.65-0.98)	0.90 (0.69-1.03)	0.21
**Donor diabetes (N (%))**				0.26
No	60 (92.3%)	291 (96.7%)	131 (94.9%)	
Yes	5 (7.7%)	10 (3.3%)	7 (5.1%)	
**Donor hypertension (N (%))**				0.62
No	58 (89.2%)	254 (85.2%)	116 (84.1%)	
Yes	7 (10.8%)	44 (14.8%)	22 (15.9%)	
**Donor malignancy (N (%))**				0.010
No	63 (96.9%)	305 (99.3%)	131 (94.9%)	
Yes	2 (3.1%)	2 (0.7%)	7 (5.1%)	
**Immunological characteristics**				
**Number of HLA mismatches (HLA A, B and DR) (N (%))**				<0.001
1	8 (12.3%)	3 (1.0%)	0 (0.0%)	
2	10 (15.4%)	17 (5.5%)	2 (1.4%)	
3	26 (40.0%)	57 (18.6%)	14 (10.1%)	
4	12 (18.5%)	74 (24.1%)	35 (25.4%)	
5	8 (12.3%)	104 (33.9%)	57 (41.3%)	
6	1 (1.5%)	52 (16.9%)	30 (21.7%)	
**HLA mismatches A (N (%))**				0.83
0	2 (11%)	9 (13%)	3 (8%)	
1	8 (44%)	30 (43%)	14 (37%)	
2	8 (44%)	31 (44%)	21 (55%)	
**HLA mismatches B (N (%))**				0.35
0	0 (0%)	4 (6%)	0 (0%)	
1	7 (39%)	18 (26%)	10 (26%)	
2	11 (61%)	48 (69%)	28 (74%)	
**HLA mismatches DR (N (%))**				<0.001
0	17 (51.5%)	8 (5.2%)	3 (4.3%)	
1	14 (42.4%)	93 (60.8%)	26 (37.1%)	
2	2 (6.1%)	52 (34.0%)	41 (58.6%)	

**HLA mismatches DQ (N (%))**				<0.001
0	16 (89%)	6 (9%)	0 (0%)	
1	2 (11%)	49 (71%)	6 (16%)	
2	0 (0%)	14 (20%)	32 (84%)	
**Current flow B cell (N (%))**				0.90
< 2 SD compatible	31 (96.9%)	146 (93.6%)	70 (94.6%)	
2-3 SD compatible	1 (3.1%)	5 (3.2%)	1 (1.4%)	
weak positive	0 (0.0%)	1 (0.6%)	1 (1.4%)	
positive	0 (0.0%)	4 (2.6%)	2 (2.7%)	
**Current flow T cell (N (%))**				0.48
< 2 SD compatible	30 (93.8%)	148 (94.9%)	72 (97.3%)	
2-3 SD compatible	2 (6.2%)	4 (2.6%)	0 (0.0%)	
weak positive	0 (0.0%)	1 (0.6%)	0 (0.0%)	
positive	0 (0.0%)	3 (1.9%)	2 (2.7%)	
**Induction treatment (N (%))**				
No Induction	0 (0.0%)	0 (0.0%)	0 (0.0%)	NA)
Basiliximab	11 (16.9%)	37 (12.1%)	12 (8.7%)	0.23
Thymoglobulin	53 (81.5%)	263 (85.7%)	125 (90.6%)	0.17
Steroid	65 (100.0%)	307 (100.0%)	137 (99.3%)	0.26
Alemtuzumab	0 (0.0%)	4 (1.3%)	2 (1.4%)	0.64
**<u>Outcomes</u>**				
**Proportion of Antibody Mediated Rejection of the subset of patients who underwent biopsy (N (%))**				0.057
No	20 (95.2%)	79 (74.5%)	35 (68.6%)	
Yes	1 (4.8%)	27 (25.5%)	16 (31.4%)	
**Proportion of rejection of the subset of patients who underwent biopsy (N (%))**				0.031
No	18 (85.7%)	62 (58.5%)	27 (52.9%)	
Yes	3 (14.3%)	44 (41.5%)	24 (47.1%)	
**Proportion of T-cell Mediated Rejection of the subset of patients who underwent biopsy (N (%))**				0.036

No	18 (85.7%)	72 (67.9%)	28 (54.9%)	
Yes	3 (14.3%)	34 (32.1%)	23 (45.1%)	
**Post-Transplant de novo Donor Specific Antibodies (N (%))**				<0.001
No	61 (93.8%)	252 (82.4%)	98 (72.1%)	
Yes	4 (6.2%)	54 (17.6%)	38 (27.9%)	
**Dd-cf-DNA elevation (N (%))**				0.20
No	55 (84.6%)	243 (79.2%)	102 (73.9%)	
Yes	10 (15.4%)	64 (20.8%)	36 (26.1%)	
**Delayed Graft Function (N (%))**				0.70
No	61 (93.8%)	289 (94.1%)	127 (92.0%)	
Yes	4 (6.2%)	18 (5.9%)	11 (8.0%)	
**Death (N (%))**				0.71
No	61 (93.8%)	294 (95.8%)	133 (96.4%)	
Yes	4 (6.2%)	13 (4.2%)	5 (3.6%)	
**Graft Loss (N (%))**				0.22
No	65 (100.0%)	305 (99.3%)	135 (97.8%)	
Yes	0 (0.0%)	2 (0.7%)	3 (2.2%)	

Values are expressed as mean (standard deviation), median (interquartile range), or number (%). Continuous variables were compared via t-tests or Mann-Whitney U tests. Categorical variables were compared via Chi-square tests.

Table S47: Patient characteristics for the donor-derived cell-free DNA cohort based on eplet mismatch load for HLA-DRB1 locus

	Zero Eplet Mismatch Group	Low Eplet Mismatch Group	High Eplet Mismatch Group	p-value
**N**	28	242	218	
**<u>Recipients' characteristics</u>**				
**Age (years), mean (SD)**	53.2 (15.9)	52.0 (14.8)	49.9 (15.2)	0.25
**Sex (N (%))**				0.86
male	16 (57.1%)	149 (61.6%)	136 (62.4%)	
female	12 (42.9%)	93 (38.4%)	82 (37.6%)	
**Race/ethnicity (N (%))**				0.57
White	23 (82.1%)	151 (64.8%)	143 (70.4%)	
Hispanic	4 (14.3%)	55 (23.6%)	33 (16.3%)	
African American	0 (0.0%)	6 (2.6%)	6 (3.0%)	
Asian	1 (3.6%)	10 (4.3%)	9 (4.4%)	
Other	0 (0.0%)	10 (4.3%)	12 (5.9%)	
Unknown	0 (0.0%)	1 (0.4%)	0 (0.0%)	
**Body Mass Index (kg/m2), mean (SD)**	28.1 (5.6)	28.8 (5.4)	29.4 (5.7)	0.36
**Dialysis vintage (months), median (IQR)**	24.6 (11.1-36.0)	33.8 (17.0-56.5)	35.8 (17.9-64.0)	0.042
**Cause of kidney failure (N (%))**				0.80
Diabetes mellitus	7 (25.0%)	78 (32.2%)	75 (34.4%)	
Hypertension/Vascular disease	5 (17.9%)	32 (13.2%)	30 (13.8%)	
Cystic kidney disease	2 (7.1%)	23 (9.5%)	20 (9.2%)	
Glomerulonephritis	6 (21.4%)	56 (23.2%)	57 (26.1%)	
Other/unknown	8 (28.6%)	53 (21.9%)	36 (16.5%)	
**Prior kidney transplant (N (%))**				0.23
No	27 (96.4%)	240 (99.2%)	217 (99.5%)	
Yes	1 (3.6%)	2 (0.8%)	1 (0.5%)	
**Multi-organ transplantation (N (%))**				0.29
Liver + kidney	0 (0.0%)	0 (0.0%)	0 (0.0%)	
Pancreas + kidney	0 (0.0%)	0 (0.0%)	2 (0.9%)	
Heart + kidney	0 (0.0%)	0 (0.0%)	0 (0.0%)	
None	28 (100.0%)	242 (100.0%)	216 (99.1%)	

Liver +Heart+Kidney	0 (0.0%)	0 (0.0%)	0 (0.0%)	
**CMV risk categories (N (%))**				0.31
Low	6 (21.4%)	57 (23.6%)	60 (27.5%)	
Intermediate	10 (35.7%)	108 (44.6%)	100 (45.9%)	
High	12 (42.9%)	65 (26.9%)	51 (23.4%)	
Unknown	0 (0.0%)	12 (5.0%)	7 (3.2%)	
**Was the donor organ pumped? (N (%))**				0.65
No	9 (36.0%)	79 (35.1%)	64 (31.1%)	
Yes	16 (64.0%)	146 (64.9%)	142 (68.9%)	
**En block kidney (N (%))**	0 (0.0%)	0 (0.0%)	0 (0.0%)	NA
**Dual kidney (N (%))**	0 (0.0%)	0 (0.0%)	0 (0.0%)	NA
**Transplantation characteristics**				
**Cold ischemic time (hours), median (IQR)**	17.1 (3.2-22.2)	11.5 (3.8-18.2)	13.5 (4.8-20.6)	0.12
**cPRA (%), median (IQR)**	0 (0-0)	0 (0-0)	0 (0-0)	0.40
**Donors' characteristics**				
**Age (years), mean (SD)**	36.3 (16.1)	38.0 (13.6)	39.4 (15.2)	0.42
**Sex (N (%))**				0.59
male	13 (46.4%)	123 (50.8%)	119 (54.6%)	
female	15 (53.6%)	119 (49.2%)	99 (45.4%)	
**Race/ethnicity**				0.79
White	26 (92.9%)	199 (82.2%)	175 (80.3%)	
African American	0 (0.0%)	5 (2.1%)	6 (2.8%)	
Asian	0 (0.0%)	5 (2.1%)	5 (2.3%)	
Other	1 (3.6%)	4 (1.7%)	3 (1.4%)	
Unknown	1 (3.6%)	29 (12.0%)	29 (13.3%)	
**Donor type (N (%))**				0.42
Living	11 (39.3%)	95 (39.3%)	73 (33.5%)	
Deceased	17 (60.7%)	147 (60.7%)	145 (66.5%)	
**Donor DCD (N (%))**				0.33
No	10 (58.8%)	72 (49.0%)	83 (57.2%)	
Yes	7 (41.2%)	75 (51.0%)	62 (42.8%)	
**KDPI, median (IQR)**	21 (11-47)	28 (15-45)	31 (16-54)	0.42
**Donor cause of death (N (%))**				0.63
Anoxia	7 (41.2%)	75 (51.0%)	74 (51.0%)	
Cerebrovascular/stroke	3 (17.6%)	24 (16.3%)	21 (14.5%)	
Head trauma	7 (41.2%)	42 (28.6%)	46 (31.7%)	

Central nervous system tumor	0 (0.0%)	0 (0.0%)	2 (1.4%)	
Other	0 (0.0%)	6 (4.1%)	2 (1.4%)	
**Donor terminal creatinine (mg/dl), median (IQR)0**	0.82 (0.69-1.05)	0.82 (0.67-1.03)	0.83 (0.66-1.01)	0.95
**Donor diabetes (N (%))**				0.51
No	28 (100.0%)	228 (95.4%)	206 (95.8%)	
Yes	0 (0.0%)	11 (4.6%)	9 (4.2%)	
**Donor hypertension (N (%))**				0.11
No	25 (89.3%)	212 (88.7%)	174 (82.1%)	
Yes	3 (10.7%)	27 (11.3%)	38 (17.9%)	
**Donor malignancy (N (%))**				0.52
No	28 (100.0%)	238 (98.3%)	212 (97.2%)	
Yes	0 (0.0%)	4 (1.7%)	6 (2.8%)	
**Immunological characteristics**				
**Number of HLA mismatches (HLA A, B and DR) (N (%))**				<0.001
1	6 (21.4%)	4 (1.7%)	1 (0.5%)	
2	8 (28.6%)	19 (7.9%)	2 (0.9%)	
3	7 (25.0%)	63 (26.0%)	17 (7.8%)	
4	5 (17.9%)	65 (26.9%)	48 (22.0%)	
5	2 (7.1%)	72 (29.8%)	86 (39.4%)	
6	0 (0.0%)	19 (7.9%)	64 (29.4%)	
**HLA mismatches A (N (%))**				0.89
0	1 (11%)	7 (12%)	6 (11%)	
1	3 (33%)	26 (45%)	21 (38%)	
2	5 (56%)	25 (43%)	29 (52%)	
**HLA mismatches B (N (%))**				0.52
0	0 (0%)	2 (3%)	1 (2%)	
1	3 (33%)	20 (34%)	12 (21%)	
2	6 (67%)	36 (62%)	43 (77%)	
**HLA mismatches DR (N (%))**				<0.001
0	10 (71.4%)	18 (14.2%)	0 (0.0%)	
1	3 (21.4%)	90 (70.9%)	39 (35.1%)	
2	1 (7.1%)	19 (15.0%)	72 (64.9%)	

**HLA mismatches DQ (N (%))**				<0.001
0	7 (78%)	11 (19%)	3 (5%)	
1	1 (11%)	34 (59%)	22 (40%)	
2	1 (11%)	13 (22%)	30 (55%)	
**Current flow B cell (N (%))**				0.95
< 2 SD compatible	13 (92.9%)	123 (94.6%)	107 (93.9%)	
2-3 SD compatible	1 (7.1%)	3 (2.3%)	3 (2.6%)	
weak positive	0 (0.0%)	1 (0.8%)	1 (0.9%)	
positive	0 (0.0%)	3 (2.3%)	3 (2.6%)	
**Current flow T cell (N (%))**				0.78
< 2 SD compatible	13 (92.9%)	124 (95.4%)	109 (95.6%)	
2-3 SD compatible	1 (7.1%)	3 (2.3%)	2 (1.8%)	
weak positive	0 (0.0%)	0 (0.0%)	1 (0.9%)	
positive	0 (0.0%)	3 (2.3%)	2 (1.8%)	
**Induction treatment (N (%))**				
No Induction	0 (0.0%)	0 (0.0%)	0 (0.0%)	NA
Basiliximab	2 (7.1%)	36 (14.9%)	22 (10.1%)	0.21
Thymoglobulin	24 (85.7%)	203 (83.9%)	192 (88.1%)	0.44
Steroid	28 (100.0%)	242 (100.0%)	217 (99.5%)	0.54
Alemtuzumab	1 (3.6%)	0 (0.0%)	5 (2.3%)	0.043
**<u>Outcomes</u>**				
**Proportion of Antibody Mediated Rejection of the subset of patients who underwent biopsy (N (%))**				0.072
No	8 (100%)	59 (80%)	60 (69%)	
Yes	0 (0%)	15 (20%)	27 (31%)	
**Proportion of rejection of the subset of patients who underwent biopsy (N (%))**				0.023
No	8 (100%)	47 (64%)	46 (53%)	
Yes	0 (0%)	27 (36%)	41 (47%)	
**Proportion of T-cell Mediated Rejection of the subset of patients who underwent biopsy (N (%))**				0.053

No	8 (100%)	51 (69%)	52 (60%)	
Yes	0 (0%)	23 (31%)	35 (40%)	
**Post-Transplant de novo Donor Specific Antibodies (N (%))**				0.009
No	27 (96.4%)	202 (84.2%)	165 (76.0%)	
Yes	1 (3.6%)	38 (15.8%)	52 (24.0%)	
**Dd-cf-DNA elevation (N (%))**				0.017
No	26 (92.9%)	196 (81.0%)	159 (72.9%)	
Yes	2 (7.1%)	46 (19.0%)	59 (27.1%)	
**Delayed Graft Function (N (%))**				0.48
No	27 (96.4%)	230 (95.0%)	202 (92.7%)	
Yes	1 (3.6%)	12 (5.0%)	16 (7.3%)	
**Death (N (%))**				0.098
No	28 (100.0%)	227 (93.8%)	212 (97.2%)	
Yes	0 (0.0%)	15 (6.2%)	6 (2.8%)	
**Graft Loss (N (%))**				0.72
No	28 (100.0%)	240 (99.2%)	215 (98.6%)	
Yes	0 (0.0%)	2 (0.8%)	3 (1.4%)	

Values are expressed as mean (standard deviation), median (interquartile range), or number (%). Continuous variables were compared via t-tests or Mann-Whitney U tests. Categorical variables were compared via Chi-square tests.

Table S48: Performance characteristics table of eplet mismatch load for HLA-A locus for donor-derived cell-free DNA

Cutpoint	Sensitivity	Specificity	Overall % correctly classified	LR+	LR-
(>= 1)	100.00%	0.00%	21.71%	1	
(>= 2)	98.88%	1.25%	22.44%	1.0012	0.9017
(>= 3)	97.75%	2.18%	22.93%	0.9993	1.0305
(>= 4)	96.63%	3.43%	23.66%	1.0006	0.9837
(>= 5)	95.51%	5.61%	25.12%	1.0118	0.8015
(>= 6)	93.26%	8.72%	27.07%	1.0217	0.7729
(>= 7)	87.64%	13.08%	29.27%	1.0083	0.9446
(>= 8)	85.39%	18.07%	32.68%	1.0423	0.8084
(>= 9)	82.02%	23.05%	35.85%	1.066	0.7798
(>= 10)	77.53%	30.22%	40.49%	1.111	0.7437
(>= 11)	74.16%	34.89%	43.41%	1.139	0.7407
(>= 12)	71.91%	39.25%	46.34%	1.1838	0.7156
(>= 13)	60.67%	44.55%	48.05%	1.0942	0.8828
(>= 14)	58.43%	49.22%	51.22%	1.1506	0.8446
(>= 15)	50.56%	53.27%	52.68%	1.082	0.9281
(>= 16)	43.82%	57.94%	54.88%	1.0419	0.9696
(>= 17)	39.33%	63.24%	58.05%	1.0698	0.9594
(>= 18)	35.96%	67.60%	60.73%	1.1098	0.9474
(>= 19)	31.46%	69.78%	61.46%	1.0411	0.9822
(>= 20)	29.21%	73.83%	64.15%	1.1164	0.9588
(>= 21)	28.09%	77.57%	66.83%	1.2523	0.927
(>= 22)	25.84%	79.44%	67.80%	1.2569	0.9335
(>= 23)	22.47%	82.87%	69.76%	1.3115	0.9356
(>= 24)	20.22%	83.18%	69.51%	1.2022	0.9591
(>= 25)	19.10%	86.29%	71.71%	1.3935	0.9375
(>= 26)	17.98%	88.47%	73.17%	1.5597	0.9271
(>= 27)	16.85%	91.28%	75.12%	1.9322	0.9109
(>= 28)	15.73%	92.83%	76.10%	2.1954	0.9077
(>= 29)	14.61%	93.77%	76.59%	2.3444	0.9107
(>= 30)	13.48%	95.02%	77.32%	2.7051	0.9106
(>= 31)	11.24%	95.64%	77.32%	2.5762	0.9281
(>= 32)	10.11%	96.88%	78.05%	3.2461	0.9278
(>= 33)	10.11%	97.20%	78.29%	3.6067	0.9248
(>= 34)	6.74%	97.51%	77.80%	2.7051	0.9564

(>= 35)	4.49%	98.75%	78.29%	3.6067	0.9671
(>= 36)	3.37%	98.75%	78.05%	2.705	0.9785
(>= 37)	3.37%	99.07%	78.29%	3.6067	0.9754
(>= 38)	2.25%	99.38%	78.29%	3.6067	0.9837
(>= 45)	2.25%	100.00%	78.78%	0.9775	
(>= 48)	1.12%	100.00%	78.54%	0.9888	
(> 48)	0.00%	100.00%	78.29%	1	

LR: Likelihood ratio

Table S49: Performance characteristics table of eplet mismatch load for HLA-B locus for donor-derived cell-free DNA

Cutpoint	Sensitivity	Specificity	Overall % correctly classified	LR+	LR-
(>= 1)	100.00%	0.00%	21.51%	1	
(>= 2)	98.94%	0.58%	21.74%	0.9952	1.8245
(>= 3)	97.87%	1.75%	22.43%	0.9961	1.2163
(>= 4)	95.74%	2.92%	22.88%	0.9862	1.4596
(>= 5)	94.68%	4.66%	24.03%	0.9931	1.1403
(>= 6)	93.62%	7.58%	26.09%	1.013	0.8421
(>= 7)	93.62%	10.79%	28.60%	1.0494	0.5917
(>= 8)	91.49%	16.33%	32.49%	1.0934	0.5213
(>= 9)	86.17%	23.32%	36.84%	1.1238	0.593
(>= 10)	84.04%	27.99%	40.05%	1.1671	0.5701
(>= 11)	77.66%	32.07%	41.88%	1.1432	0.6966
(>= 12)	71.28%	38.19%	45.31%	1.1532	0.7521
(>= 13)	58.51%	42.27%	45.77%	1.0136	0.9814
(>= 14)	54.26%	47.52%	48.97%	1.0339	0.9626
(>= 15)	44.68%	51.60%	50.11%	0.9232	1.072
(>= 16)	39.36%	55.98%	52.40%	0.8941	1.0833
(>= 17)	34.04%	61.22%	55.38%	0.8779	1.0773
(>= 18)	30.85%	65.89%	58.35%	0.9044	1.0495
(>= 19)	24.47%	69.10%	59.50%	0.7918	1.0931
(>= 20)	20.21%	73.47%	62.01%	0.7619	1.086
(>= 21)	18.09%	77.55%	64.76%	0.8056	1.0563
(>= 22)	13.83%	81.34%	66.82%	0.7412	1.0594
(>= 23)	10.64%	83.38%	67.73%	0.6402	1.0717
(>= 24)	7.45%	86.30%	69.34%	0.5435	1.0725
(>= 25)	7.45%	88.05%	70.71%	0.623	1.0512
(>= 26)	6.38%	90.09%	72.08%	0.6439	1.0392
(>= 27)	6.38%	91.55%	73.23%	0.755	1.0226
(>= 28)	5.32%	93.59%	74.60%	0.8293	1.0117
(>= 29)	3.19%	94.17%	74.60%	0.5473	1.028
(>= 30)	2.13%	95.34%	75.29%	0.4561	1.0266
(>= 31)	0.00%	95.92%	75.29%	0	1.0426
(>= 32)	0.00%	96.79%	75.97%	0	1.0331
(>= 33)	0.00%	97.38%	76.43%	0	1.0269
(>= 34)	0.00%	97.96%	76.89%	0	1.0208

(>= 35)	0.00%	98.25%	77.12%	0	1.0178
(>= 36)	0.00%	99.13%	77.80%	0	1.0088
(>= 43)	0.00%	99.71%	78.26%	0	1.0029
(> 43)	0.00%	100.00%	78.49%	1	

LR: Likelihood ratio

Table S50: Performance characteristics table of eplet mismatch load for HLA-C locus for donor-derived cell-free DNA

Cutpoint	Sensitivity	Specificity	Overall % correctly classified	LR+	LR-
(>= 1)	100.00%	0.00%	20.88%	1	
(>= 2)	97.65%	4.35%	23.83%	1.0209	0.5412
(>= 3)	95.29%	7.76%	26.04%	1.0332	0.6061
(>= 4)	92.94%	12.73%	29.48%	1.065	0.5544
(>= 5)	87.06%	18.32%	32.68%	1.0659	0.7063
(>= 6)	81.18%	24.22%	36.12%	1.0713	0.7771
(>= 7)	71.76%	31.37%	39.80%	1.0456	0.9002
(>= 8)	65.88%	41.30%	46.44%	1.1224	0.826
(>= 9)	63.53%	48.45%	51.60%	1.2323	0.7528
(>= 10)	61.18%	56.52%	57.49%	1.4071	0.6869
(>= 11)	52.94%	63.66%	61.43%	1.457	0.7392
(>= 12)	42.35%	69.25%	63.64%	1.3775	0.8324
(>= 13)	32.94%	74.22%	65.60%	1.278	0.9035
(>= 14)	29.41%	77.95%	67.81%	1.3339	0.9056
(>= 15)	23.53%	80.75%	68.80%	1.222	0.9471
(>= 16)	20.00%	83.85%	70.52%	1.2385	0.9541
(>= 17)	16.47%	86.34%	71.74%	1.2053	0.9675
(>= 18)	14.12%	89.44%	73.71%	1.337	0.9602
(>= 19)	12.94%	91.30%	74.94%	1.4882	0.9535
(>= 20)	11.76%	93.79%	76.66%	1.8941	0.9408
(>= 21)	5.88%	94.72%	76.17%	1.1142	0.9936
(>= 22)	2.35%	96.27%	76.66%	0.6314	1.0143
(>= 23)	0.00%	96.89%	76.66%	0	1.0321
(>= 25)	0.00%	98.14%	77.64%	0	1.019
(>= 27)	0.00%	98.45%	77.89%	0	1.0158
(>= 28)	0.00%	99.07%	78.38%	0	1.0094
(>= 29)	0.00%	99.38%	78.62%	0	1.0062
(> 29)	0.00%	100.00%	79.12%	1	

LR: Likelihood ratio

Table S51: Performance characteristics table of eplet mismatch load for HLA-DQA1 locus for donor-derived cell-free DNA

Cutpoint	Sensitivity	Specificity	Overall % correctly classified	LR+	LR-
(>= 1)	100.00%	0.00%	22.48%	1	
(>= 2)	94.03%	8.66%	27.85%	1.0294	0.6896
(>= 3)	79.10%	26.41%	38.26%	1.0749	0.7913
(>= 4)	61.19%	47.62%	50.67%	1.1682	0.8149
(>= 5)	43.28%	61.04%	57.05%	1.1109	0.9292
(>= 6)	31.34%	71.00%	62.08%	1.0806	0.9671
(>= 7)	25.37%	77.49%	65.77%	1.1272	0.9631
(>= 8)	8.96%	85.28%	68.12%	0.6084	1.0676
(>= 9)	8.96%	88.74%	70.81%	0.7956	1.0259
(>= 10)	7.46%	92.21%	73.15%	0.9577	1.0036
(>= 11)	5.97%	94.81%	74.83%	1.1493	0.9918
(>= 12)	5.97%	96.10%	75.84%	1.5323	0.9784
(>= 13)	2.99%	97.40%	76.17%	1.1493	0.996
(>= 14)	0.00%	98.27%	76.17%	0	1.0176
(>= 15)	0.00%	99.57%	77.18%	0	1.0043
(> 15)	0.00%	100.00%	77.52%	1	

LR: Likelihood ratio

Table S52: Performance characteristics table of eplet mismatch load for HLA-DQB1 locus for donor-derived cell-free DNA

Cutpoint	Sensitivity	Specificity	Overall % correctly classified	LR+	LR-
(>= 1)	100.00%	0.00%	22.47%	1	
(>= 2)	97.00%	4.93%	25.62%	1.0203	0.6088
(>= 3)	93.00%	8.70%	27.64%	1.0186	0.805
(>= 4)	90.00%	13.04%	30.34%	1.035	0.7667
(>= 5)	81.00%	17.10%	31.46%	0.9771	1.111
(>= 6)	73.00%	22.32%	33.71%	0.9397	1.2097
(>= 7)	59.00%	32.17%	38.20%	0.8699	1.2743
(>= 8)	54.00%	39.13%	42.47%	0.8871	1.1756
(>= 9)	49.00%	46.38%	46.97%	0.9138	1.0997
(>= 10)	48.00%	52.17%	51.24%	1.0036	0.9967
(>= 11)	42.00%	60.87%	56.63%	1.0733	0.9529
(>= 12)	38.00%	67.25%	60.67%	1.1602	0.922
(>= 13)	36.00%	70.43%	62.70%	1.2176	0.9086
(>= 14)	31.00%	75.36%	65.39%	1.2582	0.9156
(>= 15)	25.00%	80.00%	67.64%	1.25	0.9375
(>= 16)	22.00%	83.48%	69.66%	1.3316	0.9344
(>= 17)	18.00%	85.51%	70.34%	1.242	0.959
(>= 18)	16.00%	88.70%	72.36%	1.4154	0.9471
(>= 19)	13.00%	91.30%	73.71%	1.495	0.9529
(>= 20)	13.00%	91.59%	73.93%	1.5466	0.9498
(>= 21)	12.00%	92.75%	74.61%	1.656	0.9487
(>= 22)	9.00%	94.20%	75.06%	1.5525	0.966
(>= 23)	6.00%	95.94%	75.73%	1.4786	0.9798
(>= 24)	5.00%	96.52%	75.96%	1.4375	0.9842
(>= 25)	4.00%	97.10%	76.18%	1.38	0.9887
(>= 26)	4.00%	97.39%	76.40%	1.5333	0.9857
(>= 27)	3.00%	97.39%	76.18%	1.15	0.996
(>= 28)	3.00%	98.26%	76.85%	1.725	0.9872
(>= 29)	2.00%	98.55%	76.85%	1.38	0.9944
(>= 30)	2.00%	98.84%	77.08%	1.725	0.9915
(>= 31)	0.00%	99.42%	77.08%	0	1.0058
(>= 32)	0.00%	99.71%	77.30%	0	1.0029
(> 32)	0.00%	100.00%	77.53%	1	

LR: Likelihood ratio

Table S53: Performance characteristics table of eplet mismatch load for HLA-DRB1 locus for donor-derived cell-free DNA

Cutpoint	Sensitivity	Specificity	Overall % correctly classified	LR+	LR-
(>= 1)	100.00%	0.00%	22.83%	1	
(>= 2)	99.05%	2.54%	24.57%	1.0162	0.3757
(>= 3)	98.10%	5.63%	26.74%	1.0395	0.3381
(>= 4)	96.19%	6.76%	27.17%	1.0317	0.5635
(>= 5)	96.19%	9.58%	29.35%	1.0638	0.3978
(>= 6)	96.19%	10.42%	30.00%	1.0738	0.3655
(>= 7)	93.33%	13.80%	31.96%	1.0828	0.483
(>= 8)	90.48%	18.03%	34.57%	1.1037	0.5283
(>= 9)	85.71%	20.28%	35.22%	1.0752	0.7044
(>= 10)	82.86%	25.92%	38.91%	1.1184	0.6615
(>= 11)	80.00%	32.11%	43.04%	1.1784	0.6228
(>= 12)	76.19%	38.87%	47.39%	1.2464	0.6125
(>= 13)	72.38%	43.66%	50.22%	1.2848	0.6326
(>= 14)	67.62%	47.32%	51.96%	1.2837	0.6842
(>= 15)	64.76%	51.27%	54.35%	1.3289	0.6873
(>= 16)	56.19%	55.21%	55.43%	1.2546	0.7935
(>= 17)	49.52%	60.00%	57.61%	1.2381	0.8413
(>= 18)	45.71%	62.54%	58.70%	1.2202	0.8681
(>= 19)	38.10%	68.45%	61.52%	1.2075	0.9044
(>= 20)	33.33%	70.99%	62.39%	1.1489	0.9392
(>= 21)	29.52%	74.08%	63.91%	1.1392	0.9513
(>= 22)	24.76%	76.06%	64.35%	1.0342	0.9892
(>= 23)	24.76%	78.31%	66.09%	1.1416	0.9608
(>= 24)	21.90%	81.13%	67.61%	1.1606	0.9626
(>= 25)	20.95%	83.10%	68.91%	1.2397	0.9513
(>= 26)	20.00%	85.63%	70.65%	1.3922	0.9342
(>= 27)	18.10%	87.32%	71.52%	1.4275	0.9379
(>= 28)	16.19%	88.73%	72.17%	1.4369	0.9445
(>= 29)	10.48%	89.86%	71.74%	1.0331	0.9963
(>= 30)	8.57%	90.99%	72.17%	0.9509	1.0049
(>= 31)	5.71%	92.11%	72.39%	0.7245	1.0236
(>= 32)	4.76%	92.68%	72.61%	0.6502	1.0276
(>= 33)	4.76%	93.80%	73.48%	0.7684	1.0153
(>= 34)	2.86%	94.08%	73.26%	0.483	1.0325

(>= 35)	2.86%	94.37%	73.48%	0.5071	1.0294
(>= 36)	2.86%	95.21%	74.13%	0.5966	1.0203
(>= 37)	1.90%	95.21%	73.91%	0.3978	1.0303
(>= 38)	1.90%	96.62%	75.00%	0.5635	1.0153
(>= 39)	1.90%	96.90%	75.22%	0.6147	1.0123
(>= 40)	1.90%	97.46%	75.65%	0.7513	1.0065
(>= 41)	1.90%	97.75%	75.87%	0.8452	1.0036
(>= 42)	1.90%	98.03%	76.09%	0.966	1.0007
(>= 43)	1.90%	98.87%	76.74%	1.6905	0.9921
(>= 44)	1.90%	99.15%	76.96%	2.254	0.9893
(>= 47)	1.90%	99.44%	77.17%	3.3809	0.9865
(>= 48)	0.95%	99.44%	76.96%	1.6905	0.9961
(>= 52)	0.00%	99.72%	76.96%	0	1.0028
(> 52)	0.00%	100.00%	77.17%	1	

LR: Likelihood ratio

Table S54: Association of class I HLA Eplet mismatch load and elevation of donor-derived cell-free DNA using Cox proportional regression models

Model		Hazard Ratio (HR)	95% Confidence Interval (CI) of HR	p-value		Hazard Ratio (HR)	95% Confidence Interval (CI) of HR	p-value		Hazard Ratio (HR)	95% Confidence Interval (CI) of HR	p-value
UNADJUSTED	**N=452**				**N=455**				**N=446**			
	**HLA-A** (+1 eplet increase)	1.023	1.002-1.044	0.034	**HLA-B** (+1 eplet increase)	1.002	0.977-1.029	0.828	**HLA-C** (+1 eplet increase)	1.026	0.994-1.060	0.115
	**HLA-A** (ref=Zero)				**HLA-B** (ref=Zero)				**HLA-C** (ref=Zero)			
	**Low**	1.784	0.721-4.412	0.210	**Low**	1.446	0.330-6.332	0.625	**Low**	0.714	0.330-1.546	0.392
	**High**	3.293	1.185-9.149	0.022	**High**	2.637	0.647-10.744	0.176	**High**	1.351	0.641-2.844	0.429
ADJUSTED*	**N=431**				**N=434**				**N=428**			
	**HLA-A** (+1 eplet increase)	1.018	0.996-1.042	0.113	**HLA-B** (+1 eplet increase)	1.005	0.977-1.034	0.709	**HLA-C** (+1 eplet increase)	1.024	0.989-1.060	0.186
	**HLA-A** (ref=Zero)				**HLA-B** (ref=Zero)				**HLA-C** (ref=Zero)			
	**Low**	2.037	0.730-5.680	0.174	**Low**	1.574	0.343-7.223	0.560	**Low**	0.750	0.331-1.699	0.490
	**High**	3.211	0.989-10.433	0.052	**High**	2.946	0.688-12.613	0.145	**High**	1.286	0.582-2.843	0.535

*adjusted for: recipient age, recipient sex, recipient race/ethnicity, recipient BMI, dialysis vintage, cause of end stage renal disease, preemptive kidney transplantation, history of prior kidney transplantation, multi-organ transplantation, type of induction therapy, donor age, donor sex, donor race/ethnicity, donor type [living vs. deceased], cold ischemic time, cytomegalovirus risk status, cPRA at allocation

Table S55: Association of class II HLA Eplet mismatch load and elevation of donor-derived cell-free DNA using Cox proportional regression models

Model		Hazard Ratio (HR)	95% Confidence Interval (CI) of HR	p-value		Hazard Ratio (HR)	95% Confidence Interval (CI) of HR	p-value		Hazard Ratio (HR)	95% Confidence Interval (CI) of HR	p-value
UNADJUSTED	**N=488**				**N=377**				**N=510**			
	**HLA-DRB1** (+1 eplet increase)	1.019	1.001-1.037	0.038	**HLA-DQA1** (+1 eplet increase)	1.060	0.993-1.132	0.080	**HLA-DQB1** (+1 eplet increase)	1.015	0.989-1.043	0.267
	**HLA-DRB1** (ref=Zero)				**HLA-DQA1** (ref=Zero)				**HLA-DQB1** (ref=Zero)			
	**Low**	2.974	0.722-12.251	0.131	**Low**	1.513	0.730-3.138	0.266	**Low**	1.282	0.658-2.497	0.465
	**High**	4.191	1.024-17.155	0.046	**High**	2.200	1.101-4.397	0.026	**High**	1.686	0.837-3.397	0.144
ADJUSTED*	**N=464**				**N=360**				**N=486**			
	**HLA-DRB1** (+1 eplet increase)	1.019	0.999-1.039	0.060	**HLA-DQA1** (+1 eplet increase)	1.058	0.985-1.136	0.124	**HLA-DQB1** (+1 eplet increase)	1.011	0.983-1.040	0.434
	**HLA-DRB1** (ref=Zero)				**HLA-DQA1** (ref=Zero)				**HLA-DQB1** (ref=Zero)			
	**Low**	4.564	0.954-21.835	0.057	**Low**	1.418	0.663-3.030	0.368	**Low**	1.387	0.684-2.814	0.364
	**High**	6.150	1.276-29.637	0.024	**High**	2.121	1.028-4.377	0.042	**High**	1.744	0.827-3.677	0.144

*adjusted for: recipient age, recipient sex, recipient race/ethnicity, recipient BMI, dialysis vintage, cause of end stage renal disease, preemptive kidney transplantation, history of prior kidney transplantation, multi-organ transplantation, type of induction therapy,

donor age, donor sex, donor race/ethnicity, donor type [living vs. deceased], cold ischemic time, cytomegalovirus risk status, cPRA at allocation
